# α‐Halocarbonyls as a Valuable Functionalized Tertiary Alkyl Source

**DOI:** 10.1002/open.202400108

**Published:** 2024-07-11

**Authors:** Takashi Nishikata

**Affiliations:** ^1^ Graduate School of Science and Engineering Yamaguchi University 2-16-1 Tokiwadai Ube Yamaguchi 755-8611 Japan

**Keywords:** tertiary alkyl radical, tertiary alkylation, cross-coupling, asymmetric reaction, addition

## Abstract

This review introduces the synthetic organic chemical value of α‐bromocarbonyl compounds with tertiary carbons. This α‐bromocarbonyl compound with a tertiary carbon has been used primarily only as a radical initiator in atom transfer radical polymerization (ATRP) reactions. However, with the recent development of photo‐radical reactions (around 2010), research on the use of α‐bromocarbonyl compounds as tertiary alkyl radical precursors became popular (around 2012). As more examples were reported, α‐bromocarbonyl compounds were studied not only as radicals but also for their applications in organometallic and ionic reactions. That is, α‐bromocarbonyl compounds act as nucleophiles as well as electrophiles. The carbonyl group of α‐bromocarbonyl compounds is also attractive because it allows the skeleton to be converted after the reaction, and it is being applied to total synthesis. In our survey until 2022, α‐bromocarbonyl compounds can be used to perform a full range of reactions necessary for organic synthesis, including multi‐component reactions, cross‐coupling, substitution, cyclization, rearrangement, stereospecific reactions, asymmetric reactions. α‐Bromocarbonyl compounds have created a new trend in tertiary alkylation, which until then had limited reaction patterns in organic synthesis. This review focuses on how α‐bromocarbonyl compounds can be used in synthetic organic chemistry.

## Graphical abstract

In this review, we introduce α‐bromocarbonyl compounds as tertiary alkyl sources that act as both nucleophiles and electrophiles. Although α‐bromocarbonyl compounds have been used as radical initiators in the past, their use as organic synthesis reactants only began to take off around 2012.

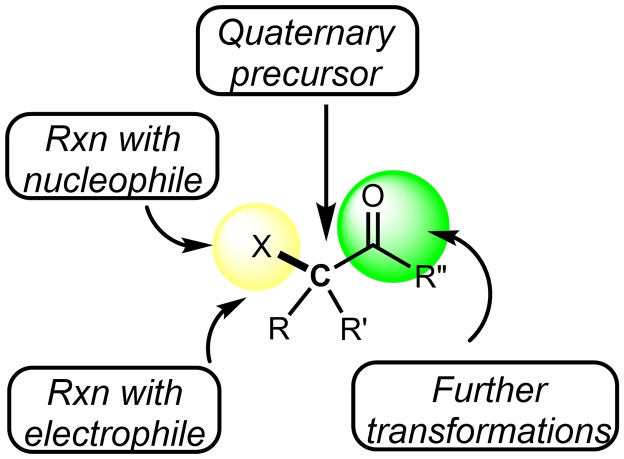

## 1 Introduction

**Figure 1:**
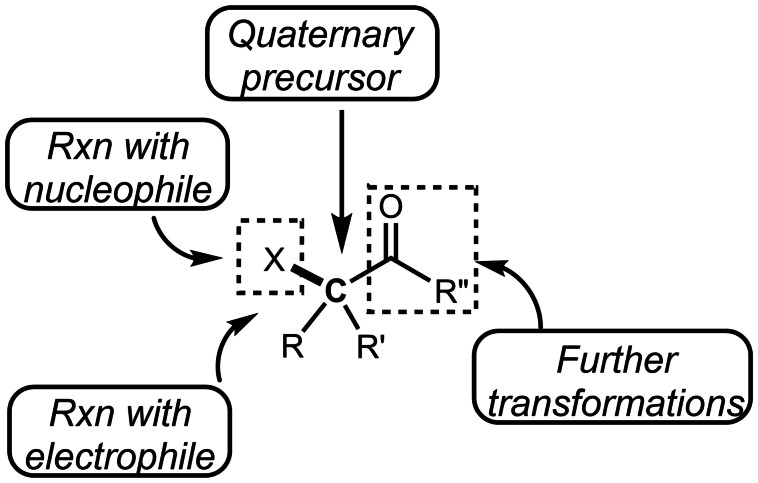
Value of an α‐bromocarbonyl compound.

Quaternary carbon or tetrasubstituted carbon compounds are among the most difficult carbon skeletons to synthesize because of their highly congested structure.[^1^, ^2^, ^3^, ^4^] To construct them, tertiary alkylation is one of the most convenient options in synthetic organic chemistry. But a practical tert‐alkylation is limited to a cation reaction, such as S_N_1 or Friedel‐Crafts reaction. In this reaction, non‐functionalized *tert*‐alkyl halides are employed as an alkyl source. This reaction is convenient in the sense that it introduces a simple alkyl group, but in order to synthesize intermediates with a wide variety of chemical transformations, it is necessary to introduce a functional group such as carbonyl to the alkyl group beforehand. In this context, α‐halocarbonyls are one of the most attractive tert‐alkyl sources. The synthetic chemistry of α‐halocarbonyls has been dominated by malonate derivatives and has been poor in terms of the diversity of carbon skeletons that can be synthesized. However, in the last decade, a wide variety of α‐halocarbonyl structures have become available via radical reactions, which have led to the synthesis of a great variety of functionalized quaternary carbon or tetrasubstituted carbon compounds. This is because previous researches have shown that α‐halocarbonyl compounds are 1) an electrophile, 2) a nucleophile, 3) quaternary precursor, and 4) able to be converted to desired quaternary structure via transformation of α‐carbonyl group (Figure 1).

The current review will focus on the synthetic values of α‐halocarbonyls mainly via transition metal catalyzed or photocatalyzed reactions, and radical reactions (partly includes recent ionic reactions) with their key mechanistic features with a few substrate scopes and describe their development until the end of 2022. Thus, this review does not highlight α‐halocarbonyls as a carbanion including Reformatzky and an enolate reaction,[^5^, ^6^, ^7^, ^8^, ^9^, ^10^, ^11^, ^12^, ^13^, ^14^, ^15^, ^16^, ^17^, ^18^, ^19^, ^20^, ^21^, ^22^, ^23^, ^24^, ^25^, ^26^, ^27^, ^28^, ^29^, ^30^, ^31^, ^32^, ^33^, ^34^, ^35^, ^36^, ^37^, ^38^, ^39^, ^40^, ^41^, ^42^] aziridinone chemistry,[^43^, ^44^, ^45^, ^46^, ^47^, ^48^, ^49^, ^50^, ^51^, ^52^, ^53^, ^54^, ^55^, ^56^, ^57^, ^58^, ^59^, ^60^, ^61^, ^62^, ^63^, ^64^, ^65^] simple substitution reactions including intermolecular[^66^, ^67^, ^68^, ^69^, ^70^, ^71^, ^72^, ^73^, ^74^, ^75^, ^76^, ^77^, ^78^, ^79^] or intramolecular reactions,[^80^, ^81^, ^82^, ^83^, ^84^, ^85^, ^86^, ^87^, ^88^, ^89^] and miscellaneous ionic reactions.[^90^, ^91^, ^92^, ^93^, ^94^, ^95^, ^96^, ^97^, ^98^] Although some reviews on α‐halocarbonyl chemistry[^99^, ^100^, ^101^, ^102^, ^103^] are reported, there are no comprehensive reviews of α‐halocarbonyls for *tert*‐alkylations. We have divided the review based on the organic reaction modes, and did not describe theoretical details of electron transfer process[^104^, ^105^, ^106^, ^107^, ^108^, ^109^] to generate radicals in this review.

## 2 Value of α‐Halocarbonyl Compounds as a Tertiary Alkyl Source

α‐Halocarbonyls have been known to be a good radical precursor, and the resulting radical is used for an initiator of atom transfer radical polymerization (ATRP), a reagent for atom transfer radical addition or cyclization (ATRA or ATRC).[^110^, ^111^, ^112^, ^113^, ^114^, ^115^, ^116^, ^117^, ^118^, ^119^] These are one of the representative radical reactions in organic synthesis.

**Scheme 1:**
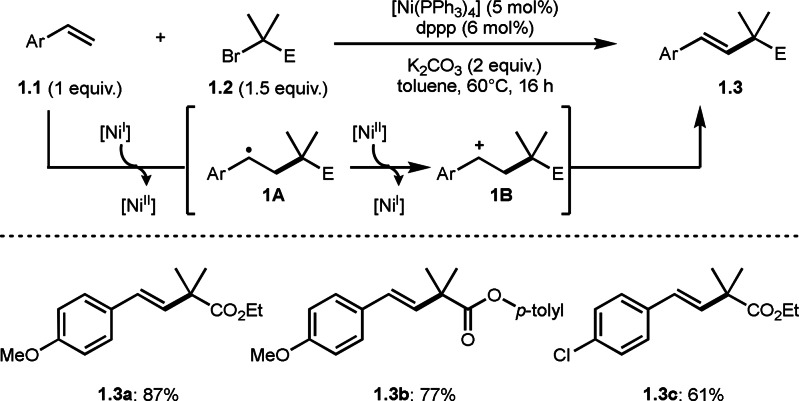
Lei's Ni‐catalyzed tert‐alkylation with terminal olefin.

On the other hand, a highly impact reaction using α‐halocarbonyls was reported by Lei group. They employed α‐halocarbonyls as a tertiary alkyl source for the Mizoroki‐Heck like cross‐coupling reaction (Scheme 1).^[120]^ The Mizoroki‐Heck (M–H) reaction is undoubtedly useful reaction to synthesize internal olefins from a terminal olefin and an organic halide in the presence of a Pd catalyst.[^121^, ^122^] And this reaction is mainly suitable for the loading of aryl and 1‐alkenyl groups. Because the reaction with alkyl halide is problematic after oxidative addition, in which undesirable β‐hydrogen elimination from the resulting alkyl metal species occurs. Therefore, alkyl halide, especially tertiary alkyl halide, was not suitable for this purpose.[^123^, ^124^, ^125^] Lei′s results, shown in Scheme 1, opened up a new trend in tertiary alkylation cross‐coupling chemistry.

The Lei's tert‐alkylation reaction conditions were: Ni(PPh_3_)_4_/dppp (1,3‐bis(diphenylphosphino)propane), K_2_CO_3_ in toluene at 60 °C. Under the optimal conditions, god yields of **1.3** were obtained. Their catalytic cycle starts from the reaction of Ni^I^ and **1.2**, in which single‐electron transfer (SET) from Ni^I^ to **1.2** occurs to generate α‐radical (tertiary alkyl radical species). As the result, Heck‐like olefination product (**1.3**) is obtained after the addition of α‐radical to **1.1** followed by Ni^II^ oxidation of **1A**/proton elimination of **1B**.

**Scheme 2:**
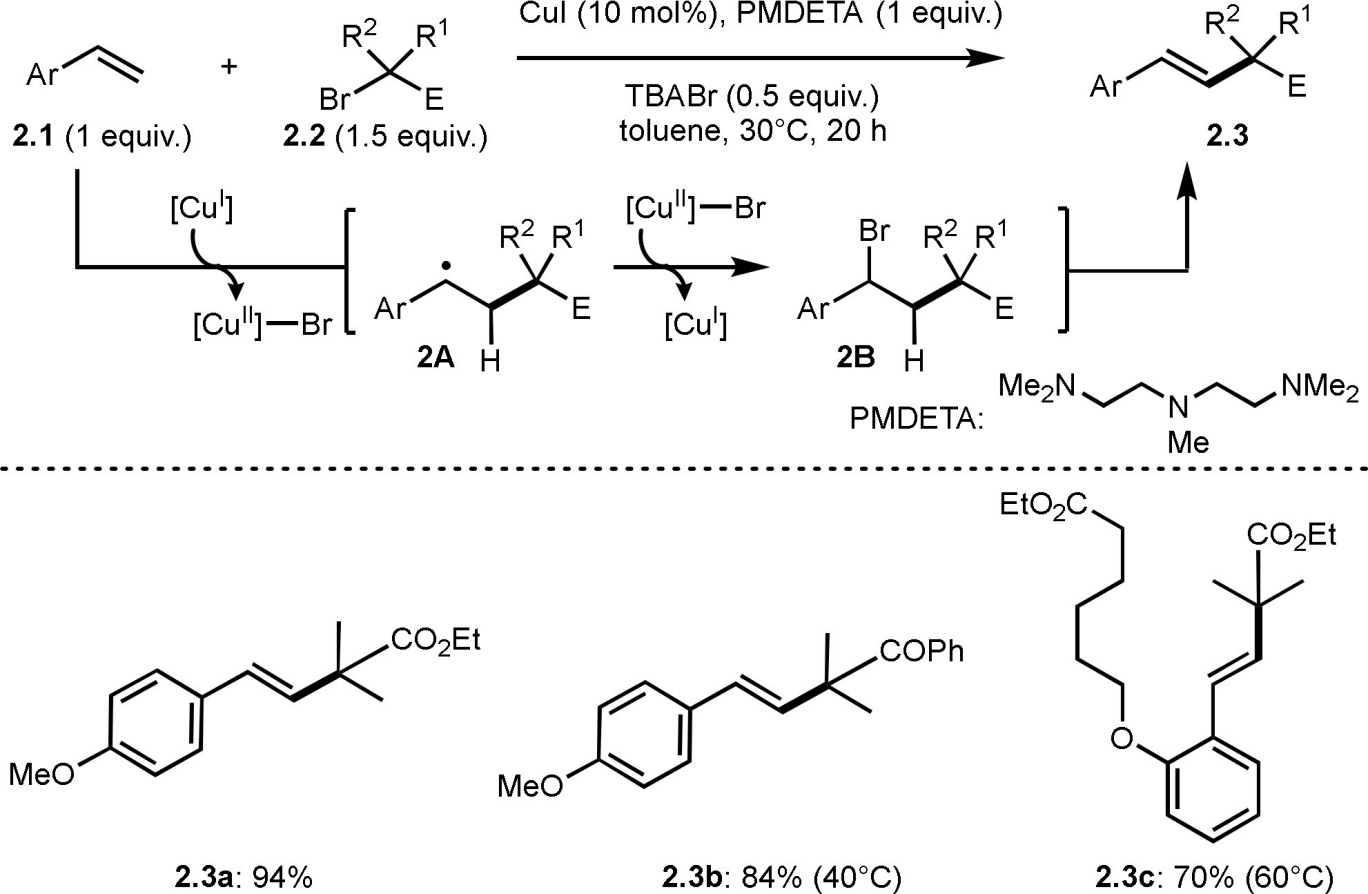
Nishikata's Cu‐catalyzed *tert*‐alkylation with terminal olefin.

Later, Nishikata's group reported Cu‐catalyzed Mizoroki‐Heck like olefination by using α‐halocarbonyls (Scheme 2).^[126]^ This reaction is regarded as the first step of the ATRP, with polymerization terminating in the first step and hydrogen elimination products, that is Heck‐like olefination, being obtained instead. In ATRP, PMDETA was used as a ligand for Cu salt.^[114]^ On the other hand, Nishikata's conditions employed PMDETA as both a ligand and a base. Instead of PMDETA (*N,N,N′,N′′,N′′*‐pentamethyldiethylenetriamine), TPMA (Tris(2‐pyridylmethyl)amine) as a ligand and *i*‐Pr_2_MeN as a base gave highly efficient reaction.^[127]^ This powerful radical reaction enabled to synthesize highly congested tertiary alkylated structures (**2.3**). The reaction starts with the generation of α‐radicals from the reaction between Cu^I^ and **2.2**. They checked the radical generation step by adding TEMPO to this reaction mixture. And alkylated TEMPO was isolated, which is the proof of the existence of the tertiary‐alkyl radical species during the reaction. After the generation of α‐radical, it adds to **2.1** to give the radical intermediate **2A**. Then, **2A** reacts with the Br−Cu^II^ species to produce ATRA product **2B**. Finally, **2B** undergoes E2 type elimination with PMDETA to give **2.3**. The Mizoroki‐Heck is involving oxidative addition/β‐hydrogen elimination in the catalytic cycle, whereas current reaction is not involving the steps. Therefore, they called this reaction atom transfer radical substitution (ATRS).^[128]^

## 3 Metal‐Catalyzed Reaction

A transition metal complex has diverse redox potentials. Especially, reduction ability of metal is suitable for SET reaction. A single electron transfers from a metal to α‐bromocarbonyl compound, which gives α‐*tert*‐alkyl radical. Under these conditions, various addition reactions were reported. This section introduces the reaction including additions, cyclizations, and couplings of α‐tert‐alkyl radicals under a transition metal catalyst system.

### 3.1 Addition Reaction

Addition reaction of radical species is a fundamental aspect in radical chemistry. But, due to the steric hinderance, addition of tert‐alkyl radical is difficult, compared with primary and secondary alkyl radicals. Consequently, there are few reports on diverse radical addition reactions catalyzed by transition metal salts.

**Scheme 3:**
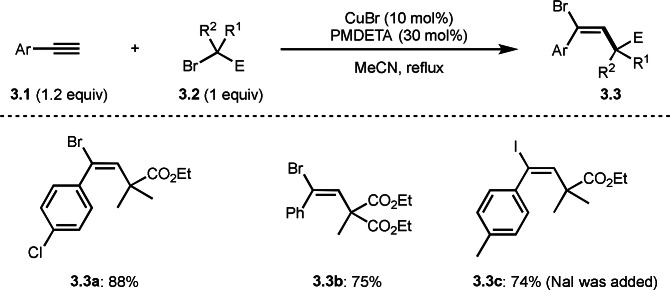
Trans ATRA.

ATRA[^111^, ^117^, ^118^] is one of the fundamental reactions in radical chemistry. But a reaction with alkyne and α‐radical from α‐bromocarbonyl produces *E‐* or *Z‐*adduct. Zhu's group reported that Cu‐catalyzed reaction of terminal alkyne (**3.1**) and α‐bromocarbonyl compound (**3.2**) produced *E*‐adduct (**3.3**), in which trans ATRA occurred (Scheme 3).^[129]^ Structures with tert‐alkyl and aryl groups pointing in the same direction are sterically very unstable, though, selectivities were perfect. The reason for this trans addition is not described but PMDETA ligand may plays an important role in the selectivity. Intriguingly, Hu's group previously reported cis ATRA using α‐bromocarbonyl possessing sec‐alkyl structure under Cu/Pybox catalyst system, and they described that a Cu ligation to C=O group affects the selectivity.^[130]^ Zhu also extended this reaction to tandem reaction system. When excess amount of **3.1** was used in the presence of Pd/Cu catalyst system, ATRA/Sonogashira coupling occurred.

**Scheme 4:**
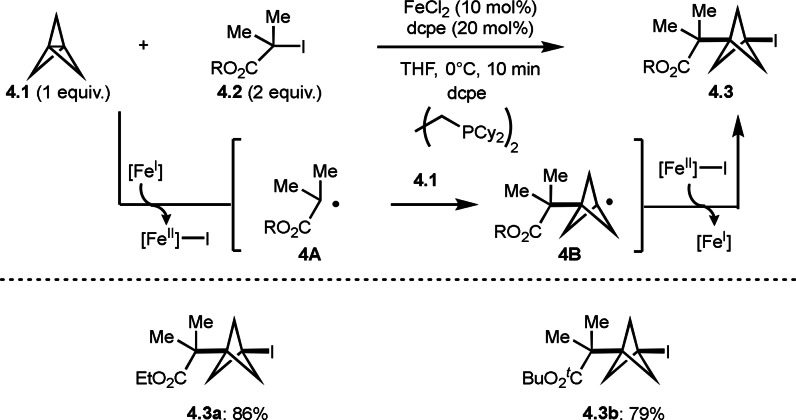
ATRA of [1.1.1]propellane **4.1**.

Bicyclo[1.1.1]pentane (BCP), as a bioisostere (para‐substituted arene, tert‐butyl motif and alkyne), has recently attracted attention in several drug discovery areas. Gutierrez's group discovered that the reaction of [1.1.1]propellane (**4.1**) and α‐bromocarboxamide (**4.2**) underwent ATRA like reaction to produce BCP (**4.3**) possessing both tert‐alkyl group and bromine (Scheme 4).^[131a]^ In this reaction, a Fe^I^ salt acted as an active catalyst, in which SET from Fe^I^ to **4.2** occurred to produce α‐radical **4A**. **4A** smoothly reacted with **4.1** to produce **4B**, and **4.3** was obtained from the reaction of [Fe^II^]−I and **4B**.

**Scheme 5:**
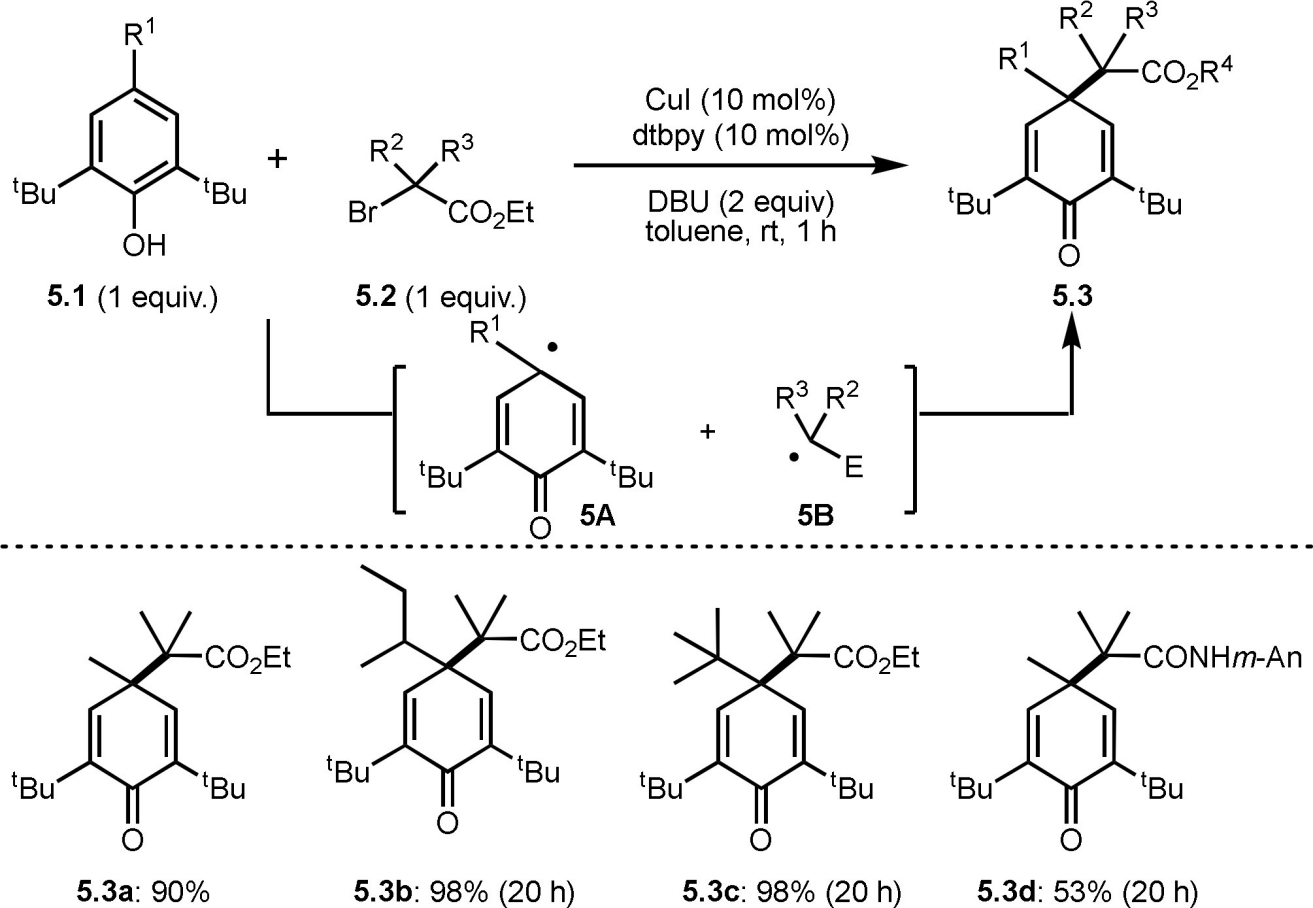
Dearomative couplings.

In general, aromatic rings are less suitable for radical addition reactions than C−C multiple bonds. This is because the product loses its aromaticity after addition to the aromatic ring. Nishikata's group discovered dearomative addition of tert‐alkyl radical from α‐bromocarbonyl compound (**5.2**) to BHT (**5.1**: butylhydroxytoluene) in the presence of Cu catalyst at room temperature (Scheme 5).^[132]^ Under their conditions, highly congested cyclic dienones (**5.3 a**–**d**) with two contiguous quaternary carbon centers were obtained. In this reaction, a radical‐radical (**5A** and **5B**) coupling occurred. SET from Cu^I^ to **5.2** occurred to produce α‐radical **5B**. **5A** was generated from the reaction of **5.1** and DBU (1,8‐Diazabicyclo[5.4.0]‐7‐undecene) followed by Cu^II^ oxidation. They also detected homocoupling of **5A** as a side product.

**Scheme 6:**
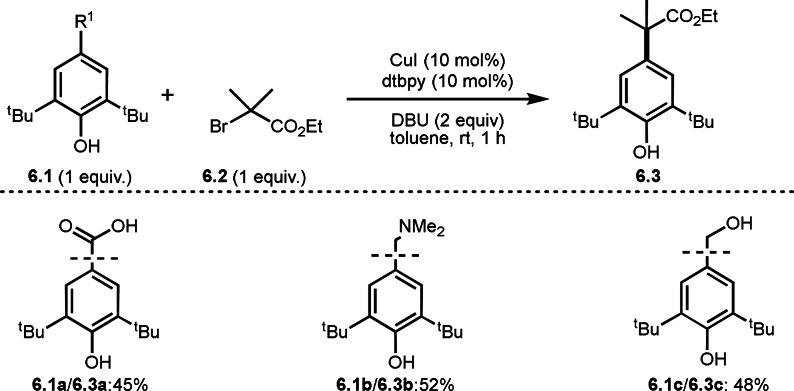
C−C cleavage couplings.

Interestingly, BHT possessing −CO_2_H, −CH_2_NMe_2_, or −CH_2_OH underwent C−C cleavage couplings instead of dearomative addition under the same conditions (Scheme 6). The reaction mechanism is not clear but BHT structure is very important. Indeed, this C−C cleavage reaction did not occur when **6.1** without ^t^Bu groups were employed.

### 3.2 Three‐Component Reaction

Three‐component reactions are a useful methodology for synthesizing complex molecules. However, radical three‐component reactions must consider the reactivity of the reaction partners because there are multiple substrates that can react with the radical species. This is because different radical species react in different orders, resulting in the formation of multiple products. Furthermore, the three‐component reactions introduced here are extremely difficult to control because the reactions involve not only radical species but also cations and organometallic species. Solving these problems is the key to the success of radical three‐component reactions.

**Scheme 7:**
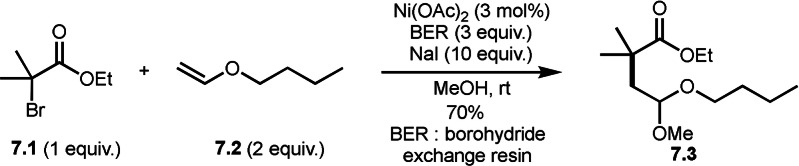
The reaction of α‐bromocarbonyl, vinyl ether and methanol.

BER (borohydride exchange resin), acting as a good electron or hydrogen source is easily removable. Yoon's group reported the reaction of α‐bromocarbonyl **7.1**, vinyl ether **7.2** and methanol in the presence of Ni catalyst (Scheme 7).[^133^, ^134^] The reaction gave three‐component coupling product **7.3** in 70 % yield. The reaction mechanism is not described in this paper, but Ni^I^ species may play an important role for the generation of α‐radicals from **7.1**. The reaction mainly employed prim‐ and sec‐alkyl groups.

**Scheme 8:**
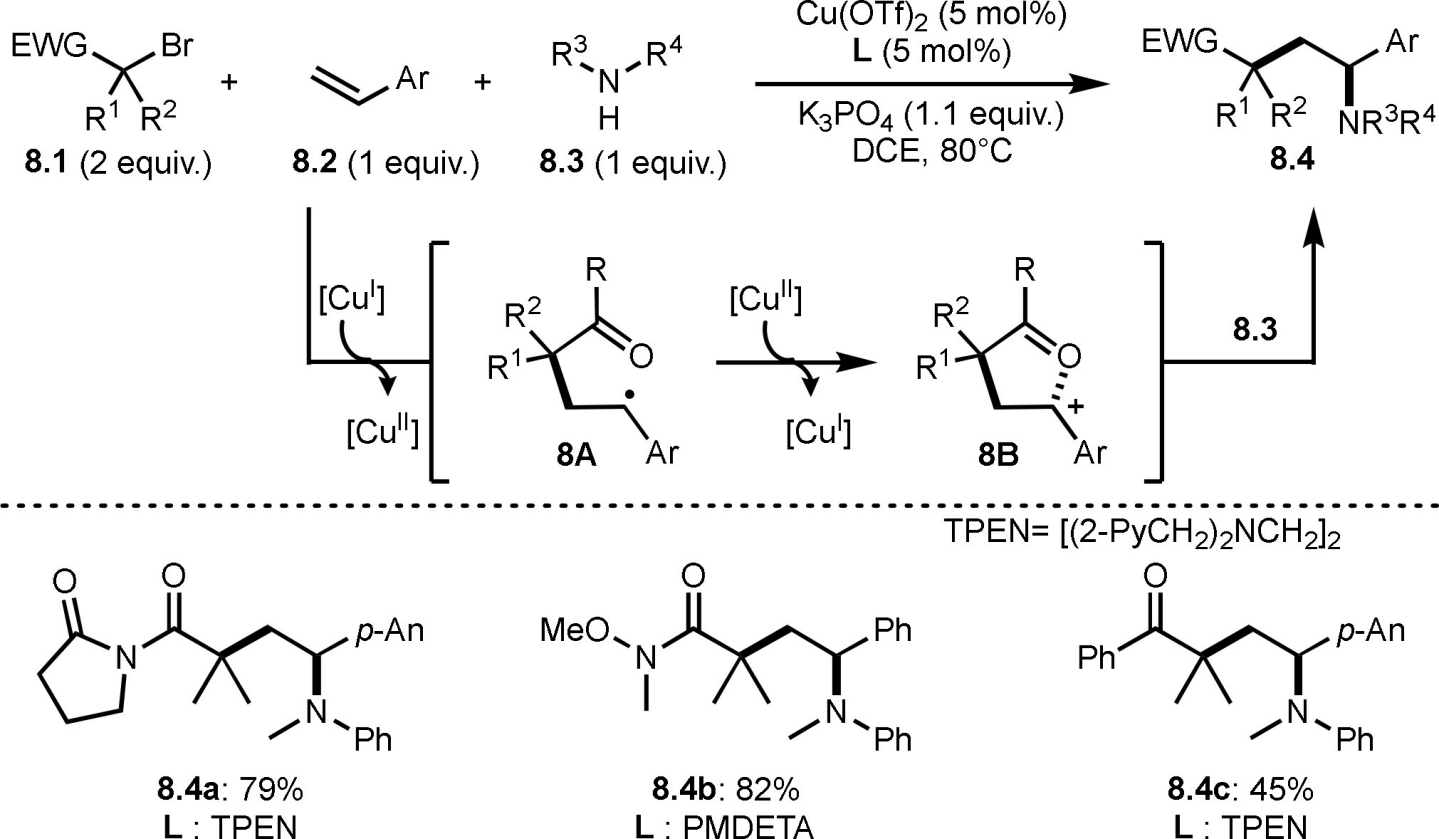
Hull's carboamination.

**Scheme 9:**
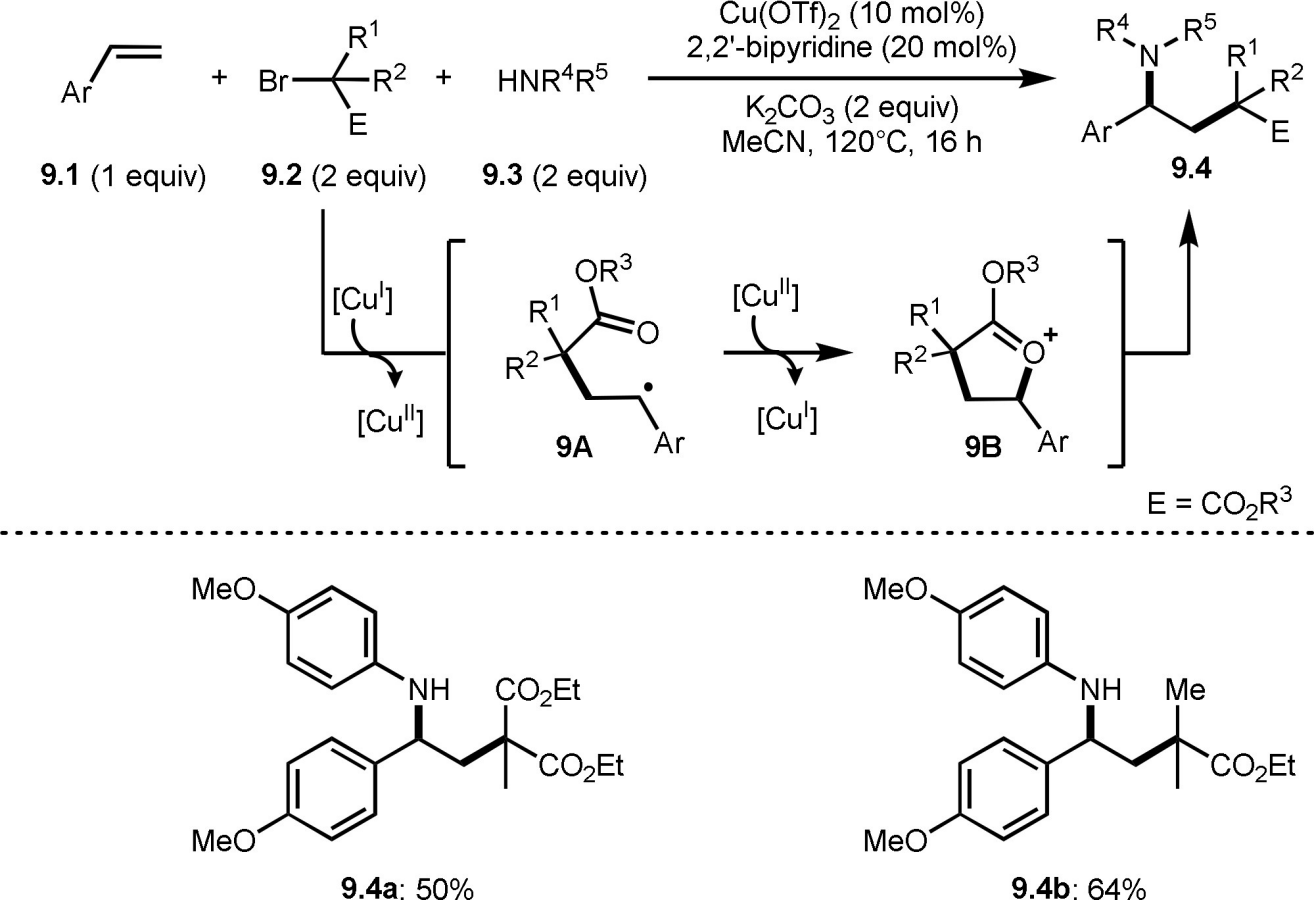
Li's carboamination.

Cu catalysts are not only very good SET reagents but also oxidizing agents that produce cationic species. Hull and Li's group independently discovered carboamination reactions of olefins using α‐bromocarbonyl compounds, in which both radical and cation reaction occurs (Scheme 8 and 9).[^135^, ^136^] Hull's group employed PMDETA or TPEN (*N,N,N′,N′*‐Tetrakis(2‐pyridylmethyl)ethylenediamine) as a ligand for Cu catalyst and accomplished efficient carboamination reactions (Scheme 8). They synthesized various tert‐alkylated products (**8.4**) from the reaction of α‐bromocarbonyl compounds (**8.1**), olefins (**8.2**), and amines (**8.3**). Alkylation process occurred through radical reaction, whereas amination process implied a cationic reaction. One of the driving forces behind this reaction is the coordination of the carbonyl group of **8.1** to the cationic intermediate **8B** produced from the copper oxidation reaction of **8A**. When α‐bromocarboxamides were used as a substrate, cyclization occurred to generate lactam, in which the nitrogen of carboxamide attacked to cation intermediate **8B** to undergo an intramolecular cyclization.

Hull's group focused on tertiary alkylation (Scheme 8), whereas Li's group focused on secondary alkylation for carboalkylation (Scheme 9). But they showed some tert‐alkylation examples. The Cu catalyzed reaction of **9.1**, **9.2** and **9.3** in MeCN at 120 °C gave tert‐alkylated products (**9.4 a** and **9.4 b**). The mechanism was similar to Hull's reports shown in Scheme 8. In this case, they also obtained lactams when α‐bromocarboxamides were used as a substrate.

**Scheme 10:**
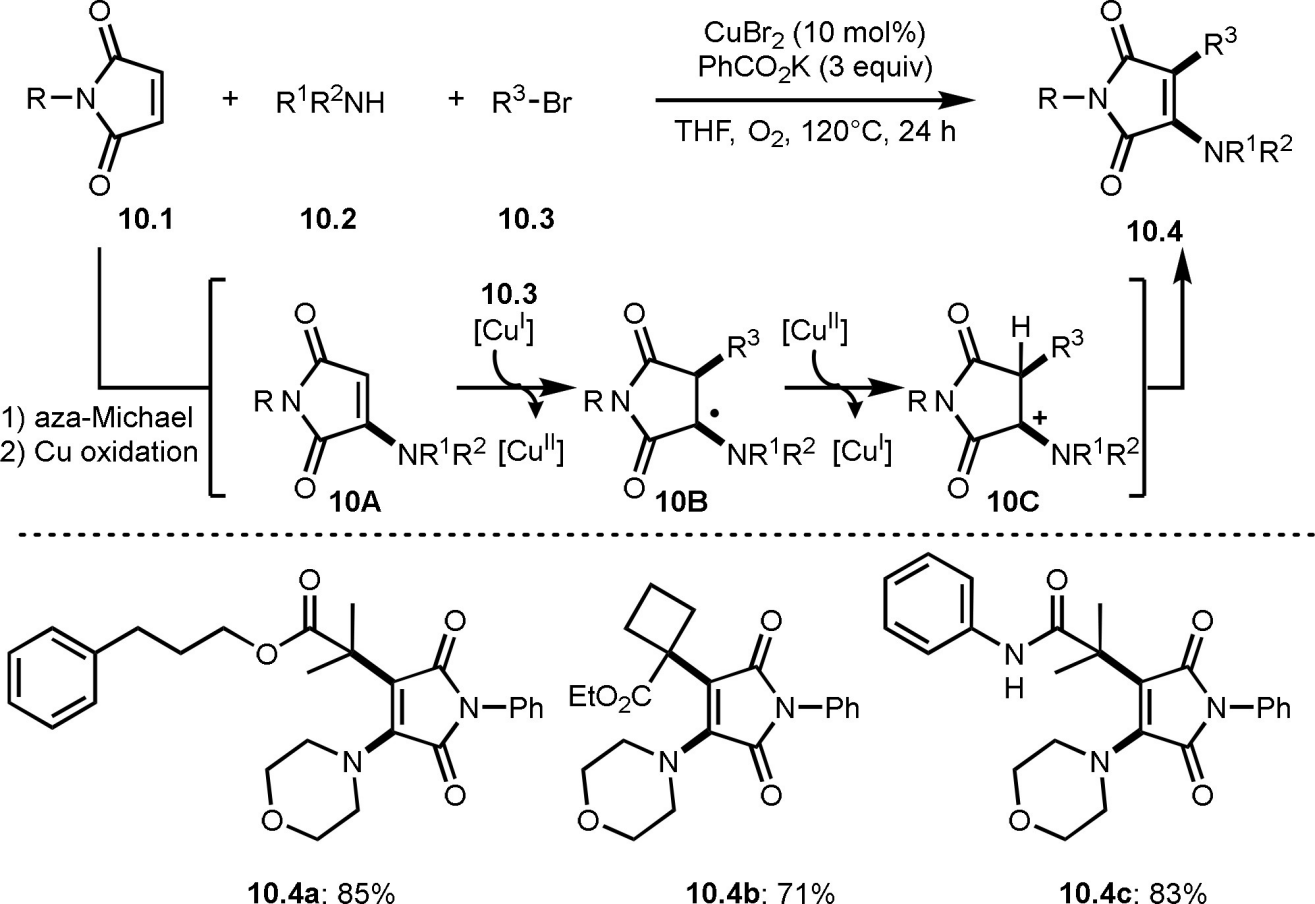
Carboamination with maleimide.

The above‐mentioned carboamination is the addition of a tert‐alkyl radical and an amine to a C−C double bond to simultaneously form a C−N bond and a C−C bond, whereby the C−C double bond is converted to a C−C single bond. Ma and Wu's group reported interesting carboamination reaction of maleiminde and α‐bromocarbonyl compound (Scheme 10).^[137]^ The Cu‐catalyzed reaction of maleimides (**10.1**), amines (**10.2**) and abromocarbonyl compounds (**10.3**) produced maleimides (**10.4**) possessing both *tert*‐alkyl group and amine while maintaining the C−C double bond. In this reaction, aza‐Michael reaction of **10.1** and **10.2** followed by Cu oxidation occurred to generate **10A**. Next, tert‐alkyl radical added to **10A** followed by Cu oxidation/proton elimination to produce **10.4**. Under these conditions, both α‐bromoesters and α‐bromocarboxamides underwent the carboamination.

**Scheme 11:**
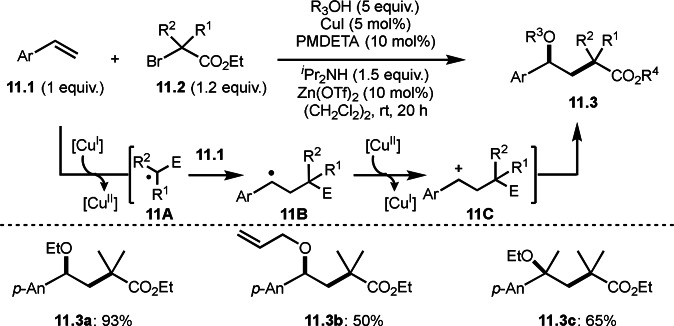
Carboalkoxylations.

Similar to carboamination, Nishikata's group reported a carboalkoxylation reaction using α‐bromoesters (Scheme 11).^[138]^ Yoon's group shown in Scheme 7 employed vinyl ethers as an olefin substrate, whereas they employed styrene derivatives (**11.1**). The reaction of **11.1**, **11.2** and alcohol in the presence of a Cu catalyst gave the corresponding carboalkoxylated product (**11.3**) in good yields. Mechanism of this reaction is also including a radical (**11A** and **11B**) and a cationic intermediate (**11C**). The addition of Lewis acid accelerated the reaction but its role was not clear. This radical/cation cross‐over reaction enables the reaction of vinyl substituted arenes, styrene derivatives, and α‐methyl substituted arenes at room temperature. Although carboaminations described above required high temperature, this carboalkoxylation occurred at room temperature.

**Scheme 12:**
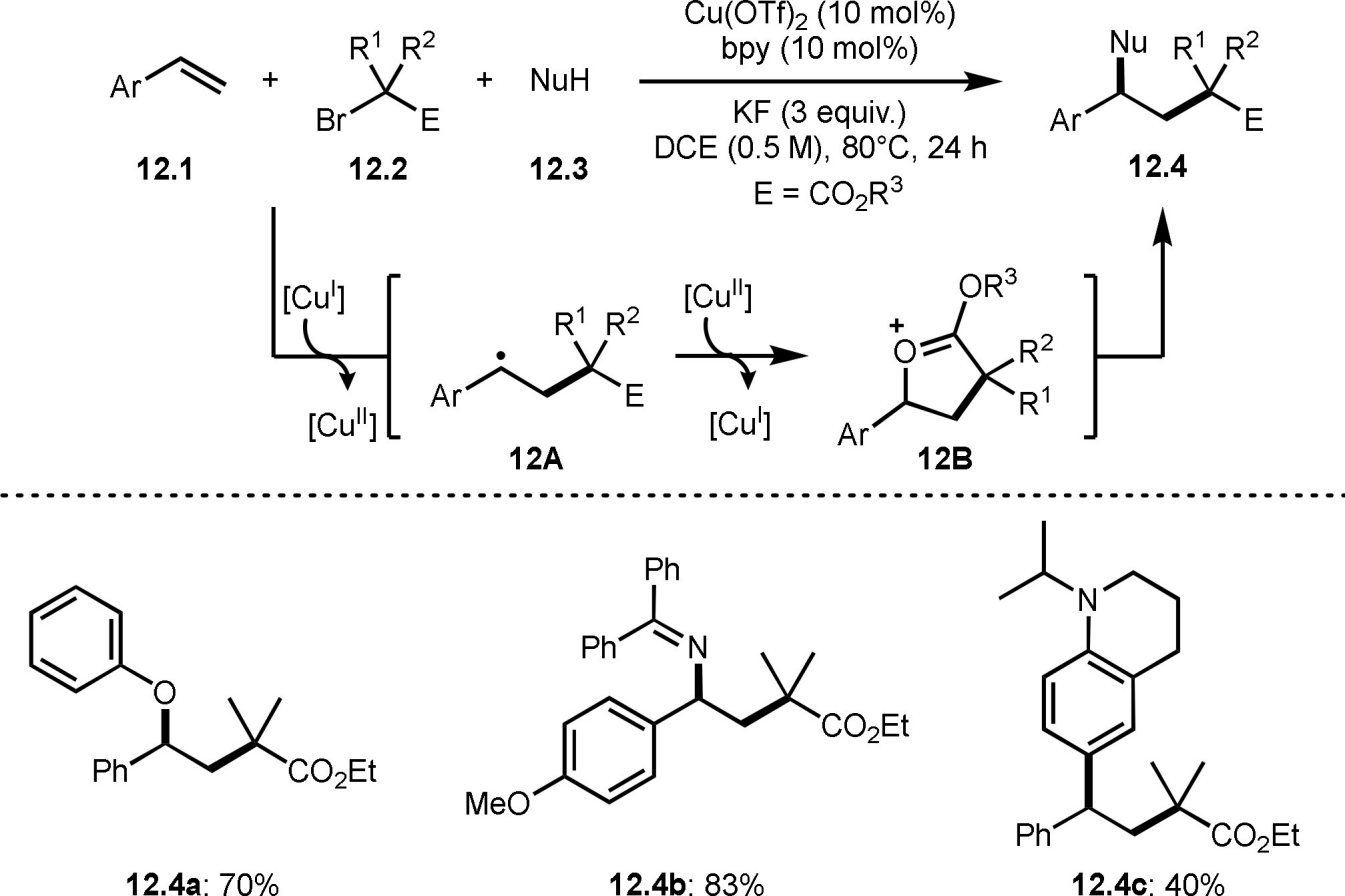
Carbofunctionalizations.

In three‐component coupling, a cationic intermediate is very important in the reaction with nucleophiles, such as amine or alcohol. Hull's group developed broad carbofunctionalization reactions of styrenes (**12.1**), α‐bromoesters (**12.2**), and nitrogen‐, oxygen‐, and carbon‐nucleophiles (**12.3**) in the presence of a Cu catalyst (Scheme 12).^[139]^ They extended their previous reaction conditions shown in Scheme 9 to carry out this three‐component reaction. Nitrogen‐, and oxygen‐nucleophiles smoothly underwent the reaction to produce **12.3**. On the other hand, carbon‐nucleophiles resulted in moderate yields of **12.4**. Carbon‐nucleophiles, mainly electron rich arenes, are probably less reactive towards intermediate **12B** due to the steric problem. This type of C−H transformations will be introduced later Scheme s.

**Scheme 13:**
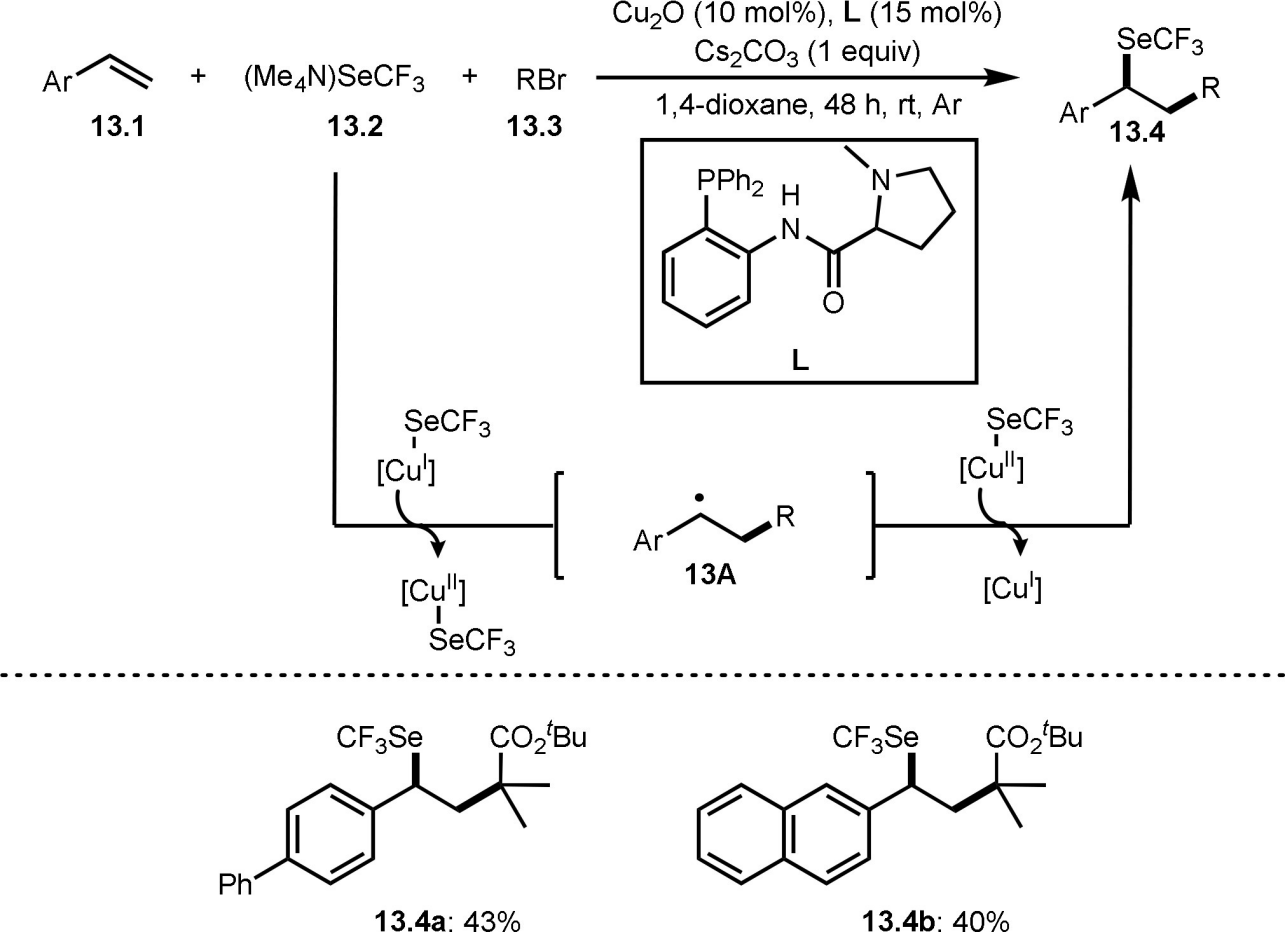
Carboselenations.

Li, Chen and Liu's group reported three‐component coupling with Selene compound (Scheme 13).^[140]^ A difficulty in three‐component reactions with α‐bromocarbonyl compounds is the interaction between the reactants. If the α‐bromocarbonyl compound reacts with only one of the reactants, a three‐component product would not be observed. In this reaction, selenide **13.2** first reacted with Cu^I^ to produce [Cu^I^]SeCF_3_. [Cu^I^]SeCF_3_ was then reacted with **13.3**, followed by radical addition to **13.1** to produce **13A** and [Cu^II^]SeCF_3_. Finally, **13A** coupled with [Cu^II^]SeCF_3_ to produce **13.4**. The yields of **13.4** were not high, but they showed carboselenation with α‐bromocarbonyl compounds as a tert‐alkyl source.

**Scheme 14:**
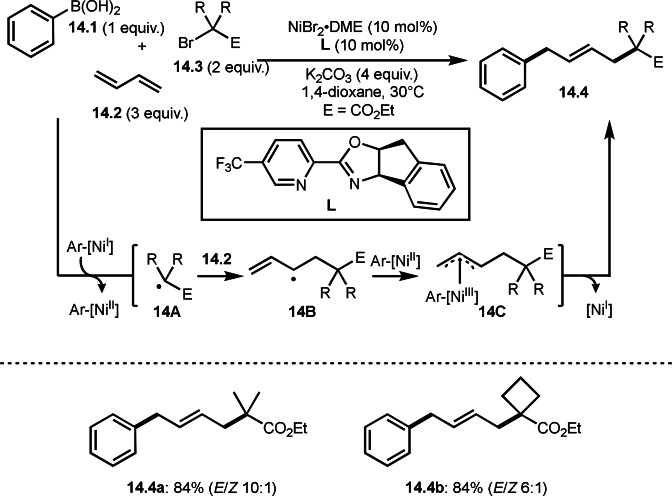
Three‐component reaction with 1,3‐butadiene.

1,3‐Butadiene is also good reaction partner in three‐component reaction. Zhang, Shi, and Wag's group reported the Ni‐catalyzed reaction of arylboronic acids (**14.1**), 1,3‐butadiene (**14.2**), and α‐bromocarbonyl compounds (**14.3**) to produce 1,4‐tert‐alkylarylation products (**14.4**) (Scheme 14).^[141]^ Their main focus in this reaction is difluoromethylation, though tert‐alkylated products (**14.4 a** and **14.4 b**) were obtained in good yields. The problem of this reaction is regioselectivity, but this reaction gave *E*‐**14.4** selectively. Although they screened various bidentate ligands (**L**), the ligands affected chemical yield rather than selectivity. The reaction was initiated by transmetalation between Ni^I^ and **14.1** to produce Ar‐[Ni^I^], which underwent SET to give Ar‐[Ni^II^] and **14A**. A key intermediate **14C** was formed by adding **14A** to **14.2** followed by the reaction of Ar‐[Ni^II^] with **14B**. Finally, the main product **14.4** was generated via reductive elimination of **14C**.

**Scheme 15:**
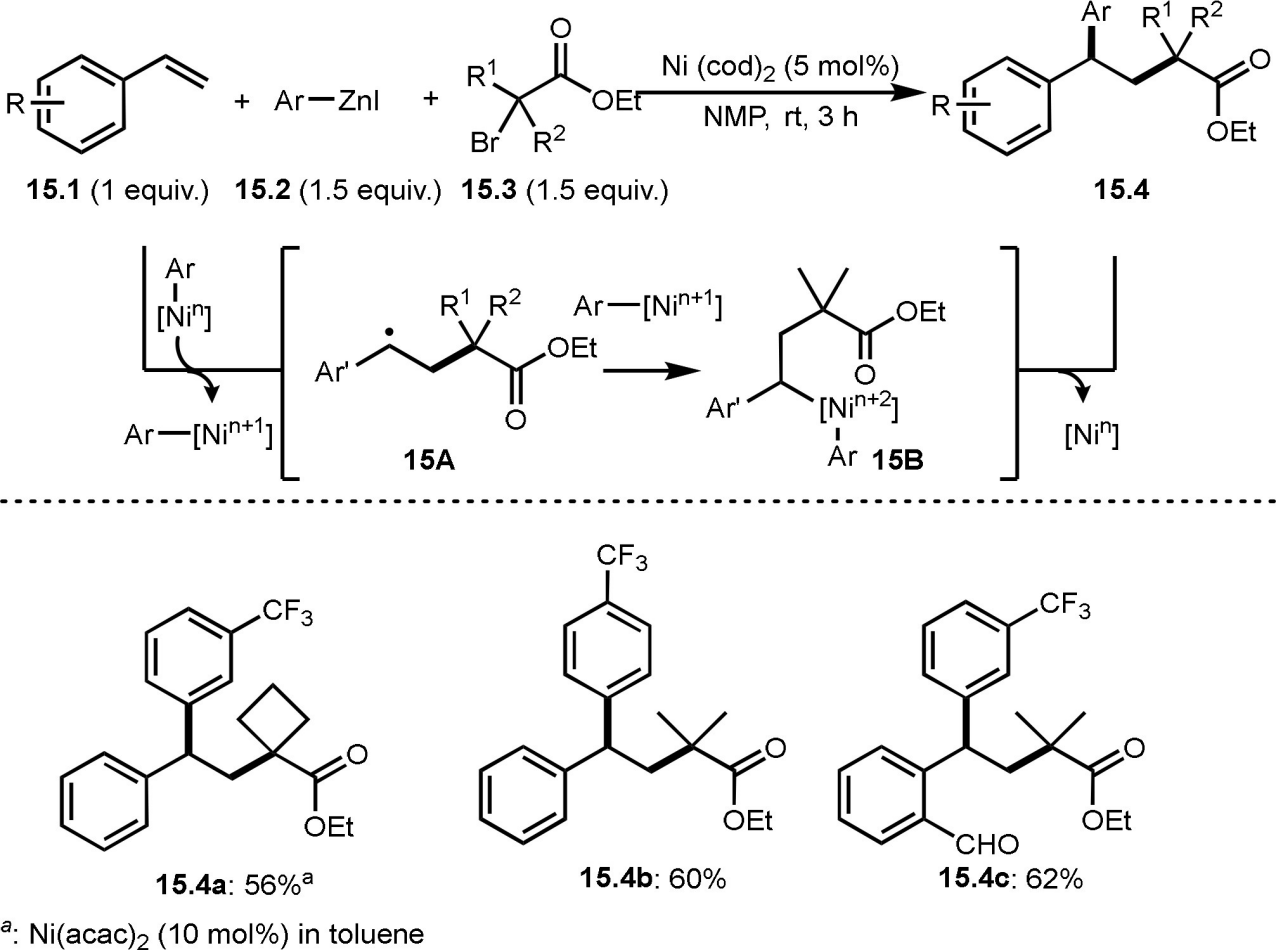
Ni‐catalyzed three‐component reaction with arylzinc reagents.

**Scheme 16:**
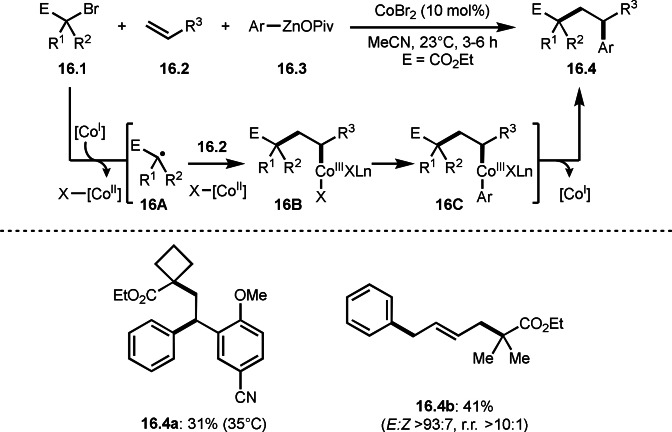
Co‐catalyzed three‐component reaction with arylzinc reagents.

Giri's group and Lei and Li's group independently reported three‐component reaction with arylzinc reagents in the presence of a Ni or Co catalyst (Scheme 15 and 16).[^142^, ^143^] Giri's group employed styrenes (**15.1**) and arylzinc reagents (**15.2**) and α‐bromocarbonyl compounds (**15.3**), in which tert‐alkylarylated products (**15.4**) were obtained (Scheme 15). Lei's group previously reported Ni catalyzed Mizoroki‐Heck like olefination with α‐bromocarbonyl compounds (Scheme 1). In the reaction, diphosphine ligand (dppp) was effective to generate α‐radicals from α‐bromocarbonyl compounds. But Giri's reaction did not require a ligand. The reaction initiated by transmetalation between Ni^n^ and **15.2** to produce Ar‐[Ni^n^], which underwent SET to give Ar‐[Ni^n+1^] and α‐radicals. As in Scheme 14, **15.4** was obtained from the addition of α‐radicals to **15.1** followed by reductive elimination of **15B**.

On the other hand, Lei and Li's group employed Co‐catalyzed reaction of **16.1**, **16.2** and **16.3** to give **16.4** (Scheme 16).^[143]^ This reaction system also did not require a ligand for a Co salt to generate α‐radicals from α‐bromocarbonyl compounds. The reaction mechanism is slightly different from Scheme 15. At first, SET from Co^I^ to **16.1** occurred to generate X‐[Co^II^] and **16A**. After the addition of **16A** and X‐[Co^II^] to **16.2**, **16B** was produced. **16B** then underwent transmetalation with **16.3** to give **16C**. The product **16.4** was obtained via reductive elimination of **16C**. This proposed mechanism is based on their control experiments. For example, the reaction of CoBr_2_ and **16.3** produced Co^I^ not Ar−Co^n^ species. Generation of carbon centered radical was observed by EPR analysis.

**Scheme 17:**
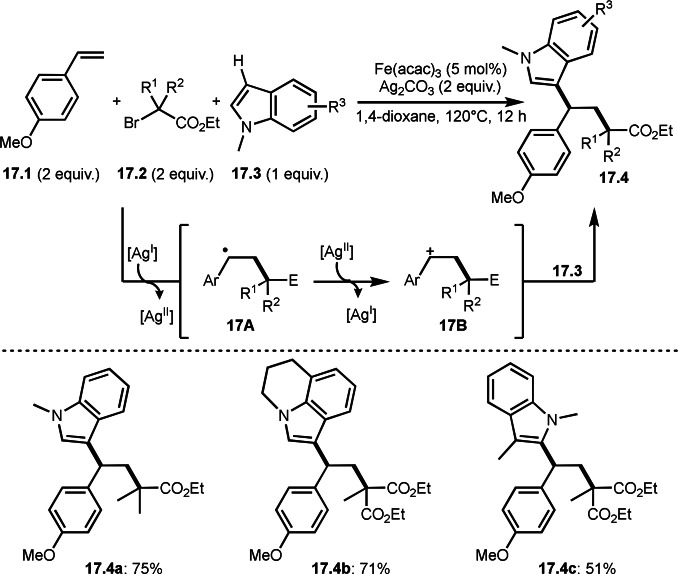
Ag‐mediated three‐component reaction with heteroaromatic C−H bonds.

Three‐component reaction is including a cationic reaction. The carbocation produced can react with an electron‐rich arene, as in the Friedel‐Crafts reaction. By using this property, Li's group successfully carried out three‐component reaction of styrenes (**17.1**), α‐bromocarbonyl compounds (**17.2**), and indoles (**17.3**) to produce **17.4** (Scheme 17).^[144]^ In this reaction, α‐radicals can be generated by the reaction of **17.2** with Ag^I^ salt. The resulting Ag^II^ oxidized the radical intermediate **17A** generated from the reaction of α‐radicals with **19.1** to give the cationic intermediate **17B**. At this step, Ag^II^ reverted to Ag^I^, but the silver salt was not used as a catalyst. **17B** then reacted with **17.3** to afford **17.4**. They described that a role of Fe(acac)_3_ might play a Lewis acid to stabilize the radical intermediates and improved the yields.

**Scheme 18:**
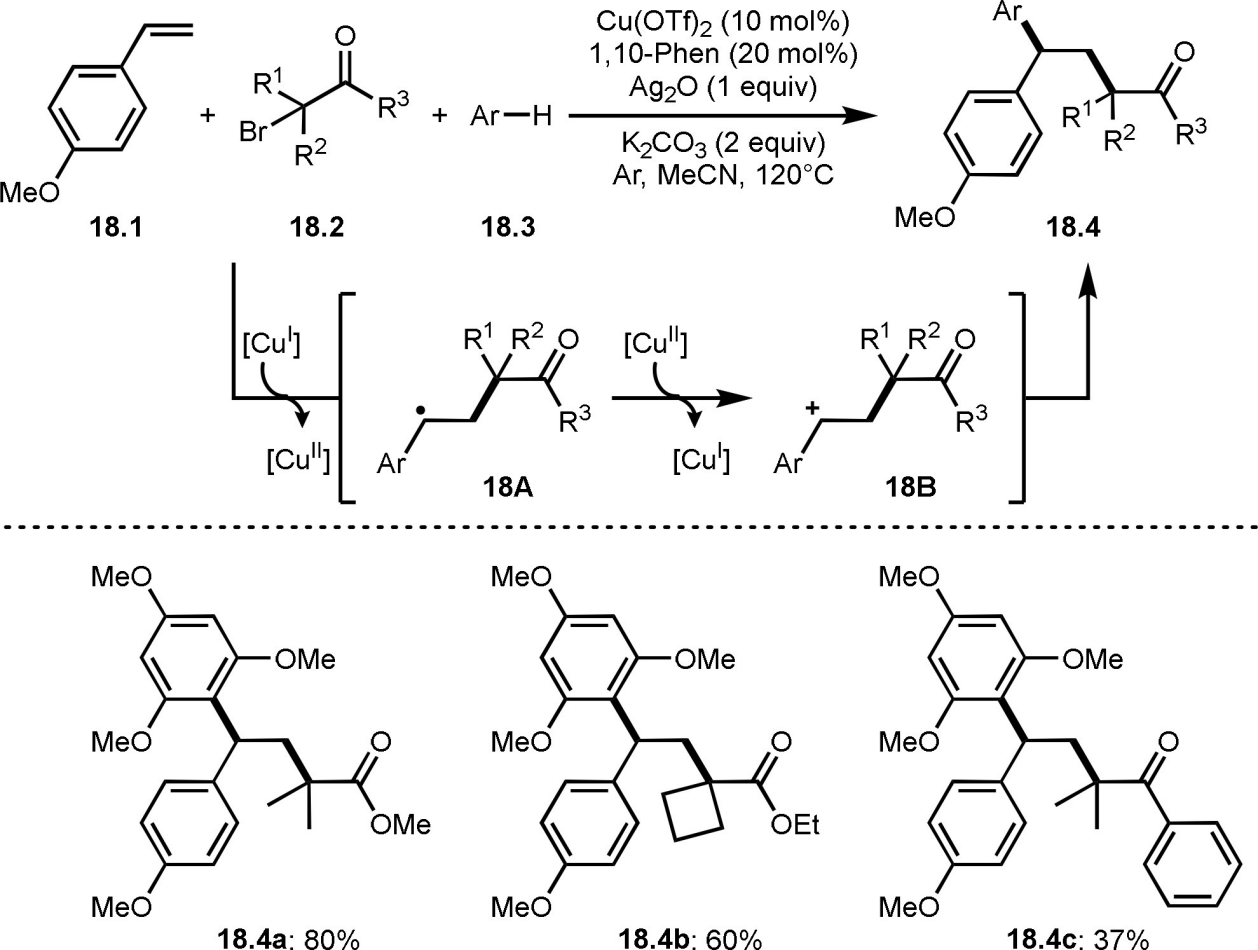
Cu‐catalyzed three‐component reaction with aromatic C−H bonds.

**Scheme 19:**
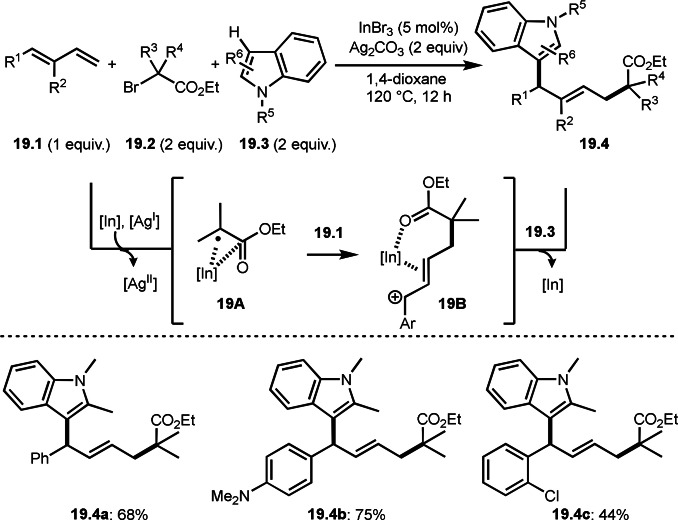
In‐catalyzed three‐component reaction with heteroaromatic C−H bonds.

Li's group also developed three‐component reaction with electron‐rich arenes (Scheme 18).^[145]^ The reaction of styrenes (**18.1**), α‐bromocarbonyl compounds (**18.2**), and electron‐rich arenes (**18.3**) in the presence of Cu catalyst and Ag oxidant furnished **18.4**. In previous report the author's employed a In salt as a catalyst to generate tert‐alkyl radical, but Cu was used as a catalyst in this case. The product **18.4** was generated through similar reaction mechanism shown in Scheme 19. The reactive arenes were mainly 1,3,5‐trimethoxybenzene, and simple or electron‐poor arenes were not able to be employed, indicating that cationic species (**18B**) were involved in this reaction.

Regio‐ and stereoselectivity is difficult problem in three‐component reaction with a diene. Because there are some radical addition sites, and *E*/*Z*‐selectivity of the resulting product is difficult to accomplish. Li's group successfully developed selective three‐component reaction with dienes (Scheme 19).^[146]^ The reaction of dienes (**19.1**), α‐bromocarbonyl compounds (**19.2**), and indoles (**19.3**) in the presence of indium catalyst and Ag salt to produce (*E*)‐1,4‐tertalkylarlation products (**19.4**) in moderate to good yields with perfect selectivities. In their hypothetical mechanism a tert‐alkyl radical/Indium intermediate **19A** is involved. **19A** next added to **21.1** followed by Ag oxidation to produce a cation intermediate (**19B**). **19.4** was obtained from the aromatic C−H substitution reaction of **19.3** and **19B**. Steroselectiviy is not clear but high regioselectivities might be due to the formation of benzylic cation intermediate (**19B**).

**Scheme 20:**
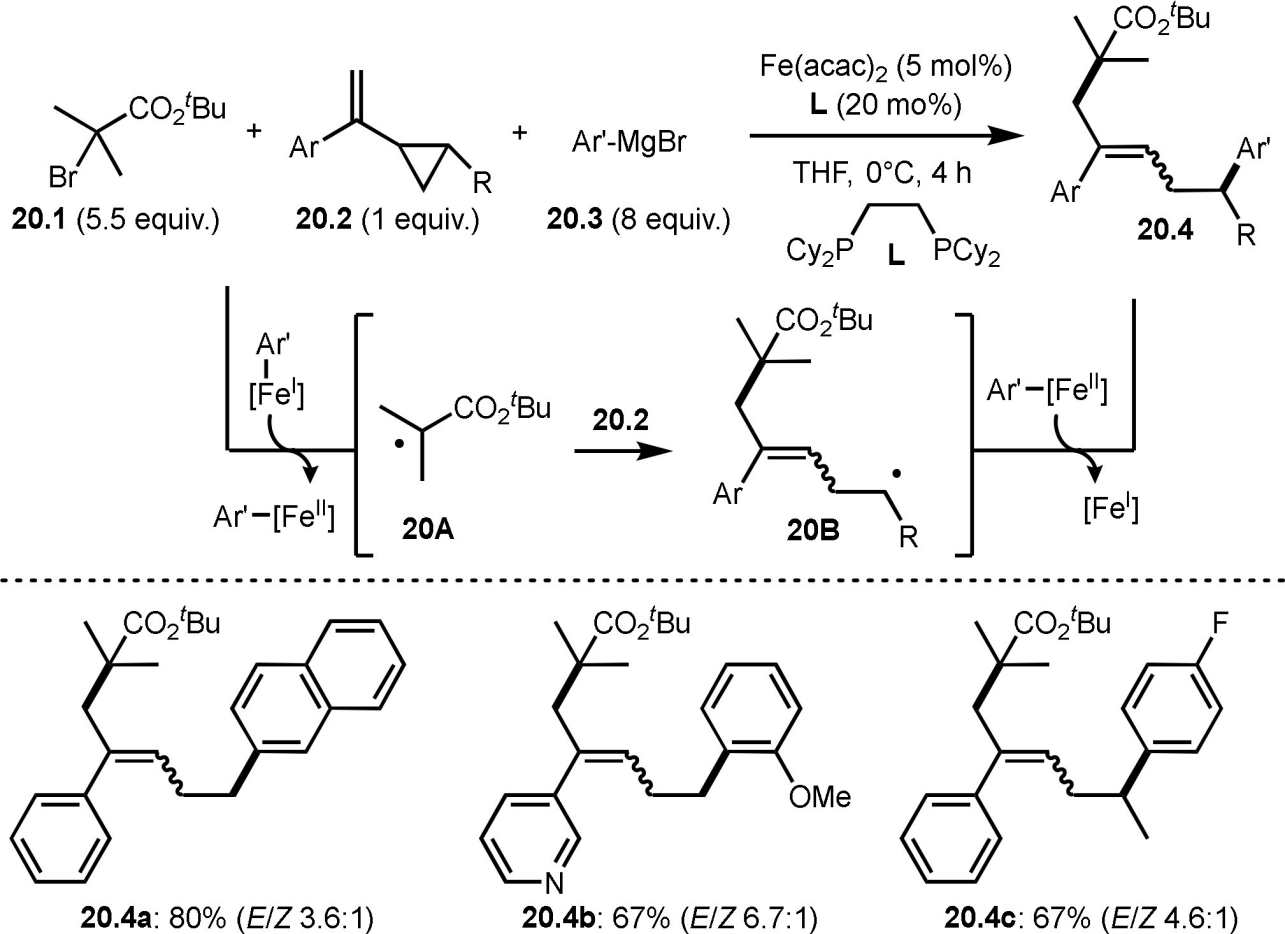
Three‐component reaction with vinyl cyclopropanes.

Vinyl cyclopropanes has interesting reactivity in three‐component reaction with tert‐alkyl radicals. Gutierrez's group discovered that the reaction of α‐bromocarbonyl compound (**20.1**), vinyl cyclo propane (**20.2**), and Grignard reagents (**20.3**) produced tert‐alkylarylated product (**20.4**) (Scheme 20).^[147]^ The reaction first underwent Grignard reaction with an iron salt to produce Ar’‐[Fe^I^]. Next, α‐radical (**20A**) was generated via SET from the reaction of Ar’‐[Fe^I^] with **20.1**. A radical ring opening reaction of a cyclopropane ring occurred in the reaction of **20.2** with **20A** to afford **20B**. Finally, **20.4** was obtained by coupling of **20B** with Ar’‐[Fe^II^]. The products (**20.4 a**–**20.4 c**) have a C−C double bond but their stereoselectivities were moderate.

**Scheme 21:**
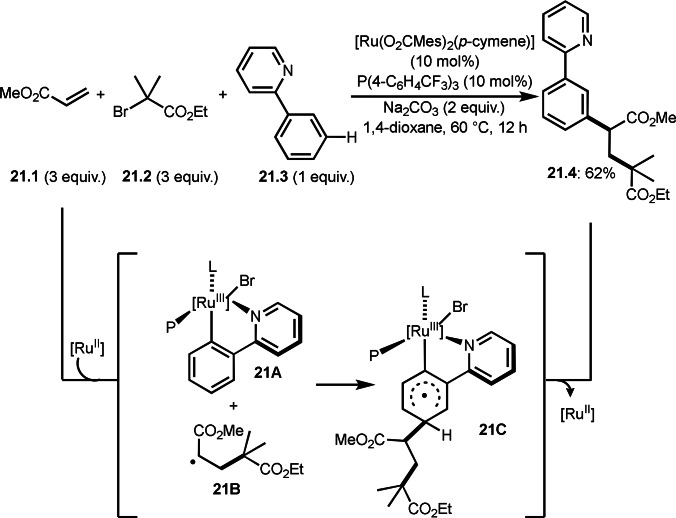
Ru‐catalyzed three‐component reaction with *meta*‐aromatic C−H bonds.

*E*/*Z*‐selectivities and regio‐selectivities are important factors in three‐component reaction. Additionally, controlling *orhto*‐, *meta*‐ and *para*‐selectivities in the multi‐component reactions involving C−H arylations are difficult. Liang's group precisely controlled arene *meta*‐C−H bond reactivities in three‐component reaction (Scheme 21).^[148]^ The reaction of acryl ester (**21.1**), α‐bromocarbonyl compound (**21.2**), and arene (**21.3**) in the presence of a Ru catalyst selectively gave *meta*‐C−H alkylated product **21.4** in 62 % yield. Their main focus in this paper was difluoromethylation, therefore, example of tert‐alkylation was only one. *tert*‐Alkyl radical is generated via SET from Ru^II^ to **21.2**. And the radical added to **21.1** to produce **21B**. Although *meta*‐selective C−H tert‐alkylation will be discussed later section, a key intermediate was C−H ruthenated intermediate (**21C**) generated from the reaction of **21A** and **21B**, in which a pyridyl group acted as a directing group. Finally, rearomatization of **21C** followed by demetallation gave **21.4**.

**Scheme 22:**
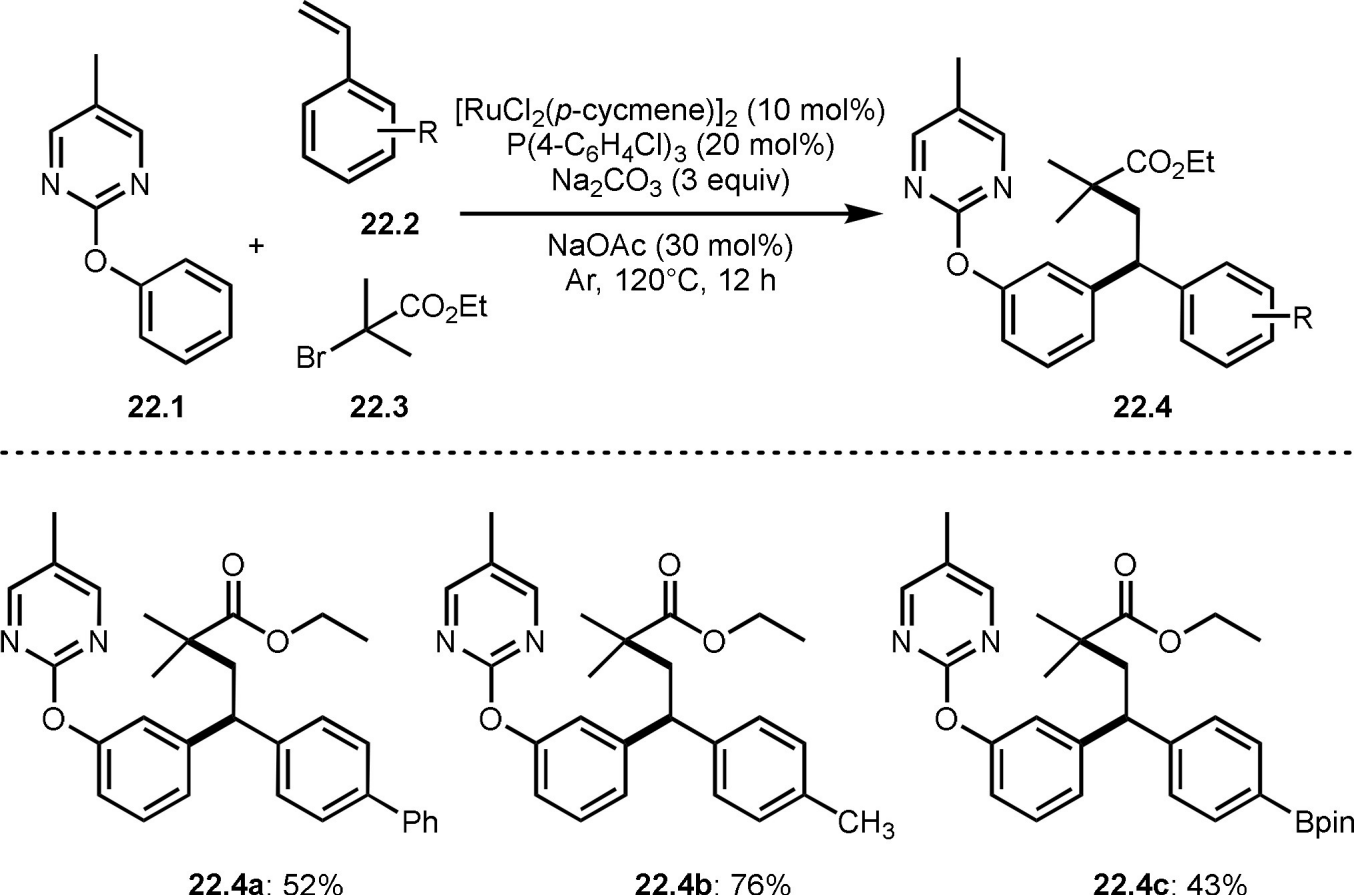
Ru‐catalyzed three‐component reaction with *meta*‐aromatic C−H bonds.

A directing group is very important to carry out aromatic C−H functionalizations. Liang's group next employed 5‐methylpyrimidine as a directing group to perform three‐component reaction (Scheme 22).[^149^, ^150^] The reaction of arenes (**22.1**), styrenes (**22.2**), and α‐bromoesters (**22.3**) in the presence of a Ru catalyst selectively gave *meta*‐C−H alkylated phenol derivatives (**22.4 a**–**c**) in moderate to good yields.

**Scheme 23:**
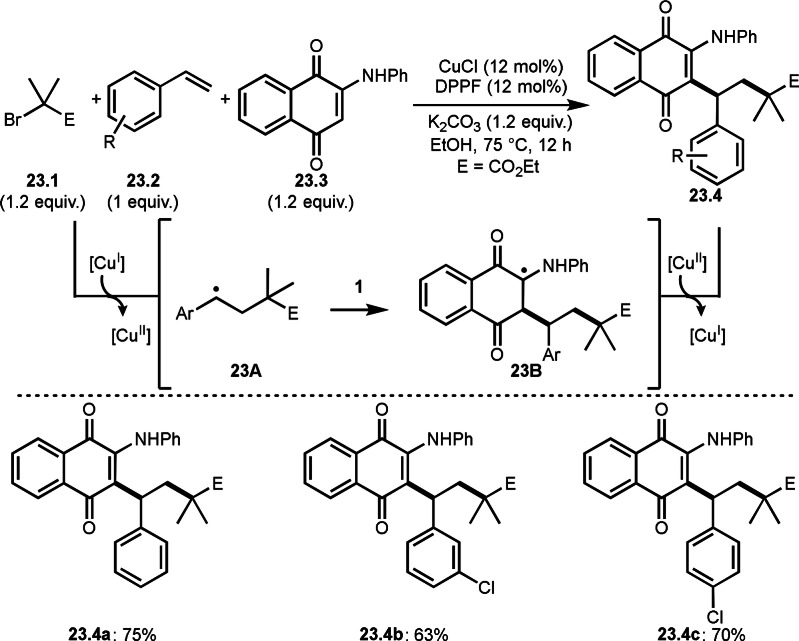
Cu‐catalyzed three‐component reaction with vinylic C−H bonds.

A C−C double bond of quinone derivatives are also a good substrate in three‐component reaction. Zhang's group reported the reaction of α‐bromocarbonyl compounds (**23.1**), styrenes (**23.2**), and 2‐amino substituted quinones (**23.3**) in the presence of a Cu catalyst to produce *tert*‐alkylated aminoquinones (**23.4**) (Scheme 23).^[151]^ The amino functionalized C−C double bond of **23.3** is reactive for radical species. After the generation of tert‐alkyl radical via SET of Cu^I^ followed by the addition to **23.2**, **23A** was produced. **23A** smoothly added to **23.3** to give **23B**. **23.3** is reactive for a radical species but it is not reactive towards tert‐alkyl radicals probably due to the steric hinderance. Desired **23.4** was produced by Cu^II^ oxidation followed by deprotonation.

**Scheme 24:**
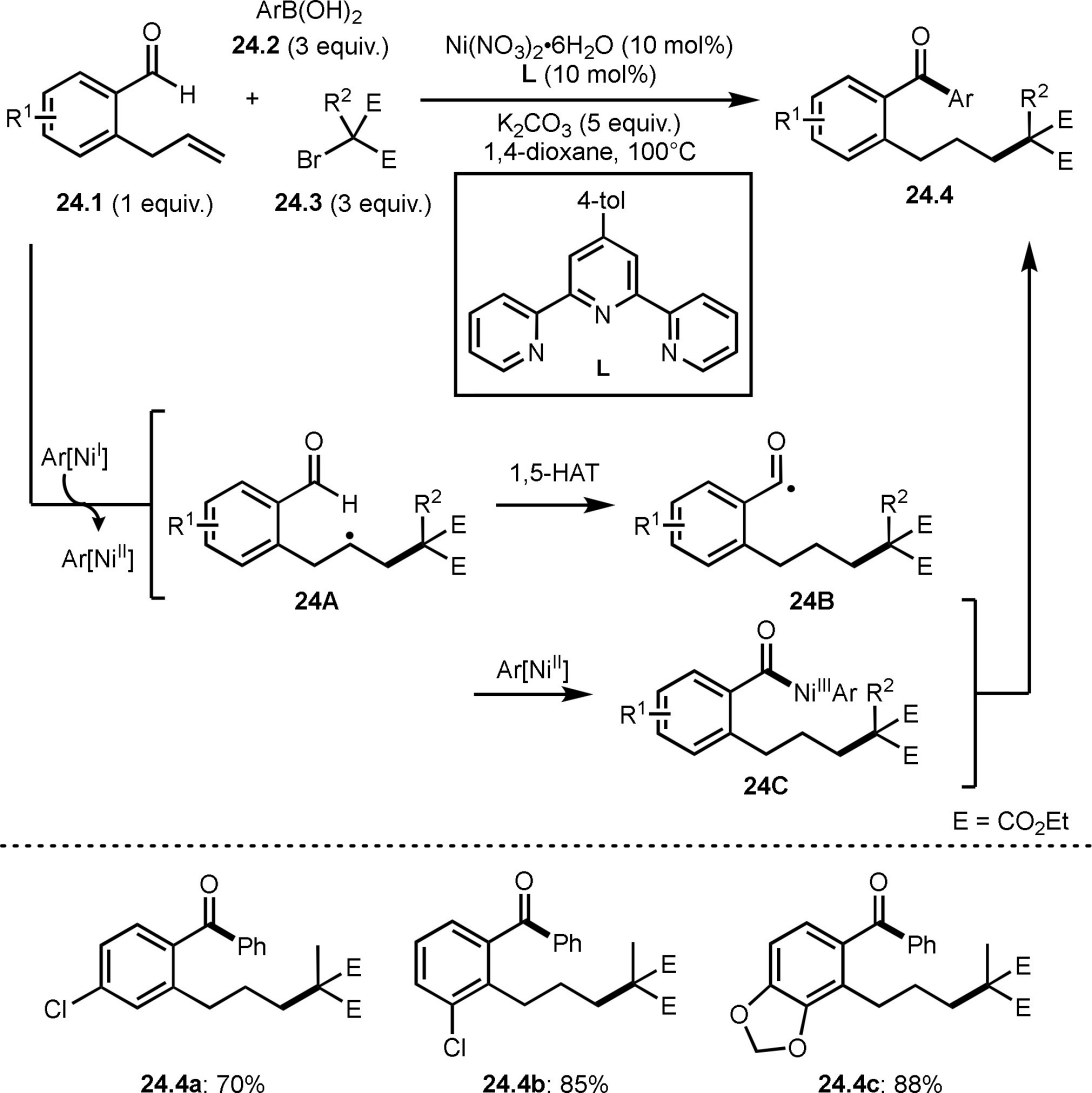
Three‐component reaction via 1,5‐HAT.

In three‐component reaction, a remote functionalization via 1,5‐hydrogen atom transfer (1,5‐HAT) was observed. Zhu's group reported the reaction of *ortho*‐allylbenzaldehydes (**24.1**), arylboronic acids (**24.2**), and α‐bromocarbonyl (malonate structure) compounds (**24.3**) in the presence of a Ni catalyst gave remote‐arylated products (**24.4**) (Scheme 24).^[152]^ *tert*‐Alkyl radical was generated from the reaction of **24.3** and Ar[Ni^I^] which generated from transmetalation between Ni^I^ and **24.2**. *tert*‐Alkyl radical then reacted with **24.1** to give radical intermediate **24A**. **24A** underwent 1,5‐HAT to produce **24B**. In former introduced reactions, C−C difunctionalization occur to give three‐component product, but radical transfer occurred in this case because the resulting radical species is close to a hydrogen atom of aldehyde. After HAT, Ar[Ni^II^] captured **24B** to produce **24C**, which then underwent reductive coupling to provide **24.4**. Under these conditions, good yields of **24.4 a**–**c** were obtained.

**Scheme 25:**
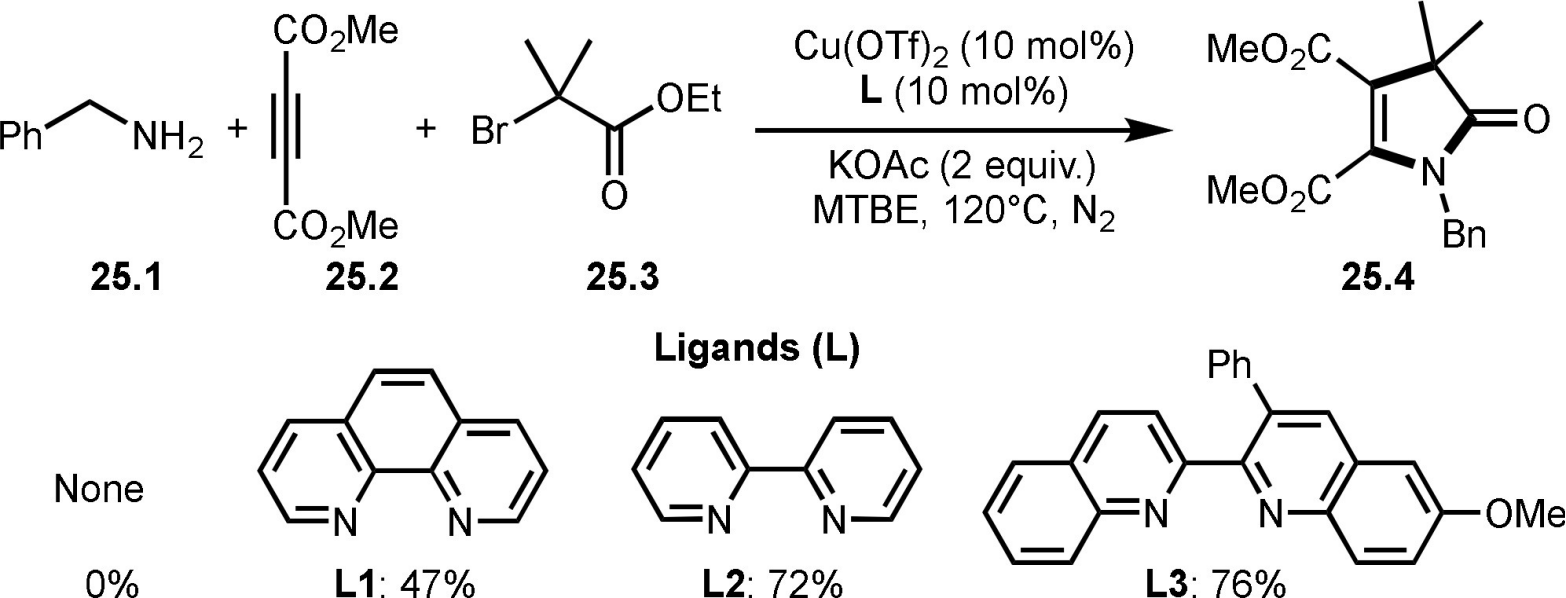
Cu‐catalyzed three‐component reaction for ligand screening.

We introduced alkenes as a platform for three‐component reactions. Next platform is alkynes. Zhu's group performed the three‐component reaction of benzyl amine (**25.1**), alkyne (**25.2**), and α‐bromoeseter (**25.3**) to produce **25.4** (Scheme 25).^[153]^ In this reaction, a ligand is very important and **L3** was found to be the best ligand. This example is not toward tert‐alkylations. But thiey showed that a radical tert‐alkylation is suitable for this kind of ligand screening.

**Scheme 26:**
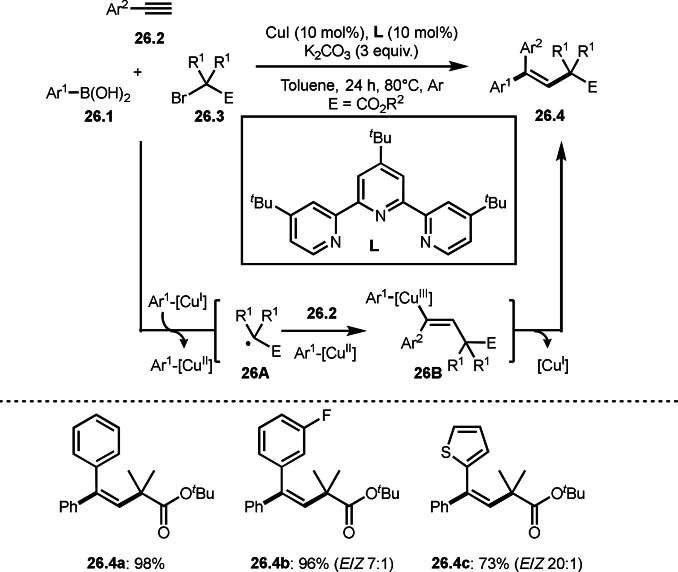
Cu‐catalyzed three‐component reaction with alkynes.

Gu, Chen, and Liu's reported interesting *anti*‐aryl‐*tert*‐alkylations in three‐component reactions of alkynes (Scheme 26).^[154]^ The reaction of arylboronic acids (**26.1**), alkynes (**26.2**), and α‐bromoeseter (**26.3**) in the presence of a Cu catalyst gave **26.4** in an *anti*‐manner. *tert*‐Alkyl radical **26A** generated via SET from Ar^1^‐[Cu^I^] to **26.3** reacted with **26.2** to produce a vinylic radical intermediate, which is captured by Ar^1^‐[Cu^II^] to provide **26B**. The author described that *the high anti stereoselectivity of the reaction is likely depending on the relative stability of the intermediate* **26B** *, where the sterically demanding alkyl group is anti to the large* Ar^1^‐[Cu^III^] *group*. Indeed, high anti‐selectivity was obtained when Ar^2^ was small. For example, a substituted phenyl group gave **26.4 b** in *E*/*Z* 7 : 1, whereas smaller thienyl group resulted in 20 : 1 of **26.4 c**.

**Scheme 27:**
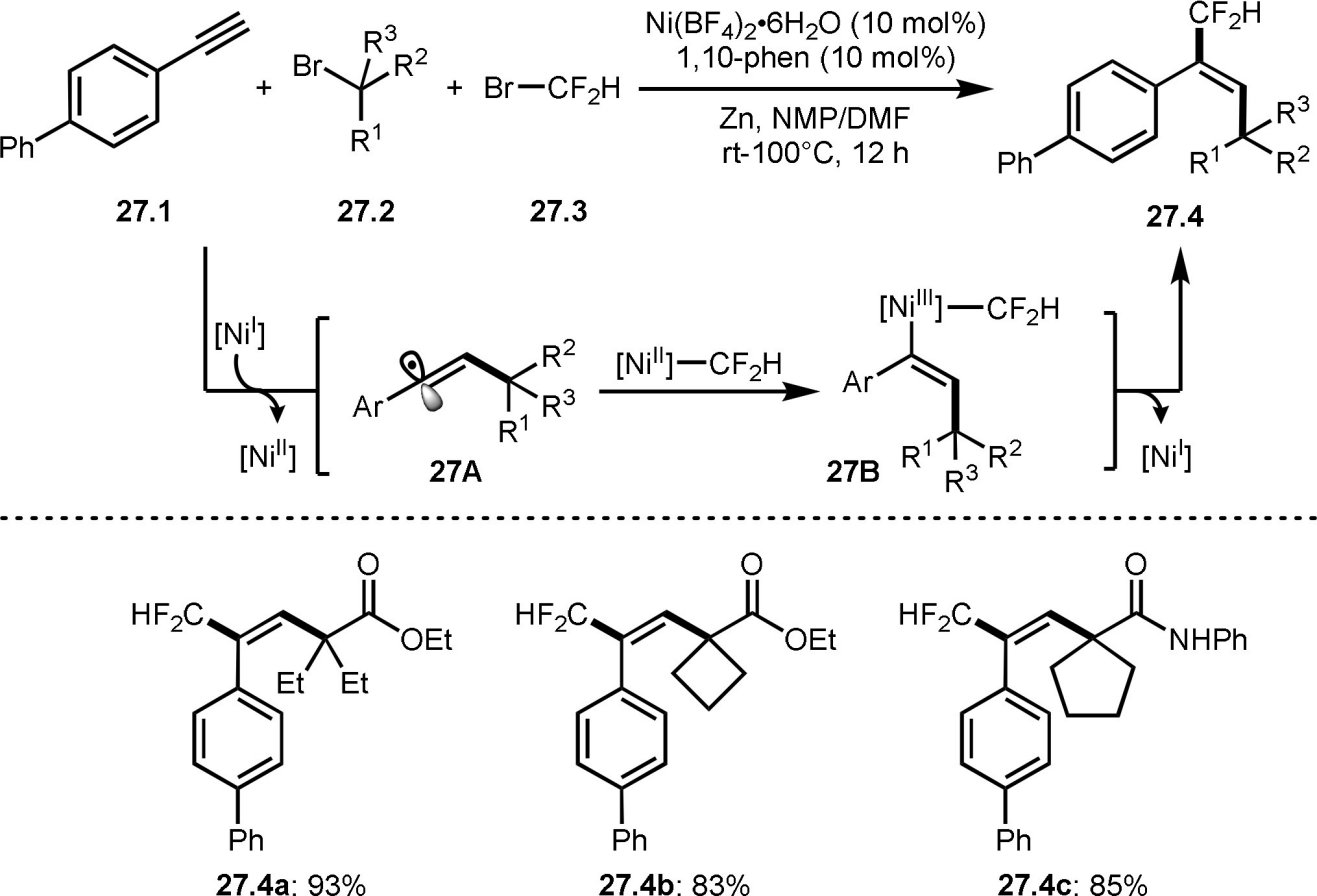
Ni‐catalyzed three‐component reaction with alkynes.

Chu's group reported three‐component reactions with difluoromethyl bromide (Scheme 27).^[155]^ The reaction of alkynes (**27.1**), α‐bromocarbonyl compounds (**27.2**), and α‐bromoeseter (**27.3**) in the presence of a Ni catalyst gave **27.4** in an *anti*‐manner. In this reaction, tert‐alkyl radical was generated from the reaction of Ni^I^ and **27.2** and reacted with **27.1** to produce vinylic radical intermediate **27A** and Ni^II^. On the other hand, Zn^0^ reacted with Br−CF_2_H to produce CF_2_H‐ZnBr, which underwent transmetalation with Ni^II^ to produce CF_2_H[Ni^II^]. **27A** then coupled with CF_2_H[Ni^II^] to give 28B. The author described that *the observed trans‐stereoselectivity could be rationalized via the rapid inversion of E/Z alkenyl radical **27A***.^[156]^ Compared with Liu's anti‐selective addition (Scheme 25), the origins of stereoselectivity is different from each other.

A radical cyclization reaction is convenient to synthesize cyclic compound including 5‐ and 6‐membered rings. For this purpose, α‐bromocarbonyl compounds are quite effective to construct highly congested and functionalized cyclic products. Inter‐ or intramolecular cyclizations are convenient synthetic options. For example, intermolecular cyclization can introduce variety of functional groups in combination with two different substrates including α‐bromocarbonyl compounds. Intramolecular cyclizations, ATRC (atom‐transfer radical cyclization), are convenient to construct simple cyclic products. In sections 3.3 and 3.4, the two types of cyclization reactions will be introduced.

### 3.3 Intramolecular Cyclization

**Scheme 28:**
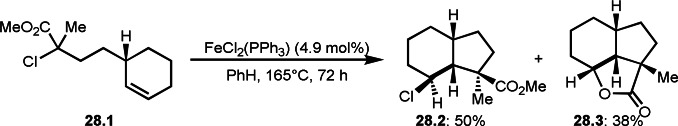
Fe‐catalyzed ATRC.

In 1988, Weinreb's group discovered Kharasch radical cyclization reaction (ATRC) using α‐bromocarbonyl compound as a *tert*‐alkyl source (Scheme 28).^[157]^ Reaction of **28.1** in the presence of an iron catalyst led to intramolecular cyclisation, producing **28.2** and **28.3** with a quaternary carbon centre. Due to the high reaction temperature, it was not possible to control whether ATRC product (**28.2**) or polycyclic product (**28.3**) was produced.

**Scheme 29:**
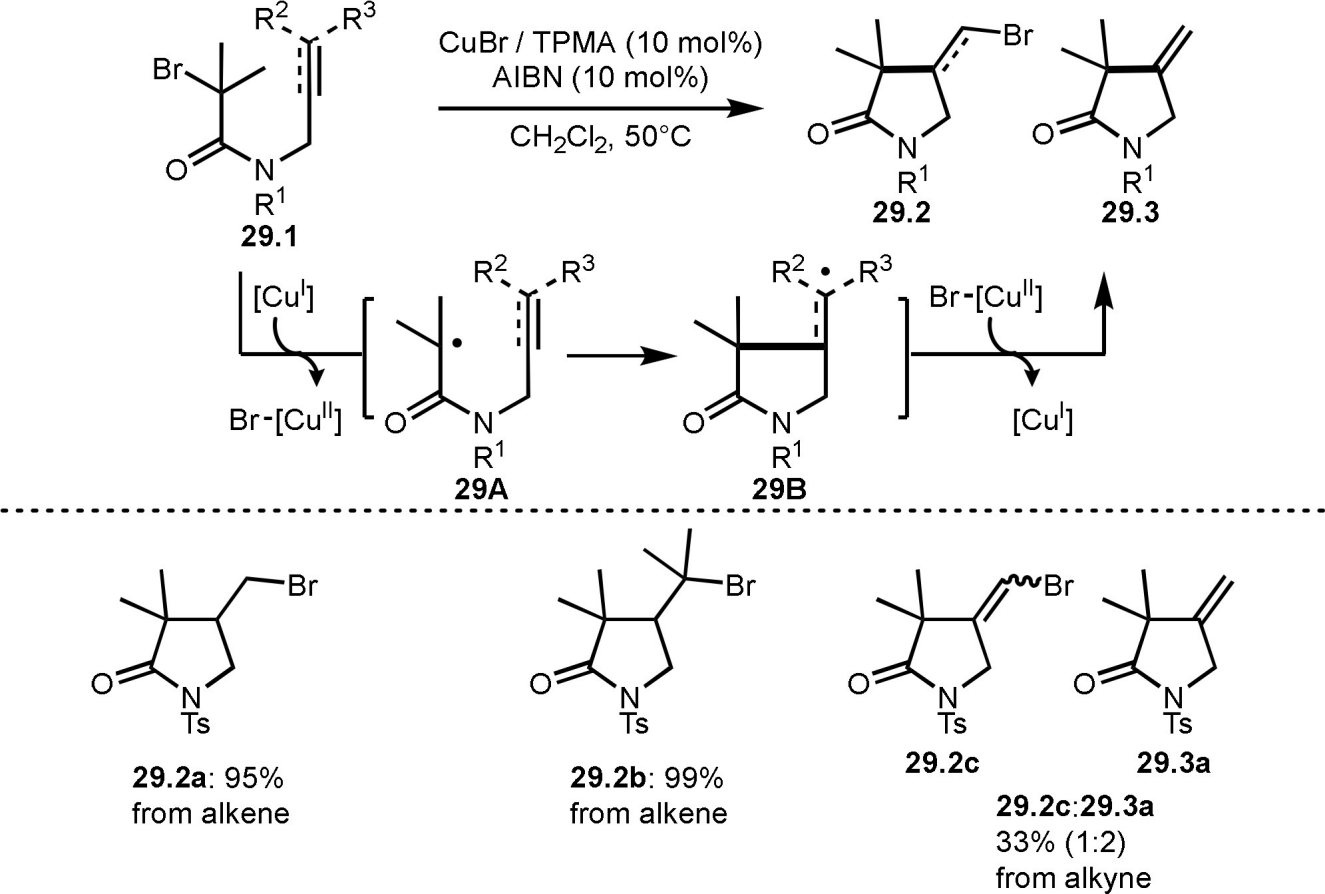
Cu‐catalyzed ATRC.

Clark's group reported ATRC of **29.1** in the presence of a Cu catalyst (Scheme 29).^[158]^ They employed alkene or alkyne substituted α‐bromocarbonyl compounds (**29.1**) as a substrate. **29.1** having an alkene moiety produced saturated cyclic compound (**29.2**) in excellent yields of **29.2 a** and **29.2 b**, whereas, **29.1** having an alkyne moiety produced both unsaturated cyclic compound **29.2** and **29.3**. ATRC with alkyne was not efficient process in this reaction. The intermediate for both cyclization was**291A**, which was generated from the reaction of a Cu salt and **29.1**. **29A** underwent cyclization to produce 31B. In this step, alkyl radical can be smoothly captured by Br[Cu^II^], but vinylic radical from the reaction with the alkyne was not. This might cause low reactivity of **29.1** having an alkyne moiety.

**Scheme 30:**
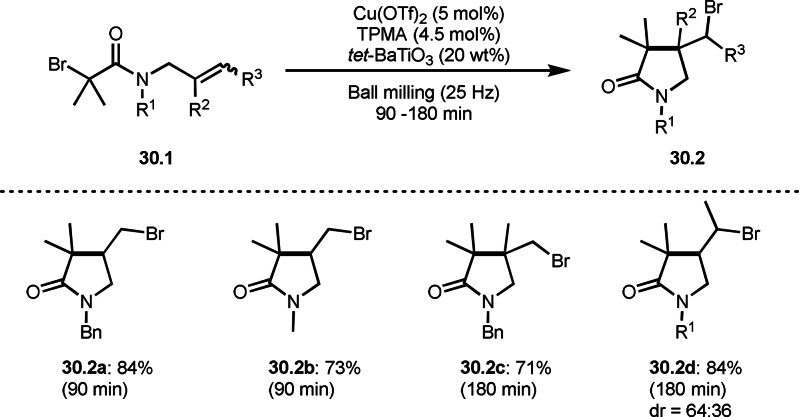
Mechanochemical ATRC.

Hernández and Bolm’ group reported ball milling technology for ATRC (Scheme 30).^[159]^ In their system, electrical polarization generated piezoelectricity to reduce a Cu^II^ precatalyst. The resulting active Cu^I^ catalyst generated tert‐alkyl radical via SET. As the result, **30.1** was converted to ATRC product **30.2** in good yields. This mechanochemical system enables solvent‐free ATRC reactions.

**Scheme 31:**
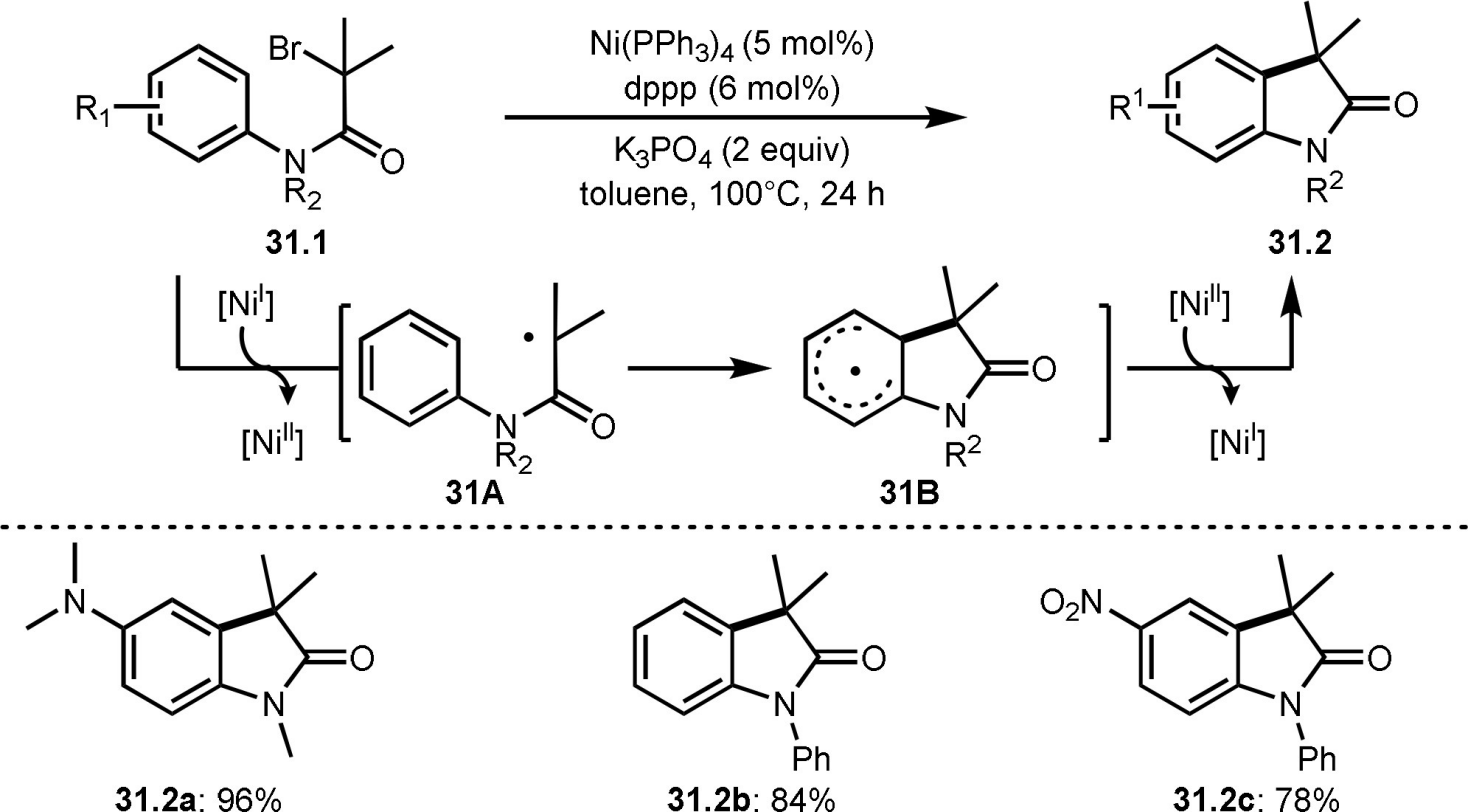
Ni‐catalyzed intramolecular C−H cyclization.

*tert*‐Alkyl radicals (α‐*tert*‐radicals) generated from an α‐bromocarbonyl compounds are not reactive compared with non‐functionalized radical, such as *tert*‐butyl radical. Therefore, if you want to use α‐*tert*‐radicals for the addition to an aromatic ring, activation of the aromatic ring would be required (See later section). Another way to add α‐*tert*‐radicals to aromatic rings is to use intramolecular reactions. Lei's group reported intramolecular aromatic C−H bond cyclization of **31.1** in the presence of a Ni catalyst (Scheme 31).^[160a]^ After the generation of an α‐tert‐radical from the reaction of Ni^I^ and **31.1**, **31A** was produced. The radicals produced are close to the aromatic rings and are therefore favorable for radical addition to the aromatic rings to obtain **31B**. By using this methodology, they obtained various aromatic cyclization products (**31.2 a**–**c**) possessing an electron‐donating group (EDG) or an electron‐withdrawing group (EWG) in good yields.

**Scheme 32:**
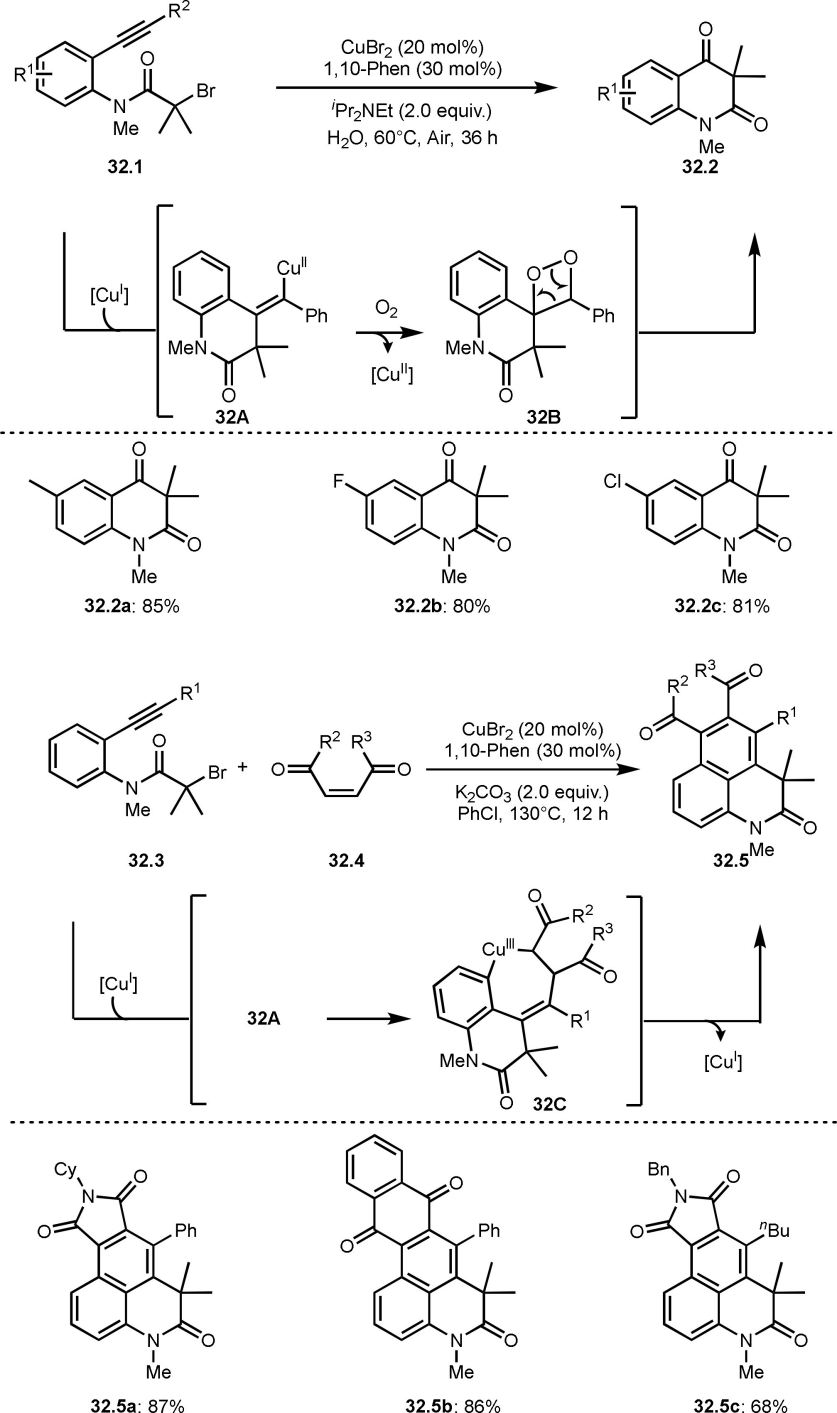
Cu‐catalyzed intra‐ and intermolecular cyclization.

Li and Wei's group reported both inter‐ and intramolecular reaction system to construct multicyclic compounds (Scheme 32).^[161]^ Compound **32.1** possessing alkyne moiety underwent Cu‐catalyzed intramolecular cyclization to produce **32.2**. In this reaction, a key is O_2_ oxidation of **32A**, in turn formed by cyclization of α‐*tert*‐radical from **32.1** followed by capturing Cu^II^. **34A** then reacted with O_2_ to generate **32B**, which underwent C−C bond cleavage to produce **32.2**. On the other hand, the reaction of **32.3** and **32.4** underwent intramolecular cyclization followed by intramolecular cyclization to produce multi‐cyclic products (**32.5**). In the case of the reaction of **32.1**, intermediate **32A** reacted with O_2_, but **32A** reacted with **32.4** instead of O_2_ to produce intermediate **32C**, which underwent a reductive elimination of Cu^II^ to produce **32.5**. Both of the reactions can be accomplished by the controlling the reactivity of vinylic copper species.

### 3.4 Intermolecular Cyclization

**Scheme 33:**
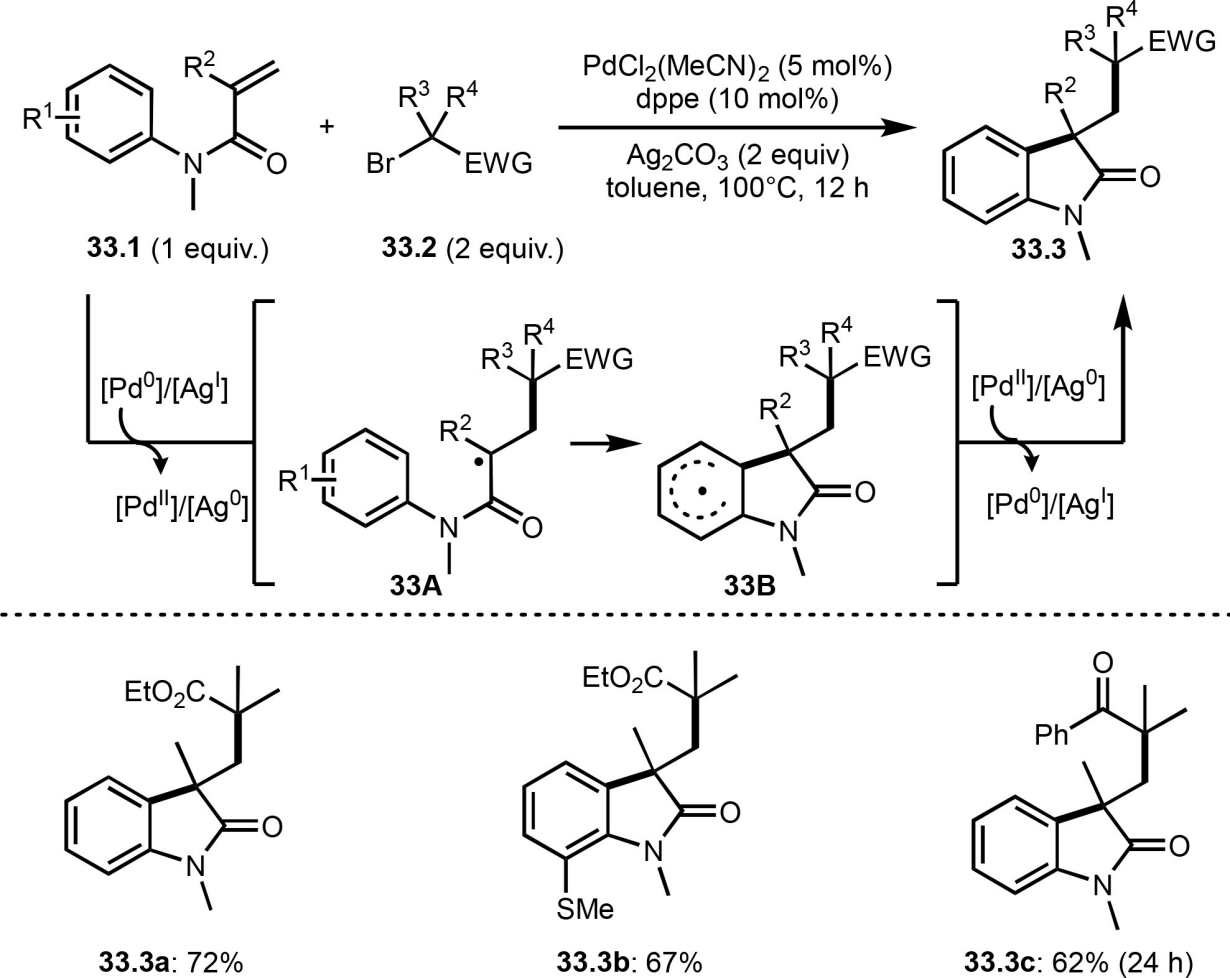
Pd‐catalyzed intermolecular cyclization.

As shown in previous section, aromatic rings are also good α‐*tert*‐radical acceptors under certain conditions. Li's group reported cascade cyclization system that is the reaction of aromatic ring possessing acrylamide moiety (**33.1**) and α‐bromocarbonyl compounds (**33.2**) in the presence of a Pd catalyst (Scheme 33).^[162]^ After the generation of α‐*tert*‐radicals from **33.2**, the α‐*tert*‐radical added to **33.1** to give **33A**. **33A** then underwent an intramolecular cyclization to provide **33B**. Desired cyclized product **33.3** was obtained via re‐aromatization of **33B**. Ag salt accelerated the re‐aromatization process. But Ag salt was not used as a catalyst. Under their conditions, α‐bromesters and α‐bromoketones can be reacted with **33.1** to produce **33.3 a**–**c** in moderate to good yields.

**Scheme 34:**
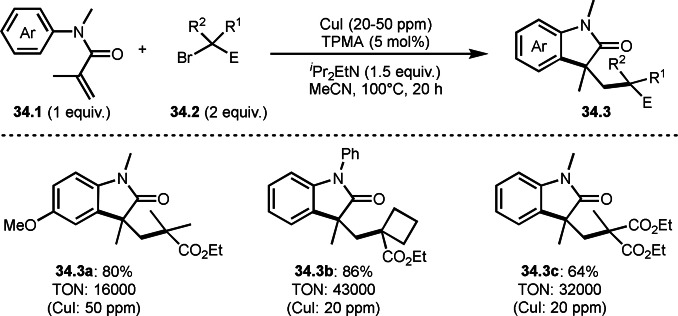
Highly efficient Cu‐catalyzed intermolecular cyclization.

Nishikata's group and Tang's group also reported same reaction pattern as Li's group shown in Scheme 33, in which the reaction of aromatic ring possessing acrylamide moiety (**34.1**) and α‐bromocarbonyl compounds (**34.2**) gave cyclized product (**34.3**) (Scheme 34).^[163]^ They used a Cu salt as a catalyst. Particularly noteworthy is the catalyst loading at the ppm level. For example, the synthesis of **34.3** employed 50 ppm Cu catalyst, which accomplished turn over number (TON), 16000. Similarly, **34.3 b** and **34.3 c** achieved high TONs of 43000 and 32000 respectively. Generally, ppm Cu catalyzed radical reactions using α‐bromocarbonyl compounds are very difficult. During the reaction, maintaining active Cu^I^ catalyst is quite difficult. It would be oxidized to Cu^II^. To prevent this, they used amine as a reductant for Cu salt.^[164]^

**Scheme 35:**
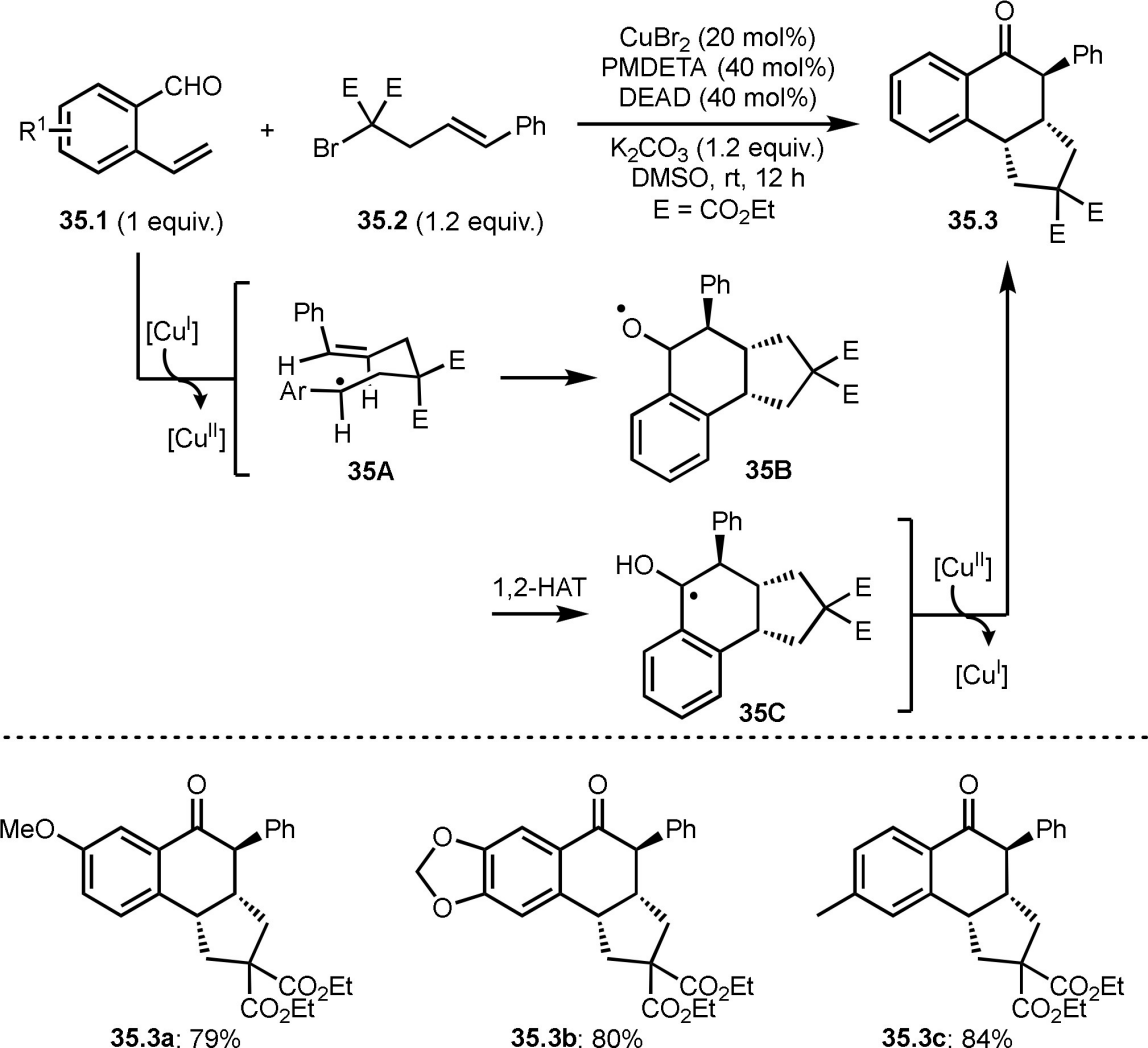
Cu‐catalyzed diastereoselective intermolecular cyclization.

Selective cyclization is one of the difficult issues in this reaction. Zhu's group accomplished diastereoselective intermolecular cyclization (Scheme 35).^[165]^ The reaction of 2‐vinyl benzaldehyde derivatives (**35.1**) and α‐bromocarbonyl compounds possessing a styryl fragment (**35.2**) in the presence of a Cu catalyst occurred to produce multi‐cyclized product (**35.3**). The reaction was diastereoselective. This selectivity is attributed to the selective formation of cyclic radical intermediate **35B** via **35A**. They explained that location of the two larger substituents (Ar and Ph) at the equatorial position of **35A** caused the cis‐substituted five‐membered ring **35B**. **35B** then underwent 1,2‐HAT to give **35C** without isomerization. Final Cu oxidation provided **35.3**. Under those conditions, diverse structures of multicyclic compounds (**35.3 a**–**c**) were synthesized in good yields with perfect diastereoselectivities.

**Scheme 36:**
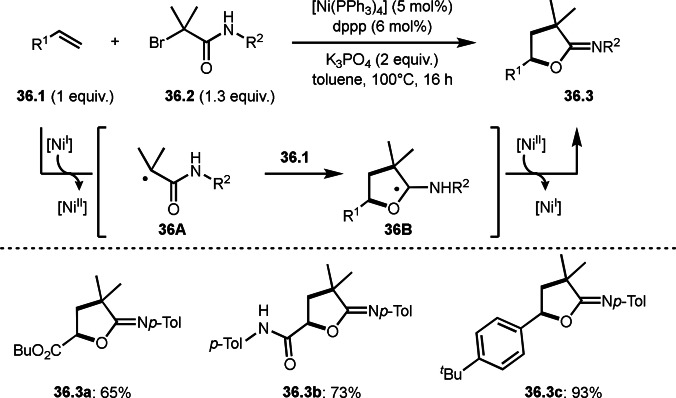
Ni‐catalyzed intermolecular cyclization with carboxamide.

A carboxamide has two nucleophilic atoms (N and O). Lei's group observed *N*‐cyclization reaction to produce iminolactones (**36.3**) in Ni‐catalyzed intermolecular cyclization reactions of olefins (**36.1**) and α‐bromocarboxamides (**36.2**) (Scheme 36).[^166^, ^167^] In this reaction, upon generation of **36A** from the reaction of **36.2** and Ni^I^, **36A** added to **36.1**, followed by radical addition to the carboxamide group to form two‐heteroatom (*O*, *N*) bonded carbon radical intermediate (**36B**). The resulting **36B** was oxidized by Ni^II^ to produce the iminolactone (**36.3**). DFT caluculations, EPR spectroscopy and radical clock experiments supported the proposed mechanism. Acrylesters, acrylamides, and styrenes can be used as an olefin substrate, which produced **36.3 a**–**c** in good yields. Generated iminolactones underwent hydrolysis to provide lactones. They also demonstrated the reaction of olefins with α‐bromoesters to produce lactones possessing a quaternary carbon centre.

**Scheme 37:**
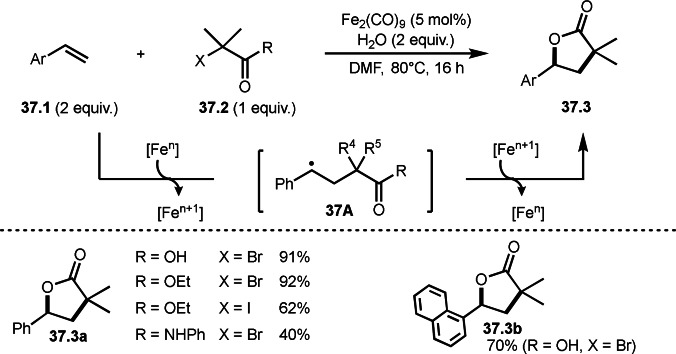
Fe‐catalyzed intermolecular cyclization.

An iron salt is also good α‐*tert*‐radical generator from α‐bromo (or idodo) carbonyl compounds (Scheme 37).^[168]^ Iwasaki and Nishihara's group reported intramolecular cyclization reactions of styrenes (**37.1**) and α‐bromocarbonyl compounds (**37.2**) to produce γ‐lactones (**37.3**). Interestingly, α‐bromoacids, esters, and carboxamides can be used for this reaction under iron‐catalyzed conditions. The reaction of iron salt and **37.2** generated α‐*tert*‐radical, which then reacted with **37.1** to form intermediate **37A**, efficiently. α‐Bromoacids, and esters resulted in **37.3 a** in good yields, whereas α‐bromocarboxamides gave moderated yields. Compared with Ni‐catalyst system reported by Lei's group shown in Scheme 36, iron‐catalyzed system is not suitable for this purpose.

**Scheme 38:**
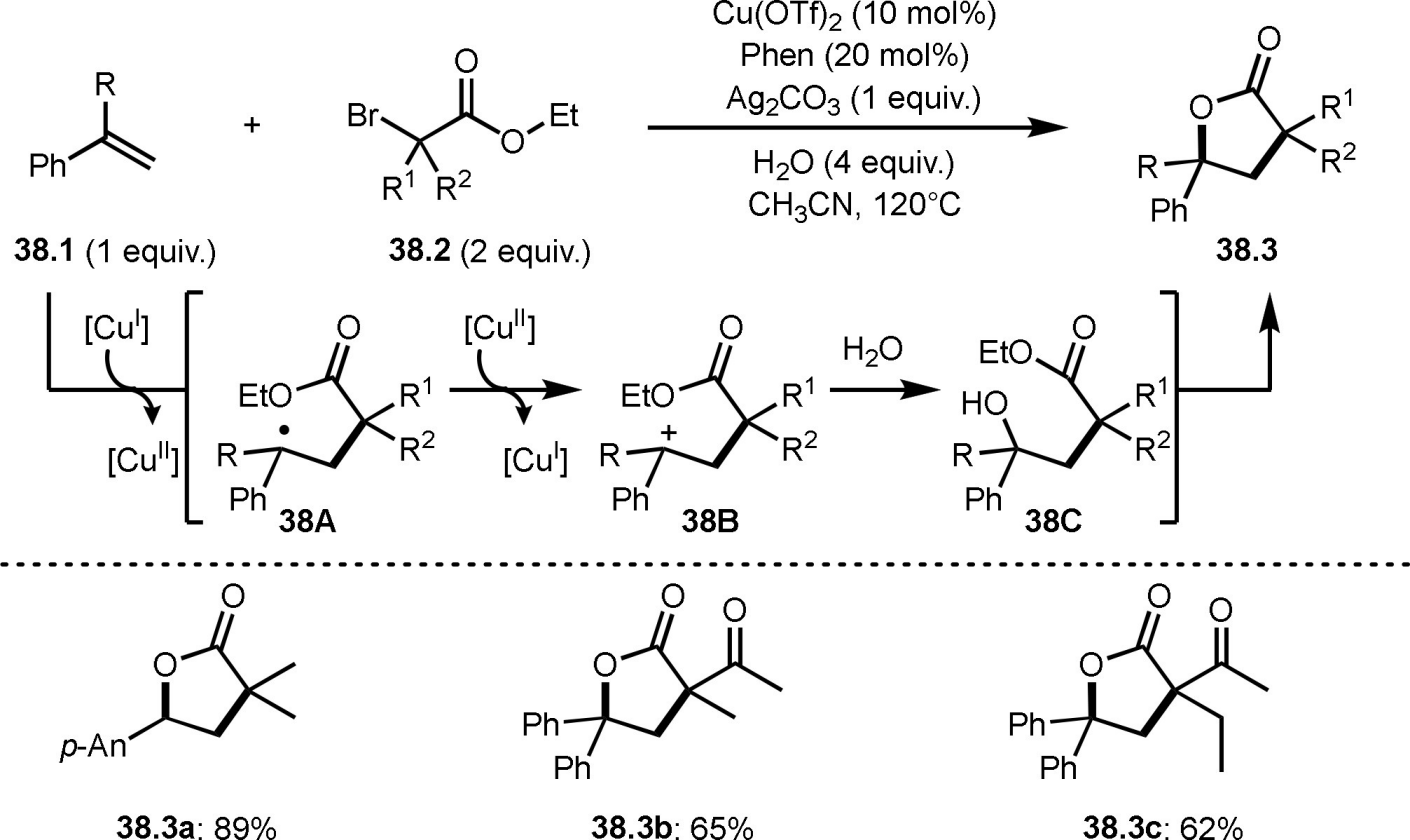
Cu‐catalyzed intermolecular cyclization via water addition

Li's group reported Cu‐catalyzed intermolecular cyclization reactions via a cation intermediate (Scheme 38).^[169]^ The reaction of styrenes (**38.1**) and α‐bromocarbonyl compounds (**38.2**) produced γ‐lactones (**38.3**). They employed α‐bromoesters and 2‐bromoketoesters as a *tert*‐alkyl source, and various γ‐lactone structures (**38.3 a**–**c**) were synthesized in moderate‐good yields. Olefin substrates were not only single substituted styrenes but also 1,1‐diarylsubstituted alkenes. Electron‐rich styrenes were generally reactive due to α‐*tert*‐alkyl radical from α‐bromocarbonyl compounds are basically electron‐deficient radicals. The proposed reaction mechanism employed radical/cation cross‐over system. α‐*tert*‐Alkyl radical generated from the reaction of Cu^I^ and **38.2** first added to **38.1** to give a radical intermediate **38A**. The resulting **38A** was oxidized by Cu^II^ to form a cation intermediate **38B**. **38B** then reacted with water to produce hydroxylated intermeidater **38C**, which underwent intramolecular esterification to produce γ‐lactones (**38.3**). In above introduced cyclization reactions including lactones and iminolactones, a radical cyclization occurred. But the reaction of water played an important role in this cyclization. To prove this step, they carried out the reaction with ^18^O labeled water. As a result, ^18^O was incorporated into the product. The oxygen atom of the newly formed ester group is from water.

**Scheme 39:**
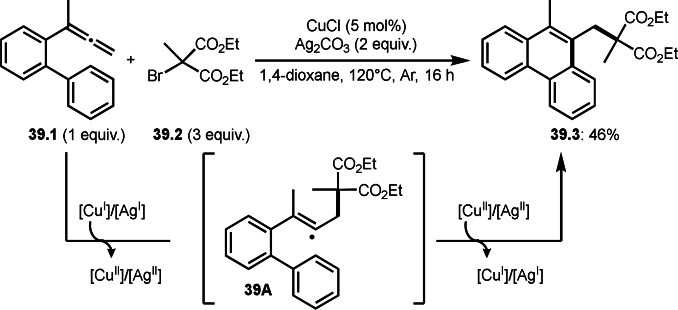
Cu‐catalyzed intermolecular cyclization with allene.

Styrenes, acrylate derivatives were good reaction partners for the cyclizations. Chen and Li's group reported Cu‐catalyzed intramolecular reactions with allenes (Scheme 39).^[170]^ The reaction of allenes (**39.1**) and α‐bromocarbonyl compounds (**39.2**) produced cyclized products (**39.3**). α‐*tert*‐Alkyl radical generated from **41.2** reacted with a terminal alkene of allenes (**39.1**) to give **39A**. **39A** underwent intramolecular aromatic C−H cyclization to produce **39.3**.

**Scheme 40:**
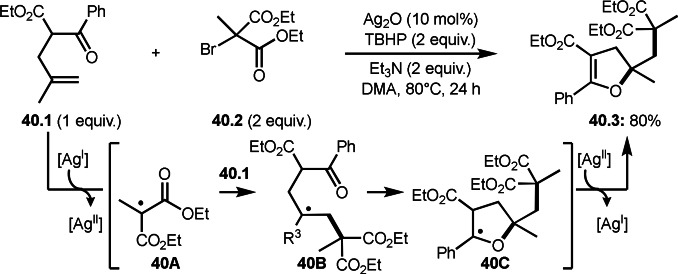
Ag‐catalyzed intermolecular cyclization.

Song and Li's group extended the intermolecular cyclization with α‐bromocarbonyl compounds (Scheme 40).^[171]^ They employed 1,1‐dialkyl substituted olefins (**40.1**) as a substrate. The reaction of **40.1** and 2‐bromomolanetes (**40.2**) produced cyclized products (**40.3**). Sterically bulky and electron‐deficient α‐*tert*‐alkyl radicals from α‐bromocarbonyl compounds generally have low reactivities toward unactivated olefins, but the reaction smoothly occurred well in this case. This high reactivity is not clear at this stage, but it might be due to silver catalyst system. The reaction started to generate α‐*tert*‐alkyl radicals from **40.2**, which added to **40.1** to form intermediate **40B**. A carbon radical of **40B** attacked the oxygen atom of C=O group to generate intermediate **40C**. **40C** was oxidized by Ag salt or TBHP followed by proton elimination to produce **40.3**.

**Scheme 41:**
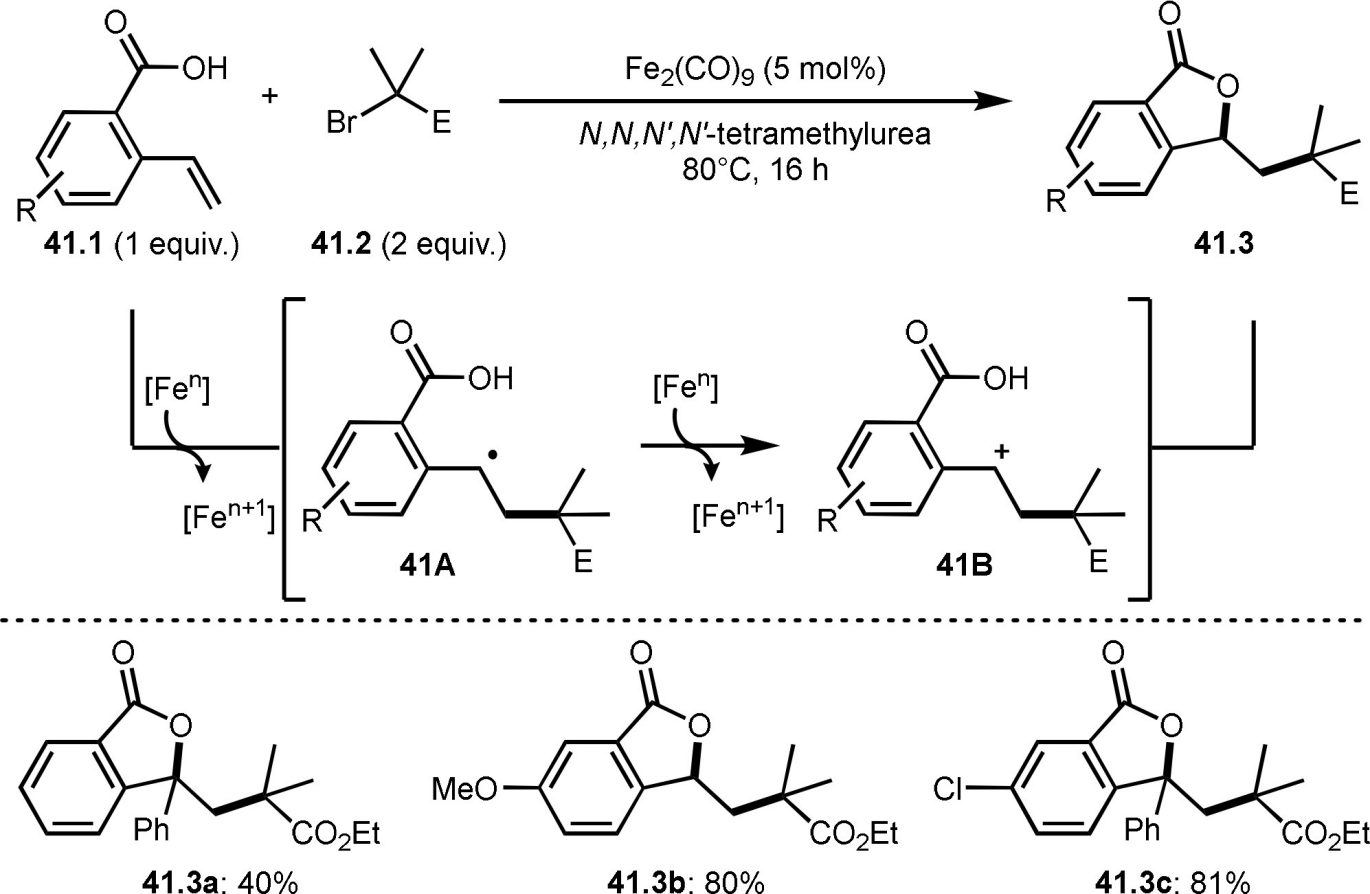
Fe‐catalyzed intermolecular cyclization.

Benzoic acids possessing a vinyl group are also good reaction partner in intermolecular cyclization (Scheme 41).^[172]^ Iwasaki and Nishihara's group reported iron‐catalyzed tert‐alkylative cyclization of *o*‐vinylbenzoic acids (**41.1**) and α‐bromocarbonyl compounds (**41.2**) to give cyclization products (**41.3 a**–**c**). In this reaction, iron acted as both an α‐tert‐alkyl radical generator and an oxidant. After the generation of α‐tert‐alkyl radicals followed by the addition to **41.1**, **41A** was formed. **41A** was then oxidized by Iron salt to produce a cation intermediate **41B**. The nucleophilic attack to the carboxylic acid on the carbocation of **41B** resulted in the formation of the targeted cyclization product (**41.3**).

**Scheme 42:**
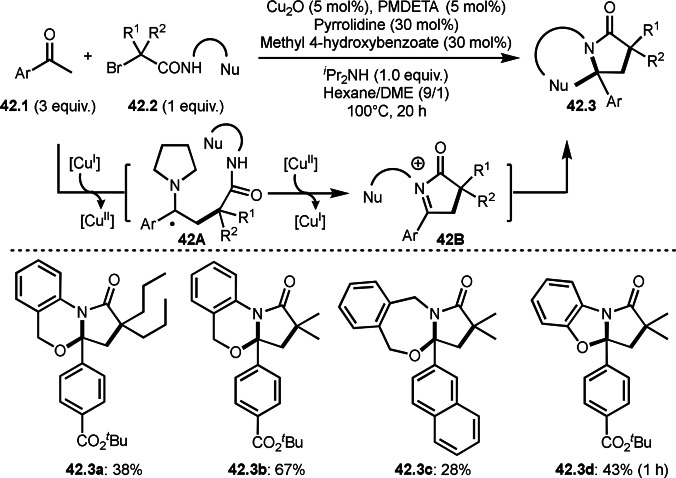
Cu‐catalyzed cascade intermolecular cyclization.

Nishikata's group unveiled a new strategy for preparing sterically congested γ‐lactam‐based multi‐heterocycles, which feature in a number of biologically relevant systems (Scheme 42).^[173a]^ They showed that a Cu‐catalyzed radical/iminium domino strategy (RIDS), in which ketones (**42.1**) are reacted with α‐bromocarboxamides (**42.2**) bearing alcohol, phenol, or carboxylate nucleophiles, enabled the efficient syntheses of γ‐lactam‐based multi‐heterocyclic structures (**42.3**) possessing quaternary carbons. In their reaction, both catalytically generated enamines from the reaction of **42.1** and pyrrolidine and iminiums (**42B**) were the key intermediates to control a cascade cyclization system. Under optimized conditions, highly congested [5,5], [5,6], and [5,7] fused rings (**42.3 a**–**d**) were synthesized in moderate yields.

**Scheme 43:**
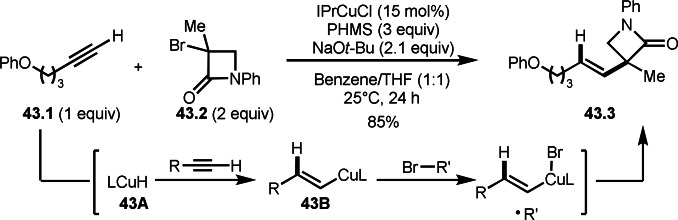
Hydroalkylation of alkyne catalyzed by a Cu salt.

A reaction of an alkyne, an α‐bromocarbonyl and hydrogen source undergoes hydroalkylation in the presence of a Cu catalyst. Lalic's group reported that the reaction of alkyne **43.1**, α‐bromocarboxamide **43.2** and PMHS (polymethylhydrosiloxane) gave *E‐*alkenes **43.3** (Scheme 43).[^135^, ^174^] Although main α‐bromocarbonyl reagent was secondary alkyl group, only **43.2** was able to be used as a tert‐alkyl source. Different from ATRA, the reaction starts with hydrocupration of **43.1** by **43A** to generate **43B**. The selectivity is determined at this hydrocupration step. The resulting **43B** undergoes SET to **43.2** giving an alkenyl copper(II) species and a carbon‐centered radical which reacting together provided the target trans **43.3**.

**Scheme 44:**
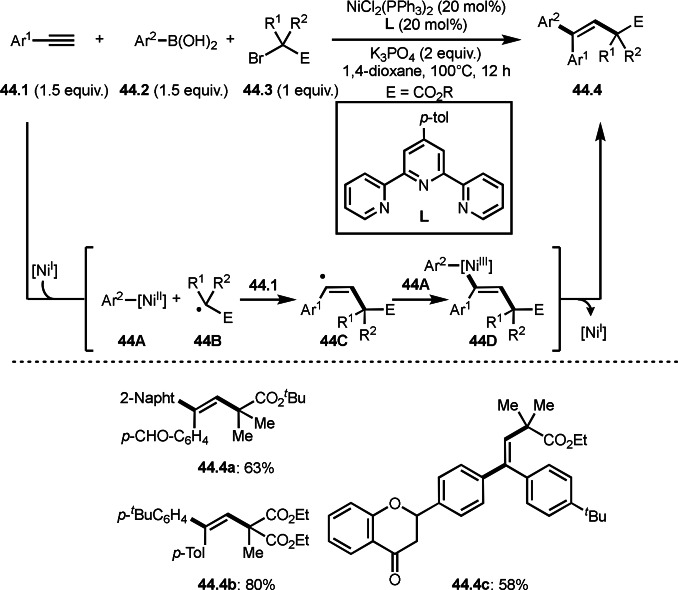
Three‐component reaction to give trisubstituted alkenes.

A three‐component reaction is useful to synthesize trisubstituted alkenes. Nevado's group reported the reaction of terminal alkynes (**44.1**), boronic acids (**44.2**), and α‐bromocarbonyl compounds (**44.3**) in the presence of a nickel catalyst (Scheme 44).[^142^, ^175^] The reaction produced tert‐alkylated trisubstituted alkenes (**44.4**) in moderate to good yields with perfect selectivities. Mechanism of this reaction starts with transmetalation between **44.2** and Ni salt to produce **44A**. **44C** was selectively generated from the reaction of **44.1** and **44B**. The key to the selective production of **44.4** is that **44C** is not *E*/*Z* isomerized, despite its very crowded structure. **44C** was then captured by **44A**. Final reductive elimination of **44D** afforded **44.4**. The high selectivity may be attributed to the formation of ATRA like intermediate before formation of **44C** but they excluded the intermediate base on the control experiments.

**Scheme 45:**
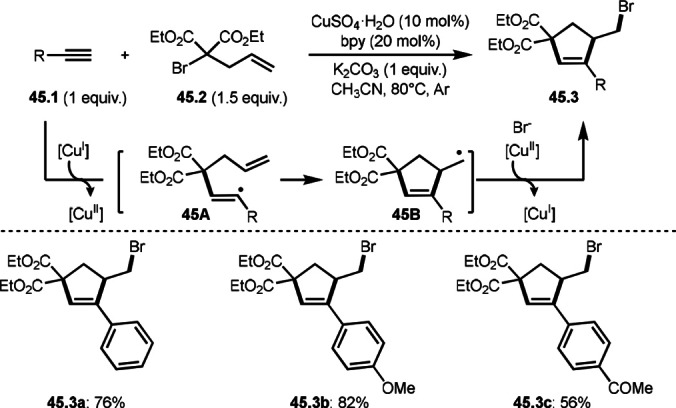
Cu‐catalyzed ATRA/ATRC reaction.

Alkynes are an interesting reactant in intermolecular cyclizations. Tang's group reported radical cascade cyclization to produce five‐membered ring possessing both a C−C double bond and C−Br bond (Scheme 45).^[176a]^ The reaction of alkynes (**45.1**) and α‐bromocarbonyl (malonate) compounds (**45.2**) to give cyclization products (**45.3 a**–**c**). The reaction of Cu salt and **45.2** followed by the addition of α‐tert‐alkyl radical to **45.1** produced radical intermediate **45A**. **45A** underwent cyclization to give **45B**. Finally, **45.3** was obtained via Cu oxidation followed by coupling with [Cu^II^]−Br. This tandem‐ATRA/ATRC process enabled the synthesis of various Br‐substituted cyclopentenes.

**Scheme 46:**
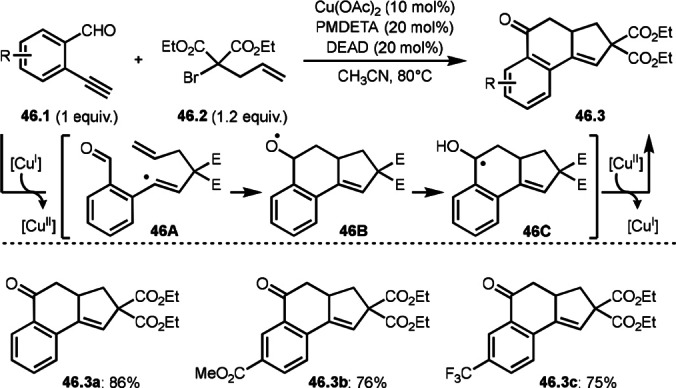
Cu‐catalyzed cascade intermolecular cyclizations of alkynylated arenes.

Zhu's group developed a Cu‐catalyzed cascade intermolecular cyclizations of enynals (**46.1**) with alkenylated α‐bromocarbonyl (malonate) compounds (**46.2**), yielding various cyclohexenone‐fused multicyclic compounds (**46.3 a**–**c**) (Scheme 46).^[177]^ Ketone formation is difficult via the addition of radical species but they accomplished the reaction by using 1,2‐H atom shift. The reaction of **46.2** with Cu salt to produce α‐tert‐alkyl radical. The α‐tert‐alkyl radical added to **46.1** to form a vinylic radical intermediate (**46A**). An intramolecular addition of vinylic radical (**46A**) to the aldehyde group generated alkoxy radical intermediate (**46B**) followed by a formal 1,2‐H shift to give the benzyl radical (**46C**). **46.3** was finally produced by Cu‐oxidation followed by proton elimination. Alkylated α‐bromocarbonyl (malonate) compounds also reacted with **46.1** to produce cyclohexenone‐fused polycyclic compounds possessing α,β‐unsaturated carbonyl moiety.

**Scheme 47:**
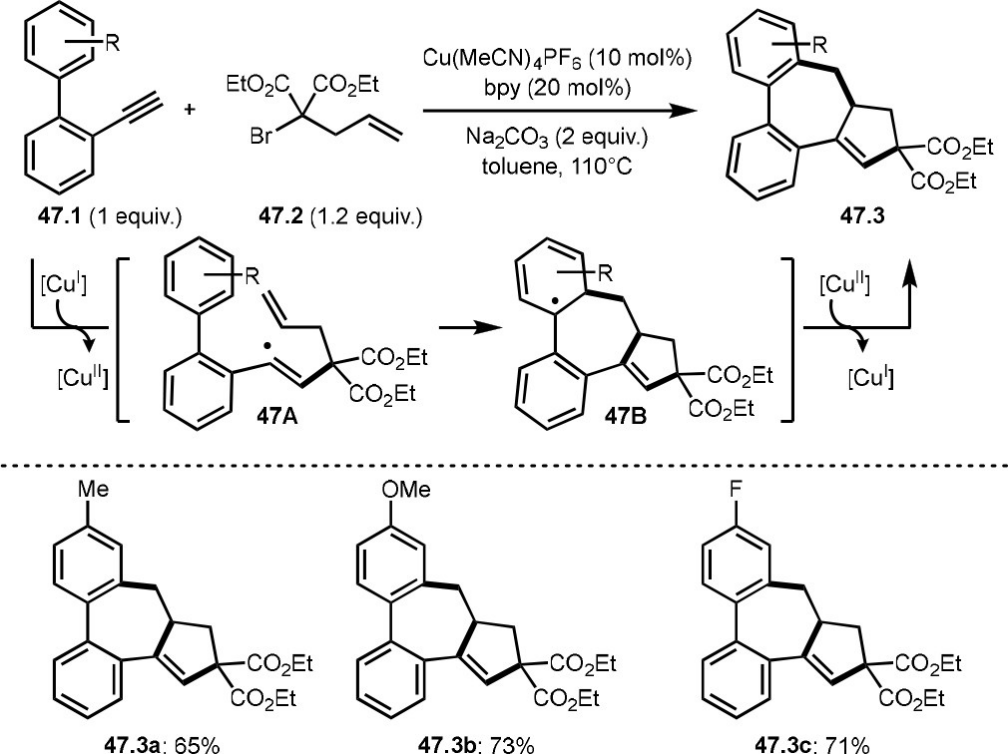
Cu‐catalyzed cascade intermolecular cyclizations of alkynylated arenes.

Zhu's group further developed a Cu‐catalyzed cascade intermolecular cyclizations of alkynylated arenes (**47.1**) with alkenylated α‐bromocarbonyl (malonate) compounds (**47.2**), yielding various dibenzocycloheptanes (**47.3 a**–**c**) (Scheme 47).^[178]^ The reaction of Cu^I^ and **47.2** generated α‐tert‐alkyl radical followed by the addition to **47.1** to form a vinylic radical intermediate (**47A**). **47A** underwent cascade radical additions to produce a seven‐membered ring radical intermediate (**47B**). **47.3** was produced from the Cu‐oxidation of **47B** followed by re‐aromatization. Under their conditions, various seven‐membered ring‐fused biaryls (**47.3 a**–**c**) in moderate to good yields.

**Scheme 48:**
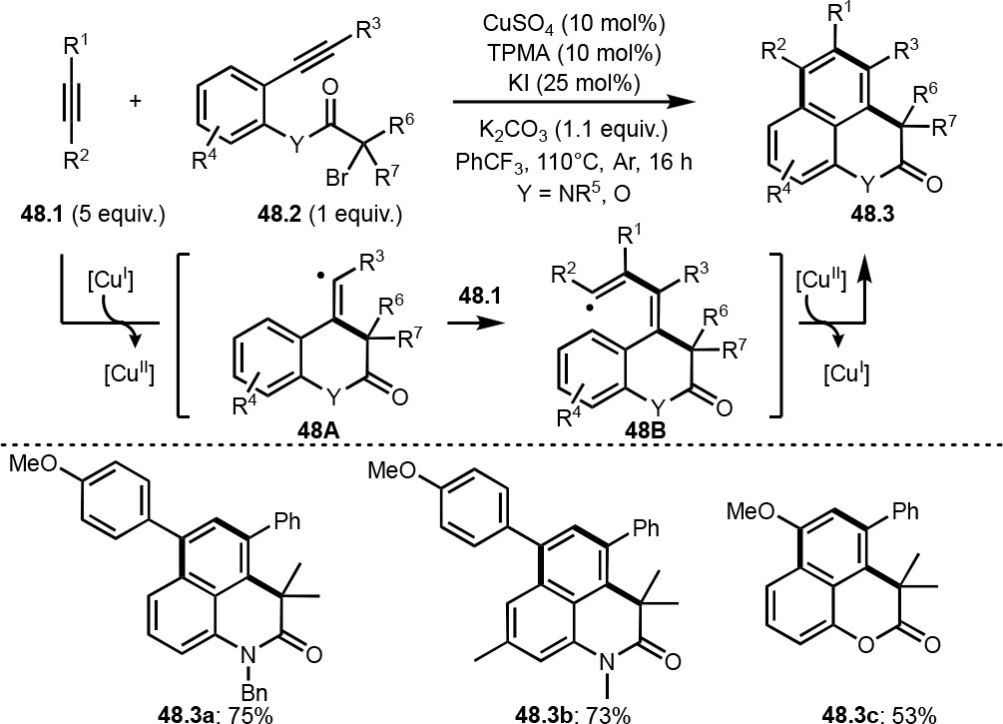
Cu‐catalyzed cascade intermolecular cyclization reaction of alkyne.

Li and He's group reported cascade intermolecular cyclization reactions (Scheme 48).^[179]^ In the presence of Cu catalyst, the reaction of alkynes (**48.1**) with alkynylated α‐bromocarbonyl compounds (**48.2**) occurred to give heteropolycycles (**48.3 a**–**c**). The α‐tert‐alkyl radicals were generated from the reaction of Cu^I^ and **48.2** followed by radical cyclization to form **48A**. **48A** then immediately reacted with **48.1** to give **48B**. Next, smooth aromatic C−H cyclization followed by Cu^II^ oxidation/re‐aromatization occurred to give **48.3**. Addition of KI was effective to improve the yield. KI is considered to be an electron‐transfer reagent, which supports the reduction of Cu^II^. Both a terminal and internal alkyne can be applied to this reaction, but terminal alkyne was much more reactive than the internal one.

**Scheme 49:**
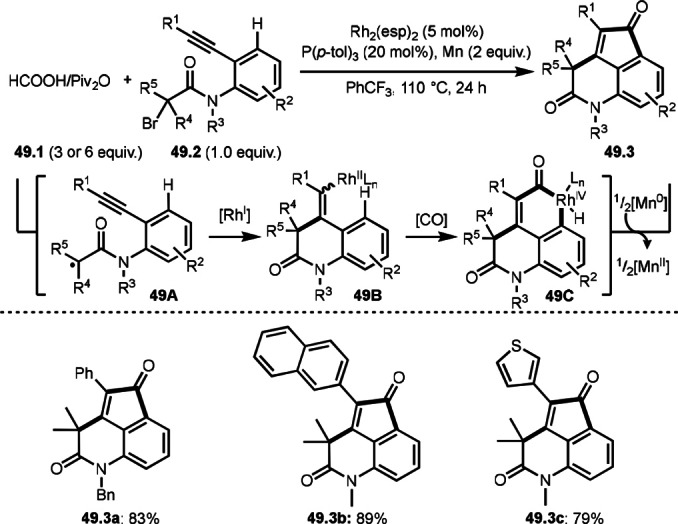
Rh‐catalyzed cascade intermolecular cyclization reaction.

He and Li's group extended their previous reaction shown in Scheme 48. They performed a formal carborhodation/C−H carbonylation in intermolecular cyclization reactions (Scheme 49).^[180]^ The reaction of formic acid/pivalic anhydride (**49.1**) with alkynylated α‐bromocarbonyl compounds (**49.2**) in the presence of a Rh catalyst (esp:α,α,α′,α′‐tetramethyl‐1,3‐benzenedipropionic acid) occurred to give heteropolycycles (**49.3 a**–**c**). In this reaction, a reductive *trans*‐*tert*‐alkylacylation toward alkyne of **49.2** occurred. *tert*‐Alkyl radicals were generated from homolysis of the C−Br bond of **49.2** to give **49A**. **49A** then underwent cyclization to generate a vinylic radical intermediate, which was then captured by Rh^I^ species to form a carborhodation intermediate **49B**. Mn was effective to reduce Rh^II^ to Rh^I^. The resulting carborhodation intermediate **49B** underwent CO insertion followed by reductive elimination to produce **49.3**.

**Scheme 50:**
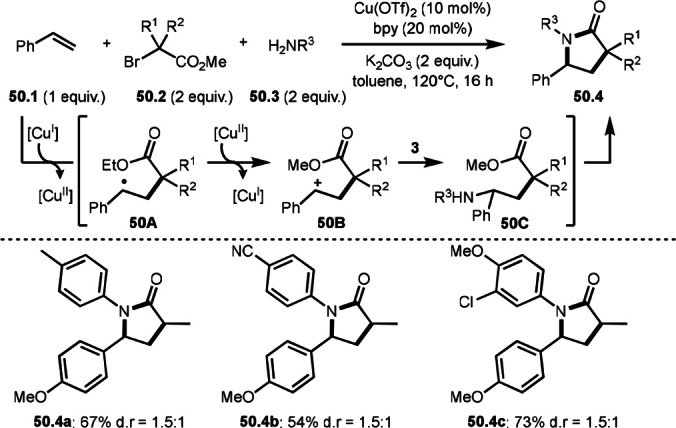
Cu‐catalyzed intermolecular cyclization reaction to give a lactam.

Hull reported three component reaction of styrenes, α‐bromocarbonyl compounds, and amines to produce *tert*‐alkylaminated compounds in Scheme 9. On the other hand, Song and Li's group reported that the reaction of styrenes (**50.1**), α‐bromocarbonyl compounds (**50.2**), and amines (**50.3**) to produce intermolecular cyclization products, lactams (**50.4**) (Scheme 50).^[181]^ Employed substrates were same but the products were different from Hull's result shown in Scheme 9. The reaction started to generate α‐*tert*‐alkyl radical from **50.2**. α‐*tert*‐Alkyl radical then added to **50.1** to form **50A**. **50A** was oxidized by Cu^II^ to give a cation intermediate **50B**, which reacted with **50.3** to produce **50C**. Although Hull's reaction was stopped at this stage to produce acyclic aminated product **50C**, but Song and Li's reaction underwent further cyclization of **50C** to produce the lactam (**50.4**). This difference is attributed to reactant amine. Primary amines produced lactams, whereas secondary amines were acyclic products.

**Scheme 51:**
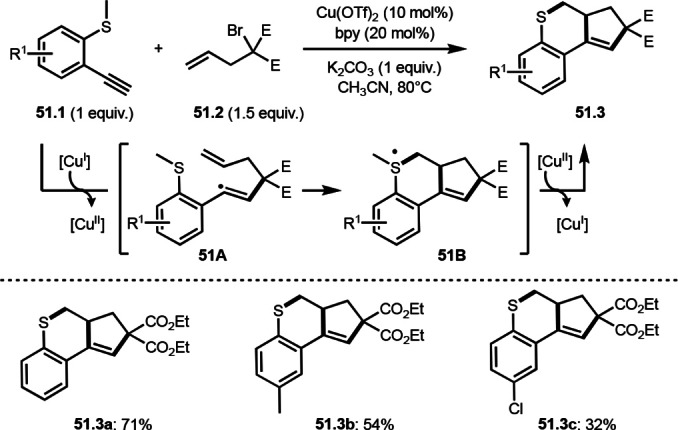
Cu‐catalyzed C−S bond formation in intermolecular cyclization reaction.

A C−S bond formation is possible by means of intermolecular cyclization reaction, which was reported by Gao and Li's group (Scheme 51).^[182]^ Arylethynylsulfanes (**52.1**) reacted with allylated α‐bromocarbonyl compounds (**51.2**) to produce *S*‐embedded multi‐cyclized products (**51.3 a**–**c**) in moderate to good yields. The reaction started to generate α‐*tert*‐alkyl radical from (**51.2**). A vinylic radical intermediate **51A** was generated after cyclization of **51.1** and α‐*tert*‐alkyl radical. 51A then underwent an intramolecular cyclization to produce sulfur radical intermediate (**51B**). Interestingly, the radical (**51B**) released Me radical followed by Cu^II^ oxidation to produce **51.3**. *S*‐Ethyl and benzyl substituted **51.1** also gave **51.3**. In the case of benzyl substituted **51.1**, BnBr was detected as a side product, which showed that S−C bond cleavage was a radical process.

**Scheme 52:**
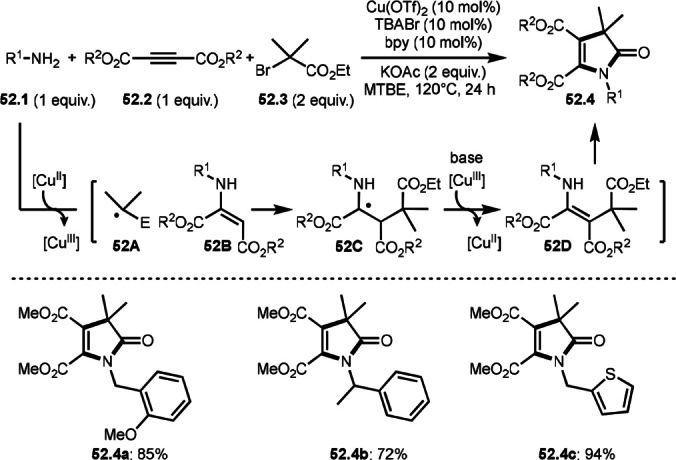
Cu‐catalyzed intermolecular cyclization reaction with internal alkyne.

Cheng's group reported the synthesis of fully substituted 1,3‐dihydro‐2H‐pyrrol‐2‐ones (**52.4**) through a highly congested tetrasubstituted alkene intermediate (Scheme 52).^[183]^ The reaction of amines (**52.1**), internal alkynes (**52.2**), and α‐bromocarbonyl compounds (**52.3**) to produce intermolecular cyclization products (**52.4 a**–**c**). The reaction of generated α‐*tert*‐alkyl radical (**52A**) from **52.3** and trisubstituted alkene (**52B**) gave a radical intermediate **52C**. Trisubstituted alkene is generally difficult to react with α‐*tert*‐alkyl radical due to the steric hinderance, but smooth reaction occurred to generate tetrasubstituted alkene (**52D**). The desired product (**52.4**) was then generated from intramolecular amidation of **52D**. They also checked the reactivity of isolated **52B** with **52.3**, and cyclized products (**52.4**) were obtained via the same mechanism. This result showed that α‐*tert*‐alkyl radical (**52A**) did not first react with internal alkynes (**52.2**).

**Scheme 53:**
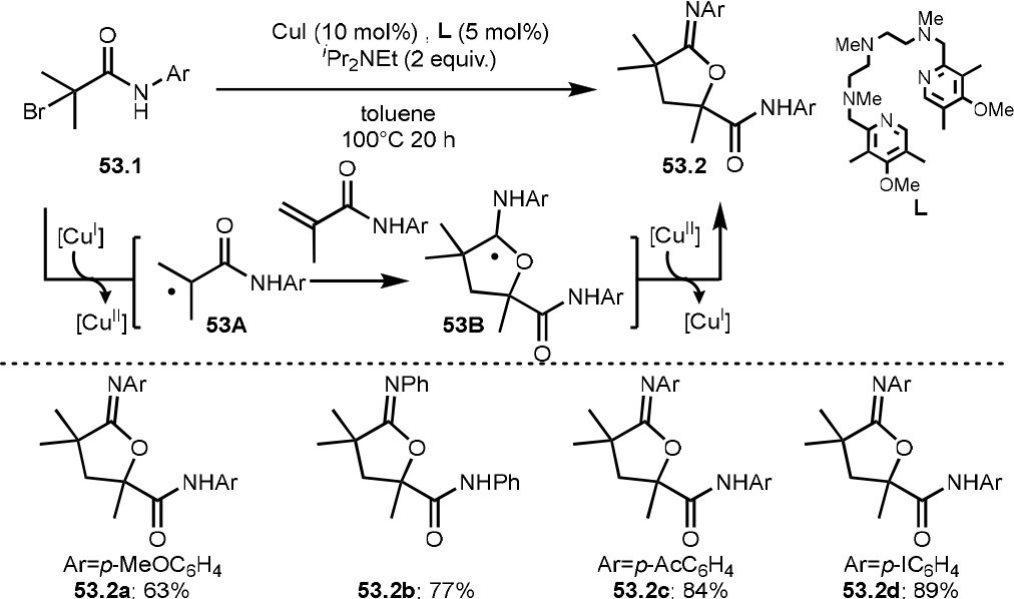
Cu‐catalyzed homodimerization reaction.

Intermolecular cyclization reactions generally employ two different molecules to carry out cascade reaction process. Another way of carrying out this reaction is to use homodimerizations from a single substrate; however, this reaction is very difficult because two different substrates have to be generated from one substrate. Nishikata's group found that the efficient control of generation of a radical and acrylamide from α‐bromocarboxamides enables an iminolactonization reaction in the presence of a copper catalyst with a ligand containing five nitrogen atoms (Scheme 53).^[184]^ Generally, α‐bromocarboxamides are known to generate the corresponding α‐*tert*‐alkyl radicals, but β‐hydrogen elimination to form acrylamide can also occur. This protocol uncovers a new type of radical homodimerization. The reaction of α‐bromocarboxamides (**53.1**) in the presence of Cu/L and amine base gave homodimerized products (**53.2 a**–**d**) in good yields. The ligand was effective to generate both α‐*tert*‐alkyl radical (**53A**) and methacrylamide, which underwent a radical addition to form a radical intermediate (**53B**). Homodimerized product was obtained via Cu^II^ oxidation followed by deprotonatation.

**Scheme 54:**
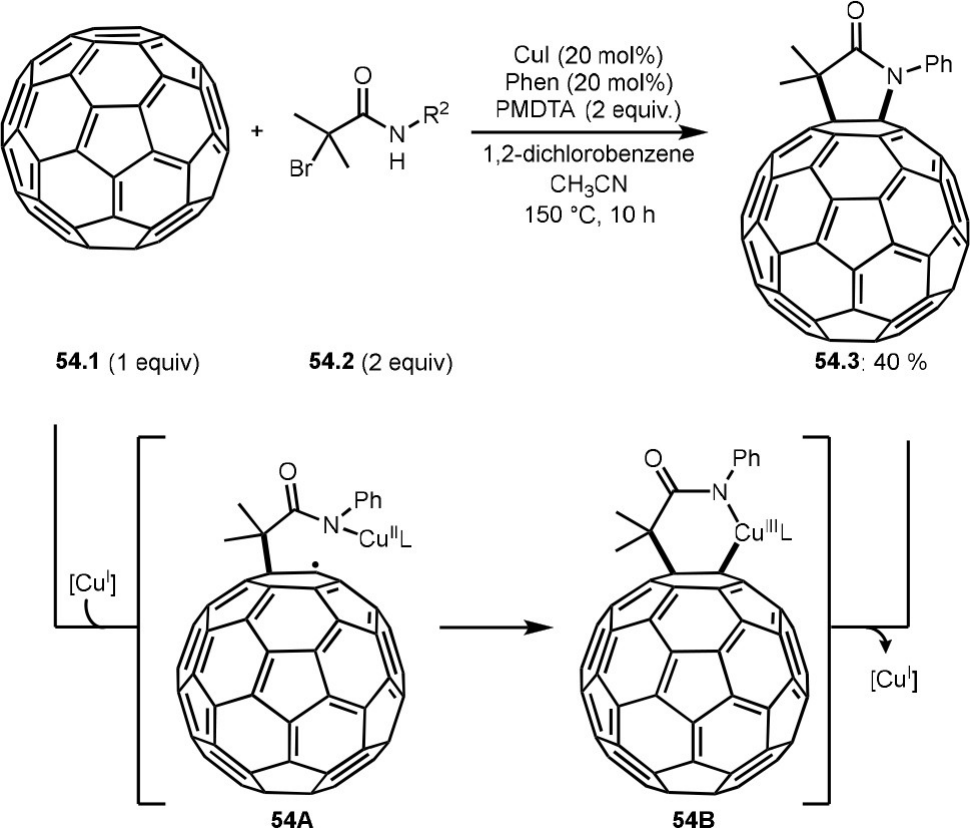
Intermolecular cyclization reaction with [60]fullerene.

Alkenes, and alkynes were a good radical acceptor in intermolecular cyclization reaction. α‐*tert*‐Alkyl radicals generated are first added to one of the substrates. On the other hand, the aromatic C−C double bond of [60]fullerene (C60) also serves good reaction site for radicals. Chen, Yang, and Wang's group reported Cu‐catalyzed radical heteroannulation reaction of C60 (**54.1**) with α‐bromocarboxamide (**54.2**) to produce C60‐fused lactam (**54.3**) in 40 % yield (Scheme 54).^[185]^ An α‐*tert*‐alkyl radical generated was added to C60 to form **54A**. As the result, **54.3** was obtained via reductive elimination of **54B**.

### 3.5 Rearrangement

**Scheme 55:**
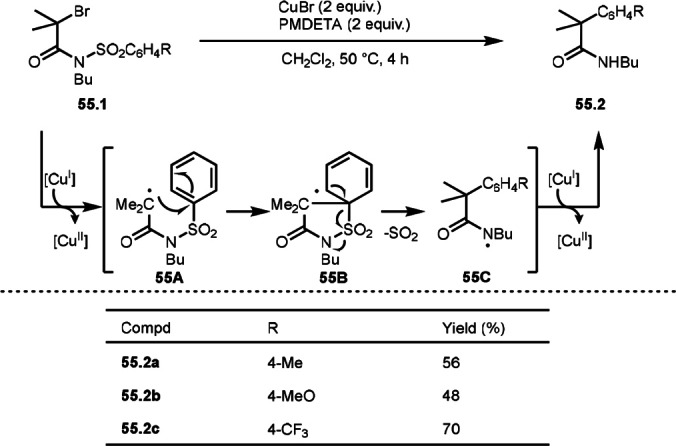
Cu‐mediated aryl rearrangement.

α‐*tert*‐Alkyl radicals from α‐bromocarbonyl compounds smoothly react with unsaturated bonds or reactive aromatic rings. On the other hand, α‐*tert*‐alkyl radical can undergo a rearrangement reaction in the presence of a metal catalyst. Clark's group discovered Cu‐mediated aryl transfer from α‐bromocarboxamides possessing a sulfonamide moiety with loss of SO_2_ (Scheme 55).[^186^, ^187^] Reaction of *N*‐alkyl‐*N*‐arylsulfonyl‐2‐bromopropionamides **55.1** with a Cu salt led to 2‐aryl propionamides (**55.2 a**–**c**) via a 1,4‐aryl migration. Initially, a generated α‐*tert*‐alkyl radical from the reaction of Cu catalyst and **55.1** produced **55A**. A carbon radical of **55A** added to *ipso*‐position of arylsulfonyl group to give **55B**. **55B** underwent re‐aromatization with loss of SO_2_ to produce **55C**. Finally, **55C** was reduced by Cu^I^ and then protonated to produce **55.2**. Yang's group also reported similar Cu‐mediated reactions.^[187]^

**Scheme 56:**
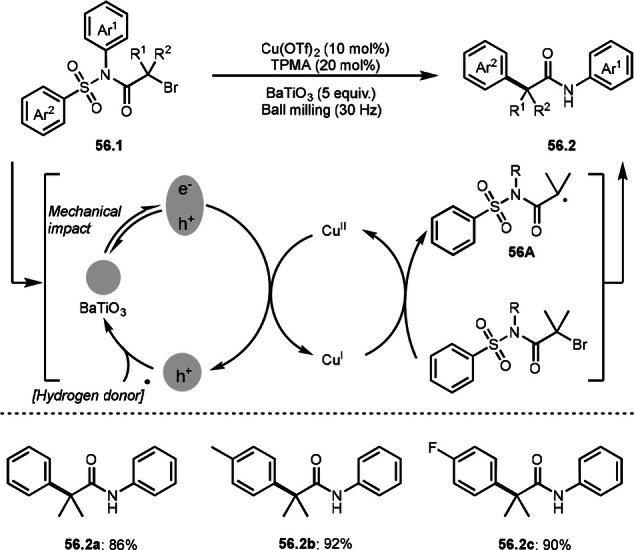
Mechanochemical Cu‐catalyzed aryl rearrangement

Aryl rearrangement shown in Scheme 55 required excess Cu salt. Because Cu salt acted as both a radical initiator and an amidyl radical reductant. The highly polarized BaTiO_3_ (ball milling) enabled efficient reduction of Cu^II^ to Cu^I^, which realized a Cu‐catalyzed intramolecular aryl rearrangement reaction of α‐bromocarboxamides possessing a sulfonamide moiety reported by a research group of Lv, Fang and Zheng (Scheme 56).^[188]^ A mechanical impact of BaTiO_3_ (30 Hz) generated a piezoelectron, which reduced Cu^II^ salt to Cu^I^. Cu^I^ reacted with **56.1** to produce **56A**. As in Scheme 55, **56A** underwent intramolecular cyclization followed by SO_2_ evolution/reduction/protonation to give **56.2**. Under those catalytic conditions, **56.2 a**–**c** were synthesized in good yields.

**Scheme 57:**
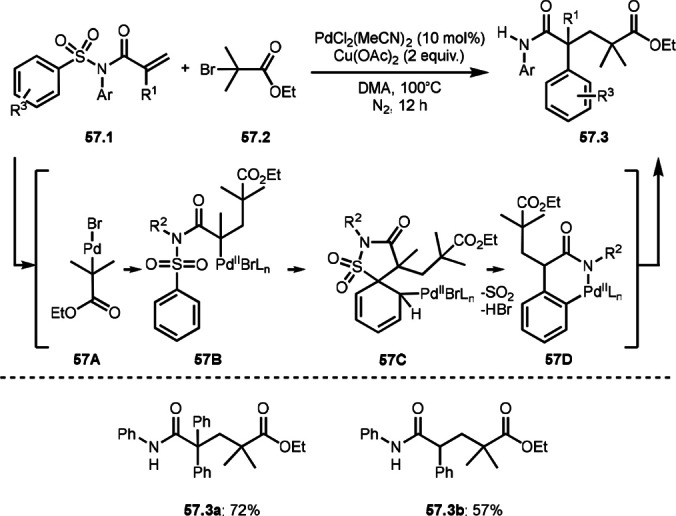
Pd‐catalyzed aryl rearrangement.

Li's group nicely extended intramolecular aryl rearrangement reaction of α‐bromocarboxamides possessing a sulfonamide moiety to intermolecular reaction. They carried out the intramolecular aryl rearrangement reaction of acryl amides (**57.1**) possessing a sulfonamide moiety with α‐bromoester in the presence of a Pd catalyst and Cu^II^ oxidant (Scheme 57).^[189]^ The reaction of **57.1** and **57.2** gave addition/rearrangement products (**57.3 a**–**c**). Their reaction mechanism employed insertion of the active Pd^II^ species into the C−Br bond of **57.1** gave *tert*‐alkyl Pd intermediate **57A**. **57A** added to **57.1** to form **57B**. **57B** underwent electrophilic C−H palladation to give **57C**, which then produced **57D** after re‐aromatization. **57.3** was produced from reductive elimination of **57D**. They explained the role of Cu salt is oxidant for Pd^0^ to produce active Pd^II^ species. But Pd^II^ might not be reactive toward **57.2** due to electronic and steric reasons. There are no scientific proofs but basically this reaction is Cu‐mediated reaction and Pd accelerates some steps in the catalytic cycle.

**Scheme 58:**
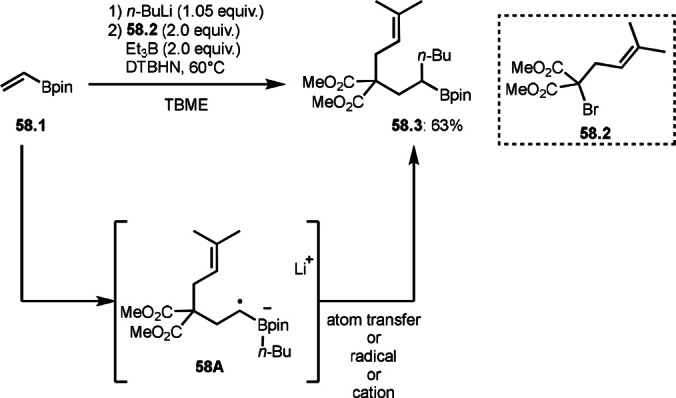
Butyl rearrangement.

Renaud's group reported vinyl boron transformations by using α‐bromocarbonyl compounds (Scheme 58).^[190]^ Vinyl boronic ester (**58.1**) reacted with *n*‐buthyl lithium to give a boron ate complex. The resulting boron ate complex then reacted with an α‐*tert*‐alkyl radical generated from **58.2** in the presence of Et_3_B/di‐tert‐butylhyponitrite (DTBHN), a radical initiator, to produce an anion radical intermediate (**58A**). Finally, the butyl group moved the α‐position of the boron atom via an atom transfer, radical, or cation process to produce **58.3**. The reaction is convenient to prepare functionalized α‐branched alkylboronate esters. They also synthesized **58.3** by using vinyl boronic ester and *n*‐buthyl lithium in the absence of a radical initiator.^[191]^

### 3.6 C−C Coupling Reaction

**Scheme 59:**
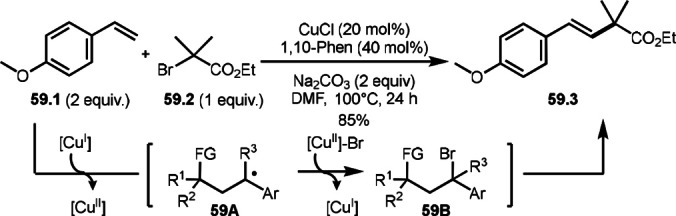
Cu‐catalyzed ATRS in the presence of a bidentate ligand.

Lei's group reported Cu‐catalyzed Heck‐like olefination (ATRS) (Scheme 59).^[192]^ Nishikata's group reported Cu‐catalyzed similar reaction by using a tridentate nitrogen ligand (PMDETA) at room temperature,^[126]^ whereas Le's group employed a bidentate ligand (1,10‐Phen). Generally, the formation of α‐*tert*‐alkyl radicals from α‐bromocarbonyl compounds with tridentate or more nitrogen ligands is much more effective than with bidentate ligands.^[114]^ With this reason, Li's reaction required high reaction temperature. The reaction mechanism is exactly the same with bidentate ligands, which is involving α‐*tert*‐alkyl radical generation from **59.2** followed by ATRA through **59A** to give **59B**. Finally, styrene terminal hydrogen atom (**59.1**) was substituted by *tert*‐alkyl group to produce Heck‐like olefination product (**59.3**).

**Scheme 60:**
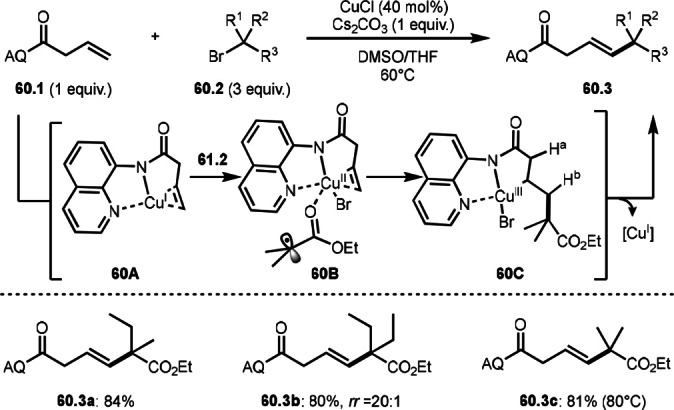
Cu‐catalyzed directed tert‐alkylation.

α‐*tert*‐Alkyl radicals from α‐bromocarbonyl compounds are bulky and less reactive than simple *tert*‐alkyl radical without α‐carbonyl group. Therefore, α‐*tert*‐alkyl radicals are not reactive to unactivated olefins. Guan and Bi's group dissolved this subtle problem in α‐bromocarbonyl chemistry. They bonded a directing group to unactivated olefins that were effective to undergo a smooth radical addition (Scheme 60).^[193]^ The reaction of **60.1** with aminoquinoline (AQ) directing group and **60.2** in the presence of a Cu catalyst gave *trans*‐Heck‐like products (**60.3 a**–**c**). A Cu ligated to **60.1** to form **60A**, which underwent SET reaction between **60A** and **60.2** to give **60B**. The resulting α‐*tert*‐alkyl radical species added to **60.1** followed by β‐hydrogen elimination to produce *trans*‐**60.3**. Both proximal H^a^ (with respect to C=O) and distal H^b^ elimination is possible, but distal products were exclusively formed. Conformationally, H^b^ elimination might be preferred to undergo β‐hydrogen elimination.

**Scheme 61:**
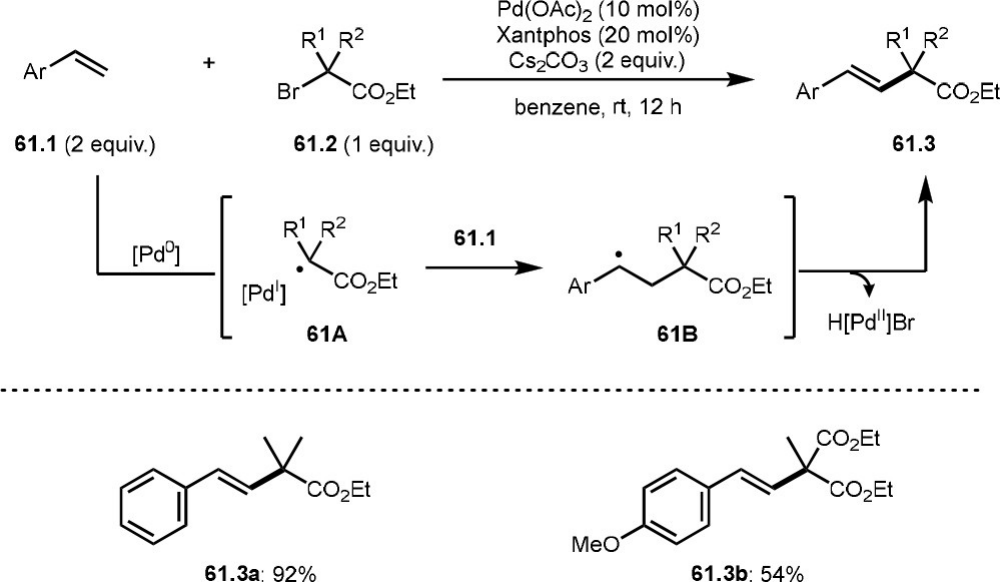
Pd‐catalyzed ATRS.

**Scheme 62:**
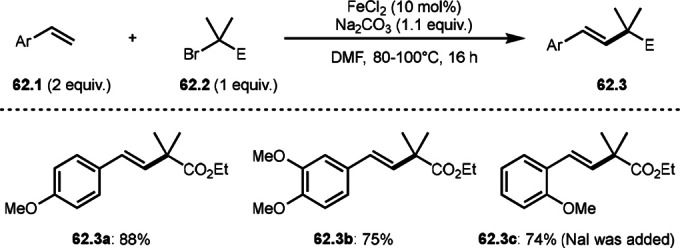
Fe‐catalyzed ATRS.

**Scheme 63:**
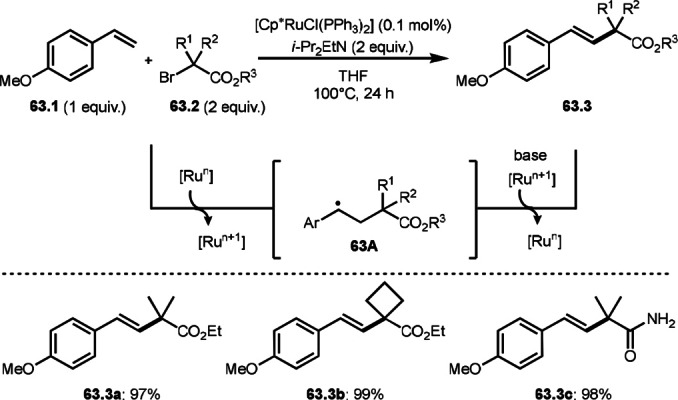
Ru‐catalyzed ATRS.

To undergo ATRS, a Cu catalyst is convenient to generate α‐*tert*‐alkyl radicals from α‐bromocarbonyl compounds. On the other hand, a Pd catalyst (Scheme 61),^[195]^ a Fe catalyst (Scheme 62),^[196]^ and a Ru catalyst (Scheme 63)^[197]^ are also effective to carry out the reactions. Initial α‐*tert*‐alkyl radical generations were accomplished by SET from a transition metal to an α‐bromocarbonyl compound. In the case of Pd and Ru, phosphine ligands were required, but iron‐catalyzed reaction did not require any ligands. Pd‐catalyst system served the mildest conditions, in which the reaction temperature was room temperature. Under those conditions various tert‐alkyl substituted olefins (**61.3**, **61.4**, and **61.5**) were obtained.

**Scheme 64:**
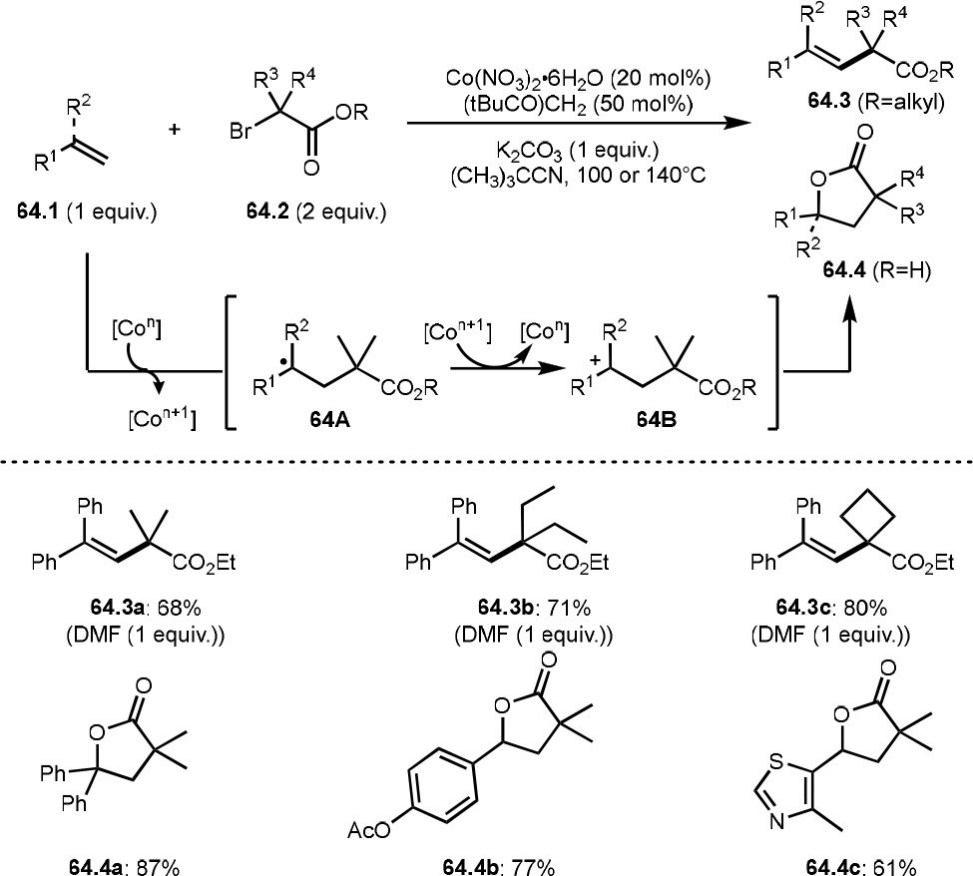
Co‐catalyzed ATRS and lactonization.

A Co catalyst was also possible to undergo ATRS (Scheme 64).^[198]^ Interestingly, both ATRS and cyclization to give lactone was possible in the reaction of **64.1** and **64.2**. The reaction of Co salt and **64.2** generated α‐*tert*‐alkyl radicals, which added to **64.1** to give **64A**. **64A** was then oxidized into a cation intermediate **64B**. **64B** underwent β‐hydrogen elimination to give ATRS product (**64.3**) when esters (**64.2**) were employed. On the other hand, lactone (**64.4**) was obtained when carboxylic acids (**64.2**) were used. In this case, generated carboxylate anion acted as a good nucleophile to undergo an intramolecular cyclization.

**Scheme 65:**
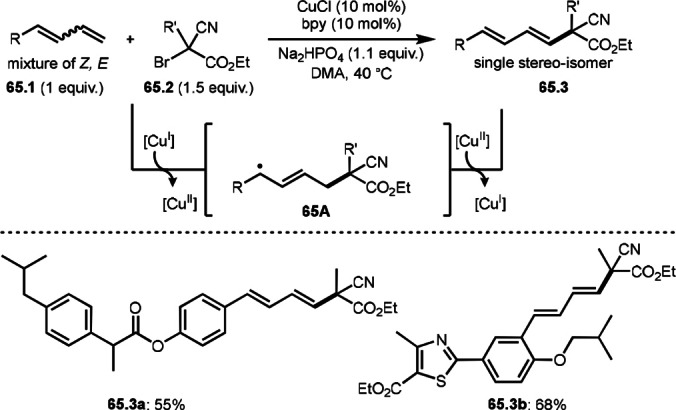
Cu‐catalyzed ATRS with conjugated diene.

ATRS reaction (or Heck‐like olefination) employs single vinylated molecules, such as styrenes. Zhu's group developed ATRS reactions with conjugated dienes in the presence of a Cu catalyst (Scheme 65).^[199]^ The difficulties with using conjugated dienes are the regioselectivity of radical addition and the stereoselectivity of the resulting product. The reaction of diene (**65.1**) and α‐bromocarbonyl compounds (**65.2**) gave ATRS product possessing diene structure (**65.3**). The reaction selectively occurred at the terminal position of diene, and the conjugated diene product was perfectly (*E*)‐selective (**65.3 a** and **b**). They proposed some reaction mechanisms, and radical intermediate (**65A**), which was formed from the reaction of α‐*tert*‐alkyl radicals and **65.1**, was suggested a common intermediate for the mechanism. The reason for the stereoselectivity was not clear, but final HBr elimination process might be an important step to advent the perfect selectivity.

**Scheme 66:**
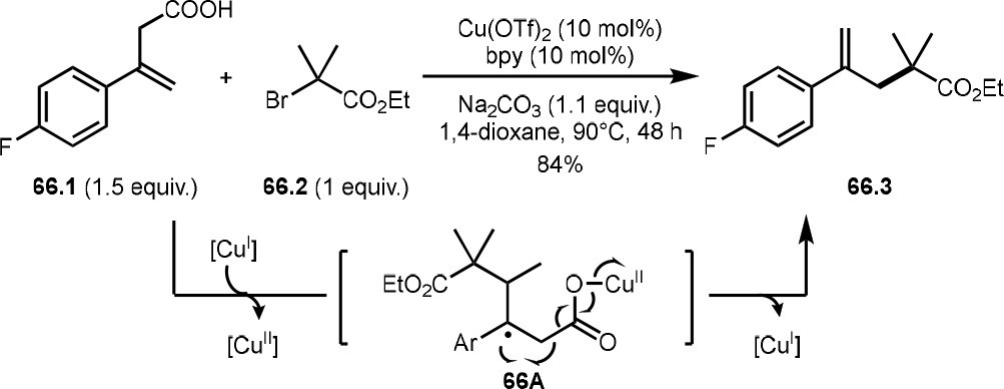
Cu‐catalyzed decarboxylative *tert*‐alkylation.

After the addition of α‐*tert*‐alkyl radicals to unsaturated C−C bonds, the resulting intermediates take specific pathways, such as hydrogen elimination, nucleophilic attack, or cyclization. Another pathway is a C−C cleavage. Wang's group reported a decarboxylative *tert*‐alkylation (Scheme 66).^[200]^ The reaction of conjugated β,γ‐unsaturated carboxylic acid (**66.1**) with α‐bromocarbonyl compound (**66.2**) provided *tert*‐alkylated product (**66.3**) in 84 % yield. α‐*tert*‐alkylation added to **66.1** to form radical intermediate (**66A**). This intermediate underwent decarboxylation to produce **66.3**.

**Scheme 67:**
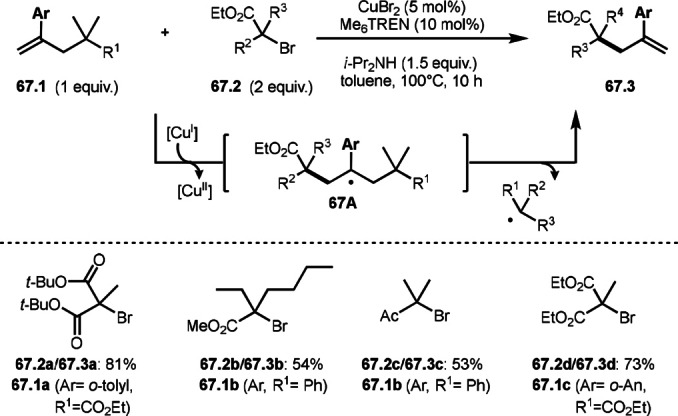
Cu‐catalyzed *tert*‐alkyl exchanging reaction.

Nishikata's group also reported C−C cleavage reaction (Scheme 67).^[201]^ Wang's group employed conjugated β,γ‐unsaturated carboxylic acid (**66.1**), whereas they used tert‐alkylated allylic compound (**67.1**), which was Wang's product (**66.3**). The reaction of **67.1** with **67.2** underwent allylic substitution via a C−C bond cleavage in the presence of a Cu catalyst. The reaction started to generate α‐*tert*‐alkyl radical species from the reaction of **67.2** and Cu^I^. The resulting α‐*tert*‐alkyl radical added to **67.1** to form a radical intermediate **67A**. **67A** then underwent C−C bond cleavage to produce **67.3**. In this case, α‐*tert*‐alkyl radicals were leaving group. Since the reaction could be reversible, two equivalents of **67.2** were required to obtain the targeted products (**67.3 a**–**d**) in good yields. They accomplished various C−C bond cleavages by using their reaction system.

**Scheme 68:**
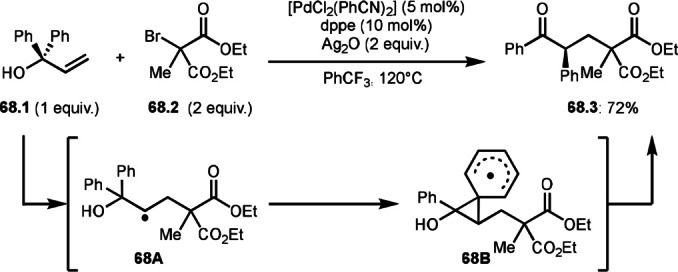
Pd‐catalyzed Ph‐rearrangement reaction.

In Scheme 66 and 67, the leaving groups after C−C bond cleavage did not join the main reaction. On the other hand, Tambar group reported Pd‐catalyzed *tert*‐alkylation via C−C bond cleavage (Scheme 68).^[202a]^ The reaction of **68.1** and **68.2** produced Ph‐rearranged product (**68.3**) in 72 % yield. The role of Pd salt was not described, but it might support the generation of an α‐*tert*‐alkyl radical from **68.2**. The resulting α‐*tert*‐alkyl radical added to **68.1** to form **68A**. Next neophyl‐type rearrangement occurred through **68B** followed by oxidation/carbonyl formation to produce **68.3**. Li's group also reported similar reaction by using photocatalyst system.^[202b]^ Although there are few reports on rearrangements^[203]^ using α‐bromocarbonyl compounds, the synthetic value is high.

**Scheme 69:**
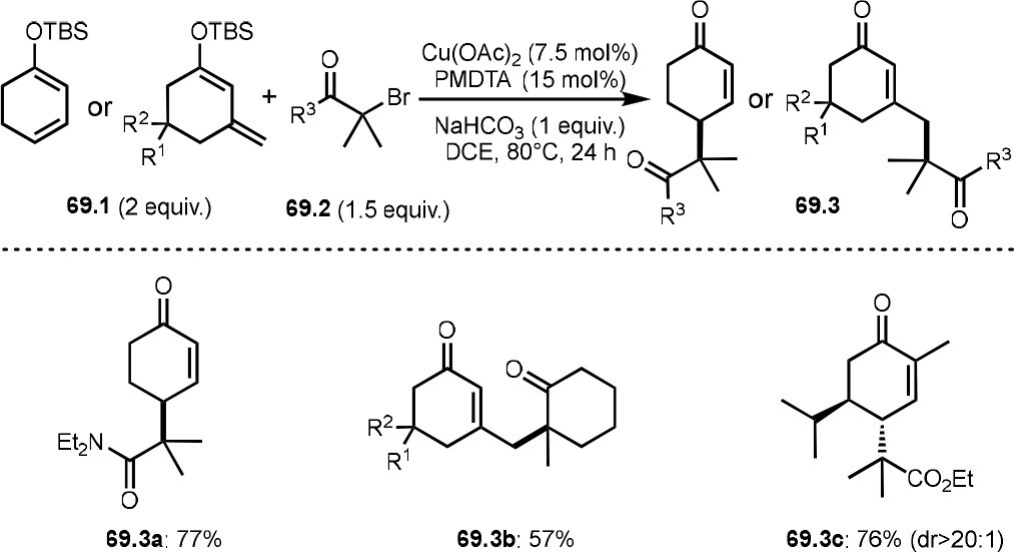
Cu‐catalyzed γ‐tert‐alkylation of silyl enol ethers.

**Scheme 70:**
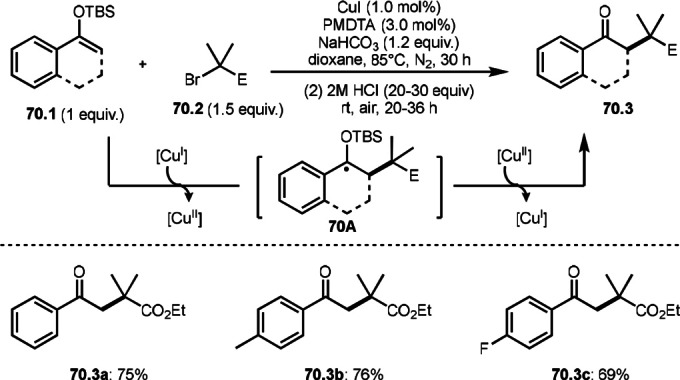
Cu‐catalyzed α‐tert‐alkylation of silyl enol ethers.

As α‐tert‐alkyl radicals are electron‐deficient radical species, α‐tert‐alkyl radicals react smoothly with electron‐rich C−C double bonds. One of the electron‐rich C−C double bonds is an enolate, which is generated from a ketone in the presence of a base. However, an enolate is not a good substrate for α‐tert‐alkyl radicals due to its stability. To obtain sufficient concentrations of enolates, silyl enol ethers are ideal. Mohr's group successfully obtained γ‐alkylation products (**69.3**) from the reaction of silyl enol ethers (**69.1**) and α‐bromocarbonyl compounds (**69.2**) in the presence of a Cu catalyst (Scheme 69).^[204]^ Silyl enol ethers possessing an electron‐rich C−C double bond has enough time to react with short life α‐tert‐alkyl radicals. Under their conditions, both internal and terminal olefin of **69.1** reacted with α‐tert‐alkyl radicals to produce 1,6‐dicarbonyls, γ‐alkylation products (**69.3**), in moderate to good yields. Later, Li also reported the reaction of silyl enol ethers (**70.1**) and α‐bromocarbonyl compounds (**70.2**) in the presence of a Cu catalyst (Scheme 70).^[205]^

**Scheme 71:**
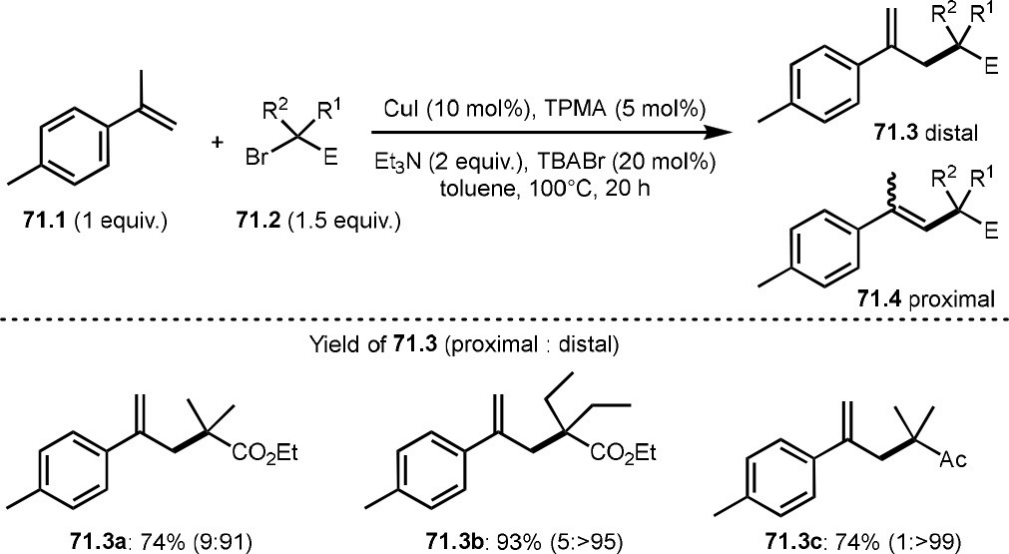
Cu‐catalyzed ATRS to form external olefins.

ATRS reaction smoothly occurs with simple vinyl substituted substrates. This is because α‐tert‐alkyl radical addition is sensitive to the steric bulkiness of olefins. Nishikata's group accomplished ATRS reaction of α‐alkyl styrenes (**71.2**) with α‐bromocarbonyl compounds (**71.2**) in the presence of a Cu catalyst (Scheme 71).^[206]^ Interestingly, the products in this case are external olefins (**71.3**, distal C−C double bond). The reason for this selectivity is not clear, but the final proton abstraction step to form a C−C double bond may determine the selectivity.

**Scheme 72:**
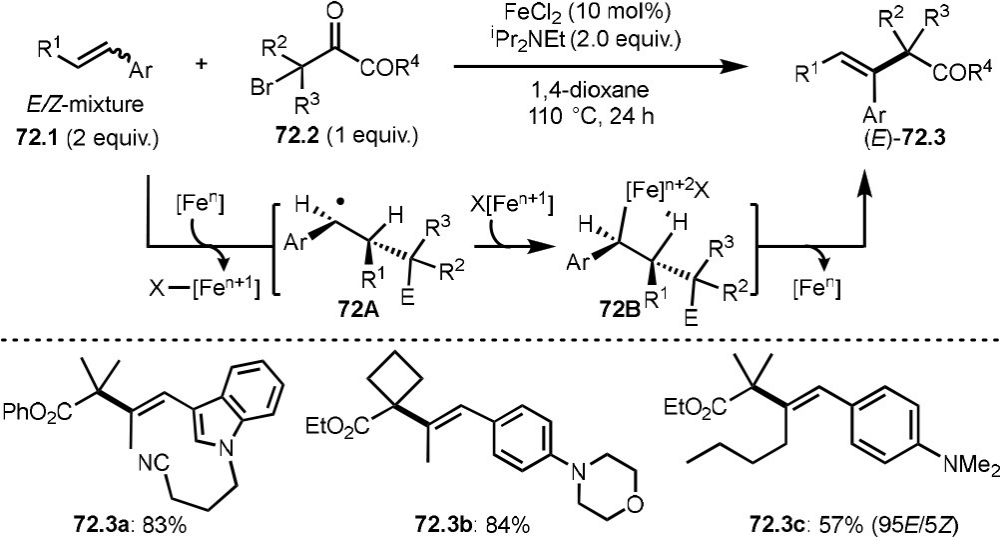
Cu‐catalyzed ATRS with *E*/*Z*‐mixed internal olefins.

Internal olefins are difficult substrate for ATRS reactions because of (*E*) or (*Z*)‐product selectivity issues. Nishikata's group accomplished (*E*)‐selective ATRS reaction in the presence of an iron catalyst (Scheme 72).[^207^, ^208^, ^209^] They employed (*E*) and (*Z*)‐mixed olefins as a starting material, but the selectivities were perfect. They explained the selectivity by using the final β‐hydrogen elimination step in the catalytic cycle. Indeed, the corresponding cyclization reaction also gave trans‐cyclic products via the same intermediate shown in Scheme 72.^[209]^ They conducted some control experiments to understand the reaction. When isolated (*E*)‐ or (*Z*)‐**72.1** was used, only (*E*)‐product (**72.3**) was generated from both substrates. The isomerization of product was not detected during the reaction. These results suggested that the selectivity came from the intermediate.

**Scheme 73:**
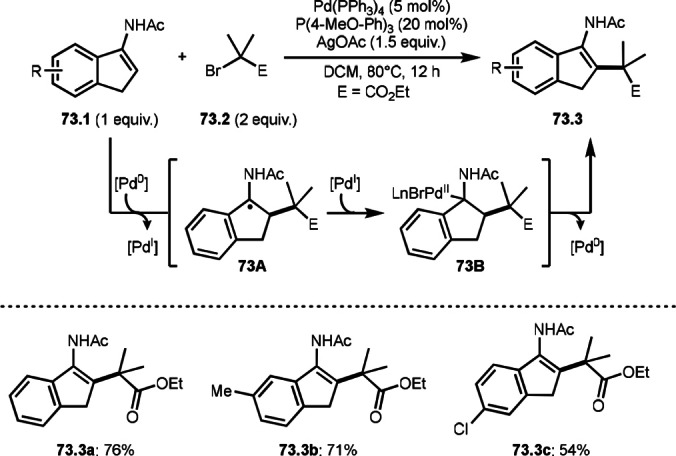
Pd‐catalyzed ATRS with cyclic enamides.

Enamides are electron‐rich vinyl substrates and are considered precursors to aldehydes. Loh's group developed the reaction of cyclic enamides (**73.1**) and α‐bromocarbonyl compounds (**73.2**) in the presence of a Pd catalyst (Scheme 73).^[210a]^ The reaction of Pd^0^ and **73.2** gave α‐*tert*‐alkyl radical species. α‐*tert*‐Alkyl radical species then added to **73.1** to produce **73A**, which was captured by Pd^I^ to give **73B**. Next, **73B** produced **73.3**. The final step to produce **73.3** is not clear, but the reformation of the C−C double bond may be accomplished by the presence of Ag salts, in which reductive elimination of **73B** followed by oxidation of the resulting intermediate by the Ag salts may occur.

**Scheme 74:**
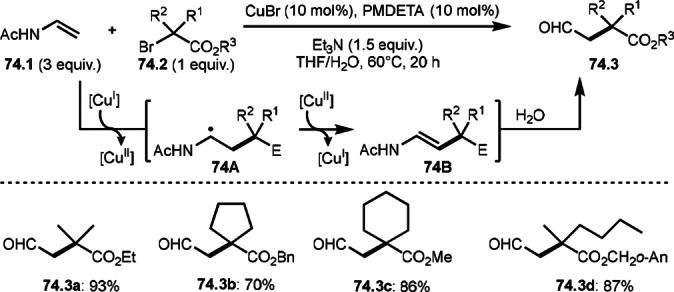
Cu‐catalyzed ATRS with enamides.

**Scheme 75:**
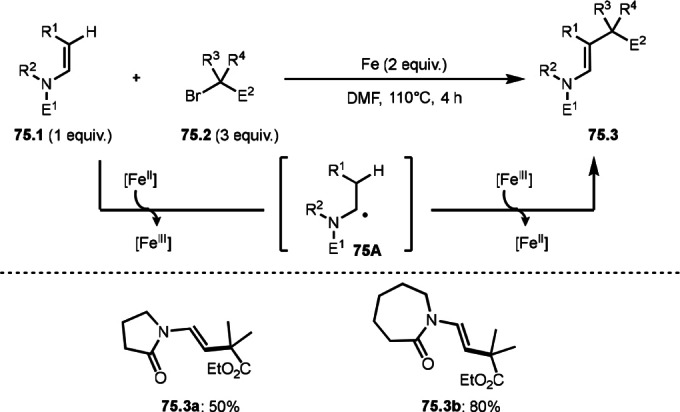
Fe‐mediated ATRS with enamides.

Nishikata's group developed the synthesis of *tert*‐alkylated aldehydes (**74.3**) from the reaction of enamides (**74.1**) and α‐bromocarbonyl compounds (**74.2**) in the presence of a Cu catalyst (Scheme 74).^[211]^ The reaction with enamide first produced ATRS intermediate (**74B**) trough **74A**. **74A** was hydrolyzed to produce *tert*‐alkylated aldehyde (**74.3**). They detected **74B**, when the reaction was carried out in the absence of water. Under those conditions, various aldehydes (**74.3 a**–**d**) can be prepared in good yields. The only drawback to this reaction is that α‐alkyl‐substituted enamides did not show any reactivity. Later, Nicolas and Gillaizeau's group reported an iron mediated ATRS of enamide (**75.1**) with α‐bromocarbonyl compounds (**75.2**) (Scheme 75).^[212]^ They obtained ATRS products (**75.2**) in good yields in the absence of water. The products were trans‐selective.

**Scheme 76:**
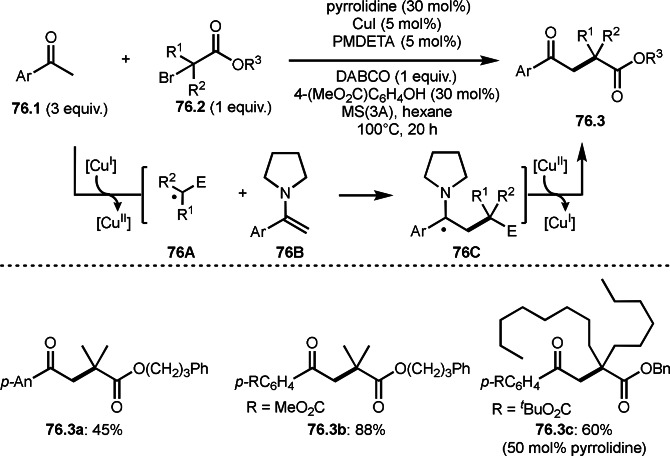
Cu‐catalyzed α‐tert‐alkylation of ketones.

Electron‐rich olefins are good substrates for the addition of α‐tert‐alkyl radical, and an enolate from a ketone is less reactive due to the concentration problem. To dissolve this problem, isolated silyl enol ethers were employed for this purpose. Nishikata's group successfully employed ketones for *tert*‐alkylation by using a catalytically generated enamine as a key intermediate (Scheme 76).^[213]^ In this reaction, ketones (**76.1**) reacted with α‐bromocarbonyl compounds (**76.2**) in the presence of a Cu and pyrrolidine catalyst to produce α‐*tert*‐alkylated ketones (**76.3 a**–**c**) in good yields. The reaction started to produce both α‐*tert*‐alkyl radical (**76A**) and enamine (**76B**). Each intermediate reacted together to form **76C**. Finally, **76.3** was produced via Cu oxidation of **76C** followed by hydrolysis. They isolated **76B** and confirmed that it produced **76.3** after the reaction of **76B** and **76.2**. Under their conditions, highly congested α‐tert‐alkylated ketones, such as **76.3**, were prepared.

**Scheme 77:**
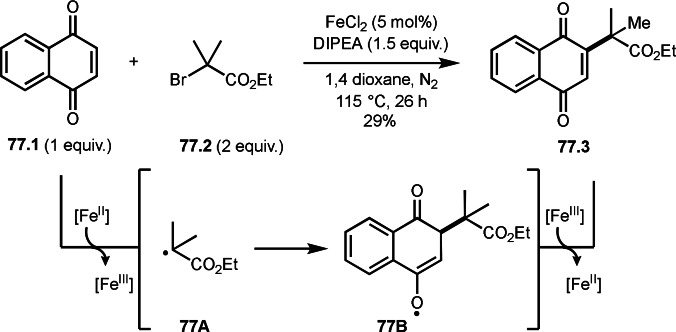
Fe‐catalyzed tert‐alkylation of 1,4‐quinone.

**Scheme 78:**
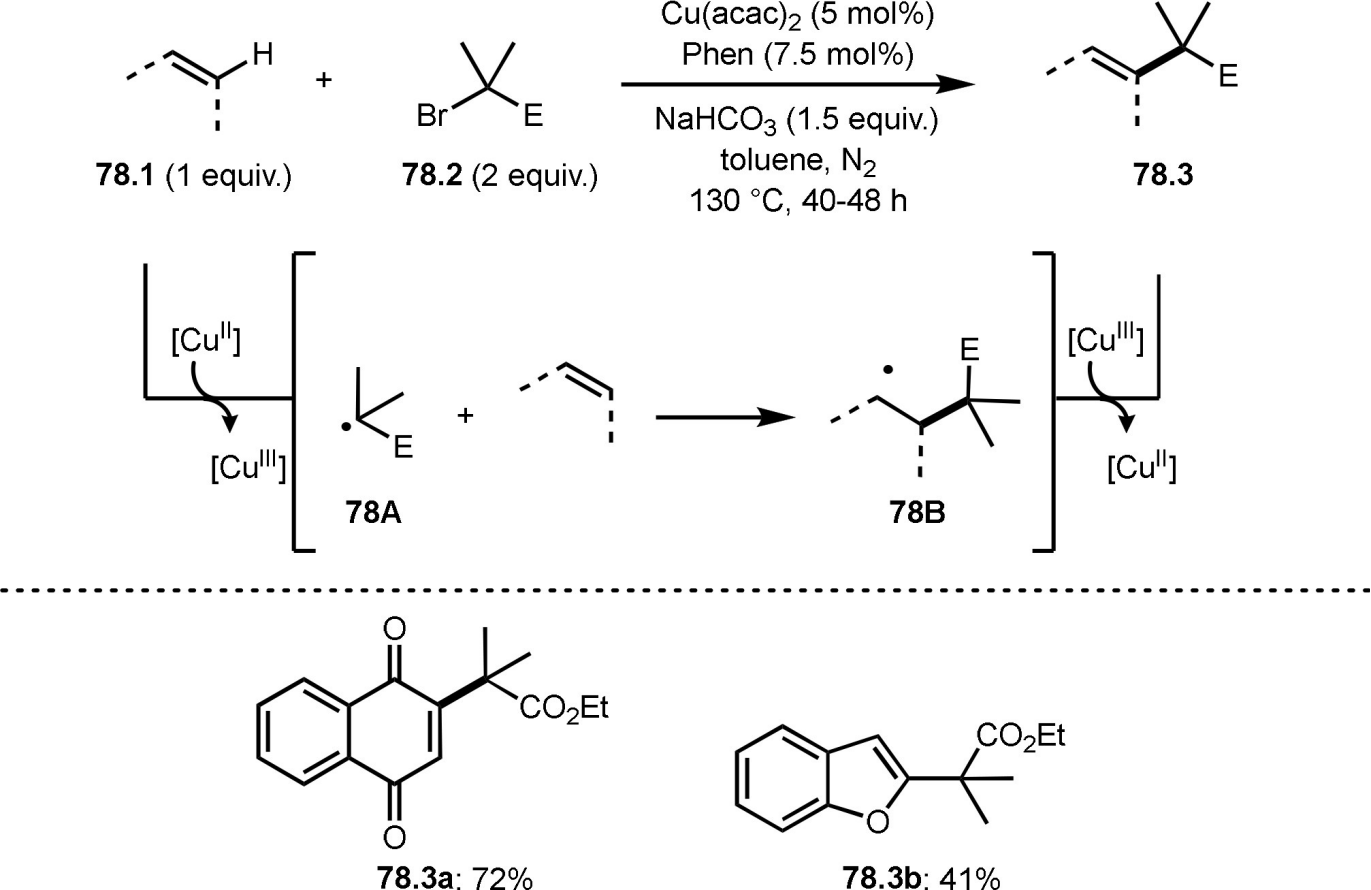
Cu‐catalyzed tert‐alkylation of cyclic olefin

Although electron‐deficient olefins are low reactive towards electron‐deficient α‐tert‐alkyl radicals. Indeed, the reaction of 1,4‐quinone (**77.1**) and α‐bromocarbonyl compounds (**77.2**) gave **77.3** in low yield, which was reported by Li and Shen (Scheme 77).^[214]^ Polarity matching of radical reactions is also very important. On the other hand, Li and Yang reported ATRS or benzofuran C−H functionalizations (Scheme 78).^[215]^ Benzofuran is slightly electron rich, whereas 1,4‐quinone is poor. Unlike the reaction shown in Scheme 76, Cu‐catalyzed conditions were effective to obtain tert‐alkylated 1,4‐quinone (**78.3**) in good yield. It is very difficult to understand the differences between the two reactions.

**Scheme 79:**
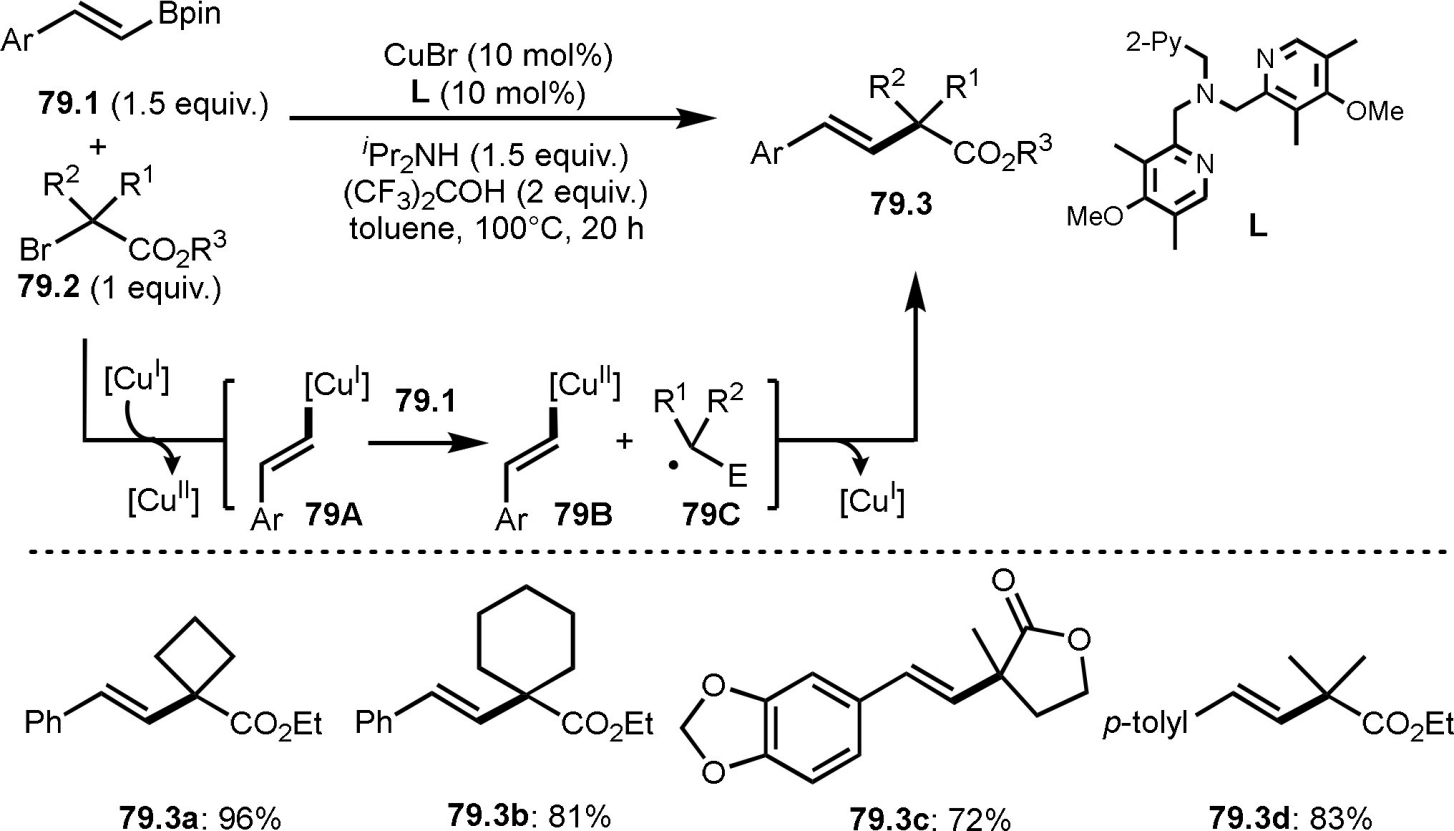
Cu‐catalyzed Suzuki‐Miyaura type coupling.

α‐Bromocarbonyl compounds can be used for cross‐couplings with organoborons or related organometallic reagents. Nishikata's group reported the reaction of 1‐alkenyl boronic esters (**79.1**) and α‐bromocarbonyl compounds (**79.2**) in the presence of a Cu catalyst to produce coupling products (**79.3 a**–**d**) (Scheme 79).^[216]^ Transmetalation between **79.1** and a Cu catalyst occurred to form 1‐alkenyl copper(I) species (**79A**). **79A** reacted with **79.2** to produce **79B** and **79C** via SET.

Finally, the reaction of **79B** and **79C** followed by reductive elimination gave **79.3**. They confirmed that the stoichiometric reaction of **79.1** and Cu salt gave **79A** and isolated **79A** reacted with **79.2** to give **79.3**.

**Scheme 80:**
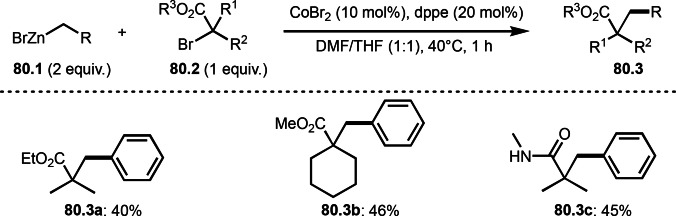
Co‐catalyzed Negishi type coupling.

Alkylzinc reagents (**80.1**) also reacted with α‐bromocarbonyl compounds (**80.2**) in the presence of a Co catalyst to produce coupling products (**80.3 a**–**c**), which was reported by Alcázar's group (Scheme 80).^[217]^ They mainly used benzyl zinc reagents, and simple alkyl zinc reagents were not reactive. The details of the reaction mechanism is not clear but they suggested the presence of Co(0) species in the reaction media.

**Scheme 81:**
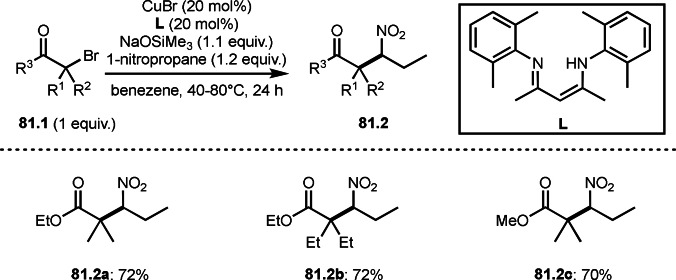
Cu‐catalyzed α‐alkylation of nitroalkane.

α‐Alkylation of nitroalkanes are one of difficult reactions. As with the ketone reactions shown in Scheme 76, it is very difficult to control the reactivity of the enolate equivalents from nitroalkanes, because *O*‐alkylation sometimes predominates in the reaction with an alkyl halide. Watson's group realized this difficult reaction under Cu/L catalyst system (Scheme 81).^[218 219]^ The reaction of NaOSiMe_3_ with 1‐nitropropane produced enolate equivalent,^[220]^ and the α‐tert‐alkyl radical generating from **81.1** immediately reacted with it to finally produce **81.2**. The reactions were *C*‐selective to give **81.2 a**–**c** in good yields with perfect selectivities.

**Scheme 82:**
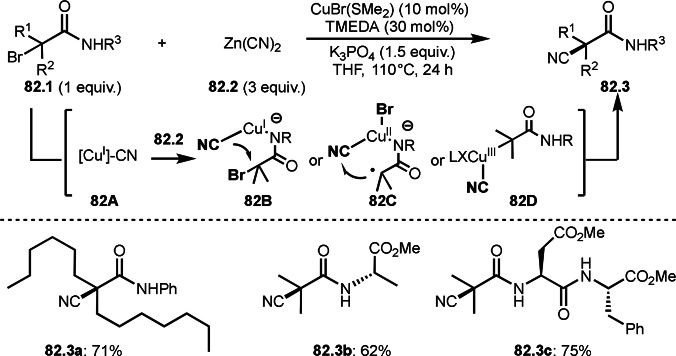
Cu‐catalyzed cyanation.

The cross‐coupling between cyanide and α‐bromocarbonyl compounds was accomplished by Nishikata's group (Scheme 82).^[221a]^ Although Sm‐mediated cyanation was known, the reported reaction is applicable to only one substrate.^[221b]^ α‐Cyanatedcarbonyl compounds are very important synthetic intermediates for β‐aminoacids. The reaction of **82.1** and zinc cyanide (**82.2**) gave the corresponding cyanated products (**82.3 a**–**c**) in moderate to good yields. The reaction is applicable to the synthesis of peptides possessing congested β‐aminoacid moiety. The key intermediate in this reaction is CuCN. Indeed, stoichiometric amounts of CuCN instead of Zn(CN)_2_ (**81.2**) and catalytic CuX underwent smooth cyanation of **82.1**. The reaction mechanism is involving intermediate **82B** (intramolecular substitution), **82C** (addition elimination), or **82D** (reductive elimination). Radical inhibitor tests (TEMPO and BHT) slightly affected the product yields. When the inhibitors were added, the yields were halved. The free‐radical reaction may not be the main reaction pathways.

**Scheme 83:**
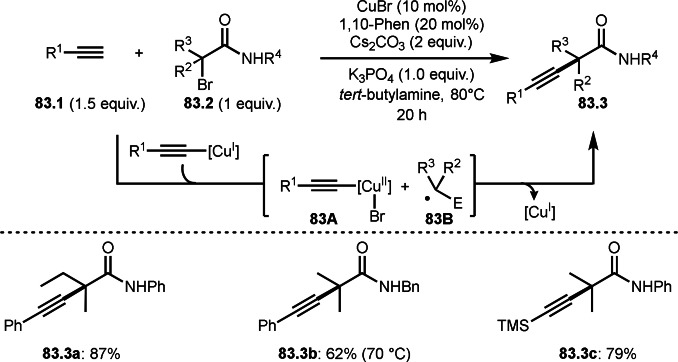
Cu‐catalyzed alkynylation.

Sonogashira coupling is the reaction between a terminal alkyne and an organic halide, and it is one of the most important reactions in alkyne synthesis. The drawback of this reaction is that the main coupling partner of the terminal alkyne is an aryl or a 1‐alkenyl group in the presence of a Pd catalyst. Nishikata's group found that α‐bromocarbonyl compounds (**83.2**) reacted with terminal alkynes (**83.1**) in the presence of a Cu catalyst to produce the corresponding coupling products (**83.3 a**–**c**) in good yields (Scheme 83).[^222^, ^223^] They suggested that in situ generated alkynyl‐Cu^I^ species reacted with **83.2** to produce **83A** and **83B**. Finally, the corresponding α‐tert‐alkynylated carbonyl product (**83.3**) was obtained by reductive elimination of the Cu^III^ complex, presumably obtained following coupling of **83A** and **83B**. They confirmed that an isolated alkynyl‐Cu^I^ complex reacted smoothly with **83.2** to produce **83.3** (Castro‐Stephens coupling).

### 3.7 C−O Coupling Reaction

**Scheme 84:**
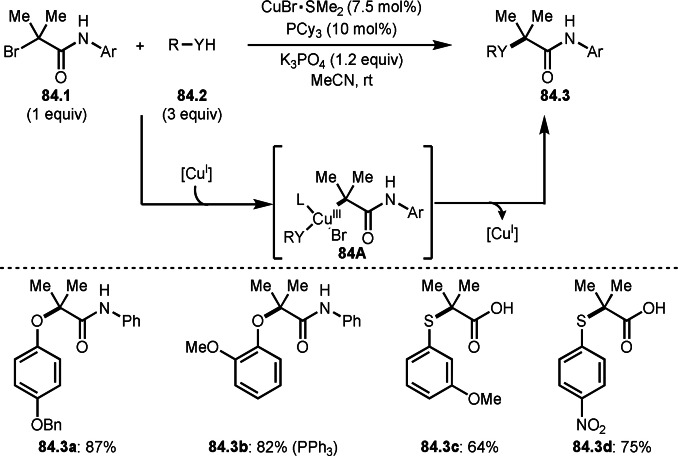
Cu‐catalyzed reactions with *O*‐ or *S*‐nucleophiles.

α‐Bromocarbonyl compounds can react with nucleophiles as well as unsaturated bonds. This means that α‐bromocarbonyl compound formally acts as an electrophile. Kürti's group reported the reaction of α‐bromocarbonyl compounds (**84.1**) and *O*‐ or *S*‐nucleophiles (**84.2**) in the presence of a Cu catalyst (Scheme 84).^[224]^ This reaction may not involve a radical process, based on their control experiments. Generally, a multidentate nitrogen ligand, such as PMDETA, is required for the generation of an α‐*tert*‐alkyl radical from α‐bromocarbonyl compound. In this case, however, no effective ligand to generate radicals was used. A Cu^I^/phosphine complex reacted with **84.1**, followed by ligand exchange with **84.2** to give **84A**. **84.3** was produced via reductive elimination of **84A**. Under their conditions, ethers and thioethers (**84.3 a**–**d**) can be synthesized in good yields.

**Scheme 85:**
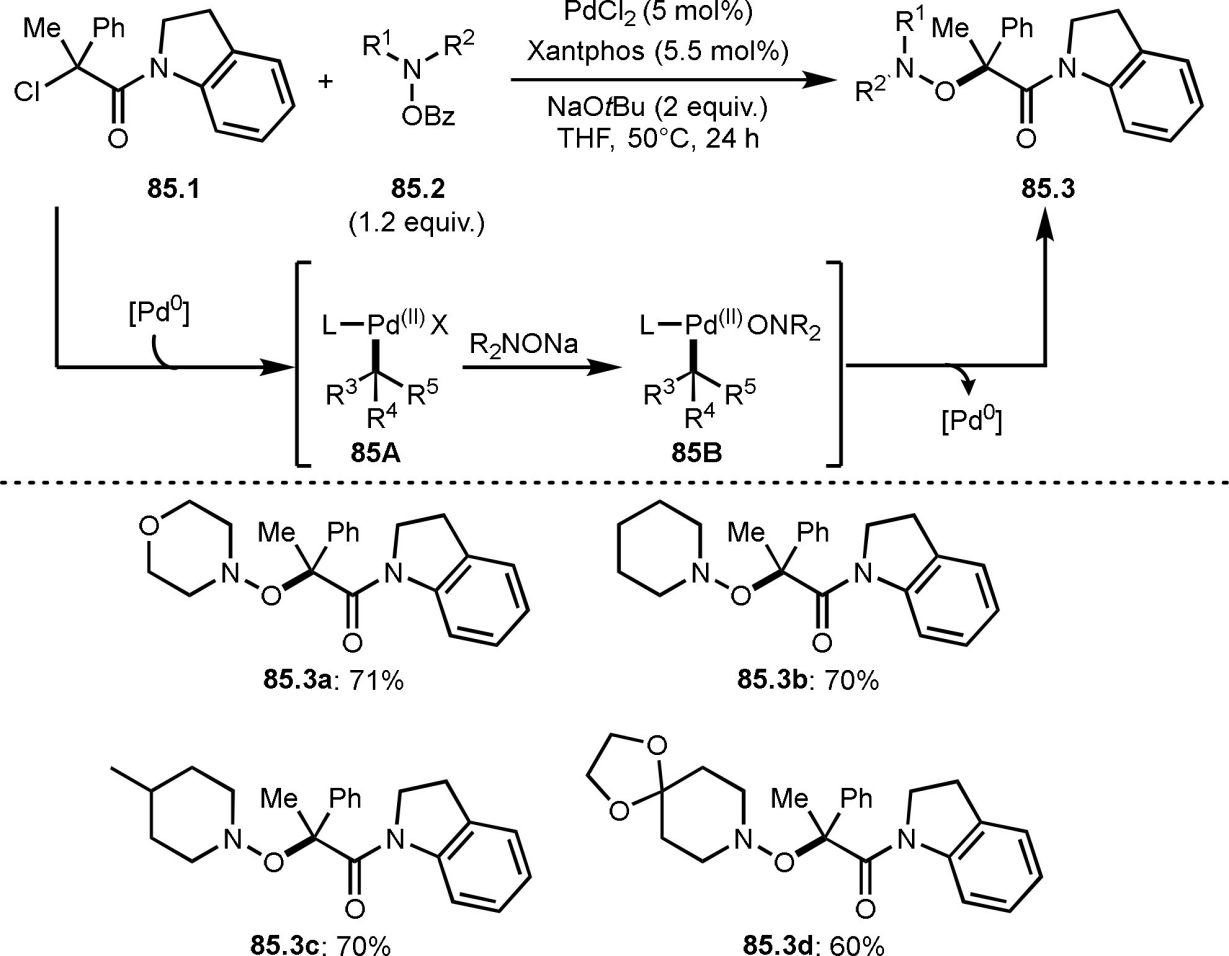
Pd‐catalyzed congested C−O bond formations.

Shao, Xiao, and Deng's group reported the reaction of α‐chlorocarbonyl compounds (**85.1**) and hydroxylamines (**85.2**) in the presence of a Pd catalyst (Scheme 85).^[225a]^ The reaction started with SET between Pd^0^ and **85.1** to give **85A**. This radical reaction process was revealed by TEMPO test, in which alkylated TEMPO was detected. Next, **85A** reacted with R_2_NONa, which is generated from the reaction of **85.2** and a base, to form **85B**. Finally, **85.3** was obtained via reductive elimination of **85B**. Under these reaction conditions, **85.3 a**–**d** were synthesized in good yields. In previous reports, α‐hydroxylation of α‐bromocarbonyl compounds required oxygen to form C−O bonds,^[225b]^ whereas in this case, Pd‐catalyzed cross‐coupling occurred.

**Scheme 86:**
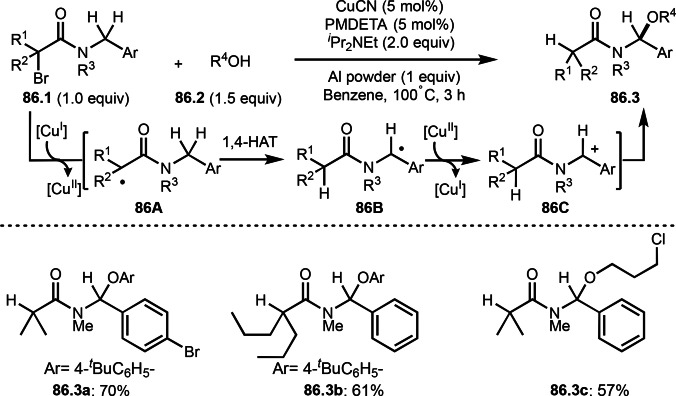
Cu‐catalyzed 1,4‐HAT.

The reaction of an α‐bromocarbonyl compound and a nucleophile occurs at carbonyl α‐position. Nishikata's group reported formally remote nucleophilic substitution via 1,4‐hydorogen atom‐transfer (HAT) process to synthesize ethers possessing *N*‐acyl‐*N*,*O*‐acetal motif (**86.3**) (Scheme 86).^[226]^ The reaction of α‐bromocarboxamides (**86.1**) and alcohols/phenols (**86.2**) in the presence of a copper catalyst. They called this reaction a remote nucleophilic substitution (rSN). The role of Al powder is probably effective to generate active Cu^I^ species during the reaction. In the presence of Al powder, the yields increased by ca. 20 %. The reaction started with the generation of α‐*tert*‐alkyl radical (**86A**) from **86.1**. The resulting **86A** underwent 1,4‐HAT to give **86B**. **86B** was then oxidized with Cu^II^, and the resulting cation reacted with **86.2** to produce **86.3**. Both alcohols and phenols can be used for the reaction to give **86.3 a**–**d** in good yields.

### 3.8 C−N Coupling Reaction

Alkylamines can be synthesized by nucleophilic substitution via SN_2_ or SN_1_ reactions. Transition‐metal‐catalyzed reactions are one of the options for synthesizing alkylamines. However, these are unsuitable for the synthesis of functionalized and congested amines possessing a four‐substituted carbon due to slow oxidative addition and undesired β‐hydride elimination. The amination reaction of α‐bromocarbonyl compounds is one of the options to synthesize *tert*‐alkyl amines, because they are also a good electrophile.

**Scheme 87:**
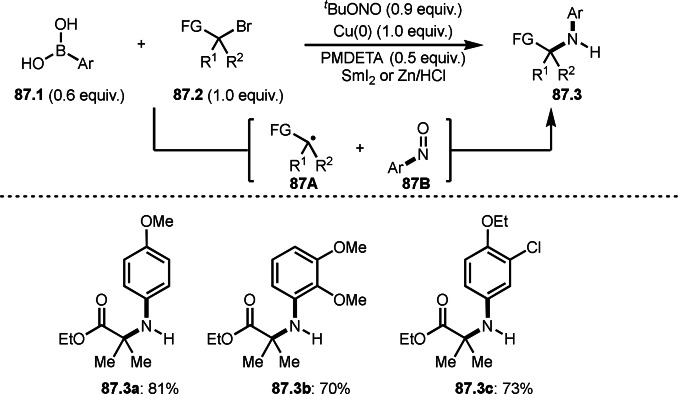
Cu‐mediated double amination.

Read de Alaniz's group accomplished *tert*‐alkylation reactions of *tert*‐butyl nitrite (Scheme 87).^[227]^ The reaction of arylboronic acids (**87.1**), α‐bromocarbonyl compounds (**87.2**) and *tert*‐butyl nitrite in the presence of a Cu salt gave the corresponding *tert*‐alkylated amines (**87.3 a**–**d**). This reaction is a kind of double amination reaction. The first amination entailed reaction between **87.1** and tert‐butyl nitrite to give **87B**.[^228^, ^229^] The second amination was the reaction of α‐*tert*‐alkyl radical (**87A**) and **87B** to produce **87.3** upon *N−O* bond cleavage with SmI_2_. Although the reaction was not catalytic, two C−N bonds can be formed in a single protocol.

**Scheme 88:**
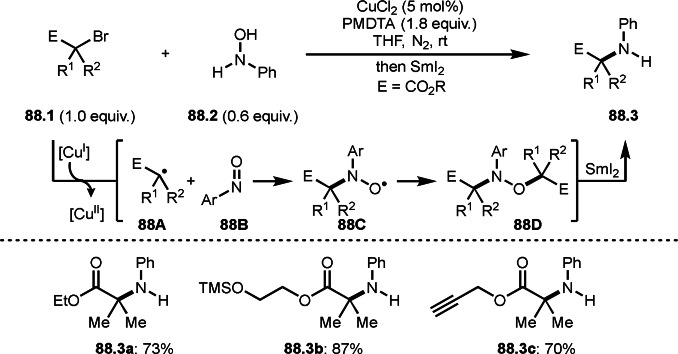
Cu‐catalyzed amination with a nitroso compound.

Read de Alaniz's group also accomplished single amination reaction with α‐bromocarbonyl compounds (**88.1**) in the presence of a Cu catalyst and hydroxylamine (**88.2**) to produce the corresponding *tert*‐alkylated amines (**88.3 a**–**d**) (Scheme 88).^[230]^ In their previous work shown in Scheme 87, aryl nitroso compounds were generated from the reaction of arylboronic acids and nitrite, in situ generated aryl nitroso compounds (**88B**) from the Cu^II^ oxidation of **88.2** were used. The resulting **88B** reacted with **88A** to form **88C**. The *O*‐radical intermediate **88C** was then captured by **88A** to give **88D**. Finally, **88.3** was produced by the reduction of **88D** with SmI_2_. Functional group compatibility of this reaction is good. For example, reaction with **88.1**, which has a terminal alkyne fragment that is a good α‐*tert*‐alkyl radical acceptor, gave the corresponding amination product **88.3** without loss of the C−C triple bond.

**Scheme 89:**
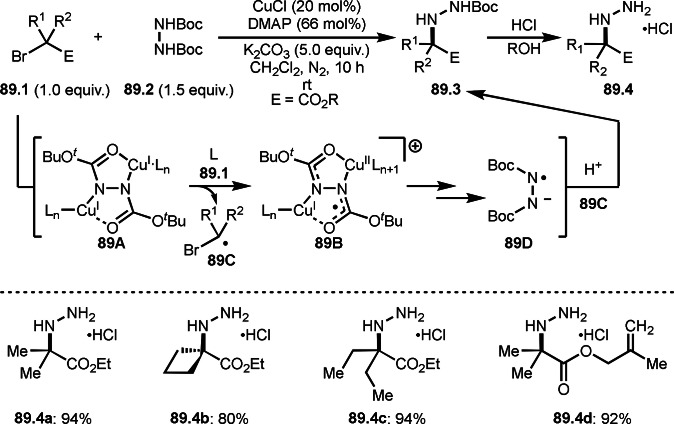
Cu‐catalyzed amination with hydrazine.

Hydrazine is also a good amination reagent. Lu and Gong'group reported Cu‐catalyzed amination of α‐bromocarbonyl compounds (**89.1**) hydrazines (**89.2**) to produce *tert*‐alkylated hydrazines (**89.3**) (Scheme 89).^[231]^ The reaction first generated an active Cu^I^ catalyst (**89A**) which was detected by HRMS analysis. SET from **89A** to **89.1** to give **89B** and **89C**. An anion radical **89D** was produced from ligand‐to‐metal charge transfer/dissociation of *N*‐radical. The resulting **89D** then coupled with **89C** to produce **89.3**, which was stabilized as HCl salt (**89.4**).

**Scheme 90:**
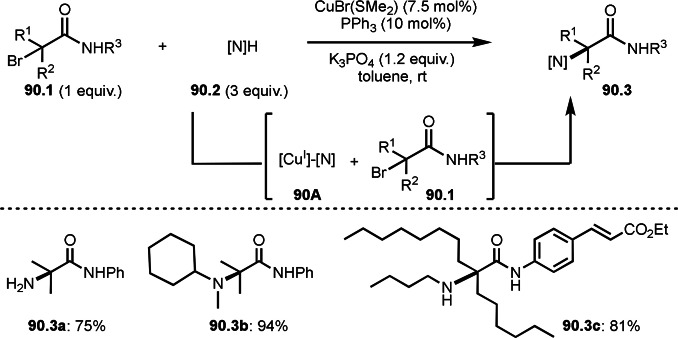
Amination under Cu/PPh_3_ catalyst system.

Nishikata's group found that a copper/phosphine catalyst system at room temperature was suitable for the reaction of amines (**90.2**), including ammonia and primary and secondary alkylamines, and amides with α‐bromocarbonyl compounds (**90.1**) to construct congested C(sp^3^)–N bonds at room temperature (Scheme 90).^[232]^ The reaction was not affected by the presence of TEMPO or BHT, nor was the yield of **90.3**. This result suggested that a free‐radical process was not the major pathway in this catalytic cycle. Moreover, a mechanistic study revealed that copper amide is a key intermediate. The reaction of a Cu salt and **90.2** produced **90A**. Isolated **90A** smoothly reacted with **90.1** to give the corresponding α‐aminated product **(90.3**). Under these conditions, highly congested amines or aminated products with ammonia were easily prepared at room temperature.

### 3.9 C−H Functionalization

An aromatic C−H bond is one of the most attractive substrates for *tert*‐alkylation reaction with α‐bromocarbonyl compounds. Generally, Friedel‐Crafts reaction is the only way to carry out tert‐alkylations of arenes via a cationic process. But this process can employ simple *tert*‐alkyl groups under strictly anhydrous conditions to use a cation intermediate. On the other hand, *tert*‐alkylation reactions with arenes using α‐bromocarbonyl compounds can employ various functional groups. The reaction conditions are generally not sensitive for oxygen or moisture.

**Scheme 91:**
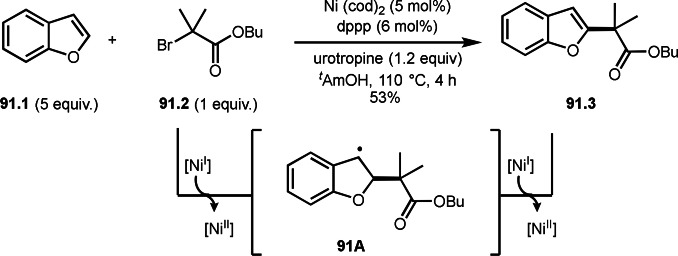
Ni‐catalyzed furan C−H bond *tert*‐alkylation.

Slightly electron poor α‐*tert*‐alkyl radicals from α‐bromocarbonyl compounds smoothly react with electron‐rich arenes. For example, furans are suitable substrates for this purpose. Hirano and Miura's group, Nishikata's group, and Evano's group reported Ni, Fe, and Cu‐catalyzed α‐*tert*‐alkylation reactions with furans, independently (Scheme 91,^[233]^ 92,^[234]^ and 93, ^[235]^). Ni catalyst system required bisphosphine as a ligand, Iron system did not need any ligands, and Cu system was a multidentate nitrogen ligand, TPMA to produce α‐*tert*‐alkyl radicals from α‐bromocarbonyl compounds. All reactions passed through the same intermediates (**91A**, **92A**, and **93A**).

**Scheme 92:**
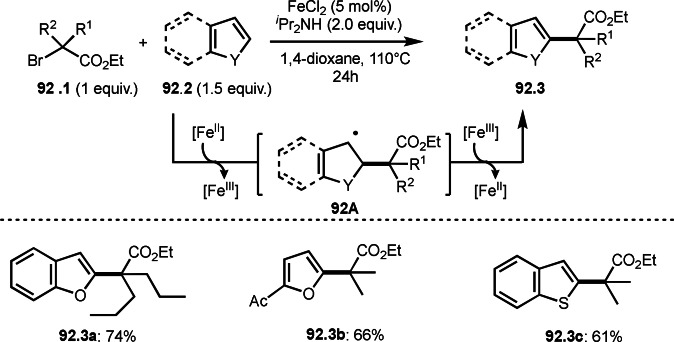
Fe‐catalyzed furan C−H bond *tert*‐alkylation.

**Scheme 93:**
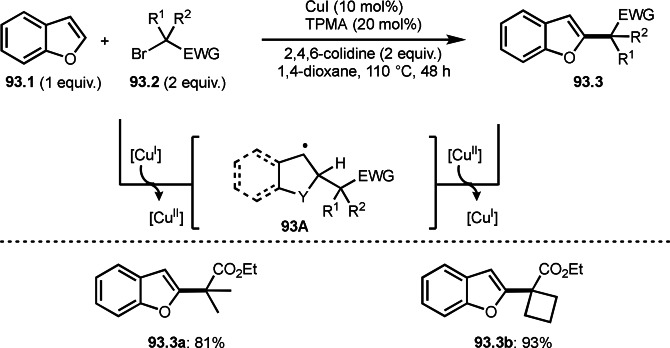
Cucatalyzed furan C−H bond *tert*‐alkylation.

**Scheme 94:**
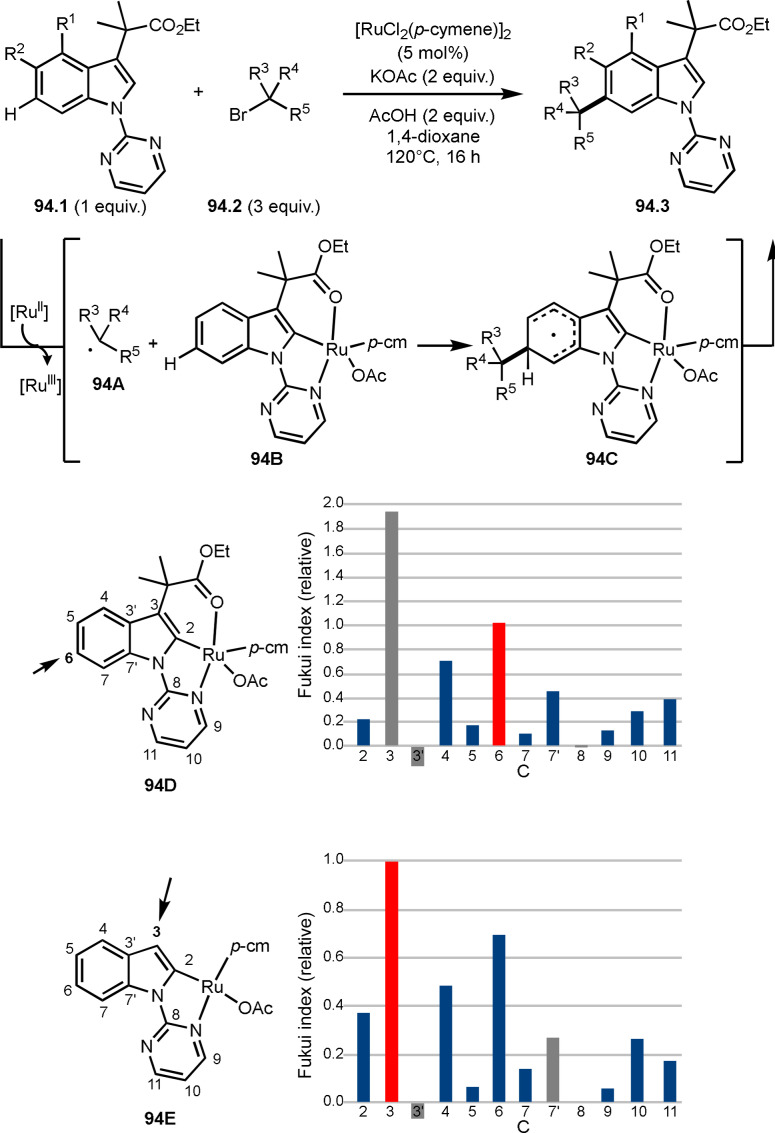
Ru‐catalyzed remote C6‐selective C−H tert‐alkylation.

α‐*tert*‐Alkyl radicals from α‐bromocarbonyl compounds reacted with electron‐rich C−H bonds in the reaction with arenes. Frost's group developed C2‐cyclometalation/remote σ‐activation of **94.1** in the presence of a Ru catalyst (Scheme 94).^[236]^ The reaction of Ru^II^ salt with **94.2** generated an α‐*tert*‐alkyl radical from α‐bromocarbonyl compound (**94.2**). Ru also acted as a blocking group. A Ru complex underwent C−H ruthenation with **94.1** to produce a ruthenacycle intermediate (**94B**). tert‐Alkylation of **94B** occurred at C6‐position to give **94C**. Desired product (**94.3**) was produced from re‐aromatization of **94C**. According to the Fukui indices, which are a simple method to predict selectivity in aromatic radical reactions, C6‐position was the most reactive of all C position of **94D**. On the other hand, **94E** without functional group at C3 was C3. Indeed, the reaction of **94E** underwent C−H radical alkylation at C3. This remote C−H alkylation is convenient to control the regioselectivities in the reaction of arenes and α‐bromocarbonyl compounds.

**Scheme 95:**
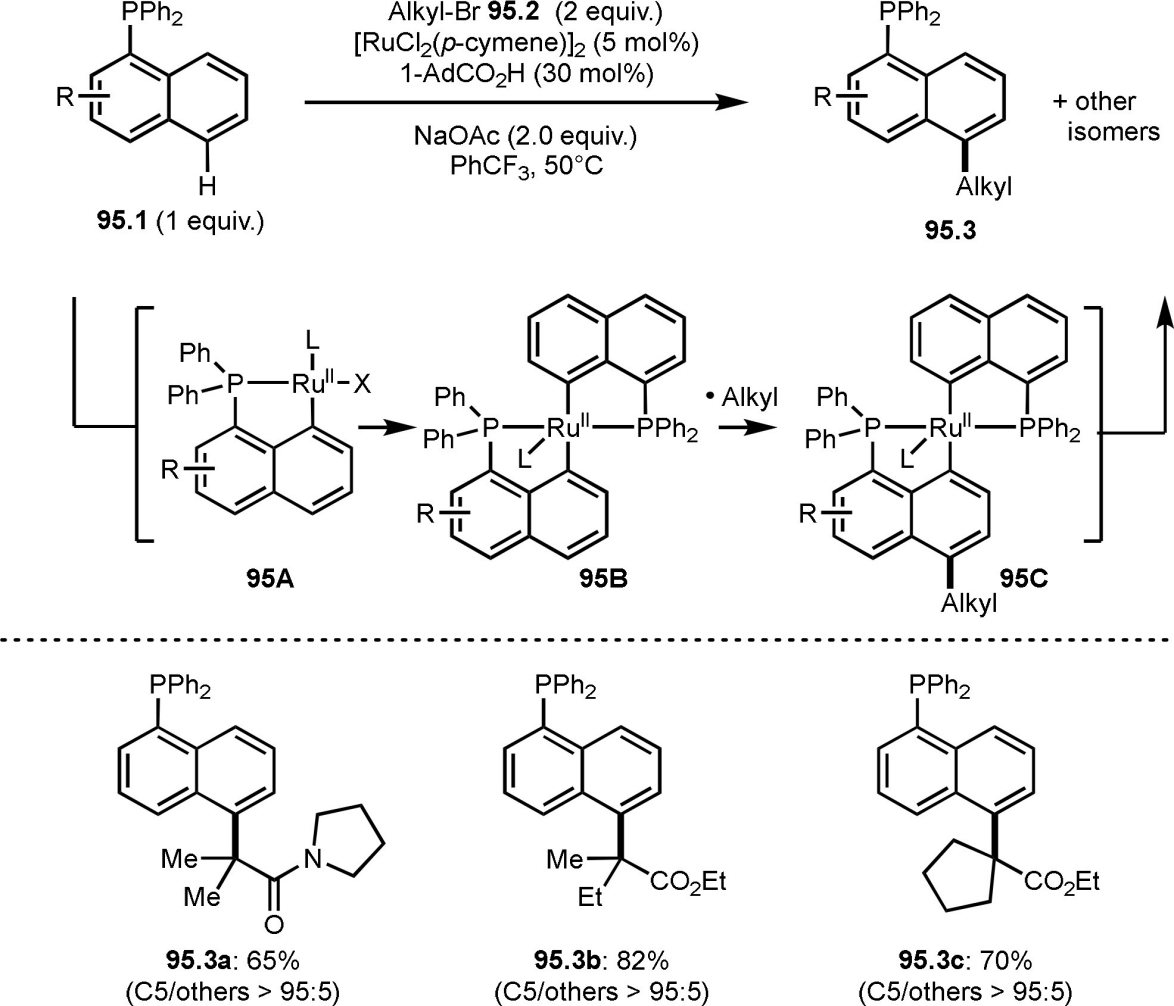
Ru‐catalyzed naphthalene C5 *tert*‐alkylation.

Liu and Zheng's group reported C5‐selective *tert*‐alkylation of phosphines in the presence of a Ru catalyst (Scheme 95).^[237]^ Naphthalene‐substituted phosphines (**95.1**) reacted with Ru catalyst to produce ruthenacycle intermediates (**95A**, **95B**), which were detected by HRMS analysis. α‐*tert*‐alkyl radical from α‐bromocarbonyl compounds (**95.2**) reacted with C5 position of naphthalene ring to give **95C**, which produced **95.3**. In this case, *para*‐position with respect to Ru was activated for radical reaction. There were some regioisomers, but the selectivities were >95 : 5.

**Scheme 96:**
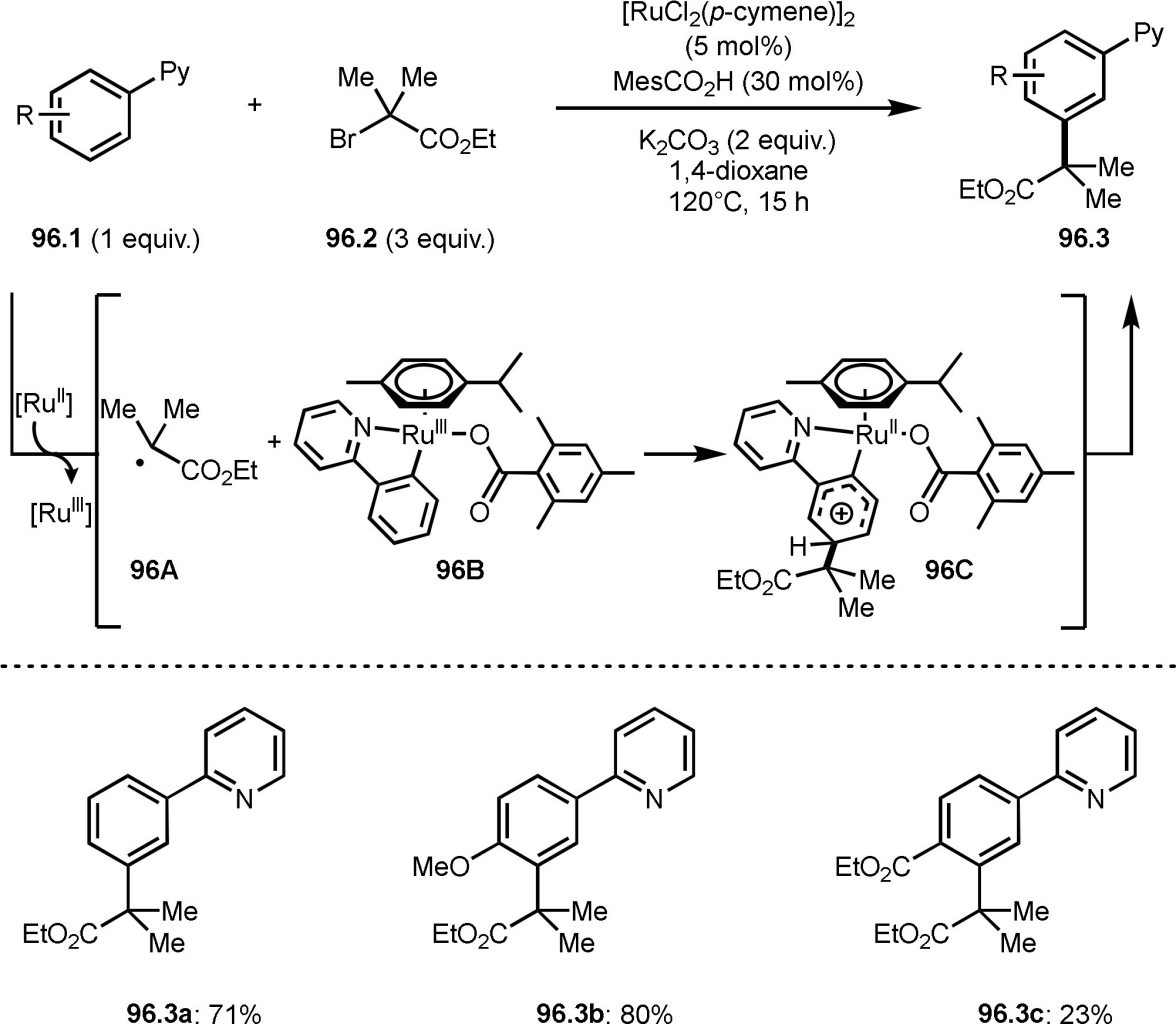
Frost's Ru‐catalyzed *meta*‐*tert*‐alkylation.

**Scheme 97:**
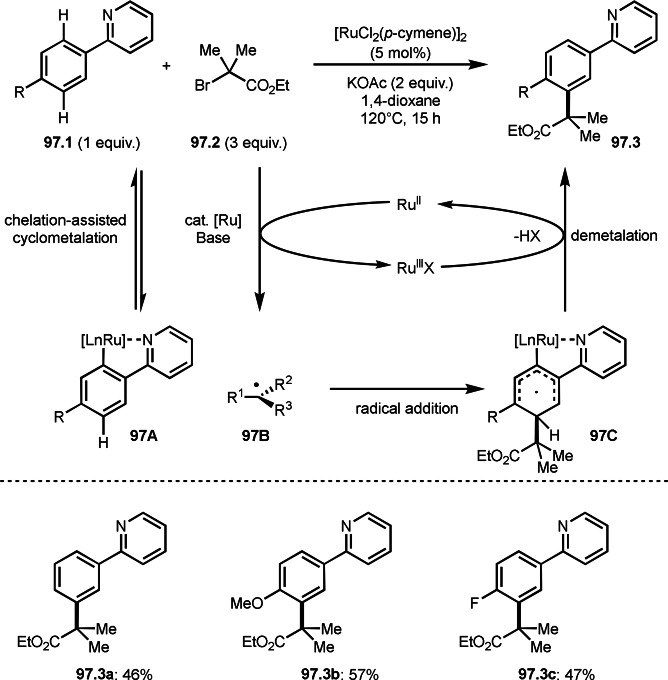
Ackermann's Ru‐catalyzed *meta*‐*tert*‐alkylation.

Frost's group^[238]^ and Ackermann's group^[239]^ reported *meta*‐*tert*‐alkylations of aromatic rings (Scheme 96 and 97). They employed 2‐pyridyl group as a directing group. Both reactions employed C−H ruthenation process to block the ortho‐position of the substrates (**96.1** and **97.1**), and *para*‐position with respect to Ru was activated for *tert*‐radical (**96A** and **97B**) generated from the reaction of Ru^II^ and α‐bromocarbonyl compounds (**96.2** and **97.2**). The resulting *tert*‐alkylated ruthenacycles (**96C** and **97C**) produced *meta*‐tert‐alkylated products (**96.3** and **97.3**) with perfect selectivities. Other Ru‐catalyzed *meta*‐selective C−H tert‐alkylations of arenes possessing various directing groups were also reported.[^240^, ^241^, ^242^, ^243^, ^244^, ^245^, ^246^, ^247^]

**Scheme 98:**
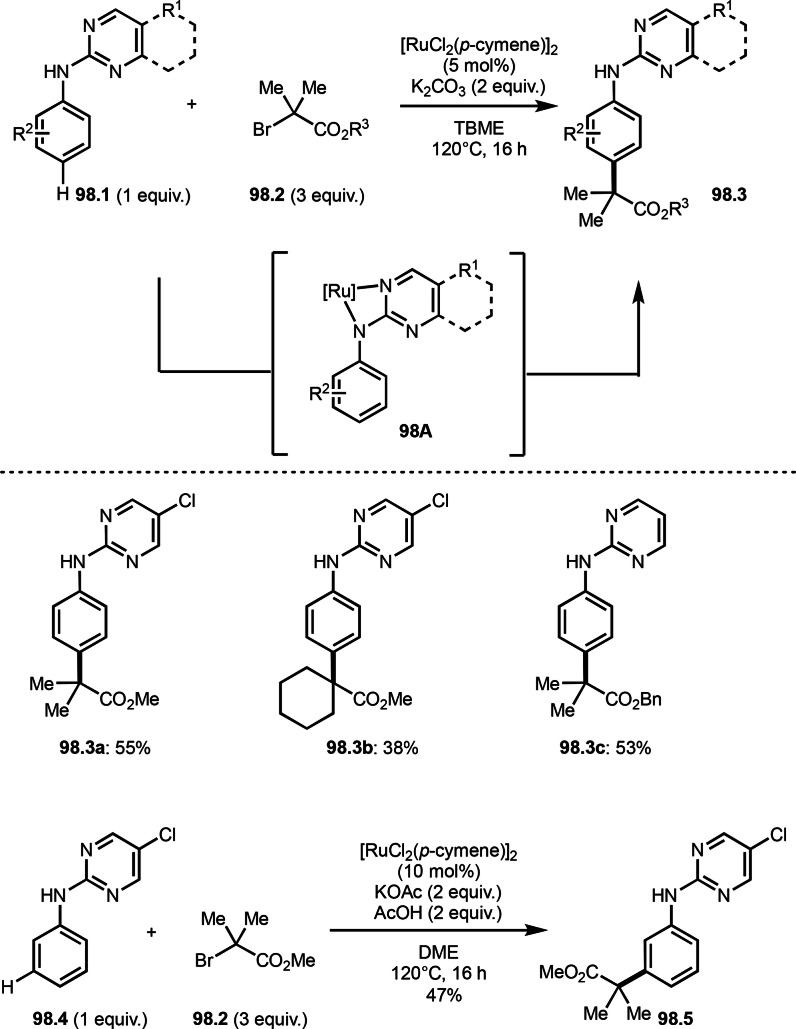
Ru‐catalyzed *para*‐*tert*‐alkylation.

Frost's group also reported Ru‐catalyzed *para*‐*tert*‐alkylations of aniline derivatives (**98.1**) with α‐bromocarbonyl compounds (**98.2**) to produce *para*‐*tert*‐alkylated products (**98.3**) (Scheme 98).^[248a]^ Unlike *meta*‐selective *tert*‐alkylations shown above, N−H cyclometalated complex (**98A**) was generated as a key intermediate, which accomplished *para*‐selectivity. This is probably due to the high electron density in the para‐position of **98A**, which makes it easier to react with the electron‐poor α‐*tert*‐radical. An interesting aspect of this reaction is the switch in selectivity from *para* to *meta*. When acetate was used instead of K_2_CO_3_, *meta*‐*tert*‐alkylated product (**98.5**) was obtained in 47 % yield with perfect *meta* selectivity. They described that *this outcome suggests that in a proposed equilibrium between N−H and C−H cyclometalated complexes, the use of a carbonate base (K_2_CO_3_) could favor an N−H cyclometalation to form para‐substituted products, whereas acetate bases (KOAc) could favor an ortho‐C−H cyclometalation to form meta‐substituted products*. Other *para*‐selective *tert*‐alkylations with α‐bromocarbonyl compounds employed acetoamido or 2‐pyrimmidinylamine as a directing group via similar mechanism shown in Scheme 94.[^249^, ^250^, ^251^]

**Scheme 99:**
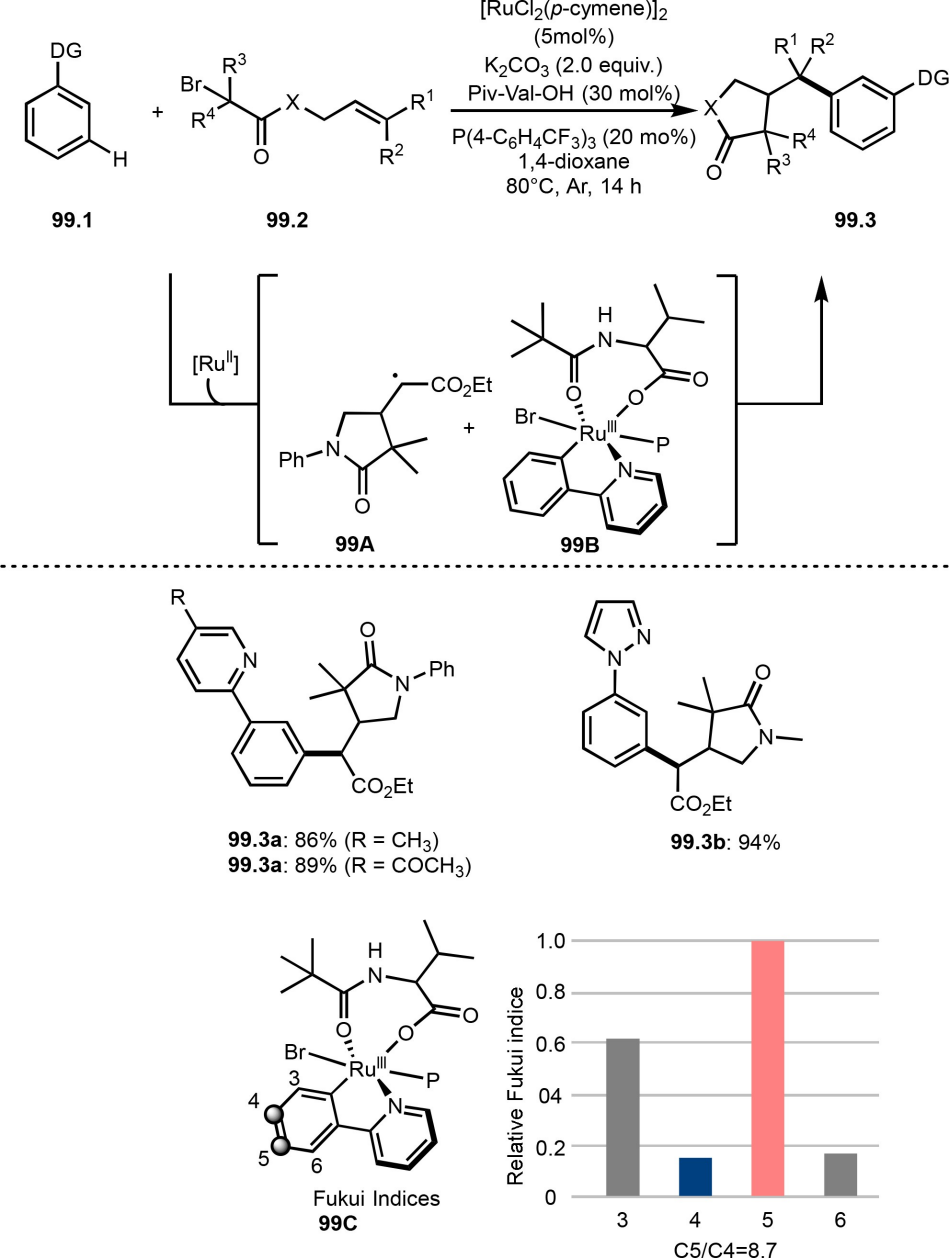
Ru‐catalyzed tandem cyclization/*meta*‐C−H alkylation.

Liang's group reported tandem cyclization/*meta*‐C−H bond alkylations to produce bicyclic products (**99.3**) (Scheme 99).^[252]^ Initial process in this reaction was radical cyclization of **99.1** to give **99A**. Next, *meta*‐C−H tert‐alkylation occurred with **99B** to produce **99.3**. Pyridine and pyrazole were acted as a good directing group in this reaction. As was demonstrated by Frost shown in Scheme 94, they explained *meta*‐selectivity by Fukui indices of ruthenacycle complex (**99C**).

**Scheme 100:**
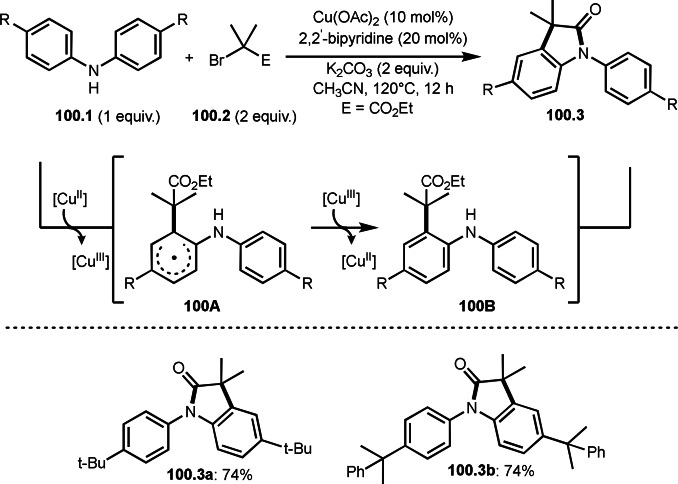
Cu‐catalyzed tandem *ortho*‐C−H *tert*‐alkylation/intramolecular amidation.

Song, Xie, and Li's group reported tandem *ortho*‐C−H *tert*‐alkylation/intramolecular amidation of diarylamine (**100.1**) and α‐bromocarbonyl compounds (**100.2**) to produce cyclic products (**100.3 a** and **b**) (Scheme 100).^[253]^ The reaction of Cu^II^ and **100.2** gave α‐*tert*‐alkyl radical, which reacted with **100.1** to form **100A**. Generally, Cu^I^ generates α‐*tert*‐alkyl radical from α‐bromocarbonyl compound via SET, but they employed Cu^II^ as an α‐*tert*‐alkyl radical generator. Next, CuII oxidation of 100A followed by re‐aromatization to produce **100B**. Final desired cyclic product (**100.3**) was obtained via an intramolecular amidation. α‐*tert*‐Alkyl radical is generally not reactive to arenes without activation like ruthenacycles shown above. But their reaction smoothly underwent α‐*tert*‐alkylations. They also suspected this process and carried out this reaction with *N*‐α‐bromoamidated **100.1**, which is generated from amidation reaction of **100.1** and **100.2**. But no intramolecular radical cyclization to give **100.3** occurred. Probably high temperature made this reaction easy.

### 3.10 Other Coupling Reaction

**Scheme 101:**
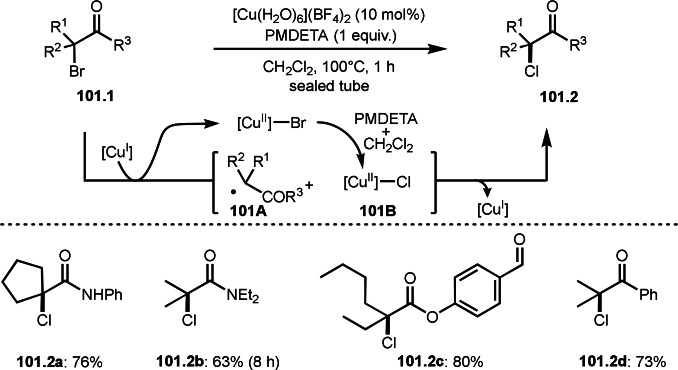
Cu‐catalyzed Br/Cl exchange reaction with α‐bromocarbonyl compound.

Halogen atom exchange reactions, such as the Finkelstein reaction, are one of the traditional methodologies for synthesizing primary and secondary alkyl halogens via nucleophilic substitution. For example, KI is added to increase the reactivities of alkyl chlorides or bromides in nucleophilic substitution reactions, in which C−Cl or C−Br is substituted by iodine anion. Nishikata's group found that the reaction of CH_2_Cl_2_ as a chlorine source with an α‐bromocarbonyl compounds (**101.1**) produced Br/Cl exchange products (**101.2 a**–**d**) in good yields (Scheme 101).^[254]^ In this reaction, CH_2_Cl_2_ acted as a good chlorinating reagent to form 3° Csp^3^−Cl in the presence of a copper catalyst. CH_2_Cl_2_ reacted with PMDETA to produce the corresponding ammonium chloride. On the other hand, Cu^I^ reacted with **101.1** to give **101A** and Cu^II^−Br, and CuCl_2_ (**101B**) was generated from the reaction of the resulting Cu^II^−Br with chlorine anion. Finally, Chlorine atom on **101B** was captured by **101A** to produce *tert*‐alkyl chloride (**101.2**).

**Scheme 102:**
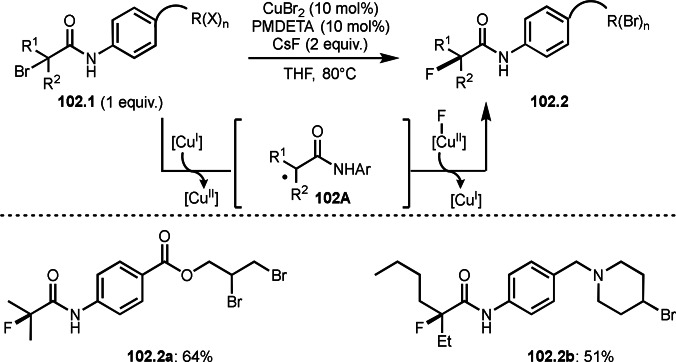
Cu‐catalyzed Br/F exchange reaction with α‐bromocarbonyl compound.

Nishikata's group also discovered Cu‐catalyzed Br/F exchange reaction with α‐bromocarbonyl compound (**102.1**) (Scheme 102).^[255]^ Traditionally, nucleophilic Br/F exchange reaction is sensitive to steric hinderance of alkyl halide. Indeed, they confirmed that tertiary alkyl‐Br was the slowest reaction compared with primary and secondary alkyl‐Br. On the other hand, tertiary‐alkyl bromide gave the fastest reaction in their fluorination. For example, in the alkyl‐Br substituted α‐bromocarbonyl compound (**102.1**), fluorination occurred only at the tertiary carbon (**102.2 a** and **b**). Initially, α‐*tert*‐alkyl radical was generated from the reaction of Cu^I^ and α‐bromocarbonyl compound. **102A** then reacted with [Cu]−F to produce fluorinated product (**102.2**). The reaction in the presence of BHT did not occur, and the reaction with excess CuF_2_/PMDETA reacted with **102.1** to provide fluorinated product (**102.2**).

## 4 Photoreaction Catalyzed by a Transition Metal Salt

Transition metal complexes have diverse redox potentials, whereas combinations of photoirradiation and a transition metal generate active metal species. This phenomenon enables difficult C**−**X bond cleavage via SET to produce carbon radicals, and unprecedented C**−**C bond formations in the reaction with α‐bromocarbonyl compound. This section introduces the reaction including additions, cyclizations, and couplings of α‐tert‐alkyl radicals under photoirradiation.

### 4.1 Addition Reaction

**Scheme 103:**
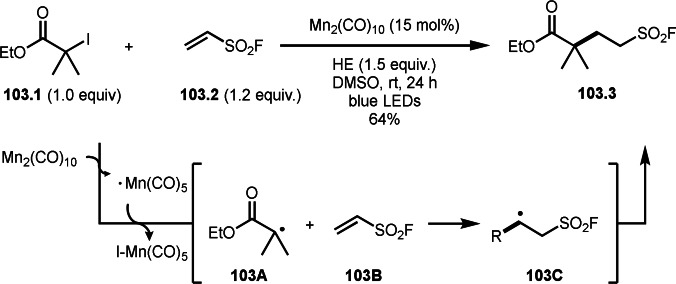
Mn‐catalyzed addition with α‐iodocarbonyl compound.

Alkyl sulfonyl fluorides are one of the most important compounds and can be seen in useful drugs. Therefore, the development of a new methodology to synthesize this skeleton would be valuable. Qin's group found that the reductive addition of α‐iodocarbonyl compound (**103.1**) to ethenesulfonyl fluoride (**103.2**) under visible light irradiation in the presence of Mn catalyst and Hantzsch ester (HE) as hydrogen source gave *tert*‐alkylated sulfonyl fluoride (**103.3**) (Scheme 103).^[256]^ Under the irradiation, the Mn complex decomposed to form active metaloradical species, which reacted with **103.1** to give α‐*tert*‐alkyl radicals (**103A**). **103A** added to **103.2** to form **103C**. Finally, **103C** was captured by hydrogen atom of HE to produce **103.3**. This Giese type addition is convenient to synthesize *tert*‐alkylated alkane and there few report on this tpic.^[257]^

**Scheme 104:**
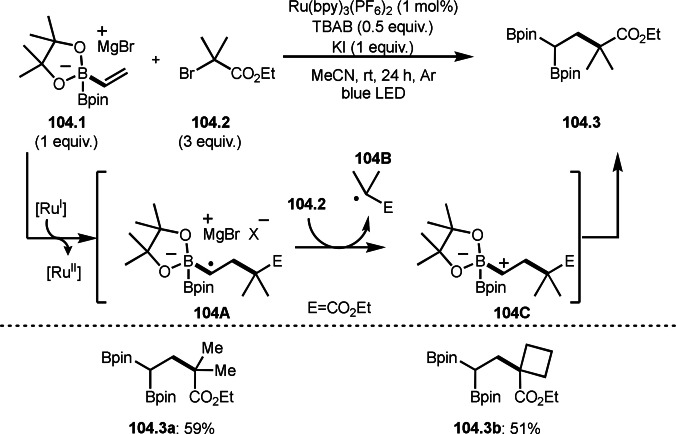
Ru‐catalyzed addition/rearrangement reaction.

In situ formed vinyl diboronate complexes are also a good radical acceptor. Shi's group reported addition/rearrangement reaction of vinyl diboronates (**104.1**) and α‐bromocarbonyl compounds (**104.2**) in the presence of a Ru photocatalyst to give diborylated compounds possessing quaternary carbon center (**104.3**) (Scheme 104).^[258]^ In this reaction, SET from a sacrificial amount of **104.1** (E_p/2_=+0.21 V vs. SCE) to the excited Ru ^II^ species (E_Ru_^II^*_/Ru_^I^=+0.77 V) to give active Ru^I^ species. The resulting Ru^I^ (E_Ru_^I/II^=−1.33 V) species undergoes SET with **104.2** (E_p/2_=ca. −0.8 V) to form α‐*tert*‐alkyl radical species (**104B**) and regeneration of the Ru^II^ catalyst. Photoexcitation of the Ru complex was necessary to produce the active Ru^I^ species. **104B** then added to **104.1** to give **104A**, which was oxidized by **104.2** to give a cation species (**104C**). Finally, 1,2‐boron migration occurred to produce **104.3**.

**Scheme 105:**
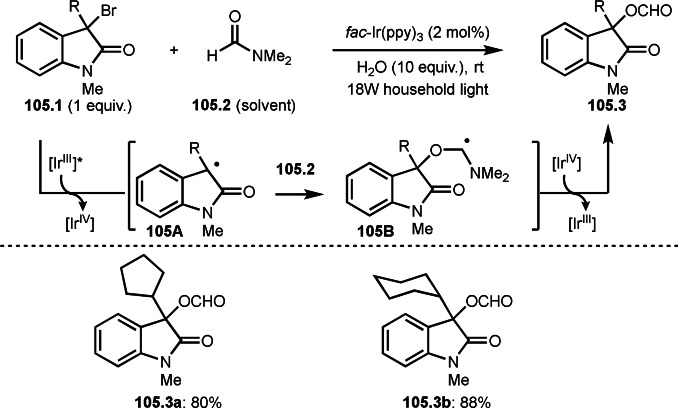
Ir‐catalyzed formyloxylation.

Photocatalyst system of a metal complex is effective to carry out C−O coupling reaction via addition reaction. Chen and Xiao's group reported the reaction of cyclic α‐bromocarbonyl compounds (**105.1**) with *N*,*N*‐dimethylformamide (DMF, **105.2**) as a solvent in the presence of an Ir photocatalyst to give formyloxylated products (**105.3**) (Scheme 105).^[259]^ Initially, a photo‐excited Ir complex reacted with **105.1** to give cyclic α‐*tert*‐alkyl radicals (**105A**). **105A** reacted with the oxygen moiety of **105.2** to produce **105B**. Finally, Ir^IV^ oxidation of **105B** followed by hydrolysis gave **105.3**.

**Scheme 106:**
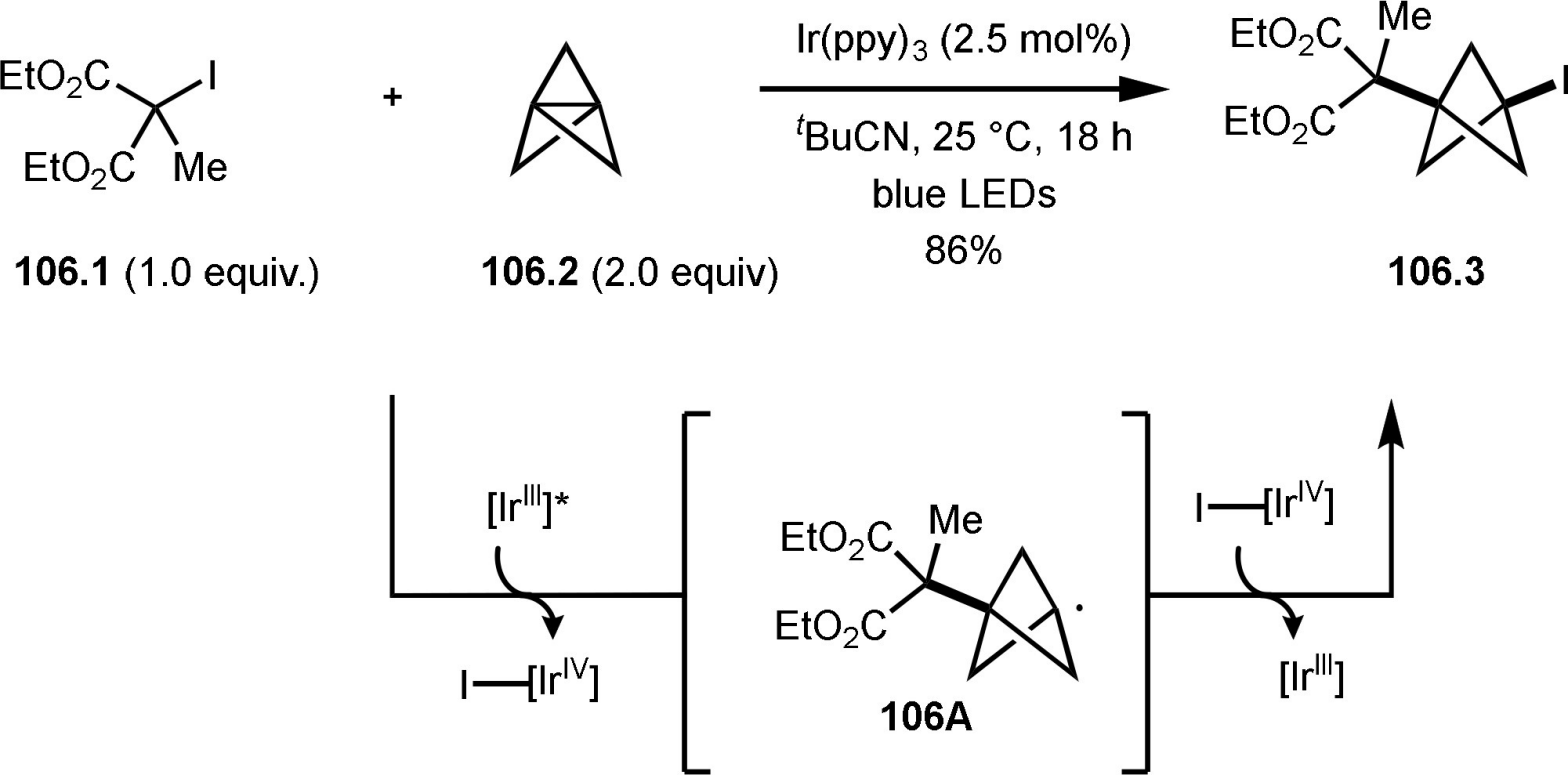
Ir‐catalyzed ATRA with [1.1.1]‐propellane.

In previous section, a transition metal‐catalyzed radical addition of [1.1.1]‐propellane to give bicyclo[1.1.1]pentanes (BCPs) was introduced. Anderson's group reported the ATRA reaction of [1.1.1]‐propellane (**106.2**) with bromomalonate ester (**106.1**) in the presence of an Ir photocatalyst, where a carbon−carbon σ‐bond is functionalized by photocatalyst‐generated radicals (Scheme 106).^[260]^ The reaction started with the formation of α‐*tert*‐alkyl radicals. The resulting radical added to **106.2** to give **106A**. Finally, **106.3** was produced by the reaction of **106A** with I‐[Ir^IV^].

### 4.2 Three‐Component Reaction

Transition metal catalyzed systems under photoirradiation are suitable for three‐component reactions. Although it is difficult to control reactivity when there are many chemical species in a reaction system, complex reactions can be achieved by selecting appropriate catalysts. However, α‐*tert*‐alkyl radicals derived from α‐bromocarbonyl compounds are not highly reactive toward substrates, so careful selection of reaction substrates is necessary to ensure smooth three‐component reactions.

**Scheme 107:**
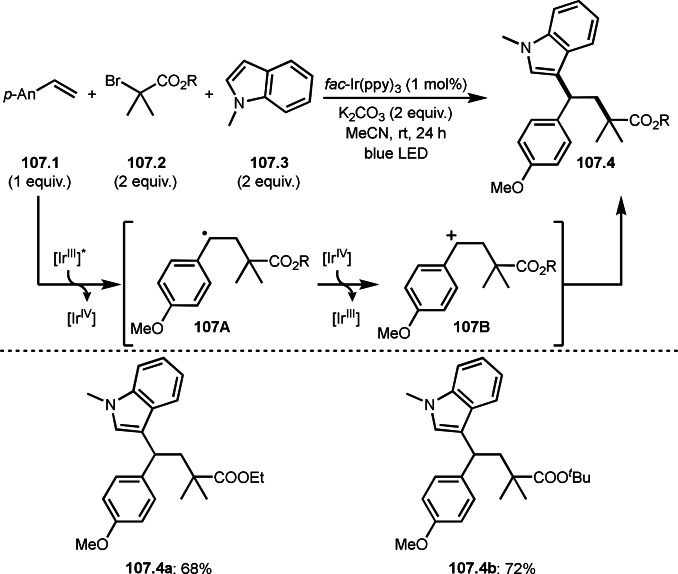
Ir‐catalyzed three‐component reaction using indoles.

Song and Li's group reported the reaction of styrene derivatives (**107.1**), α‐bromocarbonyl compounds (**107.2**), and indoles (**107.3**) in the presence of an Ir‐photocatalyst under visible light irradiation (Scheme 107).^[261]^ The reaction provided 1,2‐alkylarylation products (**107.4 a** and **b**) in moderate yields. The generation of α‐*tert*‐alkyl radicals derived from the reaction of **107.2** and an excited Ir^III^ complex took place in the first step. The resulting α‐*tert*‐alkyl radical added to **107.1** to give **107A**, which was oxidized by the Ir^IV^ complex to form a cation species (**107B**). Finally, a cation **107B** was captured by **107.3** to produce **107.4**. In previous session, α‐*tert*‐alkyl radicals can react with olefins and indoles.

**Scheme 108:**
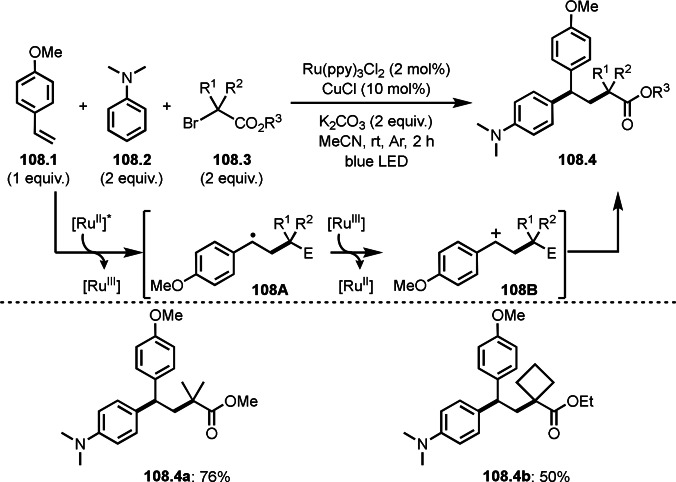
Ru‐catalyzed three‐component reaction using aniline derivatives.

But the reaction of α‐*tert*‐alkyl radicals and olefins was faster than that of indoles. This controlled reactivity has resulted in an elegant three‐component reaction. Song and Li's group also reported the reaction of styrene derivatives (**108.1**), α‐bromocarbonyl compounds (**108.2**), and aniline derivatives (**108.3**) in the presence of Ru‐photocatalyst under visible light irradiation (Scheme 108).^[262]^ The reactivity of aniline derivatives is not so high compare with indoles. But the substrates they employed reacted smoothly under Ru‐photocatalyst conditions, producing **108.4**.

**Scheme 109:**
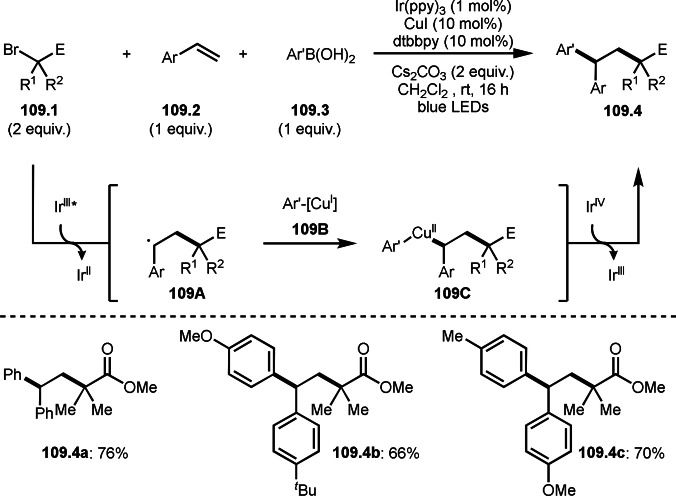
Ir‐catalyzed three‐component reaction using arylboronic acids.

Shu's group accomplished the reaction of α‐bromocarbonyl compounds (**109.1**), styrene derivatives (**109.2**), and arylboronic acids (**109.3**) in the presence of a Cu catalyst and an Ir‐photocatalyst under visible light irradiation (Scheme 109).^[263]^ To use arylboronic acids in the reaction, a transmetalation step is required. Therefore, they employed not only Ir‐photocatalyst but also Cu catalyst for transmetalation with arylboronic acid. Under their conditions, three‐component products (**109.4 a**–**c**) were obtained in moderate to good yields. An excited Ir^II^ catalyst reacted with **109.1** to give α‐*tert*‐alkyl radical. **109A** next was generated from the reaction of α‐*tert*‐alkyl radical and **109.2**. Transmetalation between the Cu salt and **109.3** occurred to form aryl copper species (**109B**), which reacted with **109A** to form alkyl copper species (**109C**). Final reductive elimination of **109C** produced **109.3**.

**Scheme 110:**
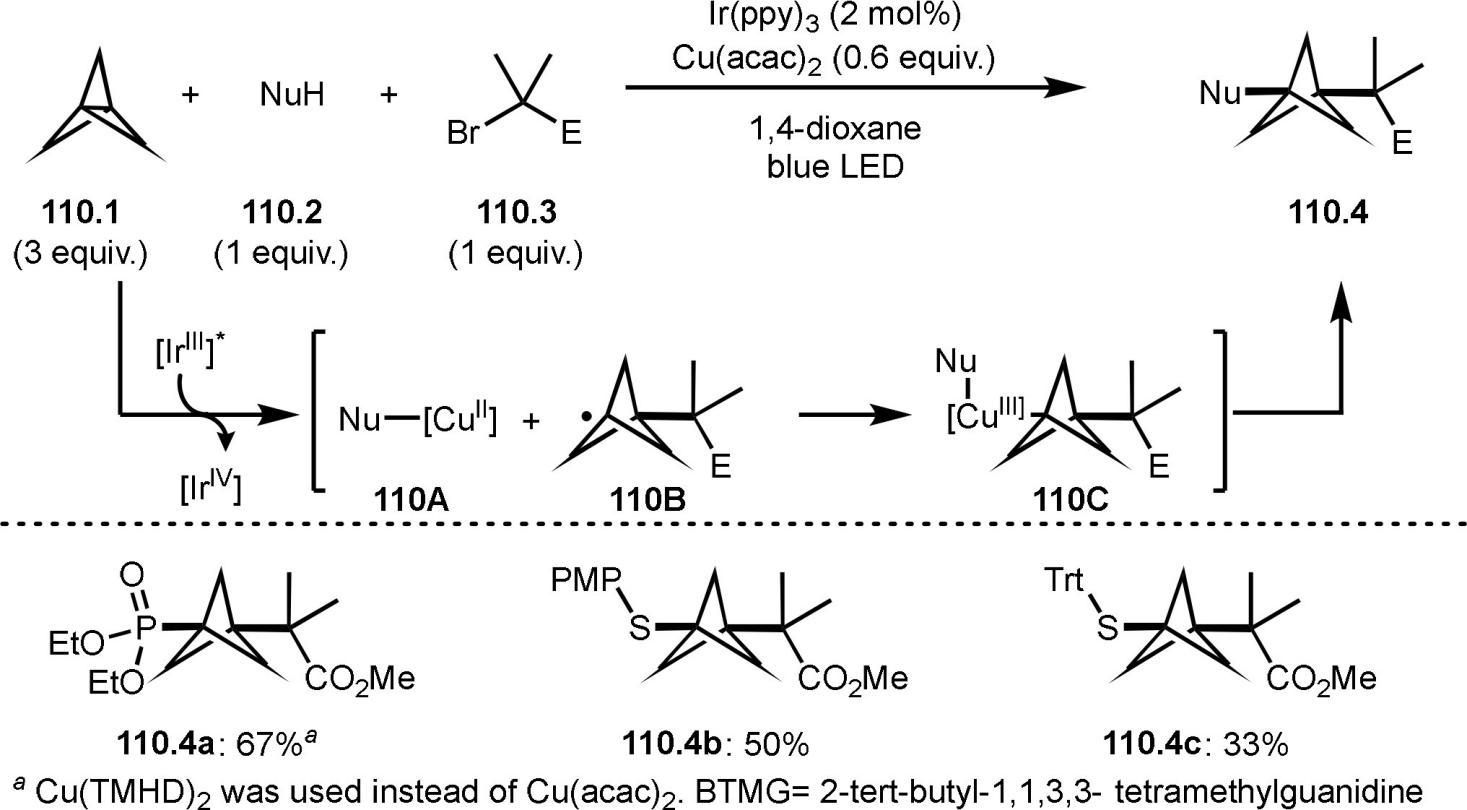
Ir‐catalyzed three‐component reaction using [1.1.1]‐propellane.

[1.1.1]‐Propellane undergoes two‐component reactions to give bicyclo[1.1.1]pentanes (BCPs), which were introduced in previous sections. MacMillan's group reported the three‐component reaction of [1.1.1]‐propellane (**110.1**), *P*‐, and *S*‐nucleophiles (**110.2**), and α‐bromocarbonyl compounds (**110.3**) in the presence of an Ir‐photocatalyst and a Cu salt under visible light irradiation (Scheme 110).^[264]^ The reactivity [1.1.1]‐propellane (**110.1**) is close to an electron‐rich C−C double, but it can be controlled in three‐component reaction. This reaction occurred via radical cross‐coupling processes, including reductive elimination. Nucleophilic reaction of EtO_2_P(O)H or R−SH (**110.2**) with Cu salt to give **110A**. On the other hand, when photoexcited Ir^II^ reacted with **110.3**, α‐tert‐alkyl radicals were formed, which reacted with **110.1** to form **110B**. **110B** then coupled with **110A** to give **110C**. Reductive elimination of **110C** gave the three‐component product (**110.4**). In this case, the reaction could be difficult to control, and a variety of products (**110.4 a**–**c**) were synthesized in moderate yields.

**Scheme 111:**
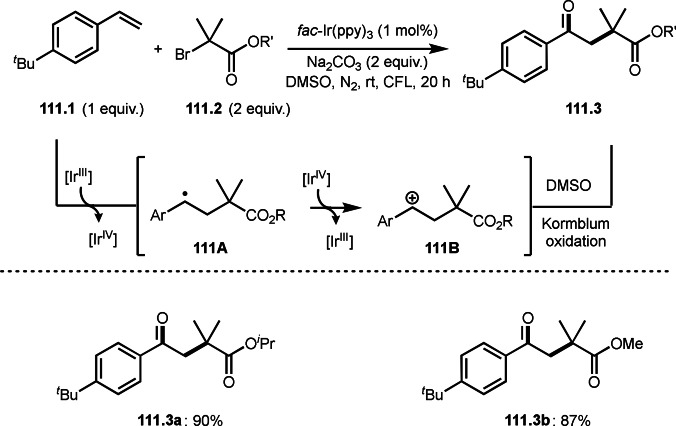
Ir‐catalyzed three‐component reaction using DMSO.

Three‐component products described above were composed of molecular fragments. Liu's group reported three‐component products composed of molecular fragments and a single oxygen atom from DMSO under ir‐photocatalyst conditions (Scheme 111).^[265]^ The reaction of styrene derivatives (**111.1**), α‐bromocarbonyl compounds (**111.2**), and DMSO gave the corresponding tert‐alkylated ketones (**111.3 a** and **b**) in good yields. The key in this reaction was Kornblum oxidation.^[266]^ The cationic species (**111B**) formed by Ir^IV^ oxidation of **111A** reacted with oxygen in DMSO to form **111.3**.

A photocatalyst is suitable for intra‐ or intermolecular cyclization with α‐*tert*‐alkyl radicals derived from α‐bromocarbonyl compounds. The following two sections present *tert*‐alkylation cyclization reactions in photoredox catalytic systems.

### 4.3 Intramolecular Cyclization

**Scheme 112:**
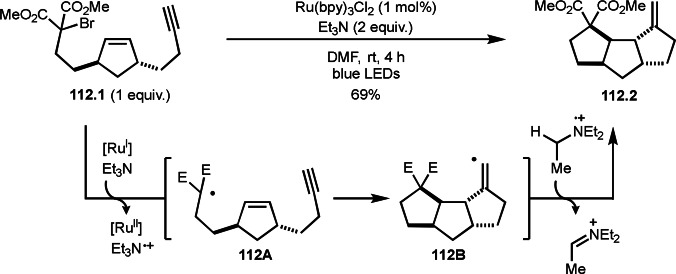
Intramolecular cyclization with an enyne by Ru‐photocatalyst.

**Scheme 113:**
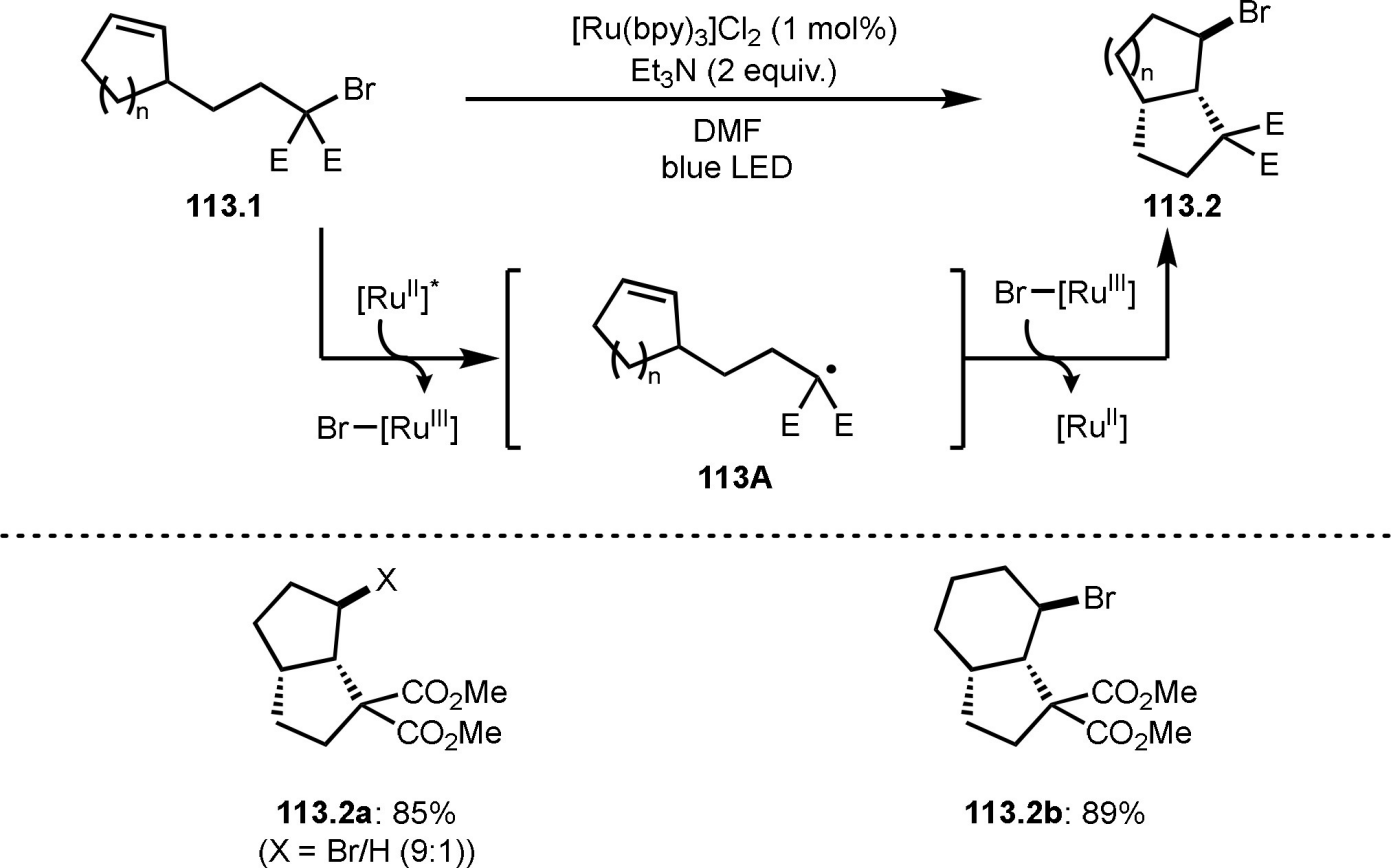
Intramolecular cyclization with cyclic olefins by Ru‐photocatalyst.

Stephenson'group reported the intramolecular cyclization of bromomalonates as an α‐*tert*‐alkyl radical source (Scheme 112).^[267a]^ They demonstrated various single cyclizations, which were also reported in next publication shown in Scheme 113.^[268]^ Highlight of their reaction is cascade radical cyclization of **112.1**. In the presence of Ru catalyst and sacrificial donor amine, **112.1** underwent intramolecular cascade cyclization to produce multicycles possessing a quaternary carbon center. Ru^II^ was first reduced with Et_3_N to generate Ru^I^ species, which then reacted with **112.1** to form **112A**. The carbon radical of **112A** reacted with the C−C double bond and then added to the C−C triple bond to form **112B**. The resulting radical **112B** captured a hydrogen atom from Et_3_N cation radical species to produce **112.2**. This reaction provided reductive cyclization, whereas their next report in Scheme 113 produced ATRC product in a trans manner. Reducing products were obtained when the product was a five‐membered ring. On the other hand, ATRC products were six‐membered rings (**113.2 b**). In the reaction with **113.1**, both ATRC and reductive cyclization occurred (**113.2 a**). Furthermore, vinyl radicals (**112B**) tended to undergo reductive reactions.

**Scheme 114:**
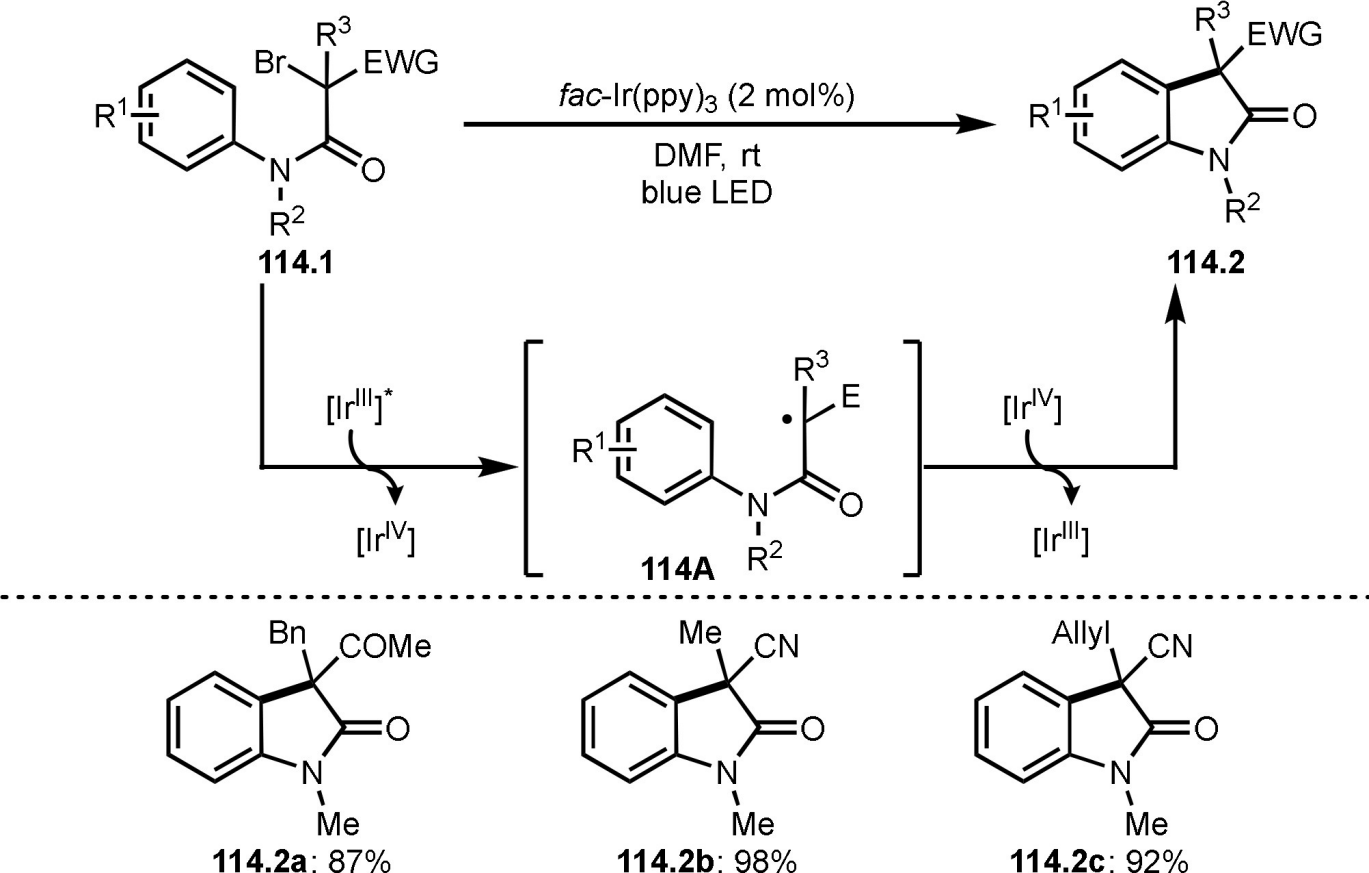
Intramolecular cyclization to give oxindoles by Ir‐photocatalyst.

Yu's group reported aromatic C−H intramolecular cyclization reaction with **114.1** to give oxindoles (**114.2**) (Scheme 114).^[269]^ Photo‐excited Ir‐catalyst reacted with **114.1** to form **114A**. **114.2** was finally obtained via cyclization/Ir^IV^‐oxidation/re‐aromatization process. The conditions were mild, but a substrates with EWG including ester, or nitrile were needed to carry out the reaction.

**Scheme 115:**
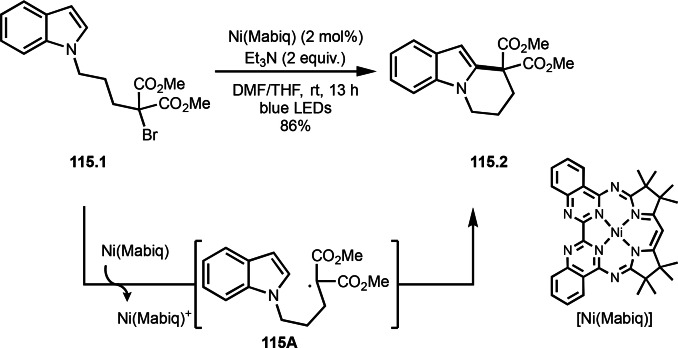
Intramolecular cyclization by Ni‐photocatalyst.

Ir or Ru photosensitizers, heavy transition metals, are quite effective for photoredox reaction. Bach and Hess's group successfully used an earth‐abundant Ni salt for an intramolecular cyclization under irradiation (Scheme 115).^[270]^ The reaction of **115.1** in the presence of Ni(Mabiq) (Mabiq: macrocyclic biquinazoline), photoredox catalyst, underwent efficient intramolecular cyclizations to give **115.2** in 86 % yield. Photo‐excited Ni^II^ catalyst reacted with alkyl amine to give active Ni^I^ catalyst, which underwent SET to **115.1** to form **115A**. **115.2** was obtained via C−H radical cyclization of **115A** followed by re‐aromatization.

**Scheme 116:**
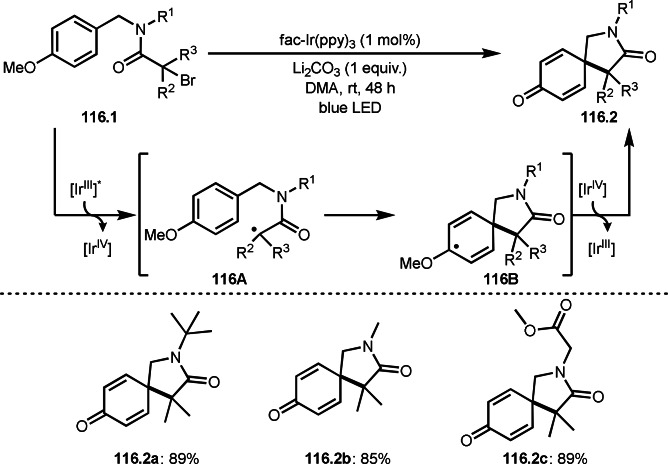
Intramolecular dearomative cyclization by Ir‐photocatalyst.

Intramolecular dearomative cyclization by Ir‐photocatalyst was reported by Zhang's group (Scheme 116).^[271]^ In Scheme 114, α‐bromocarboxamides (**114.1**) underwent aromatic C−H intramolecular cyclization, whereas the reaction α‐bromocarboxamides (**116.1**) in the presence of Ir‐photocatalyst provided dearomative products (**116.2 a**–**c**). The generated α‐tert‐alkyl radical (**116A**) underwent an addition reaction at the ipso position to give **116B**. Since the resulting **116B** had a quaternary carbon in the new bond, re‐aromatization did not occur. Finally, the MeO group in **116.1** was converted to a carbonyl group to give **116.2**.

### 4.4 Intermolecular Cyclization

**Scheme 117:**
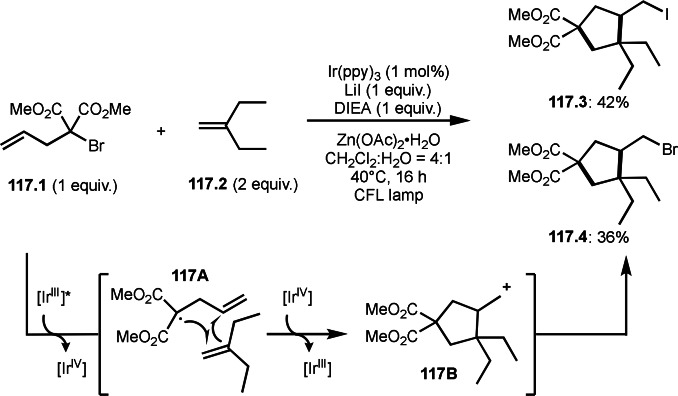
Intermolecular cyclization to give cyclopentanes by Ir‐photocatalyst.

Li and Yao's group performed intermolecular ATRC of **117.1** with **117.2** in the presence of Ir‐photocatalyst (Scheme 117).^[272]^ Their main purpose of this reaction is ATRC of dimethyl‐2‐(iodomethyl) cyclopropane‐1,1‐dicarboxylate with olefins under Ir‐photocatalyst, and the reaction shown in Scheme 117 was one of the control experiments to check the generation of **117B**. The reaction of **117.1** with **117.2** gave the mixture of **117.3** and **117.4**, 42 % and 36 %, respectively. The formation of iodide (**117.3**) was attributed to the reaction of **117B** and LiI.

**Scheme 118:**
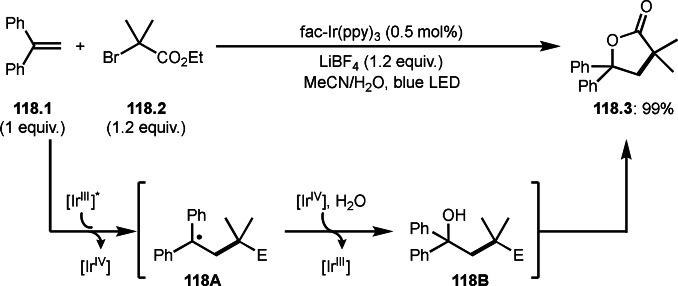
Intermolecular cyclization to give lactone by Ir‐photocatalyst.

Scheme 117 showed the synthesis of all carbon cyclic compounds, whereas Wu and Liu's group reported the synthesis of a heterocyclic compound, lactone (**118.3**) via Ir‐catalyzed intermolecular cyclization under photo‐irradiation (Scheme 118).^[273]^ In situ generated α‐*tert*‐alkyl radical species from the reaction of **118.2** and photo‐excited Ir^III^ reacted with **118.1** to give **118A**. **118A** was oxidized with Ir^IV^, followed by the addition of water to give **118B**. This process was confirmed by the addition of H_2_^18^O labeling experiment. When H_2_^18^O was used instead of water, ^18^O was incorporated into the product. Intramolecular esterification of **118B** gave lactone (**118.3**). LiBF_4_ promoted the final esterification process.

**Scheme 119:**
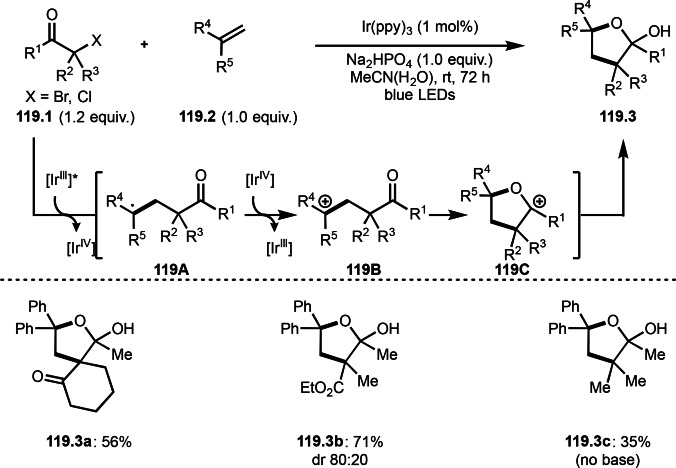
Intermolecular cyclization to give cyclopentyl hemiketals by Ir‐photocatalyst.

An α‐bromoester in intermolecular cyclization gave a lactone shown in Scheme 118. Lombardo's group reported that the reaction of α‐bromoketones (**119.1**) and olefins (**119.2**) provided cyclopentyl hemiketals (**119.3 a**–**c**) in the presence of Ir‐photocatalyst (Scheme 119).^[274]^ **119A**, generated from the reaction of **119.1** and photo‐excited Ir^III^ species, was oxidized by an Ir^IV^ species to form a cation species (**119B**). **119B** then underwent intramolecular cyclization to give **119C**. Cyclopentyl hemiketal (**119.3**) was obtained via the addition of water.

**Scheme 120:**
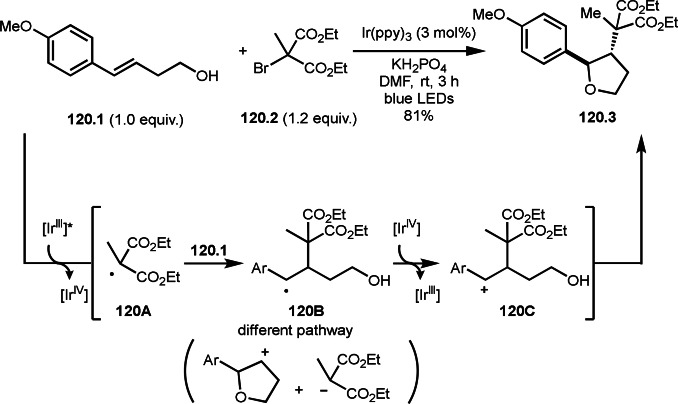
Intermolecular cycloetherification by Ir‐photocatalyst.

Intermolecular cyclization of olefins with α‐bromocarbonyl compounds gave lactones and cyclopentyl hemiketals. When a homoallylic alcohol was employed as a substrate, a cyclic ether compound was obtained, which was reported by Yang and Xia's group (Scheme 120).^[275]^ The reaction of homoallylic alcohol (**120.1**) with α‐bromocarbonyl compound (**120.2**) provided cycloetherification product (**120.3**) in the presence of Ir‐photocatalyst. α‐*tert*‐Alkyl radical species (**120A**) was added to **120.1** to give **120B**. **120B** was oxidized by an Ir^IV^ species to form a cation species (**120C**). The hydroxyl group of **120C** underwent an intramolecular cyclization to produce **120.3**. The product (**120.3**) was *trans*, probably due to steric hinderance of the *tert*‐alkyl group. In general, α‐*tert*‐alkyl radicals are less reactive toward internal olefins due to the steric reason, but the reaction occurred smoothly under these photocatalytic conditions. For this reason, they proposed a different mechanism, but one that goes through α‐*tert*‐alkyl anion is less likely.

**Scheme 121:**
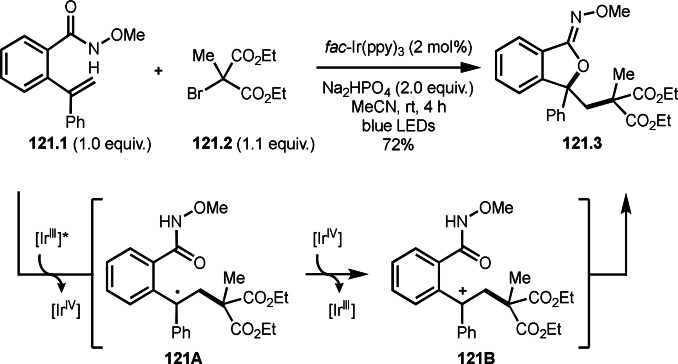
Intermolecular iminolactonization by Ir‐photocatalyst.

Because both nitrogen and oxygen atom in amides are nucleophilic, controlling their reactivity is not easy. Chen and Xiao's group accomplished *N*‐cyclization reaction of *o*‐vinyl‐*N*‐alkoxybenzamide (**121.1**) with α‐bromocarbonyl compound (**121.2**) in the presence of an Ir‐photocatalyst (Scheme 121).^[276]^ This intramolecular cyclization provided *O*‐cyclized iminolactone (**121.3**) in 72 % yield. The reaction of **121.1** and photo‐excited Ir^III^ species generated α‐*tert*‐alkyl radical species which reacted with **121.1** to form **121A**. **121A** was oxidized by an Ir^IV^ species to form a cation species (**121B**). *O*‐cyclization of **121** occurred to produce **121.3**.

**Scheme 122:**
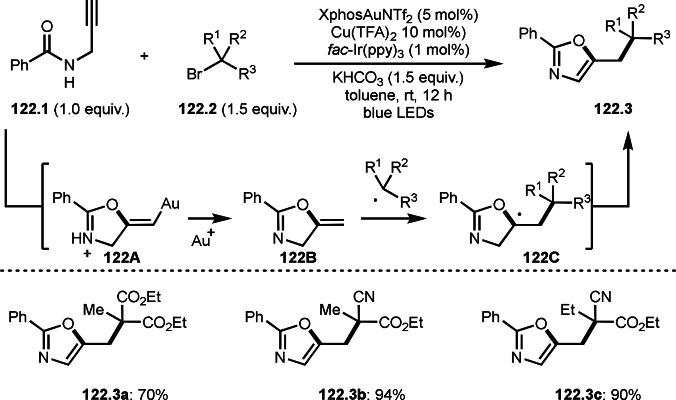
Intermolecular cyclization with alkynes by Ir‐photocatalyst.

In photoreactions catalyzed by a transition metal salt, alkynes are also good reaction partner. Zhao and Li's group reported intramolecular cyclization of terminal alkynes (**122.1**) with α‐bromocarbonyl compounds (**122.2**) to produce *tert*‐alkylated oxazoles (**122.3**) in the presence of an Ir‐photocatalyst, a Cu catalyst and an Au catalyst (Scheme 122).^[277]^ Au^I^ catalyzed cyclization of **122.1** to form **122B** via **122A**. Photo‐excited Ir^III^ species generated α‐*tert*‐alkyl radical species, which reacted with **122B** to form **122C**. **122C** was oxidized by a Cu^II^ salt followed by re‐aromatization to produce *tert*‐alkylated oxazoles (**122.3**). The reactions mainly occurred with bromomalonates or cyanomalonates (**122.2**) to produce various *tert*‐alkylated oxazoles (**122.3 a**–**c**) in good yields.

**Scheme 123:**
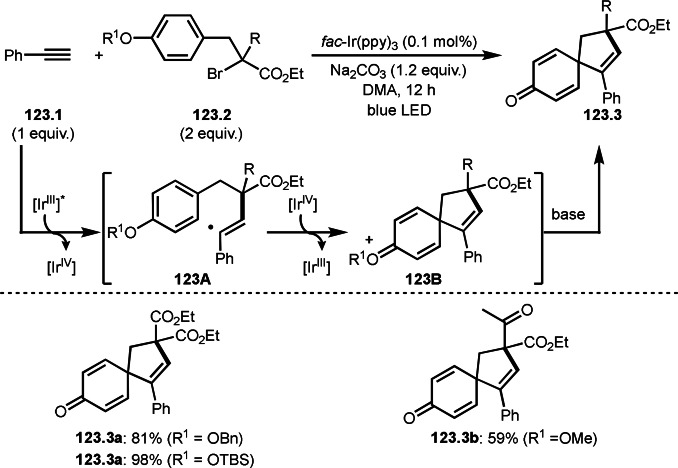
Intermolecular dearomative cyclization to give spirocycles possessing a cyclohexadienone by Ir‐photocatalyst.

**Scheme 124:**
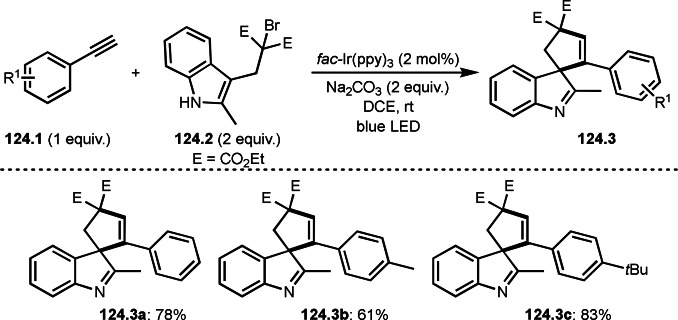
Intermolecular dearomative cyclization to give spiroindolenines by Ir‐photocatalyst.

**Scheme 125:**
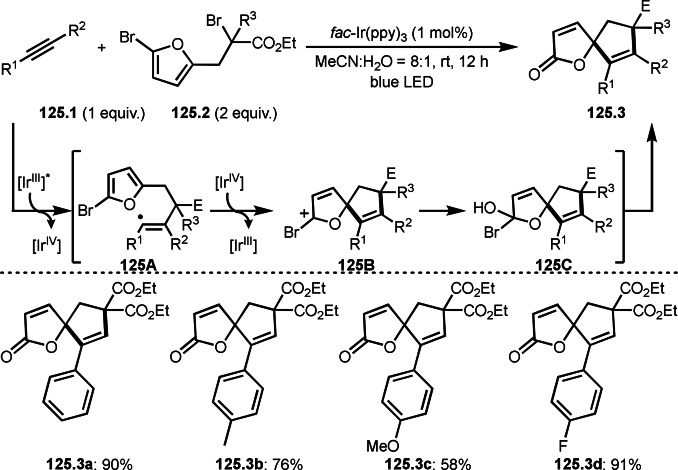
Intermolecular dearomative cyclization to give oxa‐spirocycles by Ir‐photocatalyst.

Zhang's group reported intermolecular dearomative cyclization of alkynes (**123.1**) and α‐bromocarbonyl compounds (**123.2**) to produce spirocycles possessing a cyclohexadienone (**123.3**) in the presence of an Ir‐photocatalyst (Scheme 123).[^278^, ^279^, ^280^, ^281^] Photo‐excited Ir^III^ species reacted with **123.1** to give the corresponding α‐*tert*‐alkyl radical species. In this case, they used α‐benzyl substituted α‐bromocarbonyl compounds (**123.2**). Since α‐bromocarbonyl compounds tend to undergo E2 elimination reactions to form β‐aryl substituted‐methacrylate derivatives (conjugated α,β‐unsaturated esters), the reactivity of α‐benzyl substituted α‐bromocarbonyl compounds in radical reactions is not always easy to control. But, under photocatalyst conditions, they successfully control the reactivities to undergo dearomative cyclizations. The resulting α‐*tert*‐alkyl radical species added to **123.1** to form **123A**. **123A** was oxidized by Ir^IV^ species to form an oxynium cation species (**123B**). Finally, a spirocycle (**123.3**) was released from **123B** in the presence of a base. Zhang's group also accomplished intermolecular dearomative cyclization of alkynes (**124.1**) and α‐bromocarbonyl compounds possessing an indole and furan fragment (**124.2** and **125.2**) to produce spirocycles (**124.3 a**–**c** and **125.3 a**–**d**) in the presence of an Ir‐photocatalyst (Scheme 124 and 125).[^282^, ^283^] The reaction smoothly occurred via similar pathways.

**Scheme 126:**
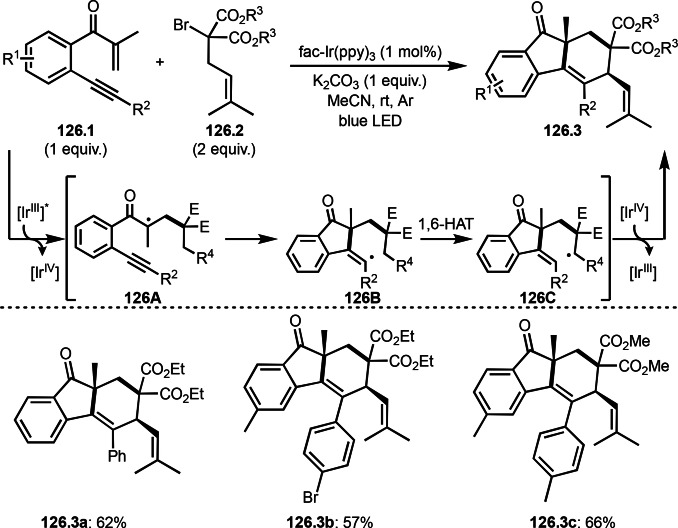
Tandem intermolecular bicyclization to give benzo‐fused bicycles by Ir‐photocatalyst.

Tandem intermolecular bicyclization to give benzo‐fused bicycles was reported by Hao, Tu, and Jiang's group (Scheme 126).^[284]^ The reaction of β‐alkynyl propenones (**126.1**) and allyl substituted α‐bromocarbonyl compounds (**126.2**) provided polyfunctionalized fluoren‐9‐ones (**126.3 a**–**c**) in moderated yields under Ir‐photocatalytic conditions. The substrates for this reaction have a variety of C−C multiple bonds that could react with α‐*tert*‐alkyl radicals, but the reaction is quite chemoselective. The *in situ* generated α‐*tert*‐alkyl radicals from the reaction of **126.2** with photoexcited Ir^III^ species selectively reacted with the C−C double bond of **126.1** to form **126A**. The resulting carbon radicals in **126A** reacted with the C−C triple bond to give **126B** *via* an intramolecular cyclization. Next, 1,6‐HAT in **126B** occurred and **126C** was formed. Finally, **126.3** was obtained by oxidation of **126C** with Ir^IV^ species followed by cyclization.

**Scheme 127:**
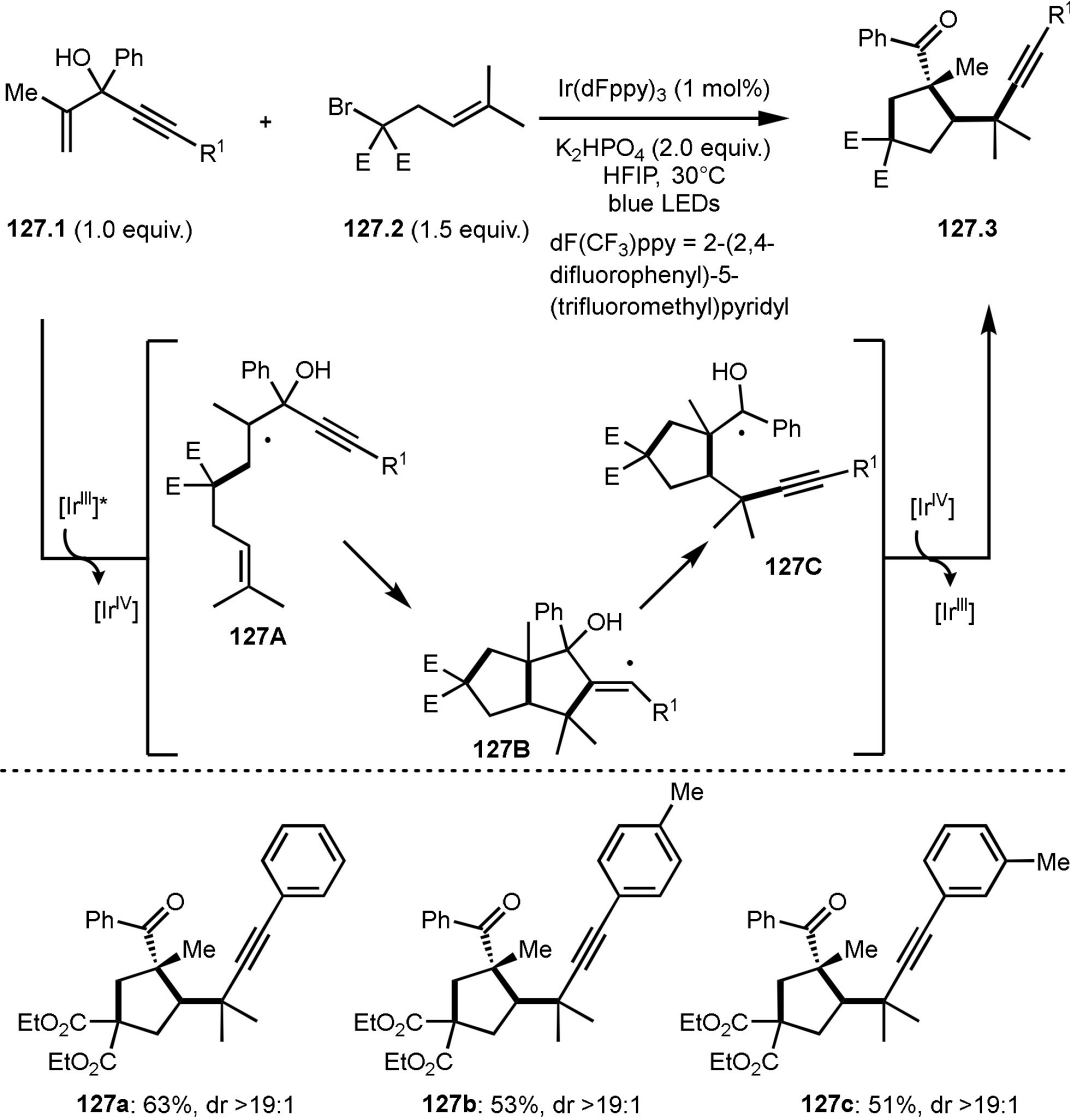
Intermolecular bicyclization involving alkynyl migration by Ir‐photocatalyst.

Tandem intermolecular bicyclization process is suitable for annulation with alkynyl migration reaction. Hao, Tu, and Jiang's group employed this process for the synthesis of alkynylated cyclic ketones (**127.3 a**–**c**) (Scheme 127).^[285]^ The reaction of in situ generated α‐*tert*‐alkyl radicals with **127.1** provided **127A**. **127A** underwent tandem bicyclization to give bicyclic radical intermediate (**127B**). Ring opening of **127B** provided **127C**, where the alkynyl migration reaction occurred. **127C** was oxidized by Ir^IV^ species to produce **127.3**.

**Scheme 128:**
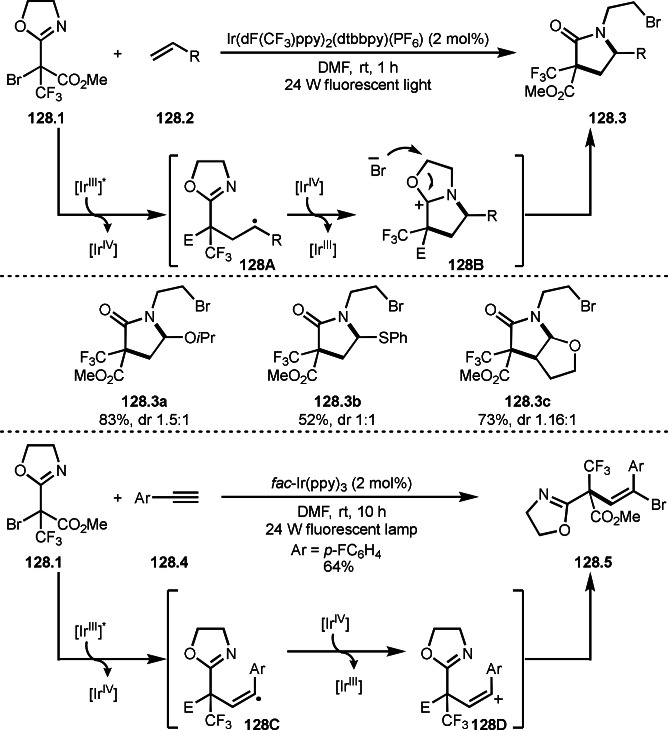
Ring‐opening reaction with olefins and ATRA with alkynes by Ir‐photocatalyst.

Chen and Guo's group reported the reaction of trifluoromethylated α‐bromocarbonyl compounds possessing an oxazolyl ring (**128.1**) and olefins (**128.2**) to give γ‐butyrolactams (**128.3**) under light‐induced Ir‐photocatalyst conditions (Scheme 128).^[286]^ Interestingly, the oxazolyl ring in **128.1** underwent a ring‐opening reaction in the presence of an olefin substrate, followed by lactamization to form a γ‐butyrolactam ring. The reaction started with the generation of α‐*tert*‐alkyl radicals (**128A**) from the reaction of **128.1** with photoexcited Ir^III^ species. **128A** underwent radical cyclization followed by Ir^IV^ oxidation to produce **128B**. Finally, nucleophilic attack on **128B** by a bromide anion produced **128.3** *via* a ring‐opening reaction. On the other hand, the reaction with alkynes (**128.4**) gave simple ATRA products (**128.5**) instead of a ring‐opening product. The vinyl radical intermediate (**128C**), unlike the alkyl radical intermediate (**128A**), may not be suitable for ring‐opening reactions, probably because of its rigid structure.

**Scheme 129:**
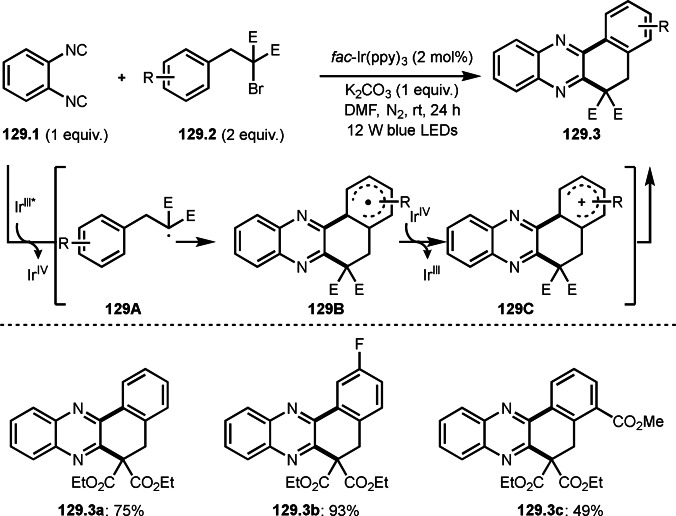
Intermolecular bicyclization with *o*‐diisocyanoarenes by Ir‐photocatalyst.

Zhang'group reported a visible‐light‐induced radical cascade intermolecular cyclization of *o*‐diisocyanoarenes (**129.1**) with benzyl substituted α‐bromocarbonyl compounds (**129.2**) to construct a benzo[a]phenazine skeletons (**129.3 a**–**c**) (Scheme 129).^[287]^ An isocyano group is also a good radical acceptor. α‐*tert*‐Alkyl radicals (**129A**) from the reaction of **129.2** with photoexcited Ir^III^ species reacted with isocyano group in **129.1**. The resulting imidoyl radical intermediate underwent radical cyclization to form **129B** which was oxidized by Ir^IV^ species to give **129C**. **129.3** was finally obtained through re‐aromatization of **129C**.

**Scheme 130:**
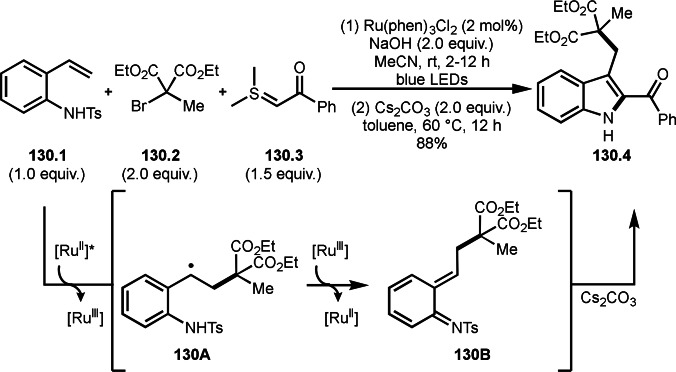
Intermolecular cyclization via aza‐*ortho*‐quinone methide by Ru‐photocatalyst.

Chen and Xiao's group three‐component intermolecular cyclization via an aza‐*ortho*‐quinone methide as an active intermediate (Scheme 130).^[288]^ The reaction of *N*‐tosyl‐2‐vinylaniline (**130.1**), α‐bromocarbonyl compounds (**130.2**) and sulfur ylide (**130.3**) produced indole derivatives (**130.4**). They successfully controlled three substrates under photocatalyst system. The reaction began with the generation of α‐*tert*‐alkyl radical species from the reaction of **130.2** with photoexcited Ru^II^ species. α‐*tert*‐alkyl radical species added to **130.1** to form benzylic radical species (**130A**). Next, aza‐*ortho*‐quinone methide (**130B**) was formed from Ru^III^ oxidation of **130A**. Finally, the [4+1] cycloaddition of **130B** and **130.3** was followed by aromatization/deprotection of the Ts group to produce **130.4**.

**Scheme 131:**
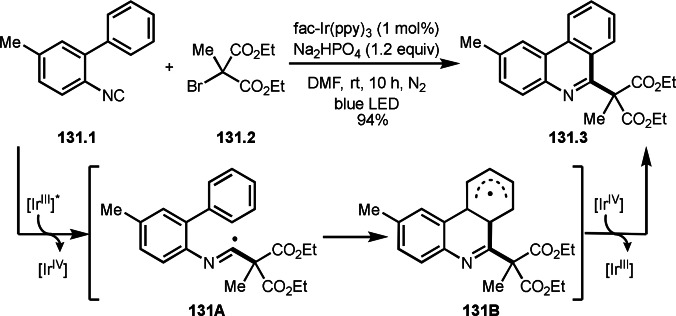
Intermolecular cyclization with biphenyl isocyanide by Ir‐photocatalyst.

As was demonstrated by Zhang'group, isocyano group was a good α‐*tert*‐alkyl radical acceptor in intermolecular radical bicyclization. Zhang and Yu's group reported the synthesis of phenanthridine derivatives (**131.3**) from the reaction of biphenyl isocyanide (**131.1**) and α‐bromocarbonyl compounds (**131.2**) under Ir‐photocatalyst conditions (Scheme 131).^[289]^ The resulting imidoyl radical intermediate (**131A**) from the reaction of **131.1** and α‐*tert*‐alkyl radical underwent radical cyclization to form **131B**. **131.3** was obtained via re‐aromatization. Similar single intermolecular cyclization was also reported by Wang's group, in which Co catalyst was employed.^[290]^

**Scheme 132:**
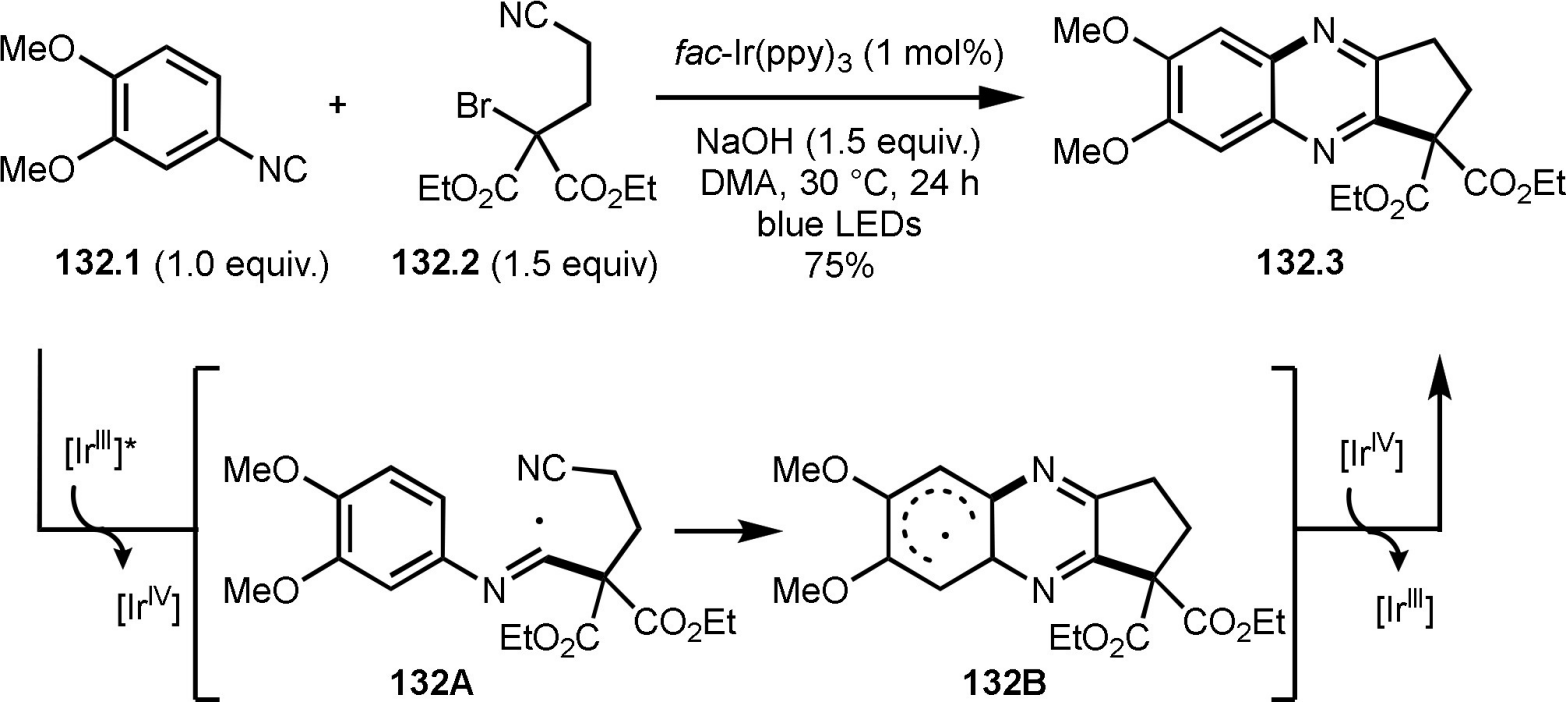
The combination of cyano and isocyano group reactivity for intermolecular cyclization.

The combination of cyano and isocyano group reactivity resulted in interesting cyclization reactions. Yu's group reported the reaction of aryl isocyanide (**132.1**) and cyano alkylated α‐bromocarbonyl compounds (**132.2**) to produce fused quinoxaline derivatives (**132.3**) under Ir‐photocatalyst conditions, in which similar structure shown in Scheme 129 was produced (Scheme 132).^[291]^ In situ generated α‐*tert*‐alkyl radical species reacted with **132.1** to give imidoyl radical intermediate (**132A**). The resulting imidoyl radical intermediate (**131A**) further cyclized to form **132B**. Fused quinoxaline derivative (**132.3**) was obtained via Ir^IV^ oxidation/re‐aromatization.

**Scheme 133:**
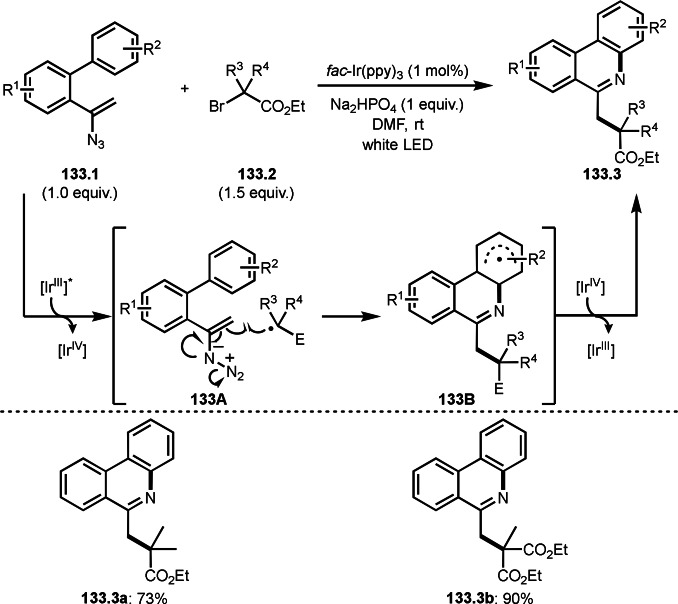
Intermolecular cyclization *via* iminyl radical species by Ir‐photocatalyst.

Yu's group found the C−C double bond of vinyl azide was a good α‐*tert*‐alkyl radical acceptor (Scheme 133).^[292]^ The reaction of vinyl azide (**133.1**) and α‐bromocarbonyl compounds (**133.2**) to produce phenanthridines (**133.3**) under Ir‐photocatalyst conditions. The reaction of photoexcited Ir^III^ species and **133.2** generated α‐*tert*‐radicals, which then reacted with **133.1** to efficiently form an iminyl radical intermediate. The resulting iminyl radical next added to R^2^‐substituted aryl group to give **133B**. **133B** was oxidized by Ir^IV^ species followed by re‐aromatization to produce **133.3**.

**Scheme 134:**
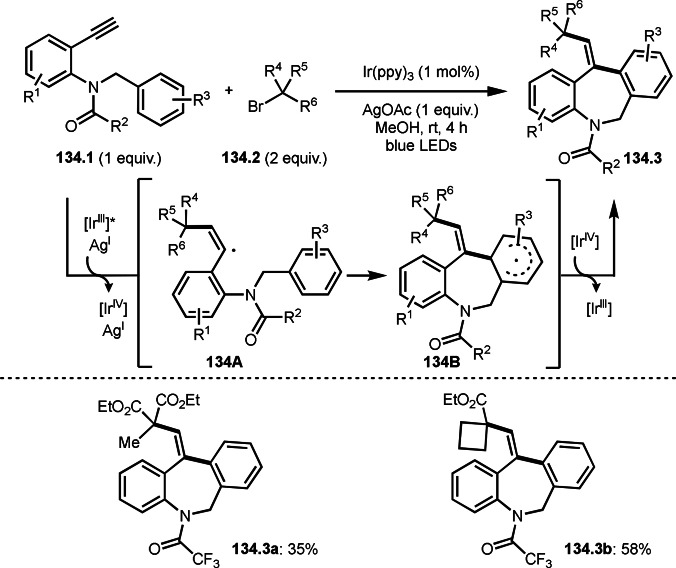
The construction of seven‐membered rings under Ir‐photocatalyst conditions.

Hu and Li reported the construction of seven‐membered rings (**134.3 a**, **b**) through intermolecular cyclization of alkynes (**134.1**) and α‐bromocarbonyl compounds (**134.2**) under Ir‐photocatalyst conditions (Scheme 134).^[293]^ α‐*tert*‐Alkyl radical species were first generated by Ir‐photocatalyst with aid of silver salt. The addition of silver salts as a scavenger of bromide ion accelerated the generation of the α‐*tert*‐alkyl radical intermediate from bromide (**134.2**). Without silver salt, the yield was slightly dropped. Photo‐excited Ir^II^ species are powerful enough to reduce α‐bromocarbonyl compounds, but in this case a silver support was required. The resulting radicals added to **134.1** to form a vinylic radical (**134A**). **134A** underwent intramolecular cyclization to form a seven‐membered ring radical intermediate (**134A**). **134.3** was obtained *via* Ir^IV^ oxidation/re‐aromatization.

**Scheme 135:**
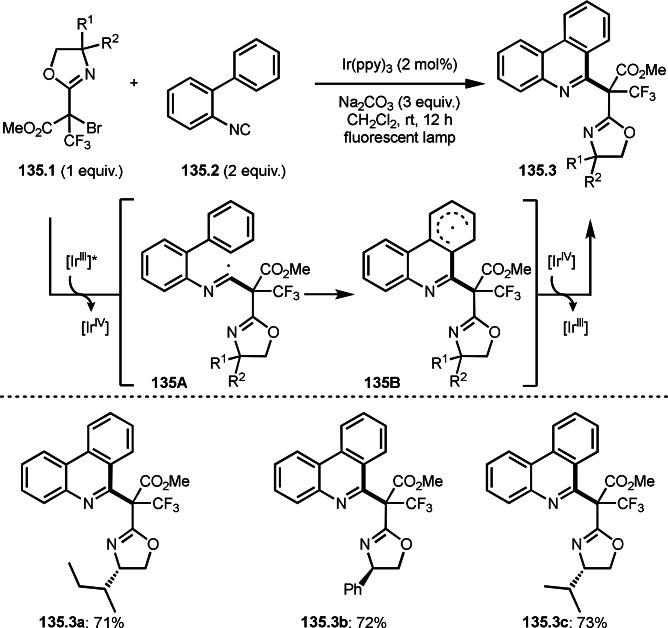
Intermolecular cyclization to give trifluoromethylated quaternary carbon compounds by Ir‐photocatalyst.

Oxazolyl ring in radical reaction underwent the ring opening reaction in Scheme 128. But next reaction did not give a ring‐opening product of oxazolyl ring. Wang, Guo and Chen's group reported the reation of trifluoromethylated α‐bromocarbonyl compounds possessing an oxazolyl ring (**135.1**) and biphenyl isocyanides (**135.2**) to give trifluoromethylated quaternary carbon compounds possessing an oxazolyl ring (**135.3 a**–**c**) under light‐induced Ir‐photocatalyst conditions (Scheme 135).^[294]^ In proposed reaction mechanism, photo‐excited Ir^II^ species reacted with **135.1** to generate α‐*tert*‐alkyl radical species, which added to **135.2** to form a vinylic radical intermediate (**135A**). **135A** underwent cyclization to give **135B**. Finally, the Ir^IV^ oxidation/re‐aromatization of **135B** produced **135.3**.

**Scheme 136:**
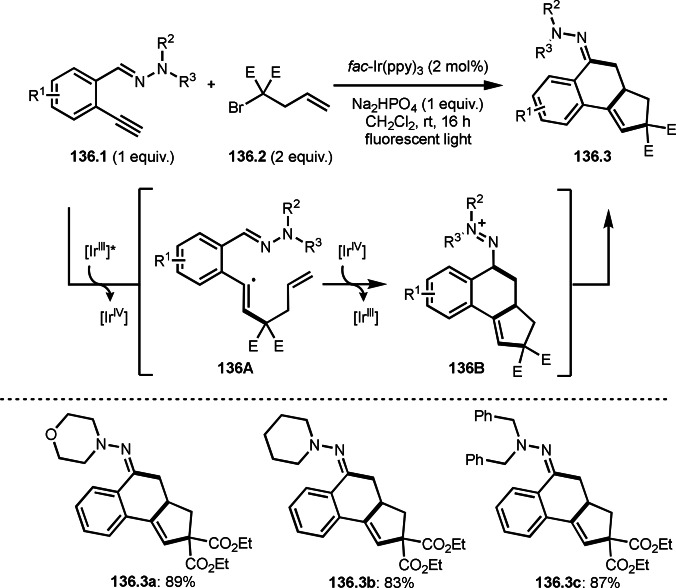
Intermolecular cyclization to give cyclohexylidenehydrazine‐fused polycycles by Ir‐photocatalyst.

The C−N double bond of hydrazon can react with α‐*tert*‐alkyl radical species. Zhu reported the reaction of 2‐ethynylaldehyde hydrazones (**136.1**) and allylated α‐bromocarbonyl compounds (**136.2**) to produce cyclohexylidenehydrazine‐fused polycycles (**136.3 a**–**c**) (Scheme 136).^[295]^ Hydrazon fragment is stable during the radical reaction. In situ generated α‐*tert*‐alkyl radical species added to **136.1** to form a vinylic radical intermediate (**136A**). **136A** was then cyclized with the allyl group of **136.2** and the resulting amidyl radical was oxidized by Ir^IV^ to give **136B**. Finally, proton elimination from **136B** produced **136.3**.

**Scheme 137:**
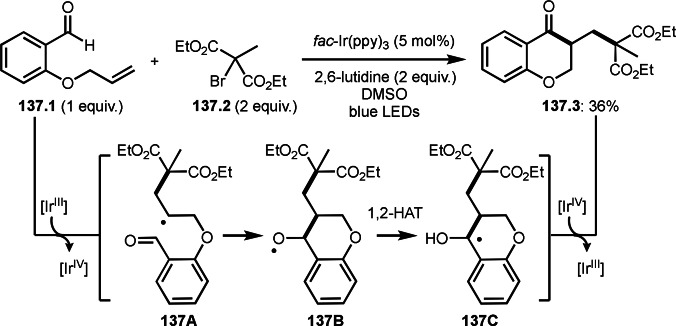
Intermolecular cyclization to give chroman‐4‐ones by Ir‐photocatalyst.

Huang reported the synthesis of chroman‐4‐ones (**137.3**) *via* cascade radical addition/cyclization of 2‐(allyloxy)arylaldehyde (**137.1**) with α‐bromocarbonyl compound (**137.2**) under Ir‐photocatalyst conditions (Scheme 137).^[296]^ The reaction is involving radical addition to aldehyde and 1,2‐HAT process. First, α‐*tert*‐alkyl radical species were formed from the reaction of photoexcited Ir^III^ species with **137.2**. α‐*tert*‐Alkyl radical species then added to **137.1** to give **137A**. The carbon radical of **137A** reacted with aldehyde to give an oxyl radical species (**137B**), which immediately underwent 1,2‐HAT to give **137C**. The desired product (**137.3**) was produced via the Ir^IV^ oxidation/carbonyl formation.

### 4.5 Rearrangement

**Scheme 138:**
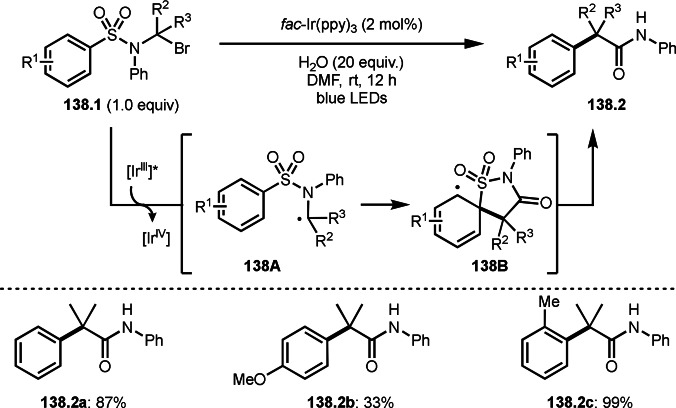
Rearrangement reaction by Ir‐photocatalyst.

One of the methodologies to introduce an aryl group at the carobonyl α‐position of an α‐bromocarbonyl compound is a rearrangement reaction. Wan and Zhang's group reported intermolecular radical addition/1,2‐aryl migration reaction of α‐bromocarbonyl compound (**138**) to produce tert‐alkylated arenes (**138.2 a**–**c**) *via* visible light‐induced Ir‐catalyst conditions (Scheme 138).^[297]^ In general, an α‐*tert*‐alkyl radical species from an α‐bromocarbonyl compound reacted with C−C multiple bonds, aromatice C−H bonds and other nucleophiles. Using this reaction characteristic, they achieved a reaction in which bond formation and dissociation occur continuously, i. e., a rearrangement reaction. In situ generated α‐*tert*‐alkyl radical species (**138A**) underwent dearomative addition reaction to form **138B**. After desulfonylation/re‐aromatization, **138.2** was obtained.

### 4.6 Cross‐Coupling Reaction

**Scheme 139:**
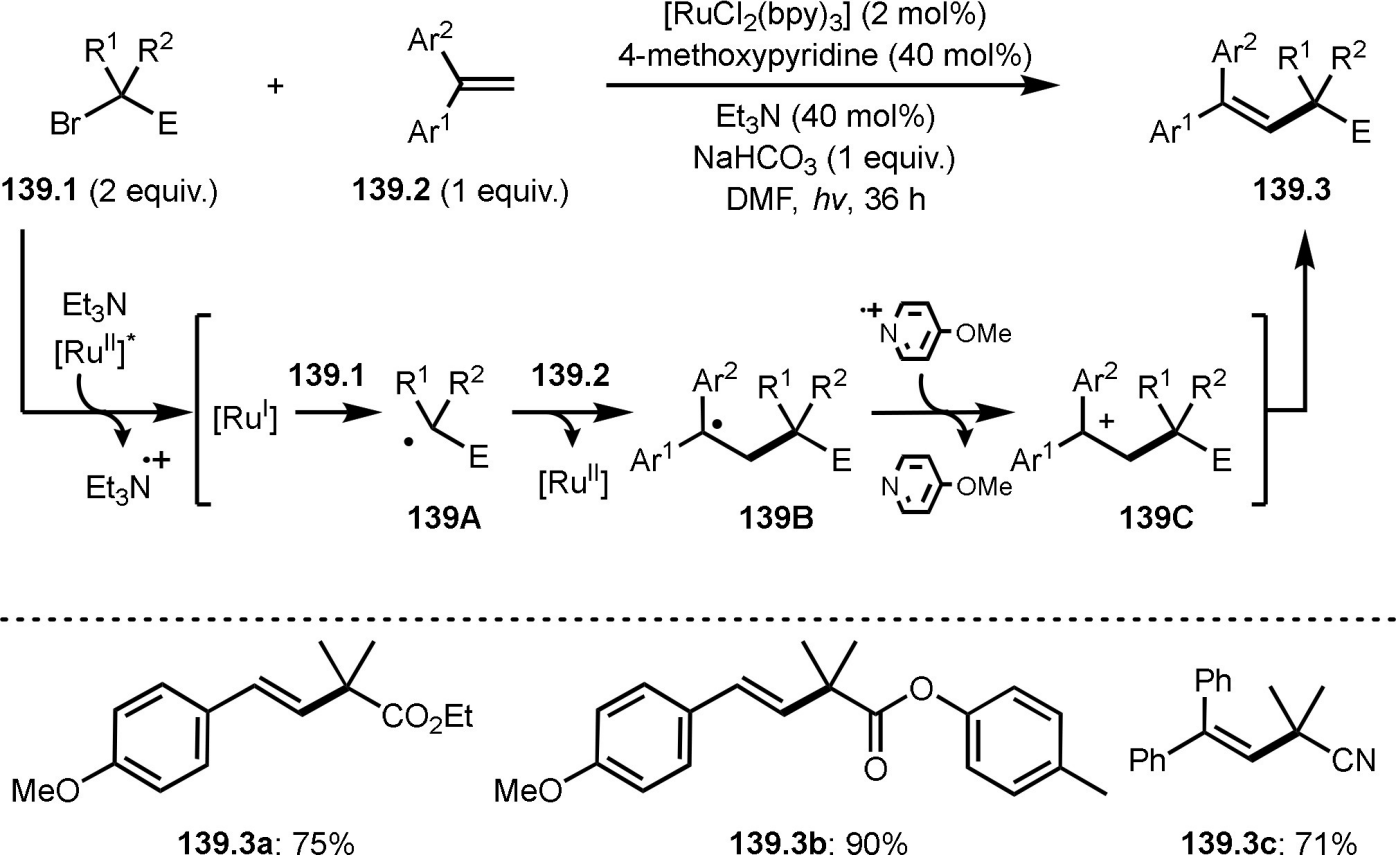
ATRS by Ru‐photocatalyst.

A Cu catalyst is suitable for ATRS reactions (Heck‐like olefination) of α‐bromocarbonyl compounds, in which α‐*tert*‐alkyl radical species can be generated from the reaction of α‐bromocarbonyl compound and a Cu^I^ species. Lei's group discovered different catalyst system to carry out ATRS reaction of α‐bromocarbonyl compound (**139.1**) with olefins (**139.2**) (Scheme 139).^[298]^ They employed Ru‐photocatalyst to generate α‐*tert*‐alkyl radical species from **139.1**. A photo‐excited Ru^III^ species reacted with sacrificial reductant 4‐methoxypyridine to form Ru^II^ species, which reacted with **139.1** to afford α‐*tert*‐alkyl radical species (**139A**). The resulting **139A** added to **139.2** to form **139B**. **139B** was oxidized by 4‐methoxypyridine cation radical, and the resulting cation species (**139C**) underwent proton elimination to produce **139.3**. The results were similar to the Cu‐catalyst system.

**Scheme 140:**
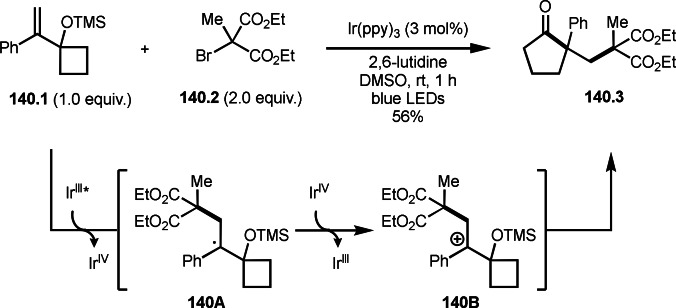
Ring expansion under Ir‐photocatalyst system.

Under Ir‐photocatalyst conditions, the reaction of styrene (**140.1**) possessing a four‐membered ring with α‐bromocarbonyl compound (**140.2**) gave a ring expansion product (**140.3**) in 56 % yield, which was reported by Kim's group (Scheme 140).^[299]^ The reaction shown in Scheme 139 underwent proton elimination after oxidation of **139A**. In this case, neighboring for‐membered ring in **140B** decomposed to form five‐membered ring (**140.3**), in which proton elimination to form an ATRS product did not occur.

**Scheme 141:**
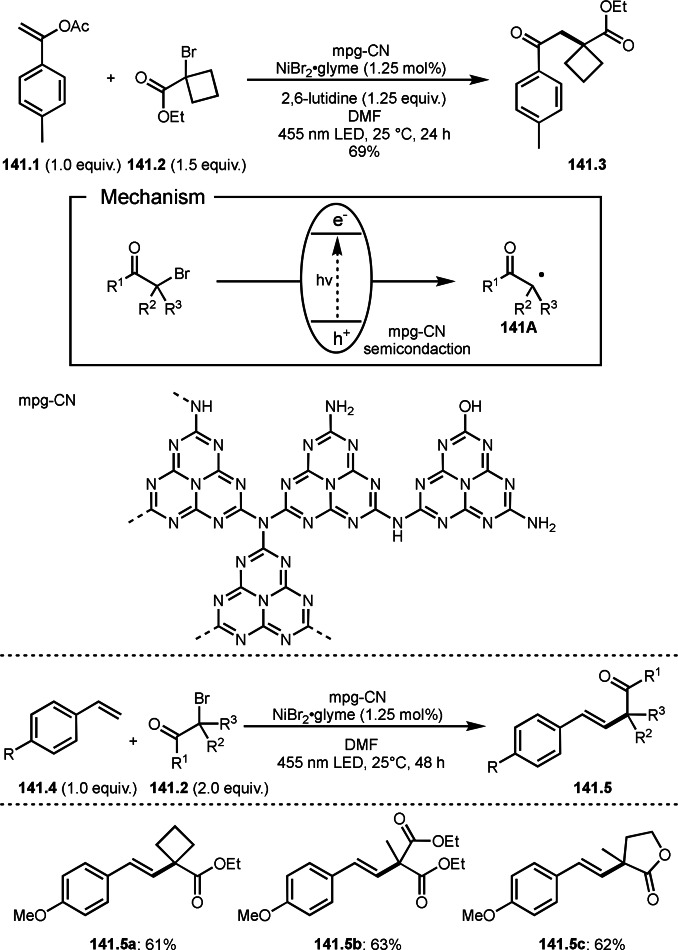
The reaction of vinyl acetate under mpg‐CN‐photocatalyst system.

A vinyl acetate has an electron‐rich C−C double bond, which is suitable for the reaction with α‐tert‐alkyl radicals. König reported the reaction of vinyl acetate (**141.1**) and α‐bromocarbonyl compounds (**141.2**) in the presence of a Ni catalyst in the presence of semiconductor photocatalysis (mesoporous graphitic carbon nitride: mpg‐CN) and a Ni catalyst (Scheme 141).^[300]^ Photoexcited mpg‐CN underwent SET to produce α‐tert‐alkyl radicals. The resulting α‐tert‐alkyl radicals added to **141.1** to produce **141.3** through ATRS mechanism. They described that *“the exact role of the nickel (II) salt is not clear; we speculate that the nickel salt acts as a Lewis acid to coordinate with the ester group of the alkyl bromide and thereby lowers the reduction potential of the alkyl bromide*.” The reaction mechanism is not clear, but the applicable substrate is not only vinyl acetate (**141.1**), but also styrene (**141.4**) which gives **141.5**.

**Scheme 142:**
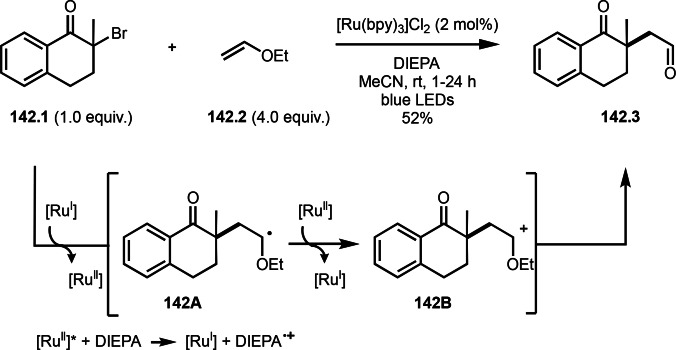
The reaction of vinyl ether under Ru‐photocatalyst system.

A vinyl ether is an electron‐rich C−C double bond and an aldehyde equivalent. Cordero‐Vargas's group reported the reaction of α‐bromocarbonyl compound (**142.1**) with vinyl ether (**142.2**) to produce the corresponding aldehyde (**142.3**) (Scheme 142).^[301]^ In situ generated active Ru^II^ catalyst reacted with **142.1** to form α‐*tert*‐alkyl radical species, which then added to **142.2** to afford **142A**. **142A** was oxidized by Ru^II^ or amine cation radical to give the corresponding cation species (**142B**), which underwent proton elimination to produce **142.3**.

**Scheme 143:**
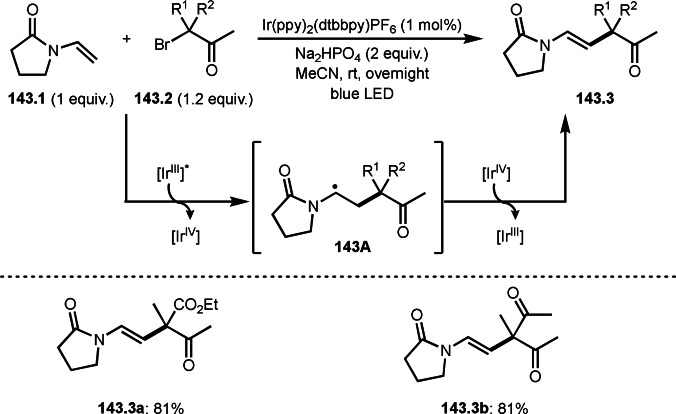
ATRS with enamide under Ir‐photocatalyst system.

Zhang and Yu's group reported ATRS reaction of enamide (**143.1**) with α‐bromocarbonyl compound (**143.2**) to produce *tert*‐alkylated enamides (**143.3 a**–**b**) under Ir‐photocatalyst conditions (Scheme 143).^[302]^ The reaction followed the ATRS reaction mechanism and produced (*E*)‐product **(143.3**) after the formation of α‐*tert*‐alkyl radical species by photoexcited Ir^II^ species.

**Scheme 144:**
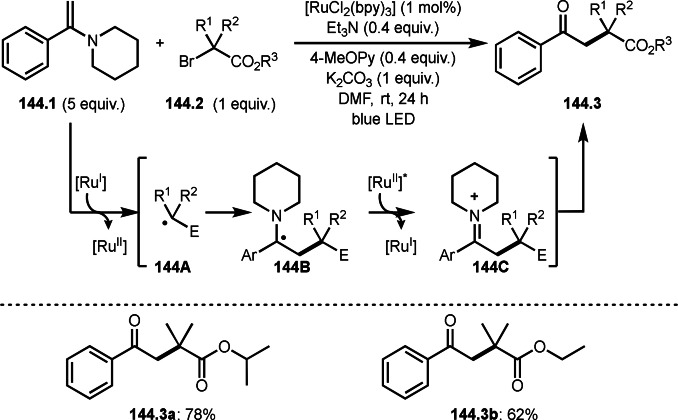
Ketone formation under Ru‐photocatalyst conditions.

On the other hand, the reaction of enamine (**144.1**) with α‐bromocarbonyl compound (**144.2**) gave different products, ketones (**144.3**), which was reported by Zhang's group(Scheme 144).^[303]^ This reaction also followed ATRS reaction after the generation of α‐*tert*‐alkyl radical species by Ru^I^ species. However, a ketone product (**144.3**) was formed after hydrolysis of the iminium ion (**144C**) formed by oxidation of **144B** with Ru^II^. This may be attributed to the instability of enamine against water.

**Scheme 145:**
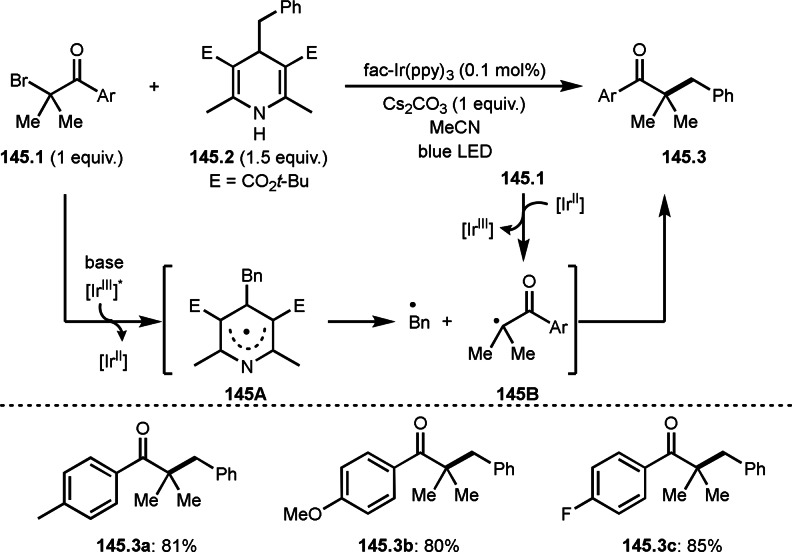
C(sp^3^)−C(sp^3^) cross‐couplings with alkylated Hantzsch ester by Ir‐photocatalyst.

A C(sp^3^)−C(sp^3^) cross‐coupling is one of the most difficult reactions. Because the reactivity of alkyl fragment is very difficult. For example, alkyl metal species have a risk to undergo β‐hydride elimination. An alkyl cation is unstable to give C−C double bond via E1 reaction. An alkyl anion and radical are also candidates for the couplings, but the reactivity of the two different alkyl species is difficult to control. Ma, Cheng, and Li's group reported C(sp^3^)−C(sp^3^) cross‐couplings of α‐bromocarbonyl compound (**145.1**) and benzylated Hantzsch ester (**145.2**) to produce quaternary carbon compounds (**145.3 a**–**c**) under Ir‐photocatalyst conditions (Scheme 145).^[304]^ The reaction basically underwent radical‐radical couplings. Benzyl radical was generated from the reaction of **145.2** with photo‐excited Ir^III^ species, in which a radical intermediate (**145A**) was formed. Upon generation of α‐*tert*‐alkyl radical species by Ir^II^ species, radical‐radical coupling immediately occurred to give **145.3**.

**Scheme 146:**
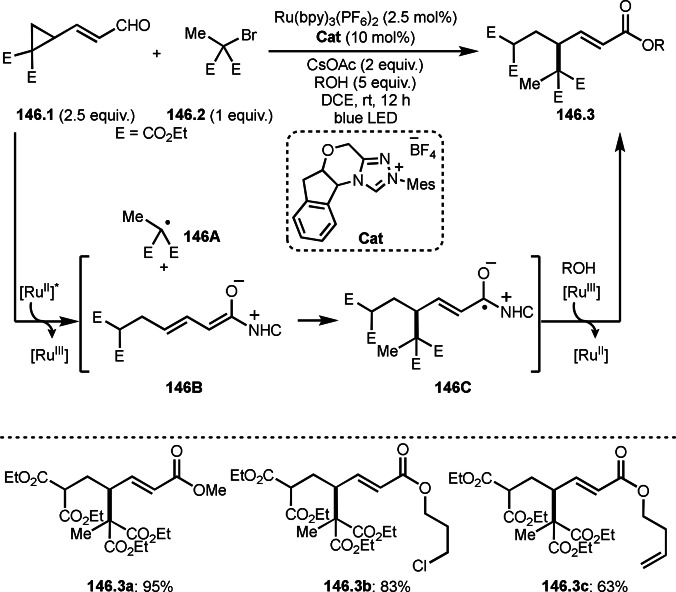
Ru‐photocatalyst/NHC enabling ring opening *tert*‐alkylation.

Ye's group reported interesting catalyst system of cyclopropane enal (**146.1**) that is the combination of Ru‐photocatalyst and NHC (*N*‐heterocyclic carbene) organocatalyst for ring opening *tert*‐alkylation (Scheme 146).^[305]^ The reaction of photo excited Ru^II^ species with α‐bromocarbonyl compound (**146.2**) provided **146A**. NHC catalyst generated homoenolate radical (**146B**) possessing diene structure. α‐*tert*‐alkyl radical species (**146A**) reacted with diene moiety in **146B** to form **146C**. **146.3** was produced *via* oxidation of **146C** with Ru^III^ species followed by the substitution with ROH. Under the conditions, **146.3 a**–**c** were obtained in moderate to good yields.

**Scheme 147:**
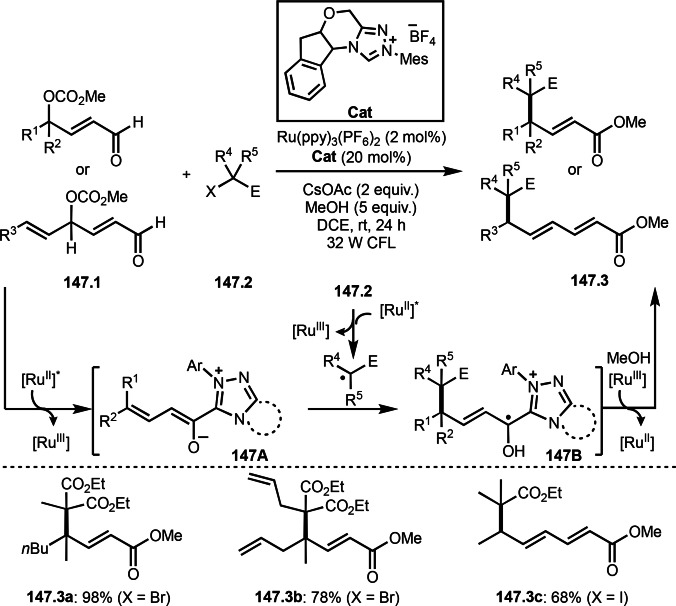
Ru‐photocatalyst/NHC enabling formal allylic substitution.

Formal allylic substitution reactions of allylic carbonates (**147.1**) possessing a formyl group with α‐bromocarbonyl compounds (**147.2**) were accomplished in a Ru‐photocatalyst/NHC combined catalyst system, which was reported by Gao and Ye's group (Scheme 147).^[306]^ Under the conditions, various *tert*‐alkylated unsaturated esters (**147.3 a**–**c**) were synthesized in moderate to good yields. The reaction of NHC and **147.1** provided electron‐rich enolate species. The resulting enolate species smoothly reacted with electron deficient α‐*tert*‐alkyl radical species to give **147B**. The radical addition only occurred at the terminal alkene in this step. **147B** then underwent oxidation/MeOH substitution to produce **147.3**.

**Scheme 148:**
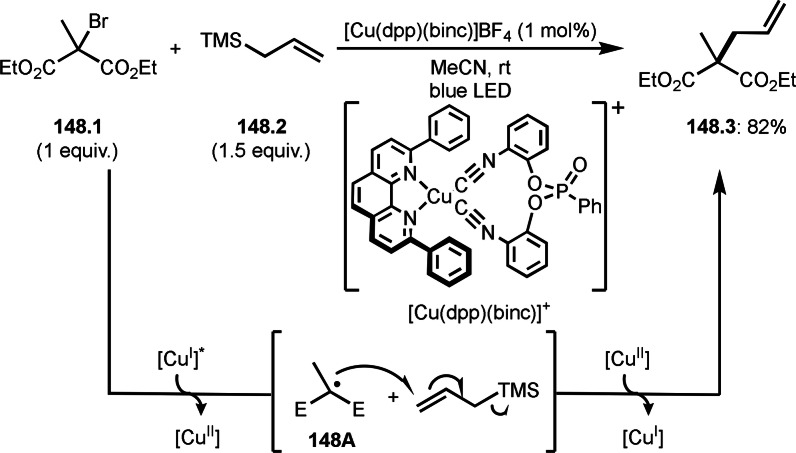
Allylation under Cu‐photocatalyst.

Reiser reported allyl couplings of α‐bromocarbonyl compound (**148.1**) with allyl silane (**148.2**) to produce *tert*‐alkylated propene (**148.3**) in the presence of Cu‐photocatalyst (Scheme 148).^[307]^ As previously described, Cu^I^/multidentate nitrogen ligand complexes can react with an α‐bromocarbonyl compound to produce α‐*tert*‐alkyl radical species *via* smooth SET. In this case, photo‐excited Cu^I^/phenanthroline/bisisonitrile complex played an important role in the radical generation step. In general, α‐*tert*‐alkyl radical species have enough reactivity for unactivated olefins. But **148A** smoothly added to **148.2** to give **148.3** in good yield. In this paper, they mainly focused on the use of benzylic halides as a primary radical source and α‐bromocarbonyl compounds as a secondary alkyl radical source. Therefore, the *tert*‐alkylation result is only one example.

### 4.7 Homocoupling

**Scheme 149:**
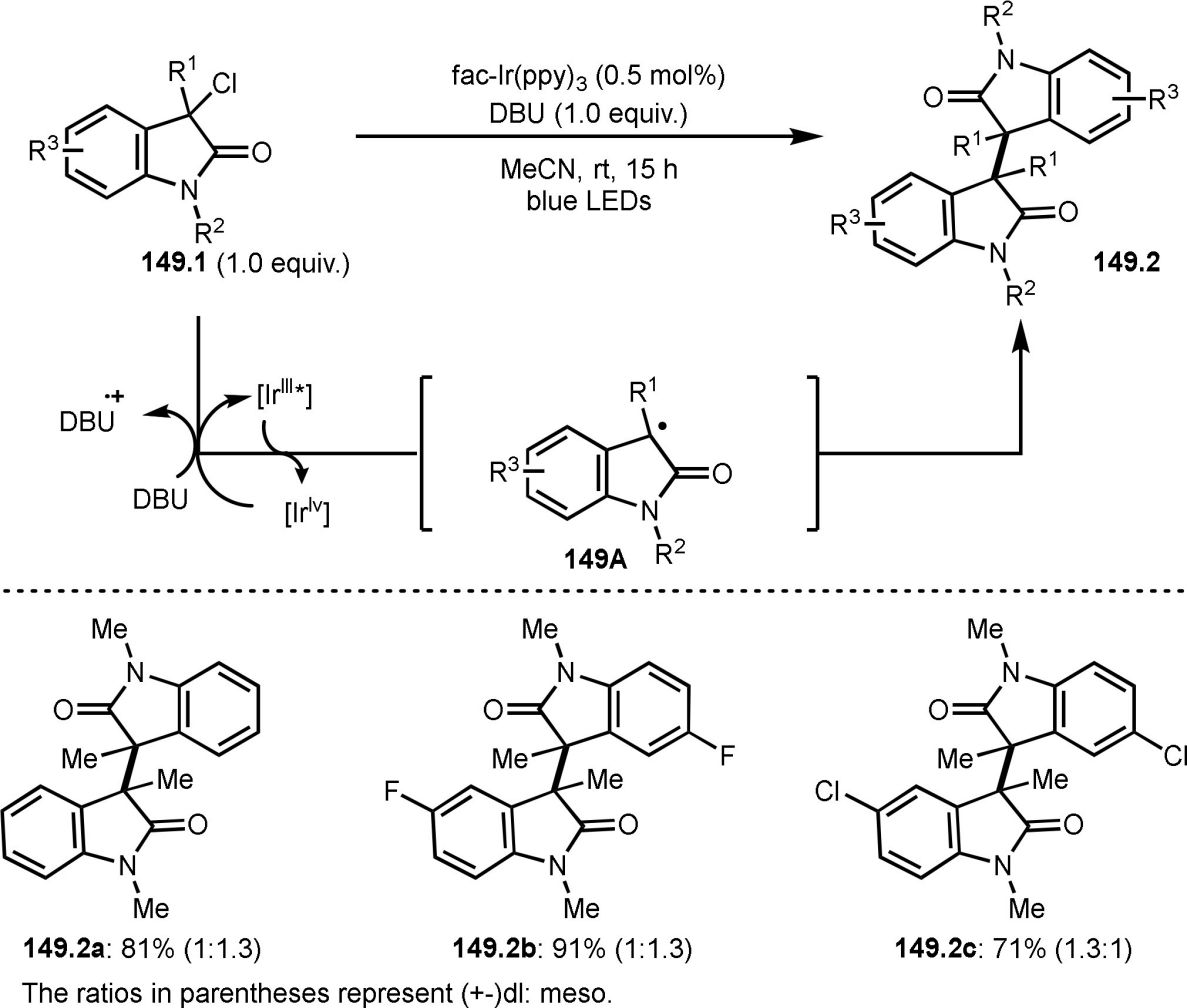
Homocoupling under Ir‐photocatalyst conditions.

Reactions of α‐halocarbonyl compounds without other substrates sometimes produce homocouplings of α‐*tert*‐alkyl radical species under radical conditions. But the yields of homocouplings are not so high due to their steric hinderance. To obtain practical chemical yield, careful optimization of both conditions and substrate structures are needed. Liu and Wu's group successfully obtained homocoupling products (**149.2 a**–**c**) from α‐chlorocarbonyl compounds (**149.1**) under Ir‐photocatalyst conditions (Scheme 149).^[308]^ This α‐halocarbonyl compounds have an aryl group at the carbonyl α‐position. Therefore, the corresponding bromide may be difficult to control radical generation. Photo‐excited Ir^III^ species reacted with **149.1** to provide α‐*tert*‐alkyl radical species (**149A**). This aryl‐stabilized radical has a lifetime sufficient to react with other radical species to produce homocoupling product (**149.2**).

### 4.8 C−H Functionalization

**Scheme 150:**
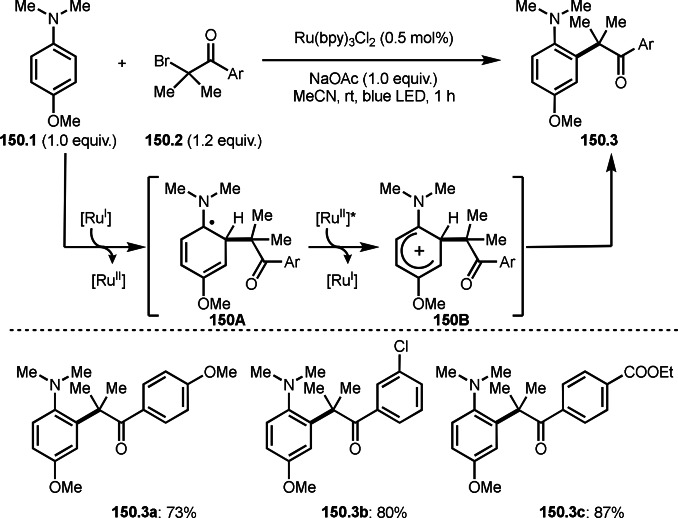
*ortho*‐C−H tert‐alkylation under Ru‐photo catalyst conditions.

In Section 3.9, transition metal‐catalyzed aromatic C−H tert‐alkylation was introduced, showing that radical tert‐alkylation at *meta*, *para*, and *remote* positions is possible with α‐halocarbonyl compounds. This type of C−H tert‐alkylation reaction is also possible using metal photocatalysts. Cheng and LI's group reported the reaction of *para*‐substituted anilines (**150.1**) with α‐bromocarbonyl compounds (**150.2**) to produce ortho‐*tert*‐alkylated anilines (**150.3 a**–**c**) under Ru‐photocatalyst conditions (Scheme 150).^[309]^ In situ generated α‐*tert*‐alkyl radical species added to **150.1** to give **150A**. The *tert*‐alkylation reaction proceeded only at the *ortho* position of **150.1** because the *para* position of **150.1** was blocked by a MeO substituent. **150.3** was obtained via re‐aromatization of a cation **150B** formed by oxidation with photo‐excited Ru^II^ species.

**Scheme 151:**
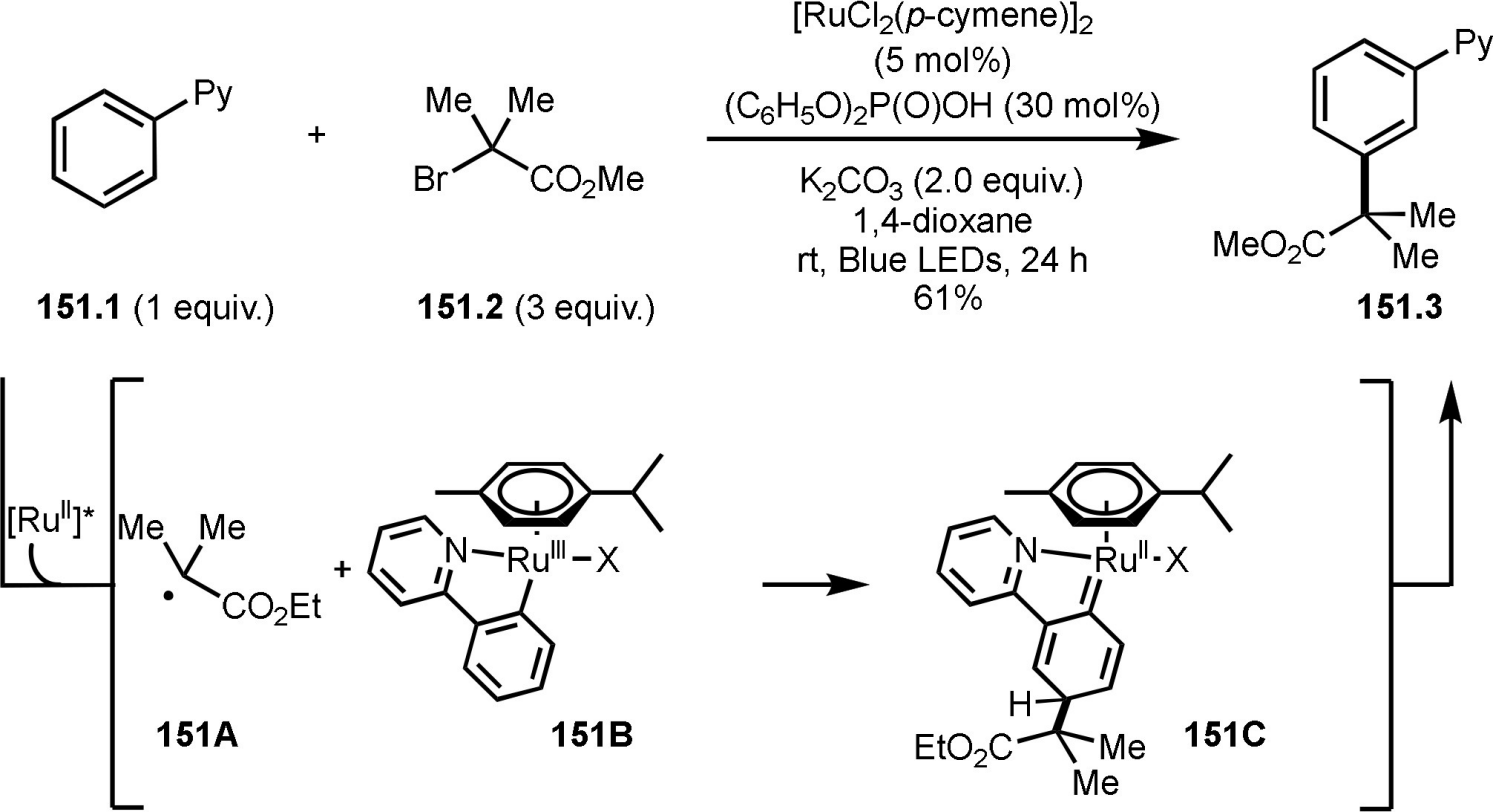
*meta*‐C−H tert‐alkylation under Ru‐photo catalyst conditions.

Ackermann's group and Greaney's group independently reported *meta*‐C−H *tert*‐alkylations of 2‐phenyl pyridine (**151.1**) with α‐bromocarbonyl compound (**151.2**) under Ru‐photocatalyst conditions (Scheme 151).[^310^, ^311^] Greaney's group employed KOAc as an additive, while Ackermann's group used phosphoric acid and potassium carbonate as additives. Ru species generated α‐*tert*‐alkyl radical species (**151A**) and ruthenacycle (**151B**) acting as a para‐selective radical acceptor giving **151C**. Finally, re‐aromatization reaction of **151C** produced *meta*‐tert‐alkylated arene (**151.3**). Scheme 96 required high temperatures for the same reaction. Under light irradiation, however, the reaction conditions were extremely mild.

**Scheme 152:**
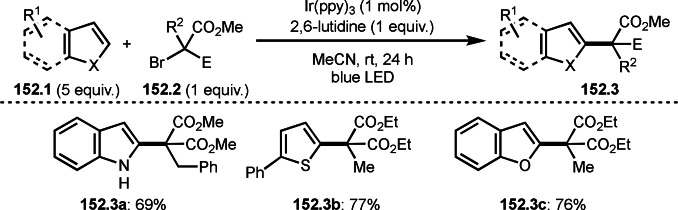
*tert*‐Alkylations of heteroarene C−H bonds under Ir‐photocatalyst conditions.

Under photo‐induced conditions, indole, thiophen, and furan derivatives can be used for C−H *tert*‐alkylations. Stephenson's group reported the reaction of heteroarenes (**152.1**) with α‐bromocarbonyl compound (**152.2**) to produce *tert*‐alkylated heteroarenes (**152.3 a**–**c**) under Ir‐photocatalyst conditions (Scheme 152).^[312]^ Electron‐rich heteroarenes are suitable for the reaction with electron‐deficient α‐*tert*‐alkyl radical species. Without light irradiation, high temperatures were required as described in the previous section.

## 5 Photoreaction by an Organocatalyst

The α‐bromocarbonyl compound receives one electron and produces an α‐*tert*‐alkyl radical species. Metal complexes and metal photocatalysts are excellent one‐electron transfer agents, but there are also organocatalysts that are nonmetallic electron sources. In this section, various organocatalyst systems for reactions with α‐bromocarbonyl compounds are introduced.

### 5.1 Addition Reaction

**Scheme 153:**
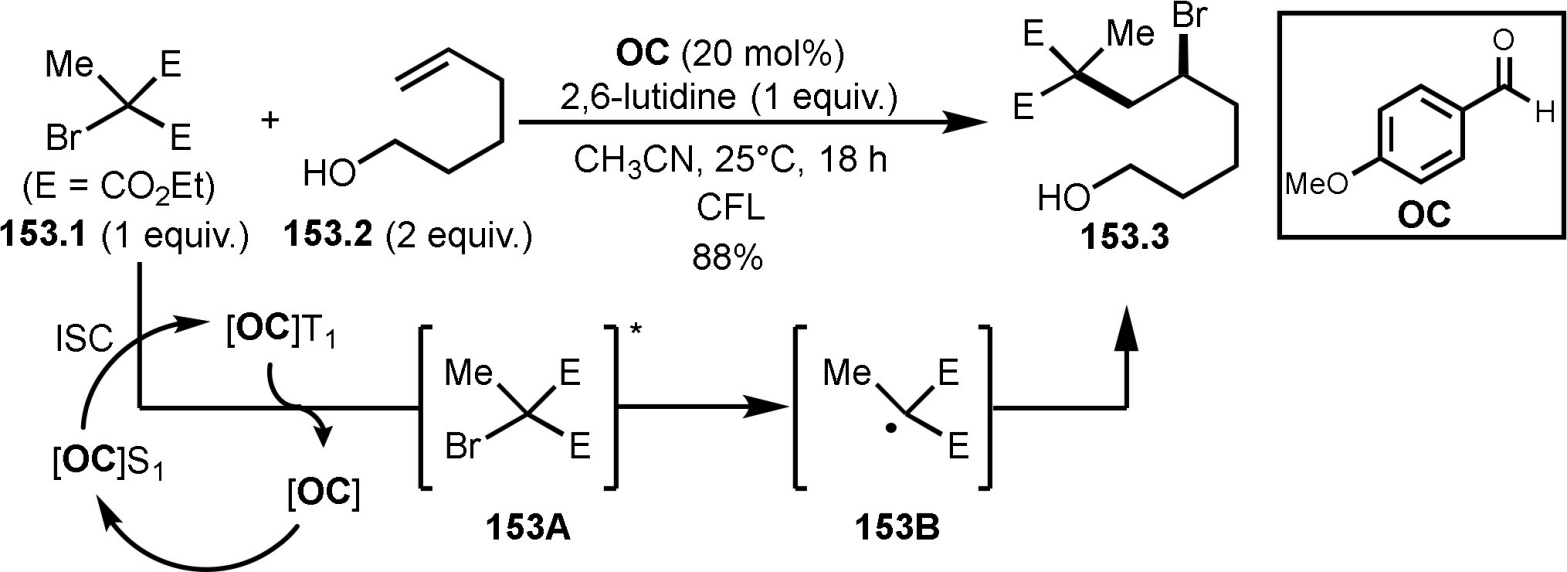
*p*‐Anisaldehyde catalyzed ATRA.

There are many reports on ATRA reactions with α‐bromocarbonyl compounds in the presence of a various radical initiators. Melchiorre reported that an aldehyde (OC) is an effective photocatalyst for ATRA of α‐bromocarbonyl compound (**153.1**) and homoallylic alcohol (**153.2**) (Scheme 153).^[313]^ Photo‐excited OC(S_1_) generated OC(T_3_) via ISC (intersystem crossing). According to their results, a triplet energy of OC (300 kJ/mol) can dissociate carbon‐halogen bond (260–300 kJ/mol) of **153A** to produce radical species (**153B**). Finally, ATRA product (**153.3**) was obtained. This reaction is quite air sensitive. Because oxygen acted as a quencher for photo‐excited OC. The role of the base was neutralization in this reaction medium. In this reaction, a small amount of HBr was formed, which acidifies the reaction. This affected the overall reactivity. Thus, a base was needed to avoid acidification.

**Scheme 154:**
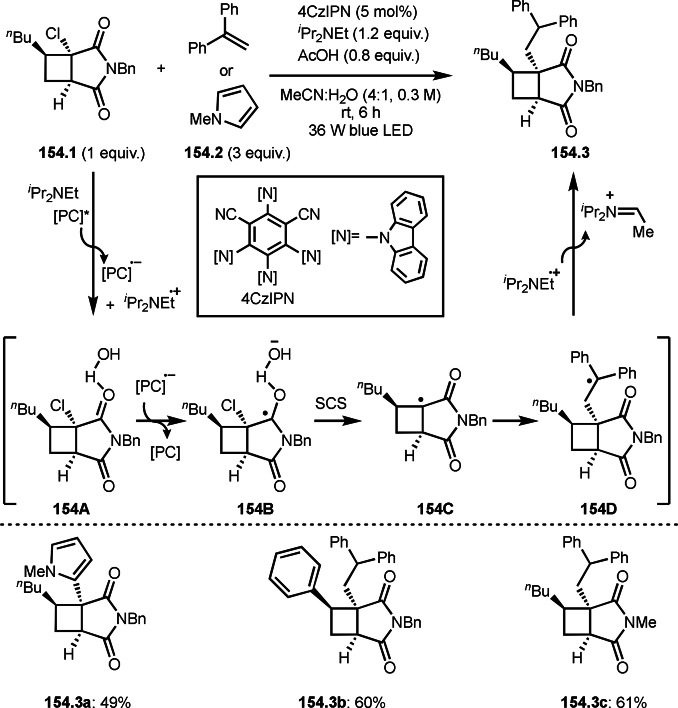
The addition reaction catalyzed by 4CzIPN.

Noble, Booker‐Milburn and Aggarwal's group reported the synthesis of densely functionalized cyclobutanes from the reaction of haloimides (**154.1**) with **154.2** in the presence of 4CzIPN (PC) as an organocatalyst (Scheme 154).^[314]^ 4CzIPN, an organic light‐emitting diode, is useful photo‐sensitizer, which was developed by Adachi's group.^[315]^ PC anion radical generated from the reaction of PC with sacrificial amine reductant. The resulting anion radical of PC underwent SET with hydrogen‐bonded complex (**154A**) to generate the protonated ketyl radical **154B**. The formation of **154A** facilitates single‐electron reduction of **154.1** via a proton‐coupled electron transfer (PCET). **154B** then generated α‐*tert*‐alkyl radical species (**154C**) via spin center shift (SCS), which added to **154.2** to produce **154.3**. In this reaction, olefins and electron‐rich arenes were good substrates. When reacted with electron‐rich arenes, addition reactions followed by re‐aromatization produced *tert*‐alkyl‐substituted arenes such as **154.3 a**.

**Scheme 155:**
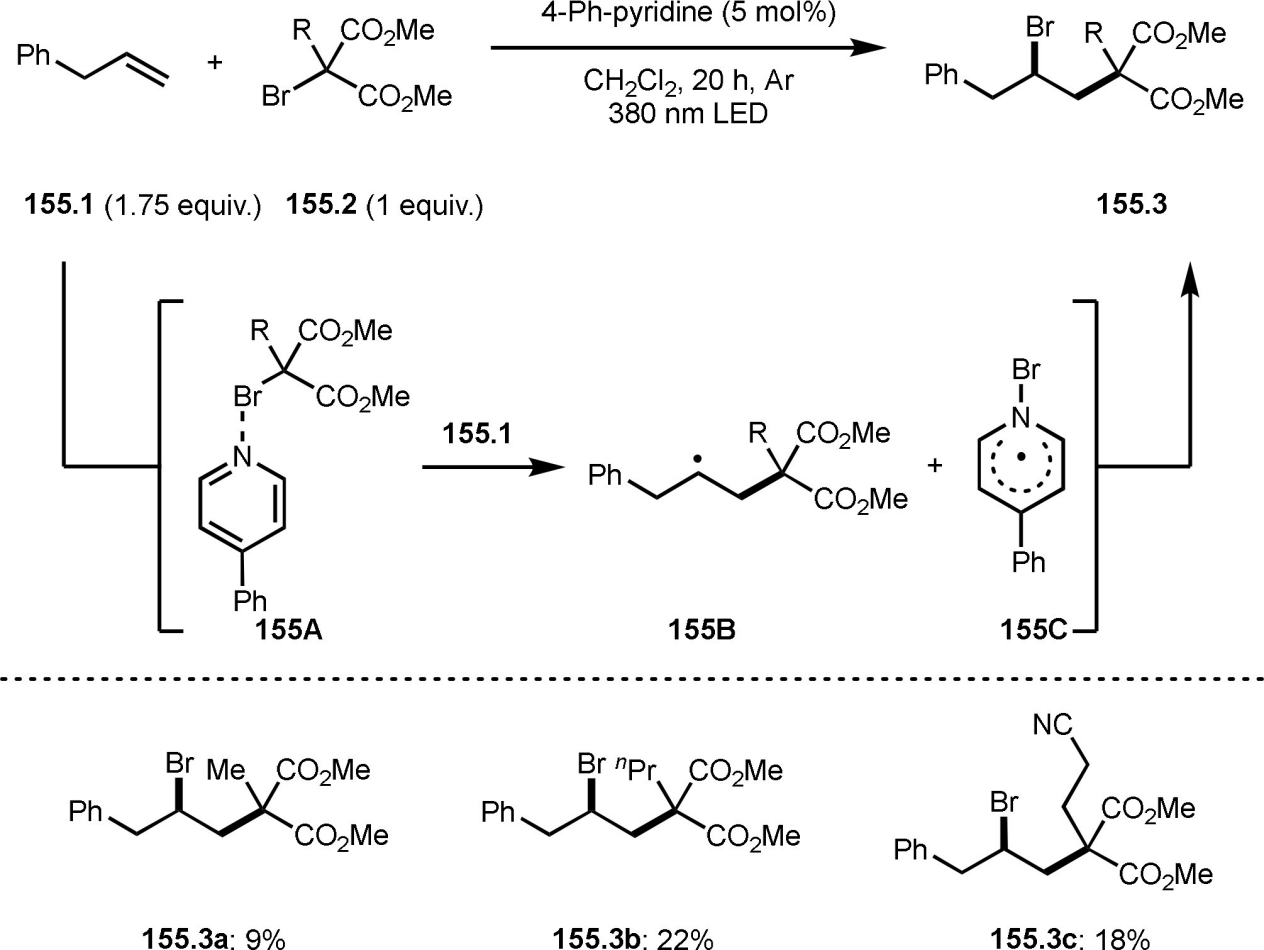
ATRA catalyzed by 4‐Ph‐pyridine.

Yamaguchi and Itoh's group discovered that 4‐Ph‐pyridine was a good catalyst for ATRA reactions of allyl benzene (**155.1**) with α‐bromocarbonyl compounds (**155.2**) (Scheme 155).^[316]^ In this case, an organocatalyst, 4‐Ph‐pyridine, was not directly excited. The reaction was initiated by photoexcitation of the halogen‐bonded complex (**155A**), causing cleavage of the C−Br bond to produce an α‐*tert*‐alkyl radical species. Activation of the substrate was accomplished by the formation of Br−N complexes, which are charge‐transfer complexes. The resulting α‐tert‐alkyl radical species added to **155.1** to give **155B** and **155C**. Radical **155B** reacted with **155C** to afford ATRA product (**155.3**).

### 5.2 Three‐Component Reaction

Three‐component reactions are among the most difficult organic reactions because the reactivity of the substrates must be precisely controlled. As shown in Section 3.2 on metal‐catalyzed reactions, several chemical species can be controlled in the presence of α‐bromocarbonyl compounds, and a variety of structures with *tert*‐alkyl moieties have been successfully synthesized. On the other hand, it is difficult to control this reaction under organophotocatalytic conditions. In fact, only two cases have been reported so far.

**Scheme 156:**
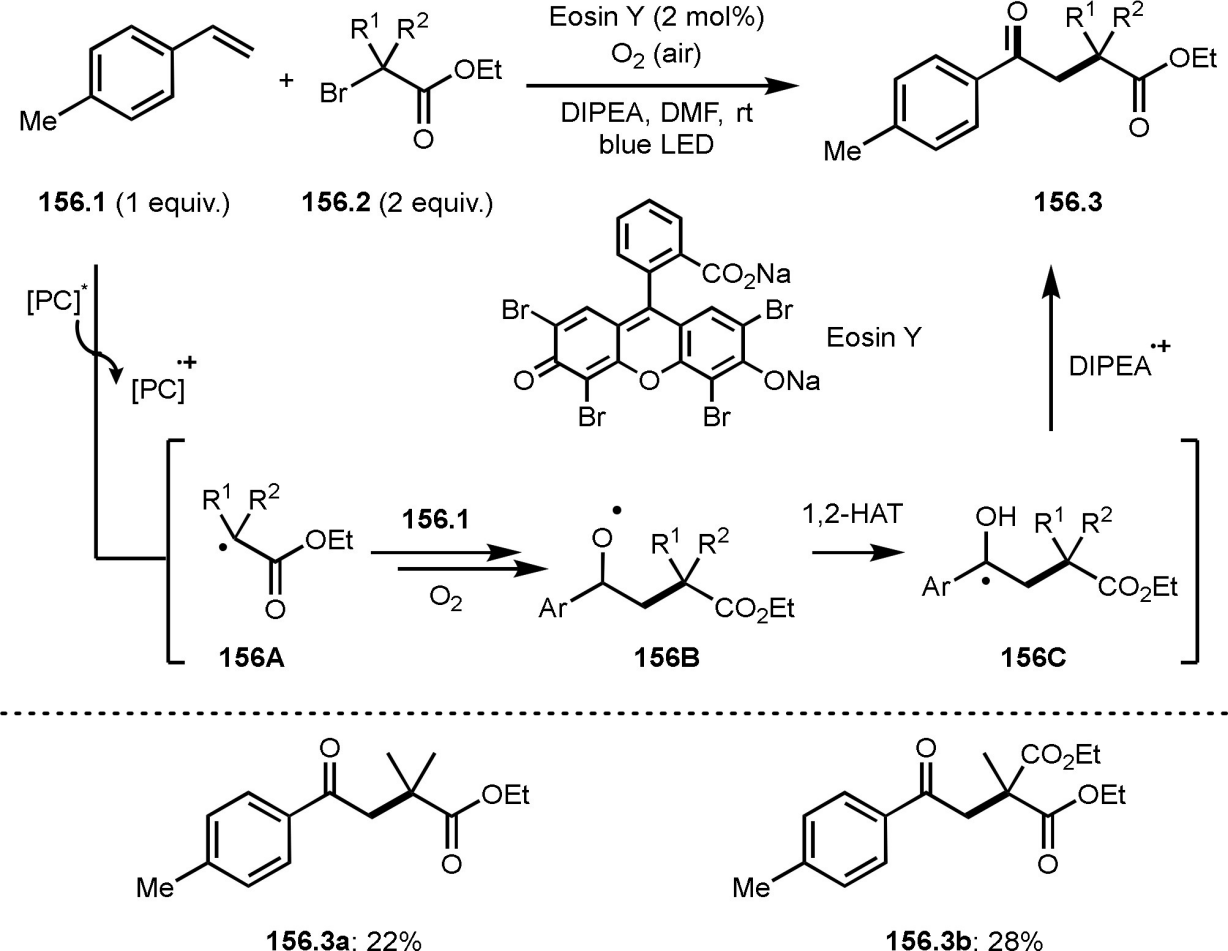
Three‐component reaction catalyzed by Eosin Y.

Maity's group reported the reaction of *p*‐methylstyrene (**156.1**), α‐bromocarbonyl compounds (**156.2**) and oxygen to produce three‐component coupled products (**156.3 a**, and **b**) in the presence of Eosin Y (Scheme 156).^[317]^ Under visible light irradiation, Eosin Y was excited. The resulting excited Eosin Y (E_EY_ ⋅ ^+^_/EY*_=−1.11 V vs SCE in CH_3_CN) reduced **156.2** (E^*red*^=−0.80 V vs SCE in CH_3_CN) to give **156A**. **156A** then reacted with **156.1** and oxygen to form an oxy radical intermediate (**156B**), which underwent 1,2‐HAT to give **156C**. **156C** was oxidized by a cation radical DIPEA to produce **156.3**. Cation radical DIPEA was produced via oxidation of DIPEA with a cation radical Eosin Y. They carried out some control experiments, and revealed that the excited Eosin Y was quenched by **156.2** and DIPEA. Light on/off experiments were also conducted to confirm the need for continuous light exposure in this reaction.

**Scheme 157:**
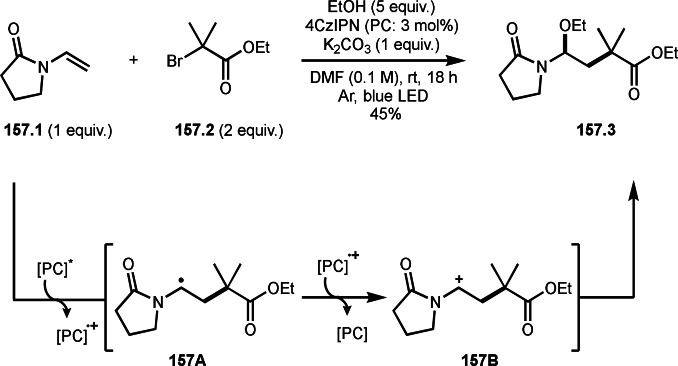
Three‐component reaction catalyzed by 4CzIPN.

Molander's group reported the reaction of enamide (**157.1**), α‐bromocarbonyl compound (**157.2**) and EtOH to produce three‐component coupled product (**157.3**) (Scheme 157).^[318]^ Although the reaction efficiency was not high, **157.3** was obtained in 45 % yield in the presence of the organophotocatalyst 4CzIPN. Since the main focus of this reaction was difluoroalkylation, there was only one reaction with the α‐bromocarbonyl compound. In this reaction, the excited 4CzIPN reduced **157.2** to give an α‐*tert*‐alkyl radical species, which added to **157.1** to give **157A**. Next, a cation species (**157B**) was generated from the oxidation of **157A** with a cation radical of 4CzIPN. Finally, EtOH reacted with **157B** to produce **157.3**.

### 5.3 Intermolecular Cyclization

**Scheme 158:**
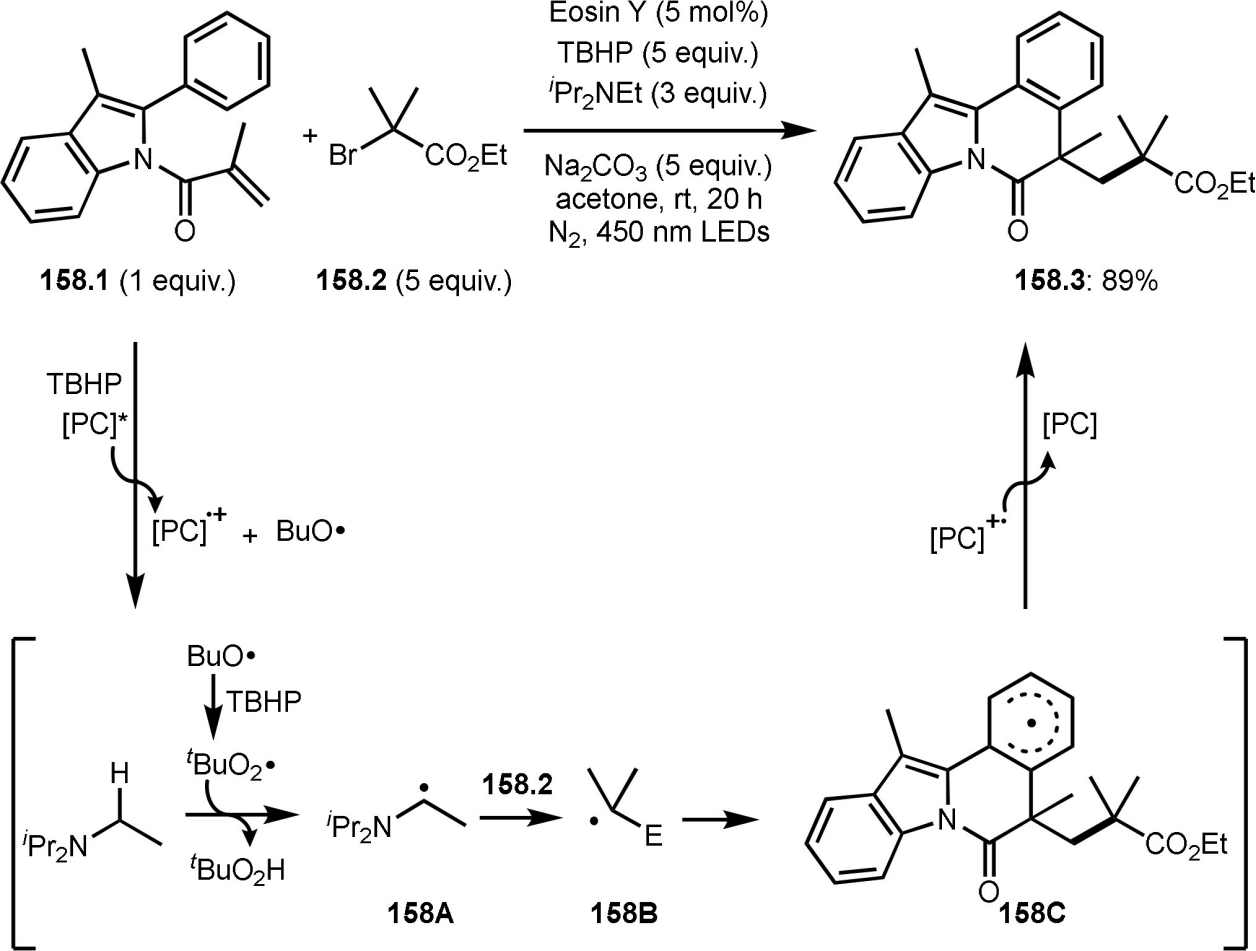
Intermolecular cyclization by Eosin Y.

There are three examples for intermolecular cyclization reaction in organophotocatalyst systems. Yang and Xia's group employed Eosin Y as an organophotocatalyst for the synthesis of multicyclic compound (**158.3**) from the reaction of an indole derivative (**158.1**) with α‐bromocarbonyl ester (**158.2**) under photoirradiation (Scheme 158).^[319]^ Photo‐excited Eosin Y reacted with TBHP to generate oxy radical (^t^BuO⋅), in which ^t^BuO⋅ reacted with TBHP to generate per‐oxy radical (^t^BuO_2_⋅). The resulting per‐oxy radical reacted with ^*i*^Pr_2_NEt to generate **158A**. In this reaction, α‐*tert*‐alkyl radical species (**158B**) was not directly generated from the reaction of Eosin Y with **158.2**. Instead, **158A** captured the bromine atom of **158.2** to generate α‐*tert*‐alkyl radical species (**158B**). **158B** then added to acrylamide moiety of **158.1** to give **158C**. Finally, **158.3** was produced via oxidation with a cation radical PC/re‐aromatization of **158C**.

**Scheme 159:**
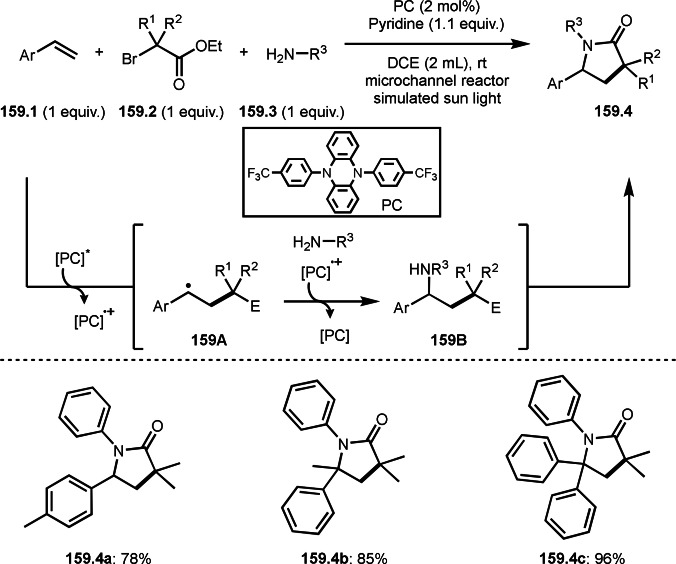
Three‐component intermolecular cyclization by an organophotocatalyst.

Yang, Fang and Guo's group has developed an organophotocatalytic synthesis of pyrrolidin‐2‐ones (**159.4**) from the reaction of styrenes (**159.1**), α‐bromocarbonyl esters (**159.2**) and amines (**159.3**) (Scheme 159).^[320]^ This reaction is three‐component cyclization reaction. They successfully controlled the reactivity of α‐*tert*‐alkyl radical under optimized conditions. Under simulated sunlight irradiation, α‐tert‐alkyl radicals were formed from the reaction of **159.2** and photoexcited PC. Next, **159A** was formed via the addition of an α‐tert‐alkyl radical to **159.1**. Finally, **159A** was oxidized with cation radical PC followed by amine addition/intramolecular condensation to give **159.4**.

**Scheme 160:**
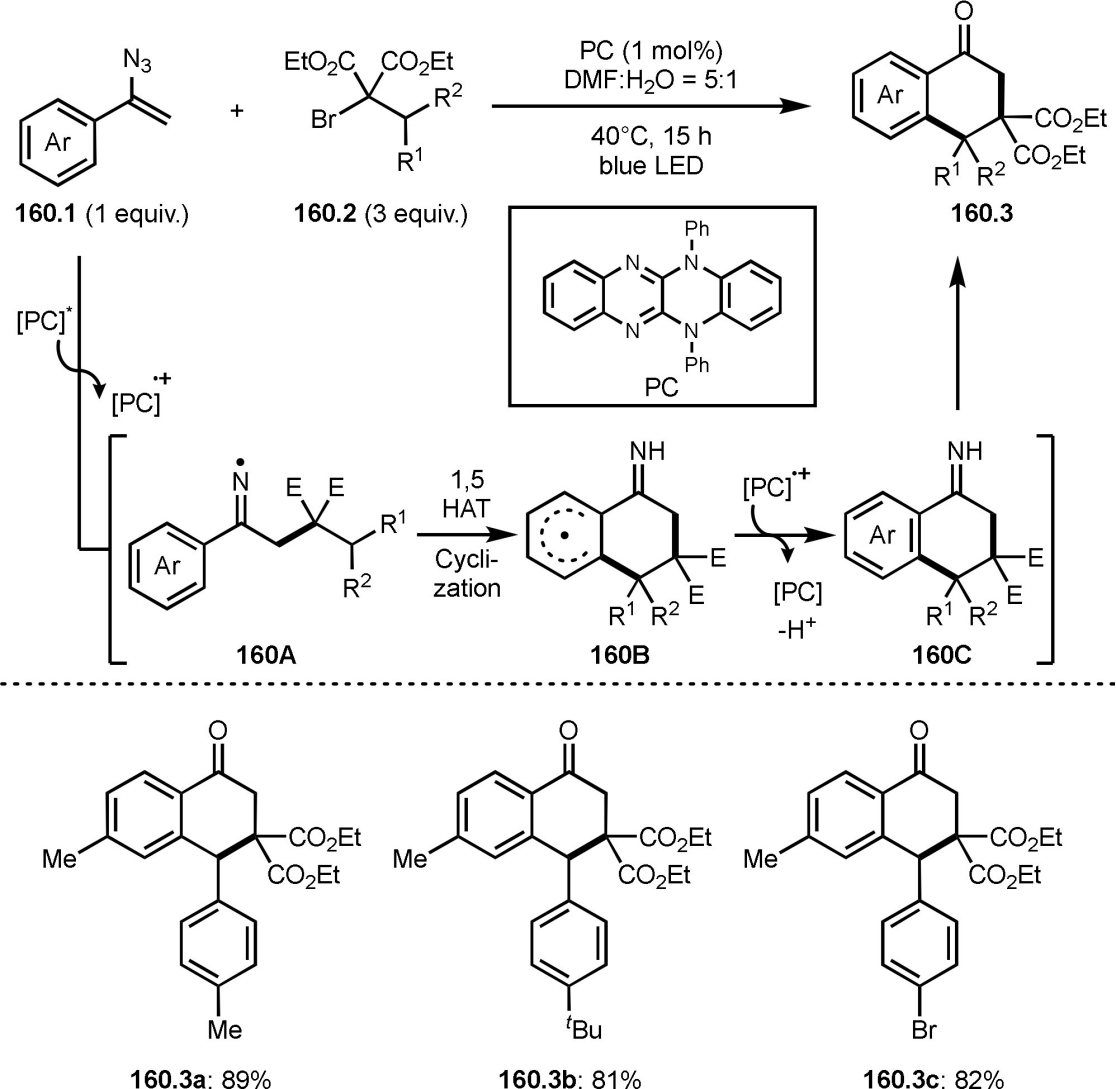
Intermolecular cyclizations of vinyl azides under organophotocatalyst conditions.

Hu and Xu's group employed vinyl azide for intermolecular cyclizations (Scheme 160).^[321]^ The reaction of vinyl azides (**160.1**) and α‐bromocarbonyl compounds (**160.2**) underwent radical addition/C−H cyclization to produce tetralone skeletons (**160.3**) in the presence of organophotocatalyst. First, α‐*tert*‐alkyl radicals were formed from the reaction of **160.2** with photoexcited PC, which added to **160.1** to give **160A** after nitrogen generation. **160A** underwent 1,5‐HAT followed by cyclization to form **160B**. Imine **160C** was obtained by oxidation of **160B** with cation radical PC followed by re‐aromatization. **160.3** was produced after hydrolysis of **160C**.

### 5.4 Cross‐Coupling Reaction

Cross‐coupling reactions are generally carried out with a transition metal catalyst, such Pd. Because oxidative addition or transmetallation is a key reaction step, which are possible through a transition metal action. On the other hand, organophotocatalyst system has accomplished Suzuki‐Miyaura and Heck‐like ATRS reactions through radical processes.

**Scheme 161:**
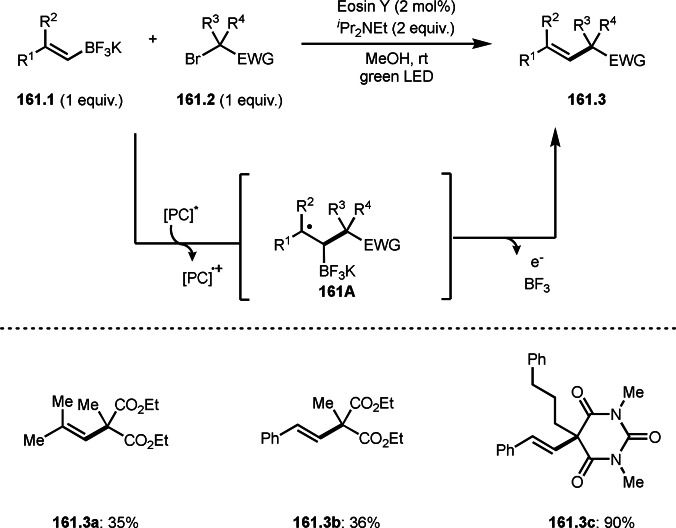
Suzuki‐Miyaura type couplings catalyzed by Eosin Y.

Sheikh and Leonori's group discovered Suzuki‐Miyaura type cross‐couplings between 1‐alkenyl trifluoroborates (**161.1**) and α‐bromocarbonyl compounds (**161.2**) in the presence of Eosin Y (Scheme 161).^[322]^ Nishikata's group also performed Suzuki‐Miyaura coupling using Cu catalyst (Scheme 79), but the mechanism is quite different from Leonori's reactions. In this case, the α‐*tert*‐alkyl radical generated from the reaction of **161.2** with photoexcited PC added to **161.1** to give **161A**. Interestingly, α‐tert‐alkyl radical species are difficult to add to internal olefins. But various 1‐alkenyl borate possessing internal C−C double bonds can be applicable to this reaction. Finally, **161.3** was produced by oxidizing **161A** followed by deboronation.

**Scheme 162:**
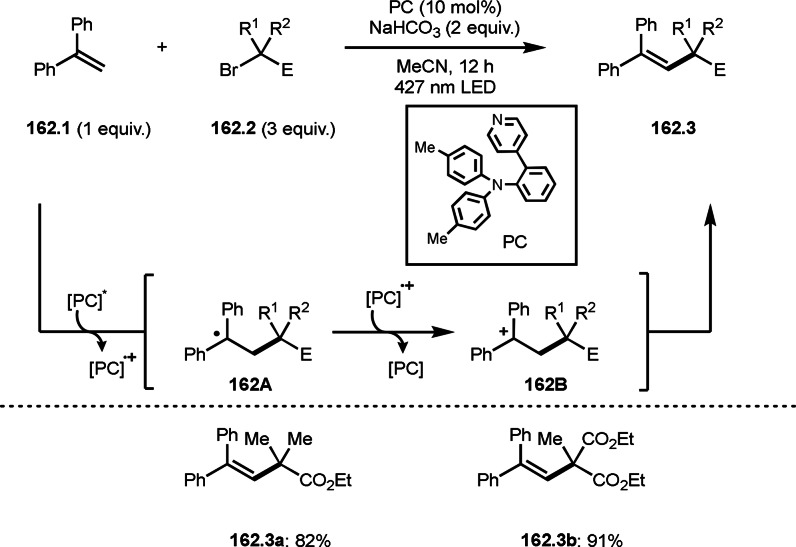
ATRS catalyzed by organophotocatalyst.

Nanjo and Takemoto's group reported the reaction of alkenes (**162.1**) and α‐bromocarbonyl compounds (**162.2**) to produce *tert*‐alkylated alkenes (**162.3 a**,**b**) in the presence of pyridine‐based organophotocatalyst (Scheme 162).^[323]^ Although α‐*tert*‐alkyl radical species can be generated from the reaction of **162.2** and photo‐excited PC, they also suggested the generation through halogen bonding between Br moiety of **162.2** and pyridine moiety of PC. But this PC has enough redox potential (E(PC^•+^/PC*)=−2.06 V vs Ag/Ag^+^)) to reduce α‐bromocarbonyl compounds. Probably this halogen bonding is only applicable for the reaction with simple alkyl bromides, such as *tert*‐butyl bromide. Overall reaction is basically followed the ATRS mechanism.

### 5.5 C−H Functionalization

**Scheme 163:**
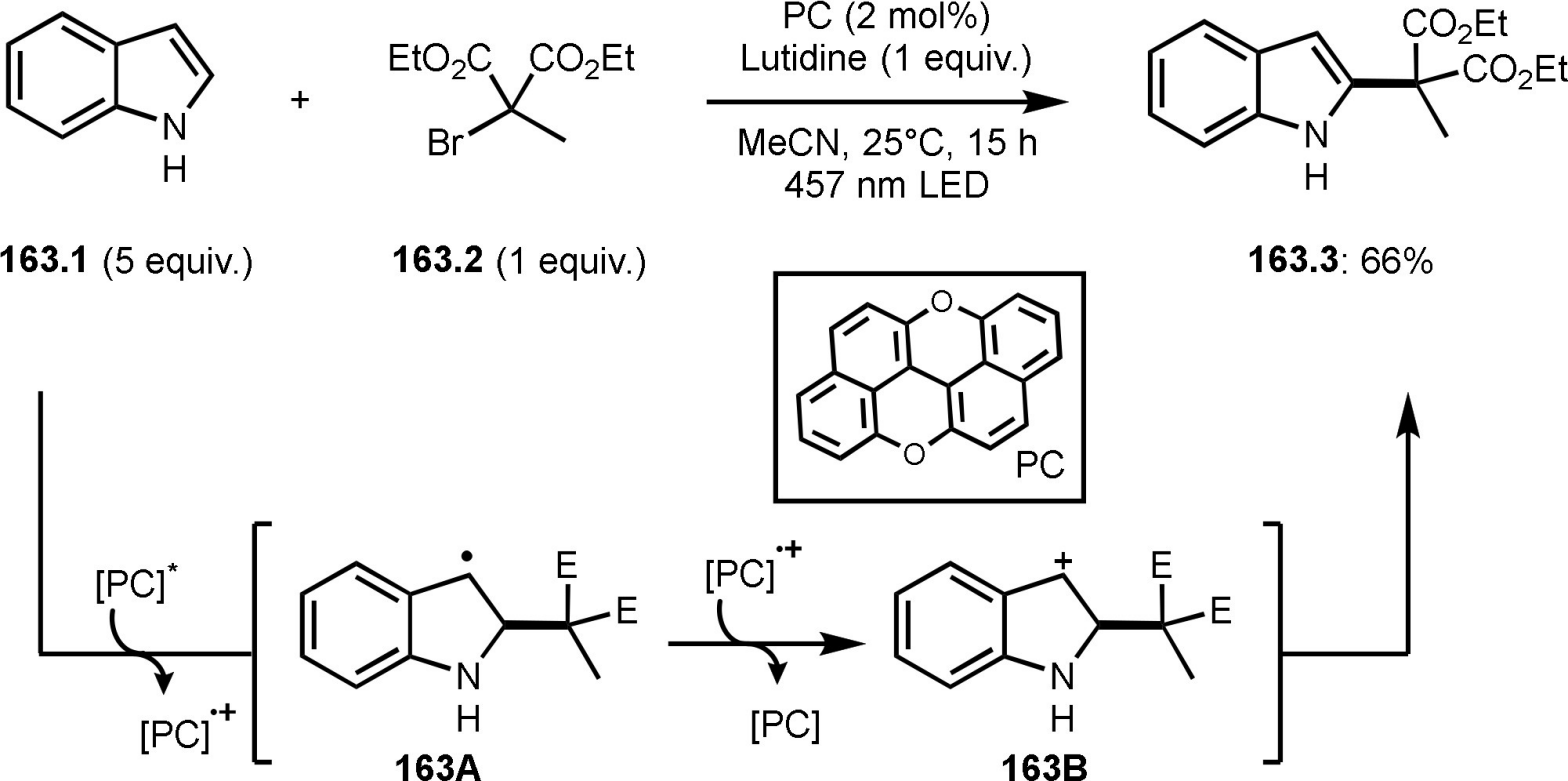
C−H *tert*‐alkylation with a PXX catalyst.

Although C−H bonds of heteroaromatics are relatively reactive compared with simple aromatic C−H bonds, Davidson and Bonifazi discovered interesting organophotocatalyzed C−H *tert*‐alkylations of indole (**163.1**) and bromomalonate (**163.2**) (Scheme 163).^[324]^ They used *peri*‐xanthenoxanthene (PXX) as a catalyst to obtain 2‐*tert*‐alkylated indoles (**163.3**) in 66 % yield under visible light irradiation.

## 6 Organocatalyzed Reaction

Transition metal catalysts and photocatalysts are effective methods for reducing α‐bromocarbonyl compounds by a single electron transfer process to produce α‐*tert*‐alkyl radical species. On the other hand, reaction systems that can sufficiently reduce α‐bromocarbonyl compounds using only organocatalysts without photo‐irradiation have been reported. Here several organocatalytic radical reactions with α‐bromocarbonyl compounds that have been reported recently are described.

### 6.1 Three‐Component Reaction

**Scheme 164:**
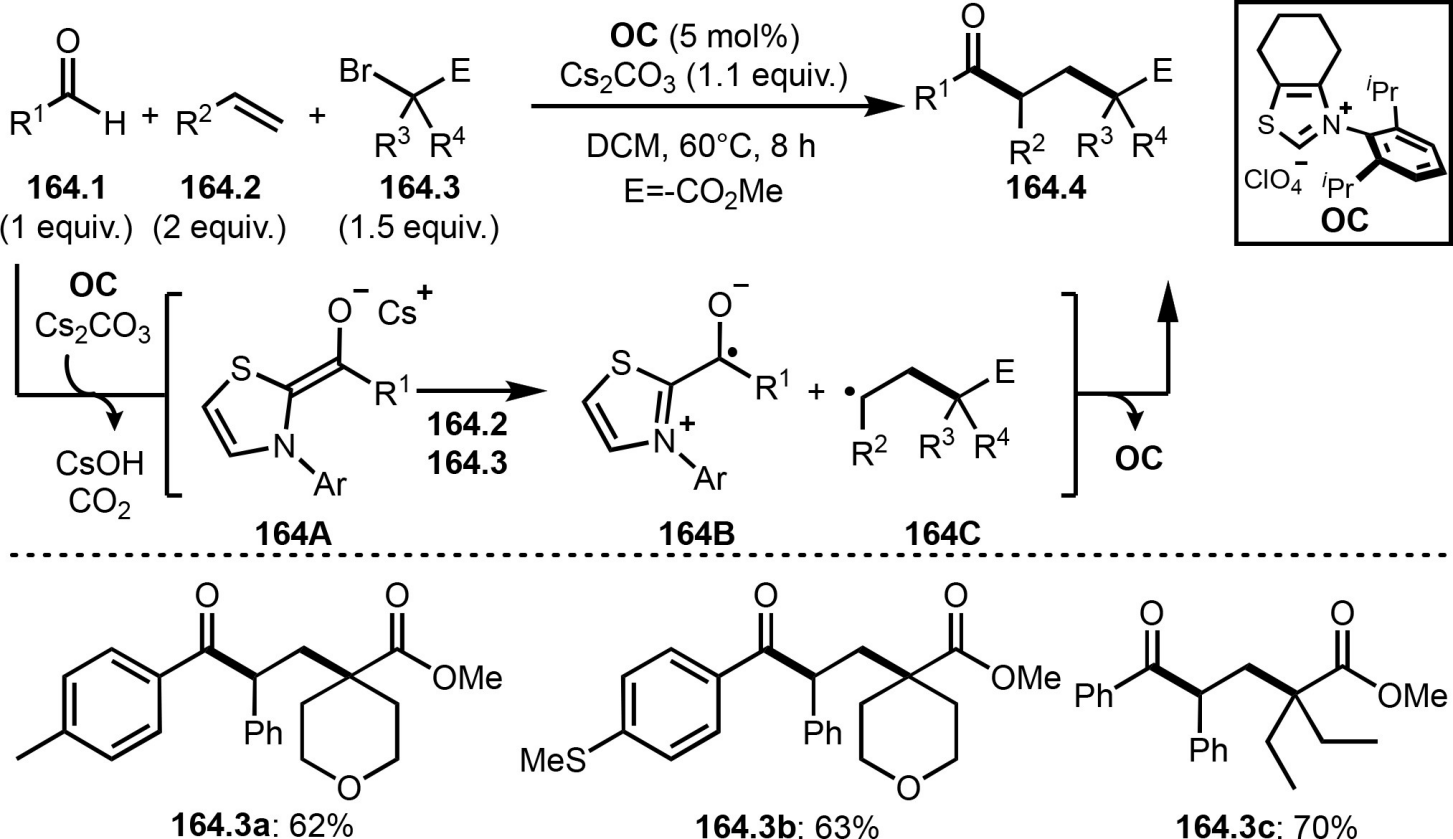
Three‐component reaction with olefins catalyzed by NHC.

Nagao and Ohmiya's group discovered N‐heterocyclic carbenes (NHC) were effective to generate α‐*tert*‐alkyl radical species from α‐bromocarbonyl compounds (**164.3**) (Scheme 164).^[325]^ The reaction of aldehydes (**164.1**), styrenes (**164.2**) and **164.3** in the presence of NHC catalyst (**OC**) gave δ‐keto esters (**164.4 a**–**c**). NHC first reacted with aldehyde (**164.1**) to produce a Bleslow intermediate (**164A**, E^0^_ox_=‐0.95 to −0.97 vs SCE) to reduce **164.3** and provide **164.4** and α‐*tert*‐alkyl radical species. Next, the addition of α‐*tert*‐alkyl radical species to **164.2** gave **164C**. Finally, the radical‐radical coupling between **164B** and **164C** followed elimination of **OC** by produced **164.4**. This NHC‐catalyzed method can be used to easily replace the hydrogen of aldehydes with an alkyl group. In this case, photo‐irradiation or metal catalyst was not required but high temperatures were necessary for smooth SET.

**Scheme 165:**
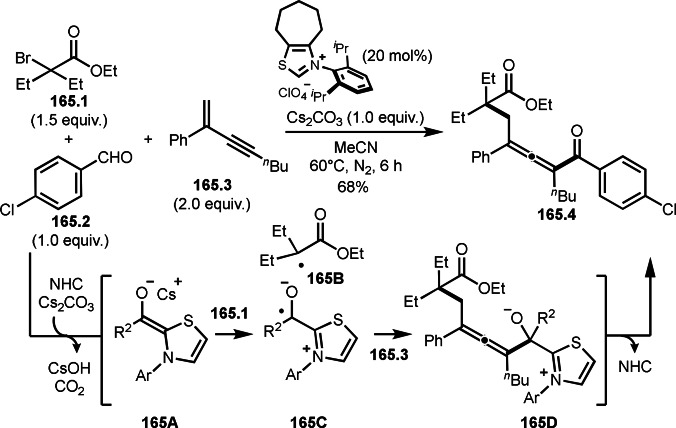
Three‐component reaction with 1,3‐enyne catalyzed by NHC.

Feng and Du's group extended Ohmiya's reaction to synthesize *tert*‐alkylated allene (**165.4**) in the presence of an NHC catalyst (Scheme 165).^[326]^ The reaction of α‐bromocarbonyl compound (**165.1**), aldehyde (**165.2**) and 1,3‐enye (**165.3**) produced **165.4**. Previous Ohmiya's reaction shown in Scheme 164 employed NHC possessing a six‐membered ring, whereas they used NHC possessing a seven‐membered ring, which was effective for the reaction with 1,3‐enynes. The proposed reaction mechanism begins with the formation of α‐*tert*‐alkyl radical species (**165B**) from the Bleslow intermediate (**165A**), similar to that of Ohmiya's report. Both **165B** and **165C** added to **164.2**, in which 1,4‐adidition occurred selectively. As the result, **165D** was formed. Finally, the NHC elimination from **165D** produced **165.4**.

### 6.2 Intramolecular Cyclization

**Scheme 166:**
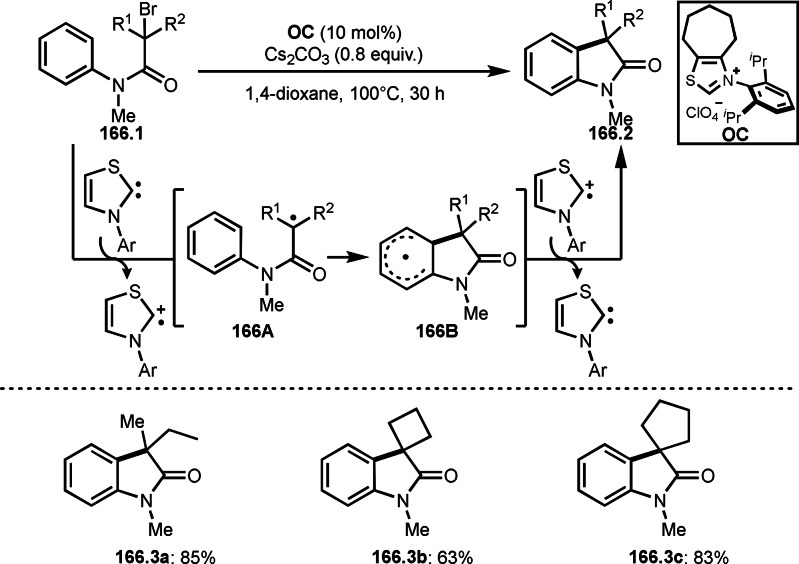
Intramolecular C−H cyclization by NHC catalyst.

**Scheme 167:**
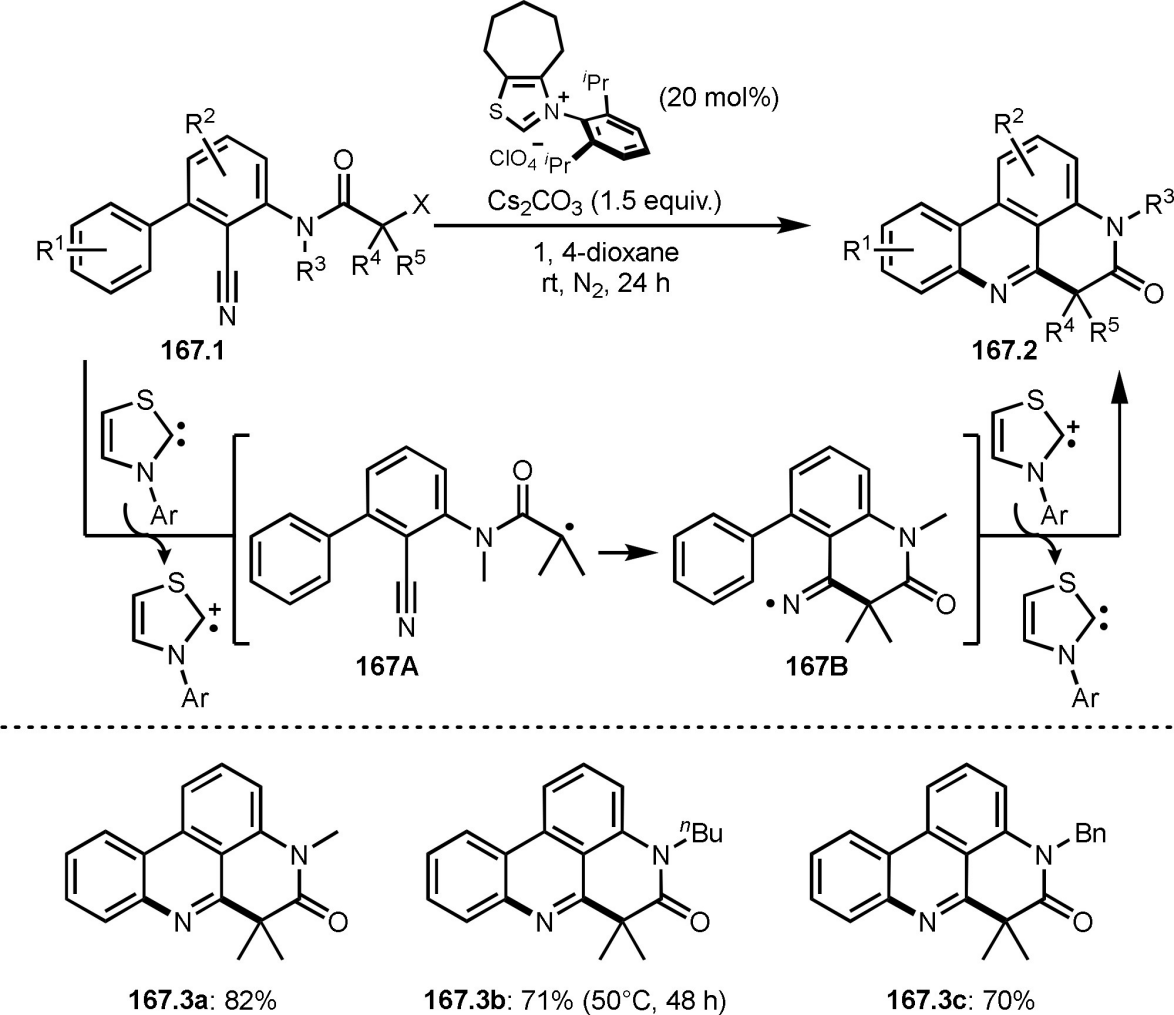
Intramolecular multi‐cyclization to give by NHC catalyst.

Intramolecular cyclizations, especially aromatic C−H cyclizations, are possible under organocatalytic conditions, Wang's group reported interesting intramolecular C−H cyclization to synthesize complex cyclic compounds (Scheme 166, ^[327]^ and Scheme 167, ^[328]^). Both reactions utilized NHC possessing a seven‐membered ring to achieve the cyclizations. The reaction of **166.1** in the presence of NHC (**OC**) produced oxindole derivatives (**166.2**). In this reaction, the NHC functioned as a single electron source. NHC underwent SET with **166.1** to produce an NHC radical cation and **166A**. **166A** was immediately cyclized to give **166B**, which was oxidized with an NHC radical cation, followed by re‐aromatization to give **166.2**. Under similar conditions, they employed **167.1** as a substrate to produce multi‐cyclization products via NHC‐catalyzed intramolecular C−H cyclization (**167.2**). This cyclization involved radical addition to a CN triple bond to give an iminyl radical (**167B**). The reaction control of this important intermediate led to a complex cyclization with α‐*tert*‐alkyl radical species.

**Scheme 168:**
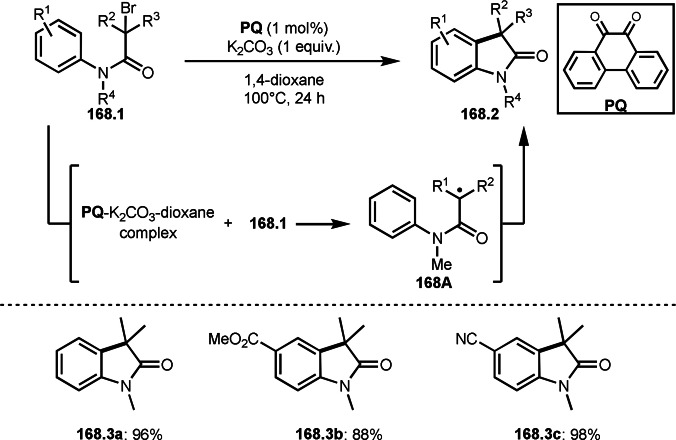
Intramolecular multi‐cyclization by PQ catalyst.

NHC is not the only effective way to use organocatalysts for alkylation without photoirradiation. Recently, Wang and Lei's group reported 9,10‐phenanthrenequinone (PQ) catalyzed intramolecular C−H cyclizations to produce oxindole derivatives (**168.2**) (Scheme 168).^[329]^ They proposed the formation of PQ−K_2_CO_3_‐dioxane complex as a source of single electron, and this key complex underwent SET with **168.1** to produce **168A**. As the result, **168.2** was produced from the intramolecular cyclization of **168A** followed by oxidation/re‐aromatization. In this case, high temperatures were necessary for smooth SET.

### 6.3 Intermolecular Cyclization

**Scheme 169:**
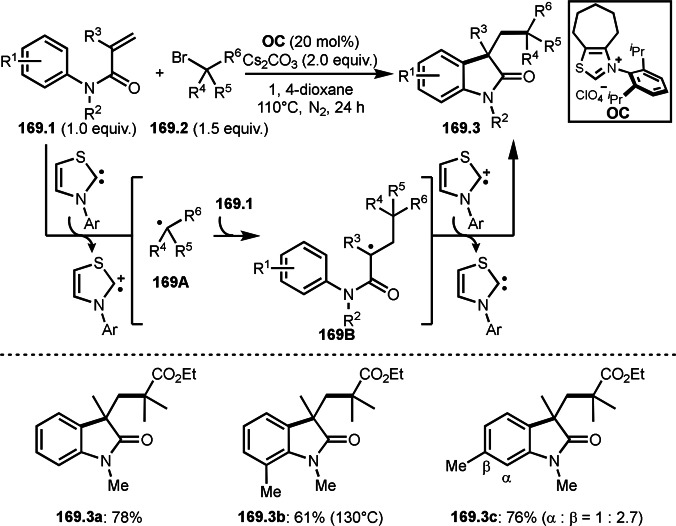
Intermolecular cyclization with α‐bromocarbonyl compound by NHC catalyst.

NHC catalysts are powerful enough to carry out intermolecular cyclizations with α‐bromocarbonyl compound. Sun, Liu and Wang's group reported intramolecular cyclization of acrylamide (**169.1**) and α‐bromocarbonyl compounds (**169.2**) in the presence of NHC catalyst (**OC**) (Scheme 169).^[330]^ Again, a seven‐membered ring NHC was used to conduct smooth α‐*tert*‐alkyl radical generation. The resulting **169A** added to **169.1** to form **169B**. **169B** then underwent intramolecular C−H cyclization followed by oxidation/re‐aromatization to produce **169.3**.

**Scheme 170:**
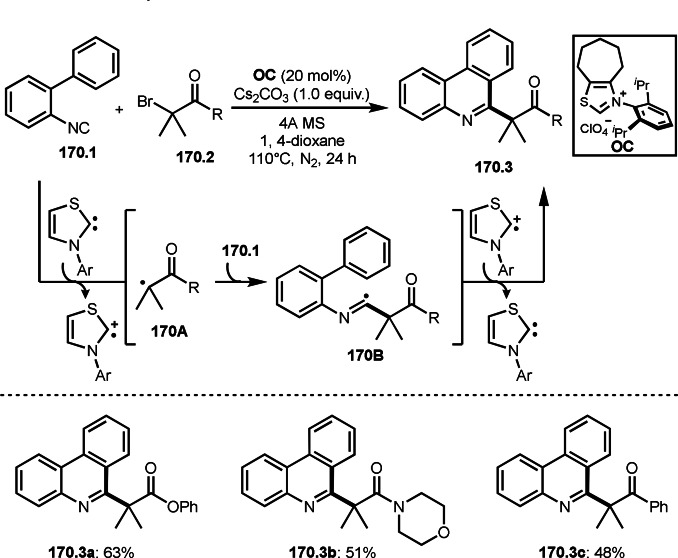
Intermolecular cyclization with isonitriles by NHC catalyst.

**Scheme 171:**
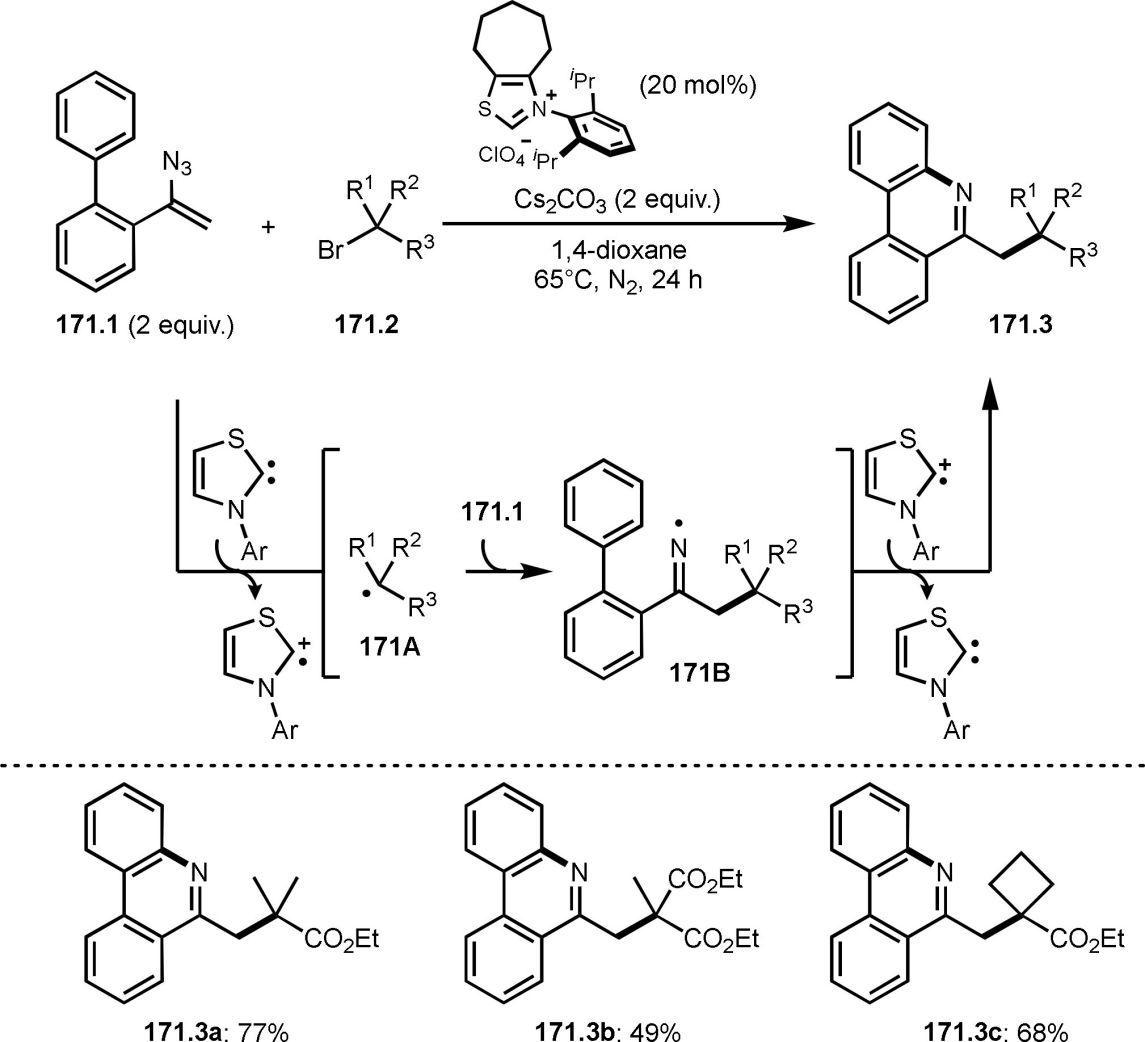
Intermolecular cyclization with vinyl azides by NHC catalyst.

Wang's group also reported the synthesis of heterocycles via intermolecular cyclization catalyzed by NHC possessing a seven‐membered ring (Scheme 170, ^[331]^ and Scheme 171, ^[332]^). The α‐*tert*‐alkyl radical species from α‐bromocarbonyl compounds react with the carbon‐carbon multiple bond and a subsequent cyclization reaction proceed. In these cases, they used isonitrile and C−C double bonds possessing N_3_ moiety as radical acceptors. Wang's group utilized their NHC radical chemistry to carry out intermolecular cyclizations. In the case of Scheme 170, NHC generated α‐*tert*‐alkyl radical (**170A**) from **170.2**, which added to **170.1** to give iminyl radical **170B**. This key intermediate underwent cyclization/oxidation/re‐aromatization to form **170.3**. Vinyl azides were also possible to undergo intermolecular cyclization with various α‐bromocarbonyl compounds (**171.2**) to produce phenanthridine derivatives via similar reaction nmechanism, in which again iminyl radical was a key intermediate.

### 6.4 C−H Functionalization

**Scheme 172:**
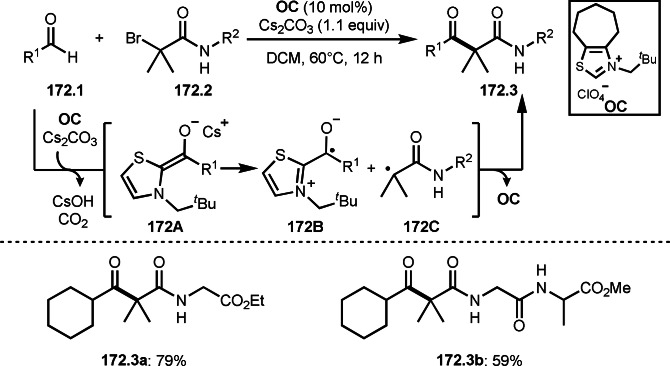
Aldehyde C−H *tert*‐alkylations by NHC catalyst.

While C−H bond transformation is a very powerful tool for aromatics, there are other C−H bonds to be replaced. Ohmiya's group discovered aldehyde (**172.1**) C−H bond transformations with α‐bromocarbonyl compounds (**172.2**) in the presence of NHC catalyst (**OC**) (Scheme 172).[^333^, ^334^] A SET from the Breslow enolate **172A** to **172.2** generated **172B** and **172C**. The subsequent radical‐radical coupling produced compound **172.3** with OC recovery. The NHC radical coupling method is effective for introducing α‐*tert*‐alkyls and is an attractive method for smoothly converting aldehyde C−H bonds.

## 7 Asymmetric Reaction

Asymmetric reactions with α‐bromocarbonyl compounds are also possible, and two reaction formats are known. One is when a tertiary alkyl radical reacts with a nucleophile and the product has a chiral tetrasubstituted carbon, and the other is when the product has a chiral carbon other than the tertiary alkyl moiety. The former is more difficult. There are two ways to control chirality: using metal catalysts with chiral ligands and using the chiral space of enzymes. Overall, this is an immature field, and further development is expected.

### 7.1 Three‐Component Reaction

**Scheme 173:**
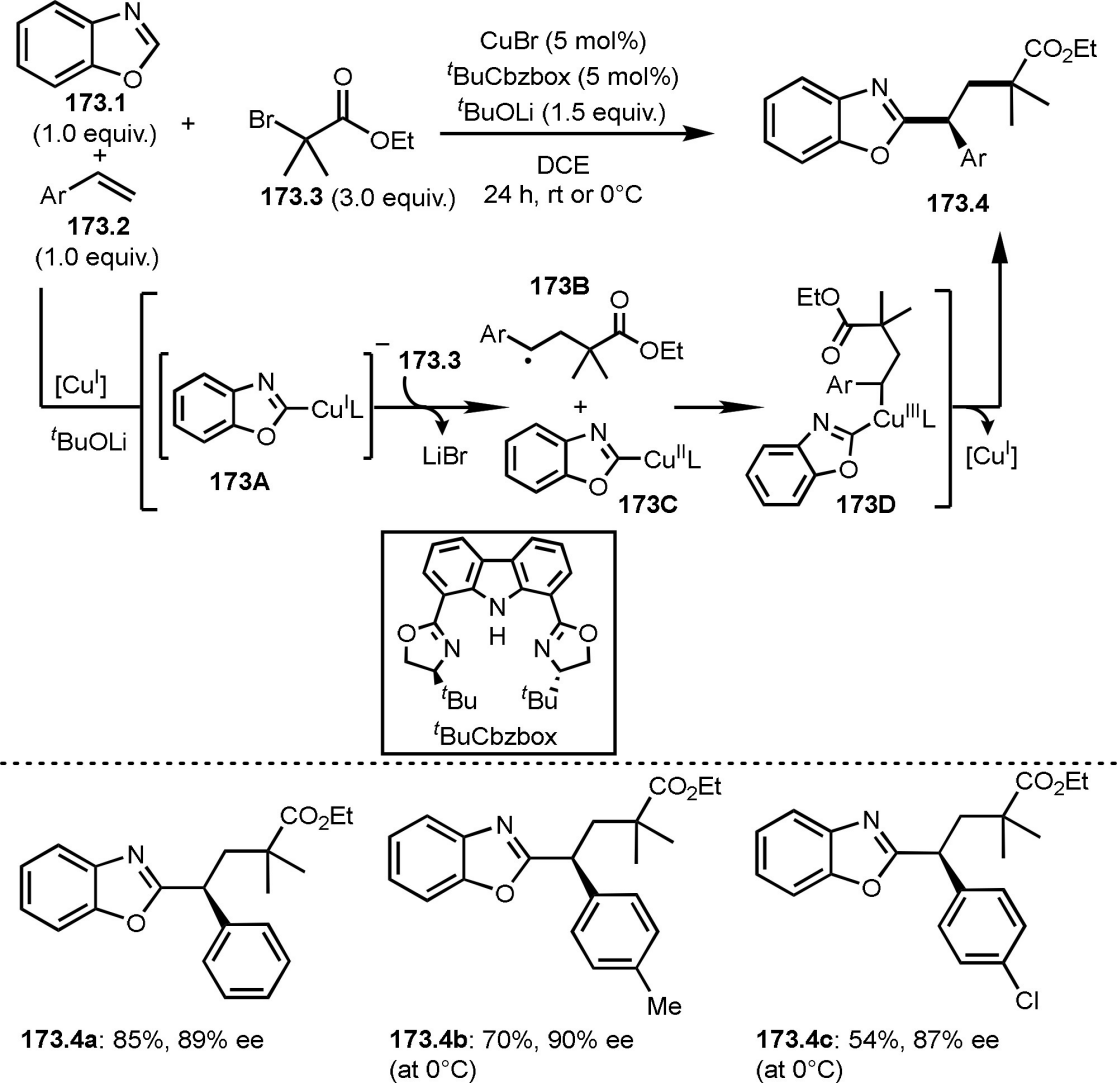
Cu/chiral Cbzbox‐catalyzed asymmetric 1,2‐difunctionalization with benzoxazole.

Zhang's group reported highly enantioselective three‐component coupling of alkenes, α‐bromocarbonyl ester, and (hetero)arenes in the presence of a Cu salt and chiral ^*t*^BuCbzbox ligand (Scheme 173).^[335]^ The reaction of benzoxazole (**173.1**), styrenes (**173.2**) and α‐bromocarbonyl ester (**173.3**) under a chiral Cu catalyst provided *tert*‐alkylated and heteroarylated styrenes (**173.4 a**–**c**) with high enantioselectivities. The reactions proceeded at room temperature or 0 °C. The reaction started with the formation of **173A**, and α‐*tert*‐alkyl radicals were generated from the reaction of **173A** with **173.3**. The α‐*tert*‐alkyl radical reacted with **173.2** to produce **173B**. This **173B** reacted with **173C** containing divalent copper to give **173D**. Enantioselectivity was determined at this stage, resulting in chiral **173.4** after the reductive elimination of **173D**.

**Scheme 174:**
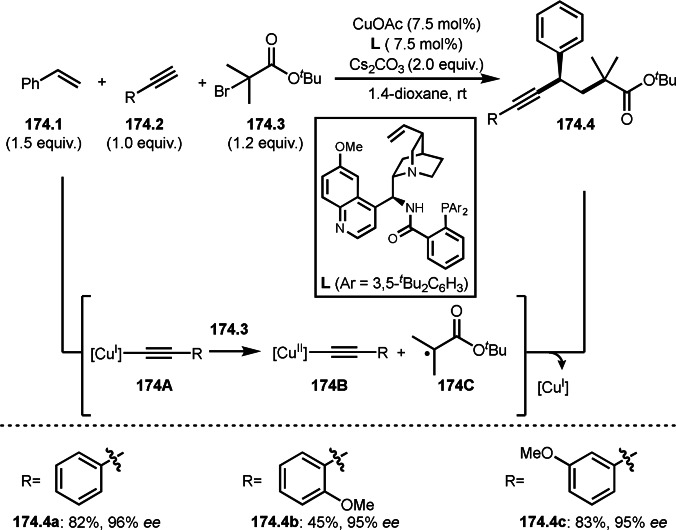
Cu/chiral modified cinchona alkaloid ‐catalyzed asymmetric 1,2‐difunctionalization with alkyne.

Liu's group reported a cinchona alkaloid‐derived multidentate ligand L was effective for asymmetric 1,2‐difunctionalization of styrene (**174.1**) with alkynes (**174.2**) and α‐bromocarbonyl esters (**174.3**) (Scheme 174).^[336]^

In this reaction, two types of unsaturated carbon‐carbon bonds, alkynes and alkenes, are used as reaction substrates, but the target of the addition reaction is the alkene. The alkyne was converted to Cu acetylide (**174A**), which reacted with **174.3** to give an alkyl radical and **174B** involving a divalent Cu. Finally, **174B** and **174C** reacted with alkenes to produce alkynylated and α‐*tert*‐alkylated **174.4**. **174.4** has an optically active carbon at the α‐position of the alkynyl group, which is a secondary carbon.

**Scheme 175:**
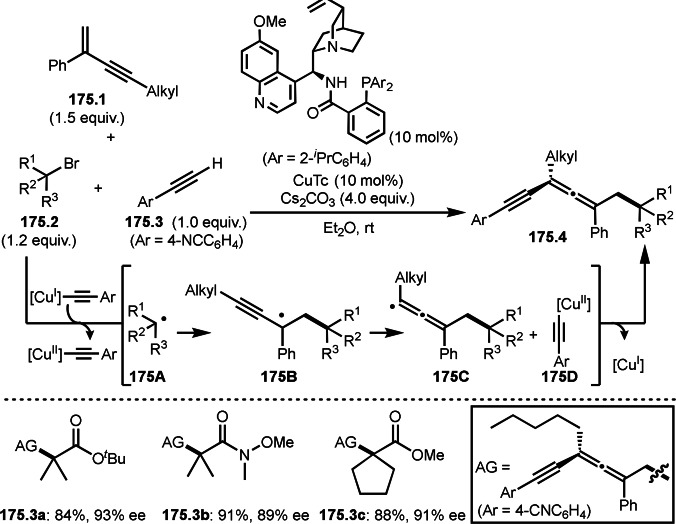
Cu/chiral modified cinchona alkaloid‐catalyzed asymmetric 1,2‐difunctionalization of enyne.

Liu's group also reported the synthesis of chiral allenes (**175.4 a**–**c**) from the reaction of enynes (**175.1**) and α‐bromocarbonyl compounds (**175.3**) under similar catalyst conditions shown in Scheme 174 (Scheme 175).^[337]^ The reaction contained one alkene and two alkynes, but the reaction selectively proceeded only with the alkene moiety. In this reaction, the key intermediate in determining enantioselectivity is **175C**. The reaction of **175C** with **175D**, which has a chiral ligand, gave an optically active product (**175.4**).

**Scheme 176:**
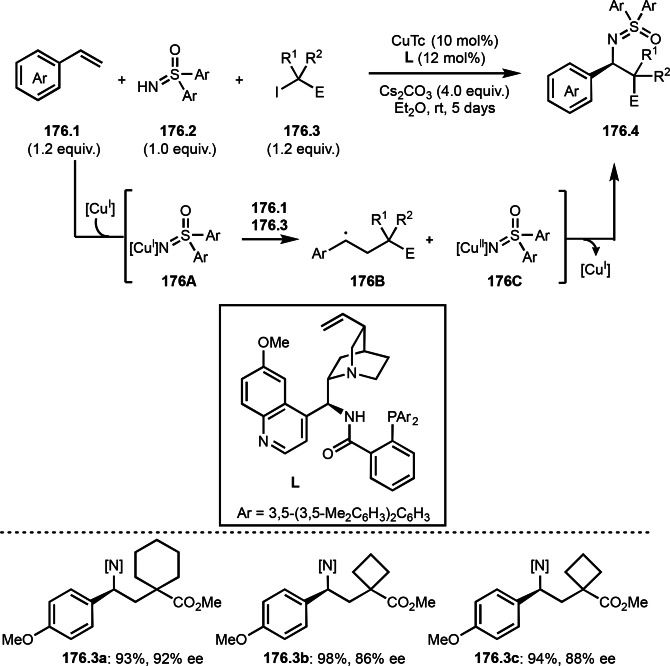
Cu/chiral modified cinchona alkaloid‐catalyzed asymmetric 1,2‐difunctionalization with sulfoximine.

Liu′s group successfully synthesized chiral amines by extending the copper‐catalyzed asymmetric addition reaction between enynes and alkynes (**176.4 a**–**c**) shown in Scheme 174 and 175 (Scheme 176).^[338]^ Enantioselective copper‐catalyzed intermolecular radical 1,2‐carboamination of alkenes (**176.1**), sulfoximines (**176.2**) and α‐iodocarbonyl compounds (**176.3**) gave high ee of **176.4** in good yields. The reaction time was very long, 5 days. The key intermediate in determining the enantioselectivity of this reaction is a chiral copper(I)‐sulfoximinato complex (**176C**). The coupling of **176B** with **176C** produced chiral **176.4**.

**Scheme 177:**
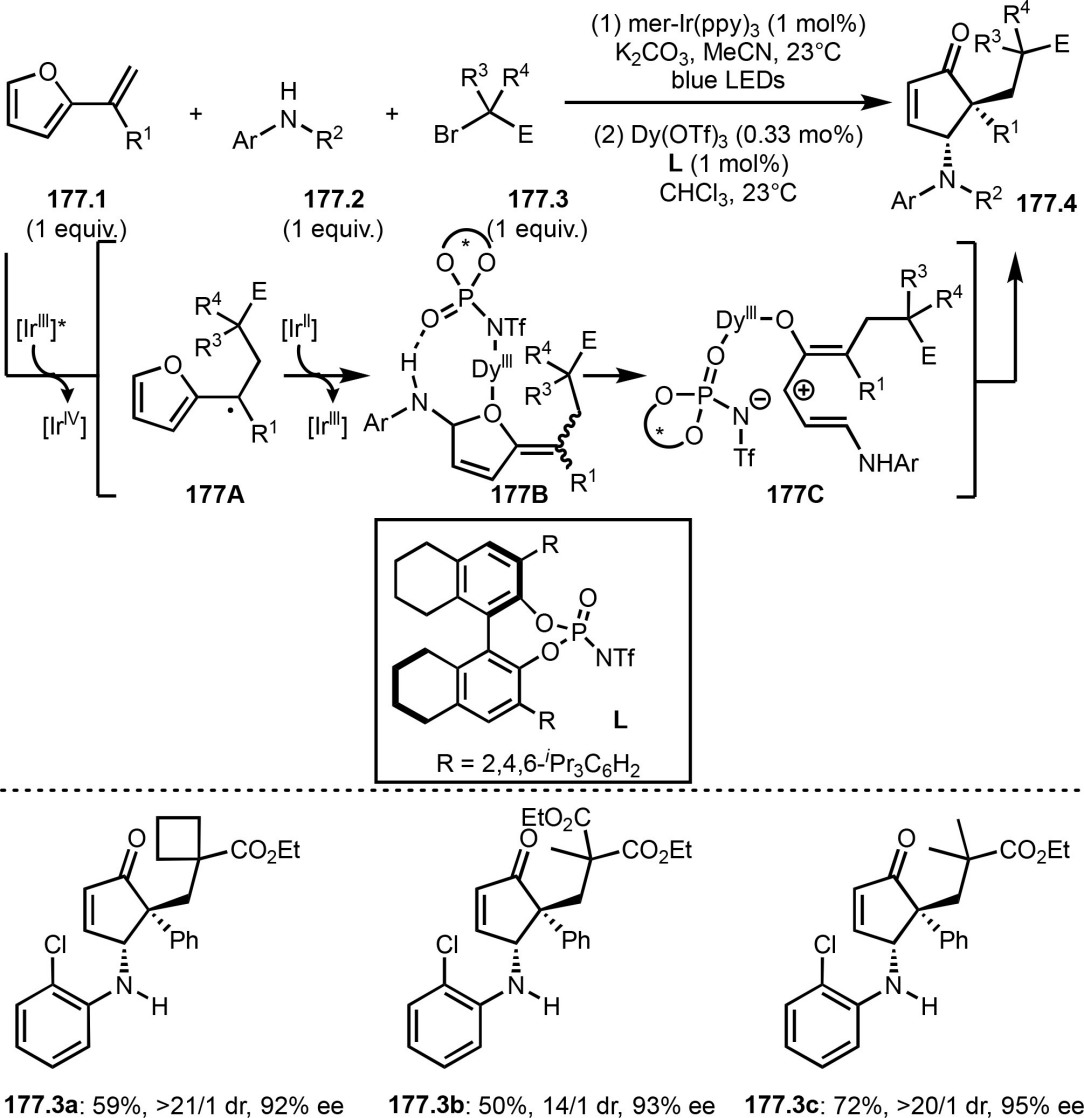
Asymmetric three‐component reaction involving rearrangement and photocatalyst reaction.

Cai's group developed an asymmetric three‐component reaction involving rearrangement and photocatalyst reactions (Scheme 177).^[339]^ The radical reaction was very complex, involving both rearrangement and photoreaction, but proceeded at a high ee. The reaction consisted of two processes. The first step was Ir‐photocatalyzed reaction to produce **177B** via **177A**. In the presence of chiral N‐triflylphosphoramides (**L**) and Dy salt, Subsequently, **177B** produced the cationic intermediate **177C**, which underwent a Dy‐catalyzed rearrangement reaction. Finally, using the asymmetric space around Dy, an asymmetric cyclization reaction proceeded, giving chiral **177.4**.

### 7.2 Cyclization

**Scheme 178:**
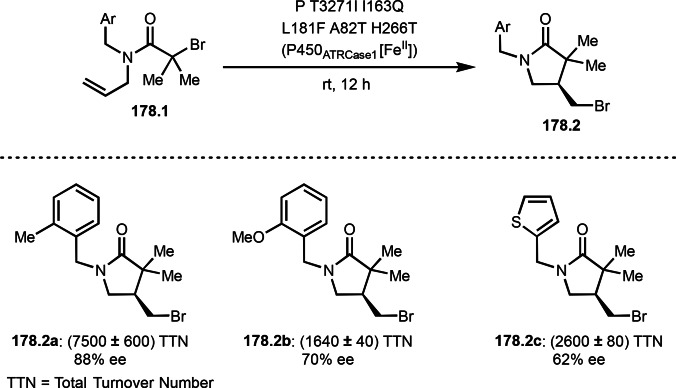
Asymmetric ATRC of α‐bromocarbonyls with metalloenzyme.

ATRC reaction is one of the most important reaction in α‐bromocarbonyl chemistry. But asymmetric ATRC is rare.^[340]^ Yang's group accomplished enzyme‐controlled asymmetric radical cyclization reaction (Scheme 178).[^341^, ^342^] They used Fe enzymes, metalloprotein, as a catalyst. In this case, engineered cytochromes P450 was the best enzyme and was suitable for asymmetric ATRC. In this asymmetric ATRC reaction, the chirality of the secondary carbons in the ring can be controlled rather than the construction of an optically active quaternary carbon. α‐*tert*‐Alkyl radical species were generated from the reaction of **178.1** and Fe^II^ in P450. The resulting radical was cyclized in asymmetric space of the enzyme. As the results, ATRC product (**178.2**) was obtained in high ee.

**Scheme 179:**
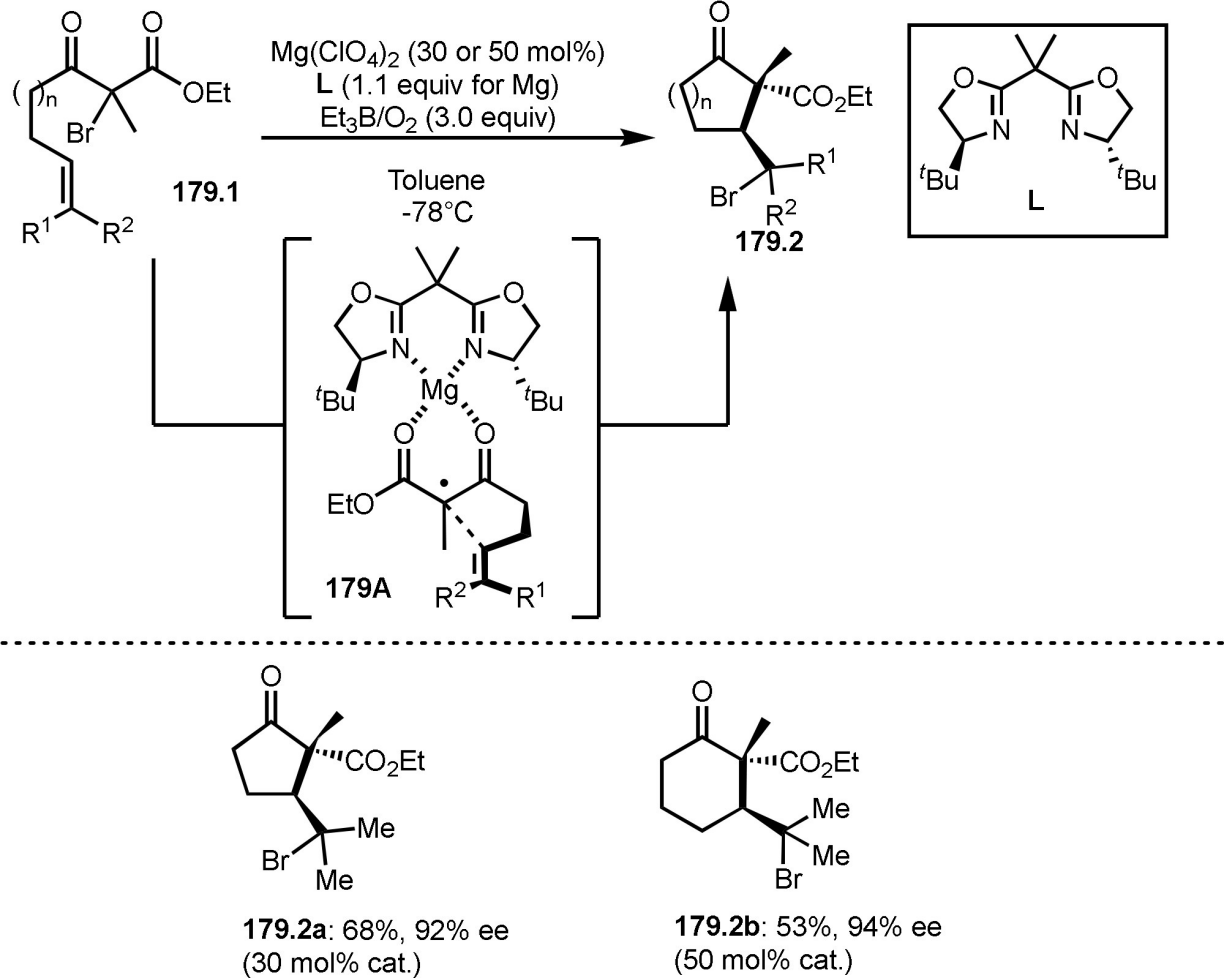
Asymmetric ATRC of α‐bromocarbonyls by a Cu/box ligand catalyst.

Yang's group reported Cu/box ligand (L)‐catalyzed asymmetric ATRC reactions of α‐bromocarbonyl compounds possessing a homoallyl group (**179.1**) (Scheme 179).[^343^, ^344^] The combination of Lewis acidic Mg salt and asymmetric box ligand (**L**) efficiently promoted the asymmetric cyclization reaction to yield the corresponding ATRC products (**179.2 a**, and **b**). In this case, α‐*tert*‐alkyl radical species were generated by the reaction of **179.1** with Et_3_B/O_2_. This radical formation was very mild and proceeded after the Mg salt activated the substrate. This allows the reaction to proceed in a specific direction during cyclization to obtain chiral ATRC products. However, the very low reaction temperatures require more catalysts (30 to 50 mol%) for efficient reactions.

**Scheme 180:**
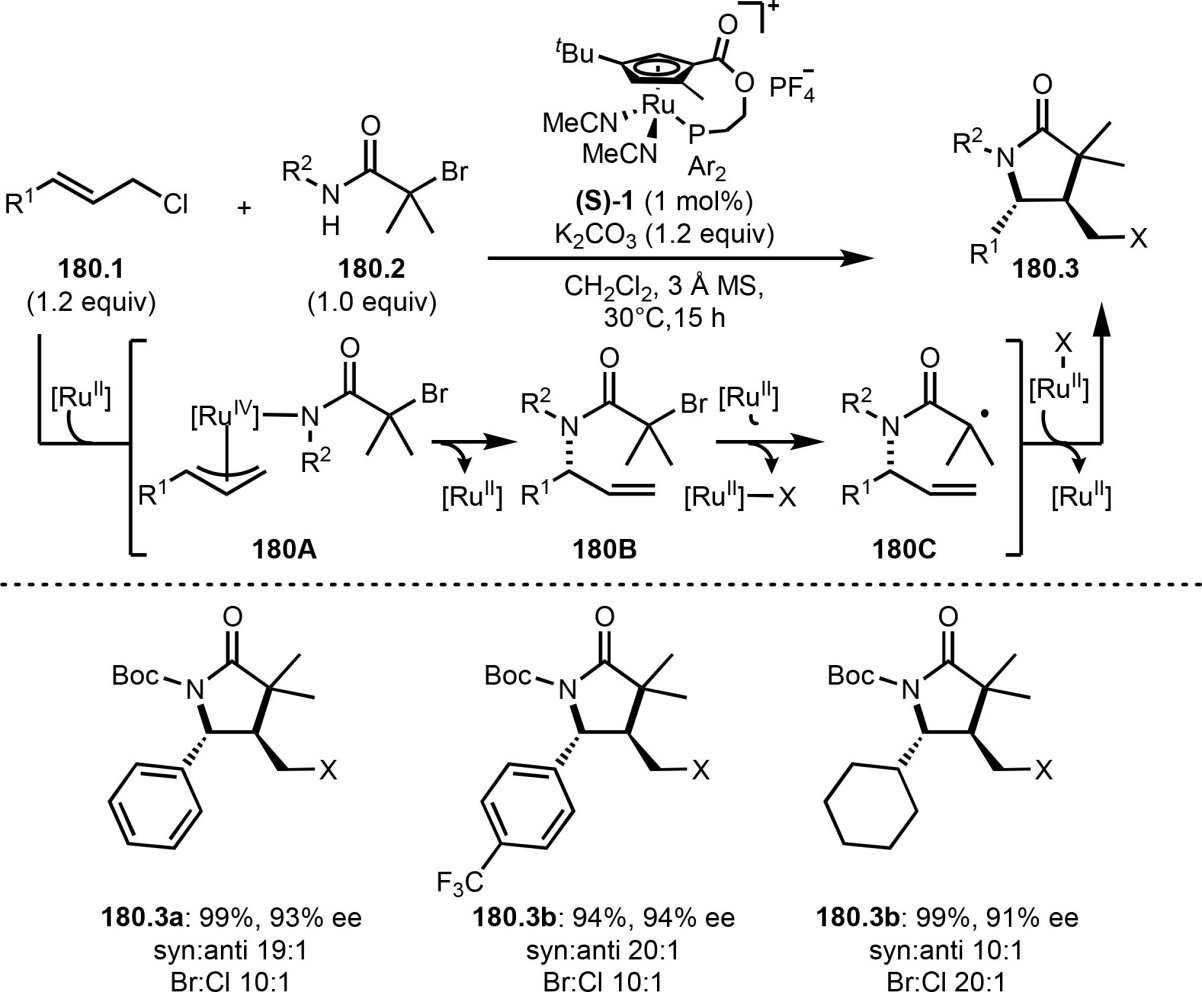
Intermolecular asymmetric ATRC reaction by chiral Ru complex.

Okamura and Onitsuka's group developed intermolecular asymmetric ATRC reaction (Scheme 180).^[345]^ Compared to intramolecular asymmetric ATRC, intermolecular asymmetric ATRC is more difficult because both substrates must be precisely controlled. They dissolved this problem by using a Ru catalyst shown in Scheme 180. Under their conditions, ATRC products possessing two chiral carbons were obtained in high yields with high ees. Initially, an allylic Ru complex was generated from the reaction of Ru, **180.1** and **180.2**. Next, **180B** was produced via allylic amination process. This step determines the chirality of the α‐position of N. The resulting Ru^I^ species reacted with α‐bromocarbonyl moiety to generate **180C**. Finally, asymmetric radical cyclization of **180C** occurred to give chiral ATRC product (**180.3**). This final step also included the generation of the chiral center.

**Scheme 181:**
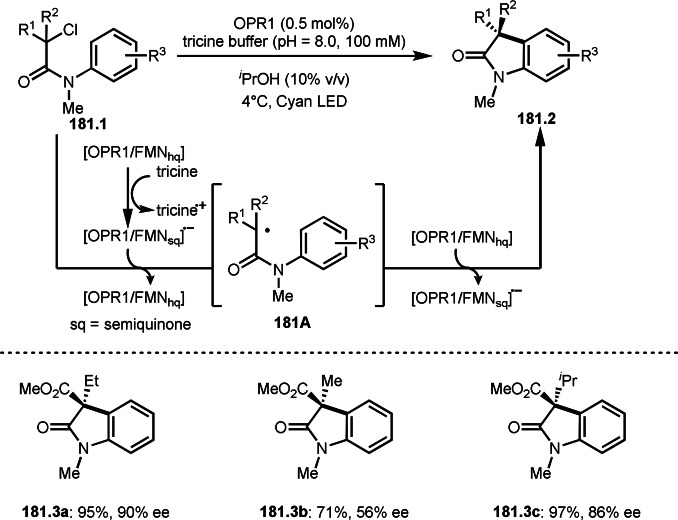
Asymmetric C−H cyclization by enzyme.

Hyster's group reported asymmetric aromatic C−H cyclization to construct chiral quaternary centers under enzyme (OPR1) conditions (Scheme 181).^[346]^ Unlike other asymmetric reactions using α‐bromocarbonyl, this asymmetric reaction is extremely difficult because the asymmetry is controlled at the α‐position of the substrate. This is because when α‐bromocarbonyl is activated by a catalyst or radical initiator, its α‐position becomes a highly active, short‐lived radical species. Enzymes achieve this asymmetric reaction. Since the enzyme has a three‐dimensional chiral space, it is well suited for the formation of quaternary carbons, which also have a three‐dimensional extent. The reaction of **181.1** and flavin hydroquinone (FMNhq) of OPR1 gave **181A** via SET. **181A** underwent asymmetric cyclization within the enzyme. Flavin hydroquinone/semiquinone (FMNhq/FMNsq) redox system is convenient for the generation of α‐*tert*‐alkyl radical species.

**Scheme 182:**
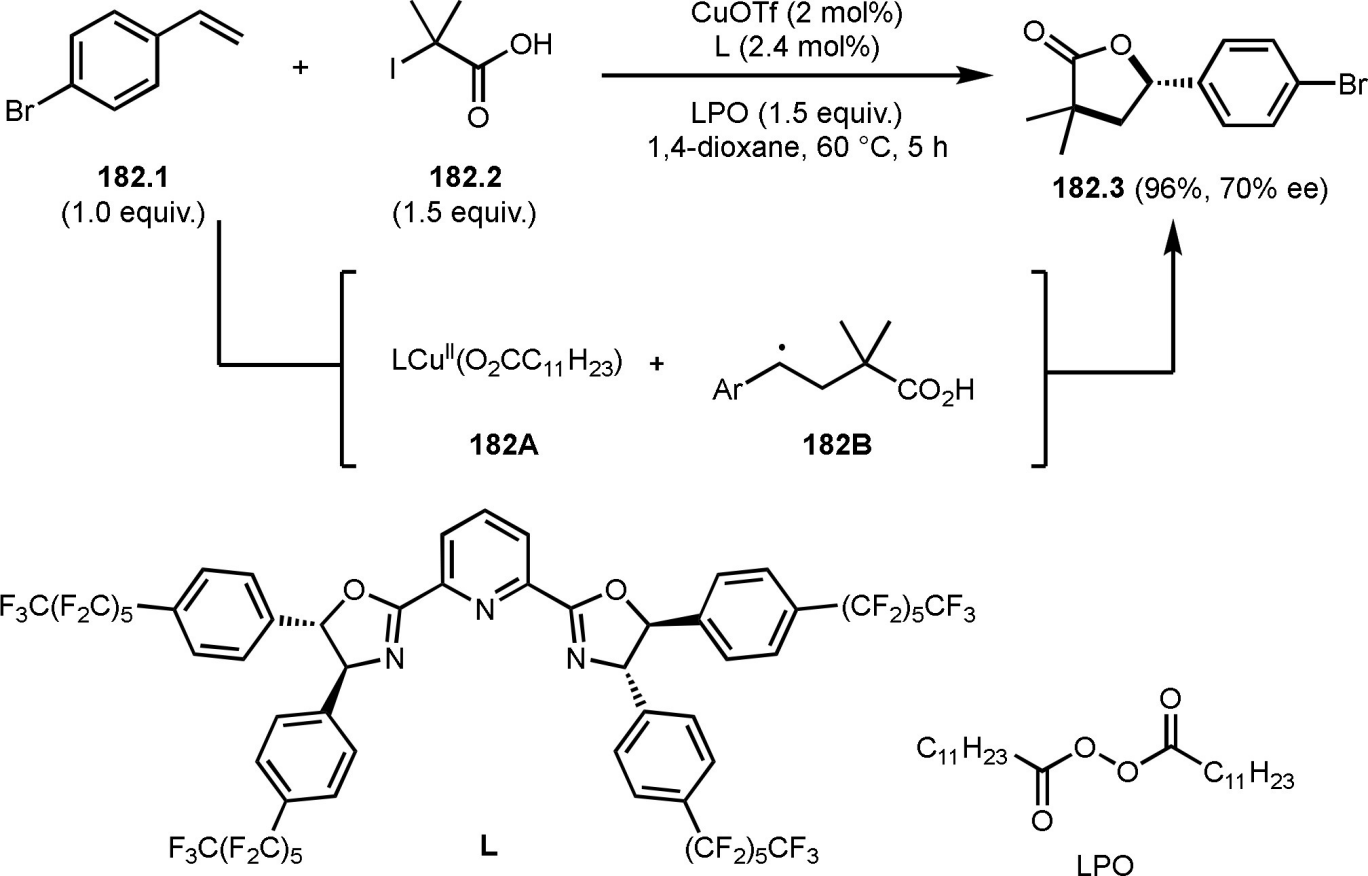
Asymmetric lactonization reaction by Cu/asymmetric pybox ligand system

Bao's group reported chiral lactone synthesis under Cu/chiral perfluoroalkylated pybox system (Scheme 182).^[347]^ The asymmetric lactonization reaction of Br‐styrene (**182.1**) with α‐iodocarboxylic acid (**182.2**) proceeded using an asymmetric pybox ligand with a very large substituent. α‐Iododocarboxylic acid possessing a *tert*‐alkyl moiety (**182.2**) was used in only one case, but the corresponding lactonization reaction proceeded at 70 % ee. Because their main focus in this paper was to use α‐iodocarboxylic acid possessing a primary‐ or secondary alkyl moiety. Although very simple reaction, the asymmetric cyclization reaction proceeds with **182B** coordinated with a chiral copper catalyst (**182A**). The low ee is probably due to the high reaction temperature. If the reaction could be performed at lower reaction temperatures, the selectivity would be much higher.

### 7.3 Cross‐Coupling Reaction

The papers commented in Section 7.1 and Section 7.2 do not report asymmetric reaction proceeding on the α‐carbon of α‐bromocarbonyl compounds. Therefore, they are slightly different from the reactions that truly utilize the α‐bromocarbonyl compounds, which is the purpose of this review. Here, we introduce a cross‐coupling that controls the chiral quaternary centers of the α‐bromocarbonyl compounds, which is the original purpose of this review.

**Scheme 183:**
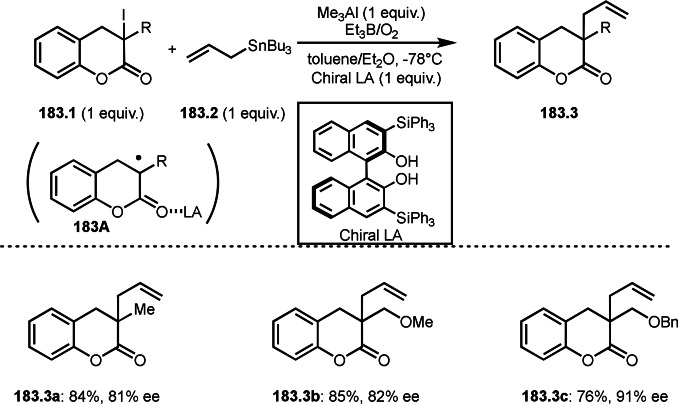
Asymmetric cross‐coupling with allyl stannane.

Hoshino's group reported asymmetric allyl couplings of cyclic α‐iodocarbonyl compounds (**183.1**) with allyltributylstannane (**183.2**) in the presence of Me_3_Al/silylated binol as a chiral LA (Scheme 183).[^348^, ^349^] Under the conditions, they successfully created chiral all carbon quaternary centers (**183.3 a**–**c**) with high ees. Cyclic α‐*tert*‐alkyl radical species were generated from the reaction of **183.1** and Et_3_B/O_2_. Linear α‐iodocarbonyl compounds are difficult to asymmetric reaction, while cyclic α‐bromocarbonyl compounds are relatively easy to asymmetric reaction because the intermediates are rigid. This is because the radical structure of the intermediate is suitable for selective reactions. In fact, the radical intermediate (**183A**) of this reaction could underwent asymmetric coupling with **183.2** by the coordination of chiral Lewis acids. To explain the selectivity, a five‐coordinated aluminum complex has been proposed.

**Scheme 184:**
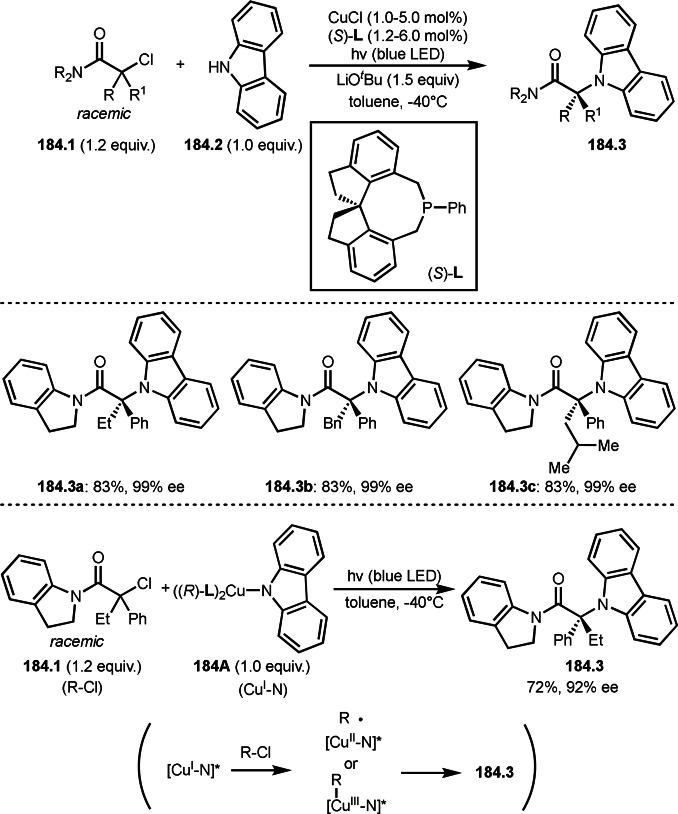
Photoinduced copper‐catalyzed asymmetric N‐tert‐alkylation.

Cyclic α‐halocarbonyl compounds have a planar structure after radical formation, making asymmetric reactions relatively easy to control. On the other hand, linear α‐halocarbonyl compounds are extremely difficult to carry out asymmetric reactions because their structures cannot be fixed after radical formation. Peters and Fu's group dissolved this difficult problem by using monodentate phosphine ligand (**L**) in amination reactions of α‐chlorocarboxamides (**184.1**) with planar carbazoles (**184.2**) (Scheme 184).[^350^, ^351^] Under their photoinduced copper‐catalyzed conditions, various N‐tert‐alkylated substituted carbazoles (**184.3 a**–**c**) can be synthesized in high ees. The reaction was initiated by the formation of Cu‐amide (**184A**). **184A** was then excited by visible light, and photo‐excited **184A** reacted with **184.1** to form α‐*tert*‐alkyl radicals, or Cu^III^ complexes, which were formed via oxidative addition. Asymmetric recognition took place at this stage, giving the final chiral product (**184.3**).

**Scheme 185:**
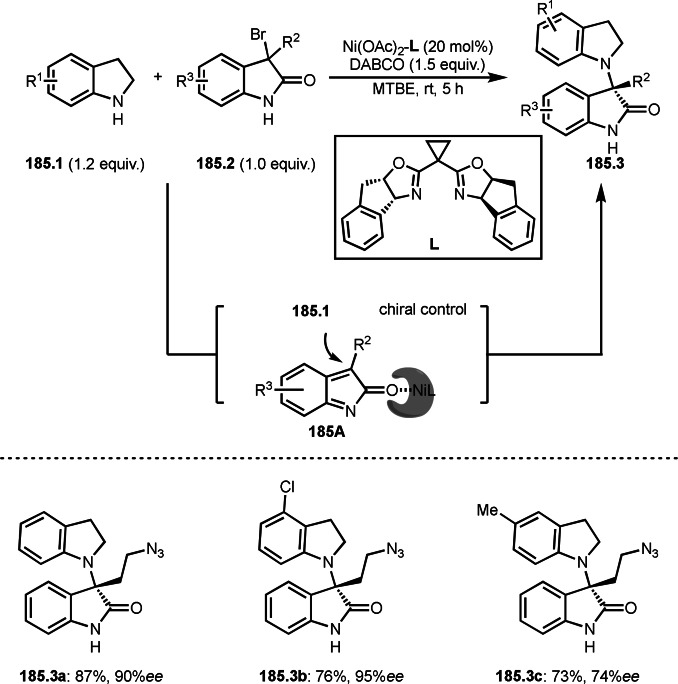
Asymmetric amination reaction by Ni/chiral box ligand catalyst.

Ni/chiral ligand catalysts are effective in the construction of chiral tetrasubstituted carbon centers. Wang's group reported Ni‐catalyzed asymmetric aminations of indolines (**185.1**) with 3‐bromooxindoles (**185.2**) to produce chiral 3‐aminooxindoles (**185.3 a**–**c**) (Scheme 185).[^352^, ^353^] Chiral box ligand (L) was found to be the best ligand for a Ni salt. In this reaction, no α‐tert‐alkyl radical species were formed; instead, **185A** was formed. This is interesting point of this reaction. A radical is formed from the α‐bromocarbonyl compound, and the S_RN_1 reaction of an amine with it is also possible. However, in this reaction, the addition of the amine to **185A** proceeded. Chiral control was achieved by the reaction of **185A** with **185.1**.

**Scheme 186:**
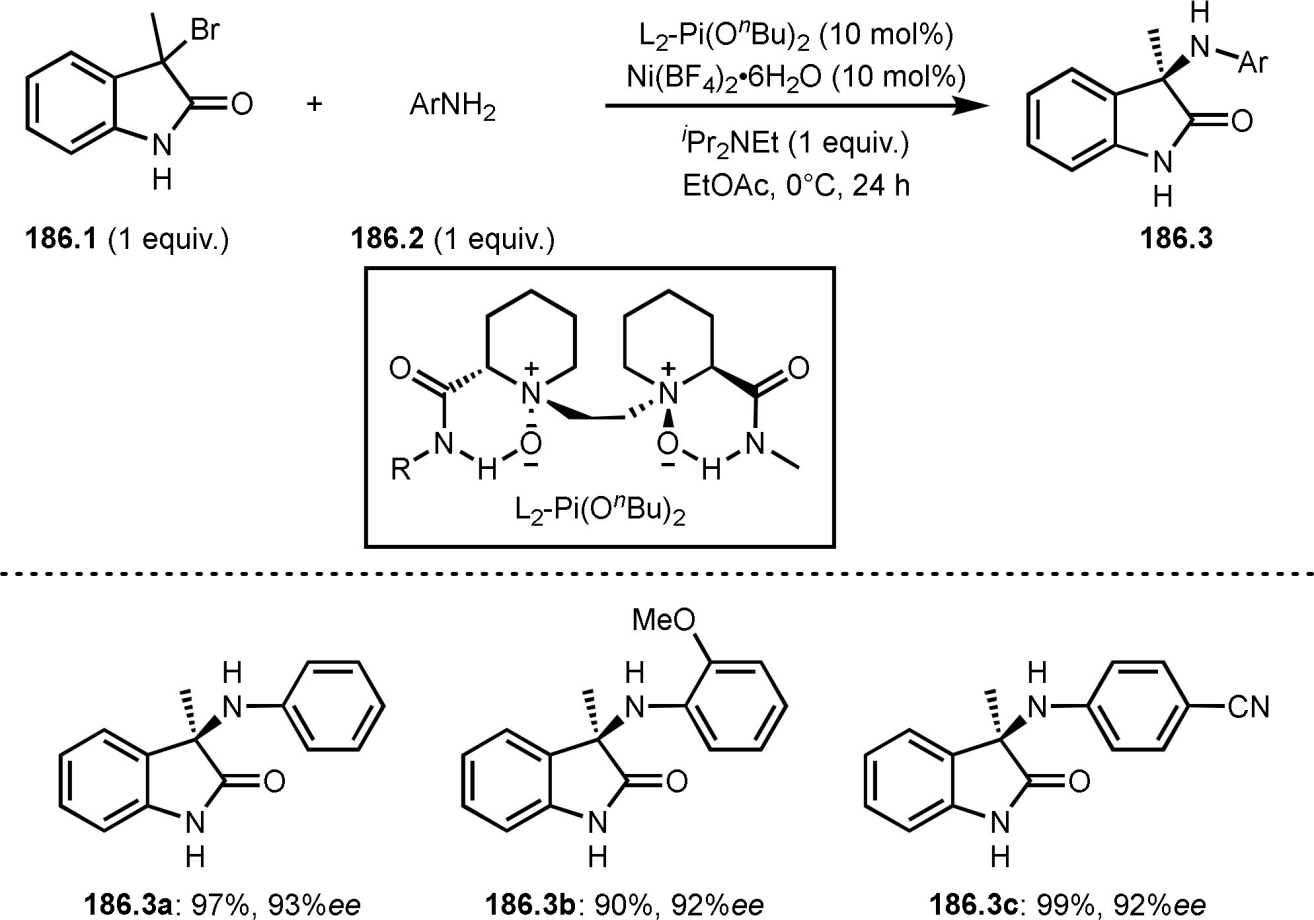
Ni/dioxide catalyzed asymmetric amination.

**Scheme 187:**
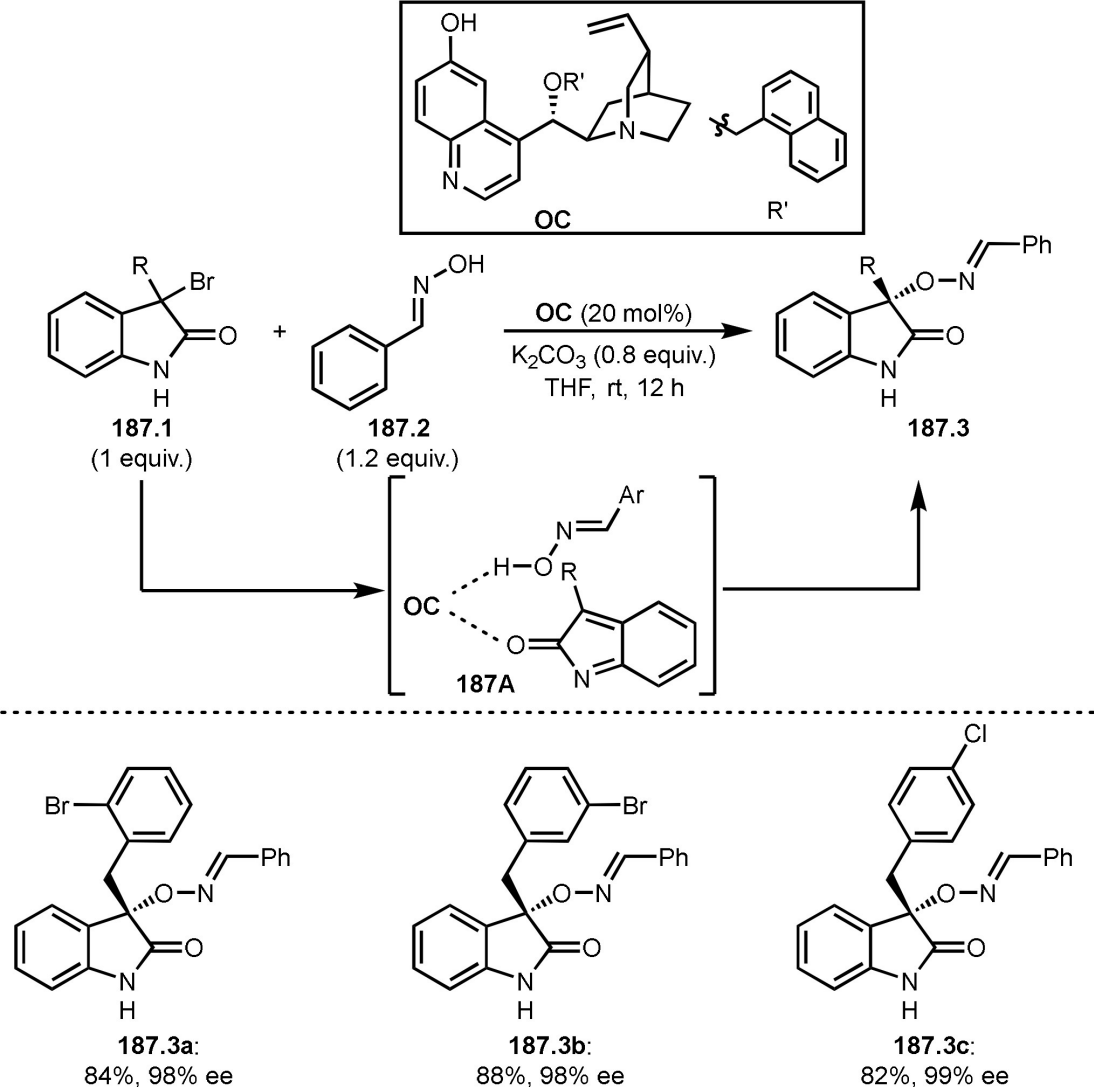
Cinchona alkaloid derivatives catalyzed asymmetric cross‐coupling with oximes.

**Scheme 188:**
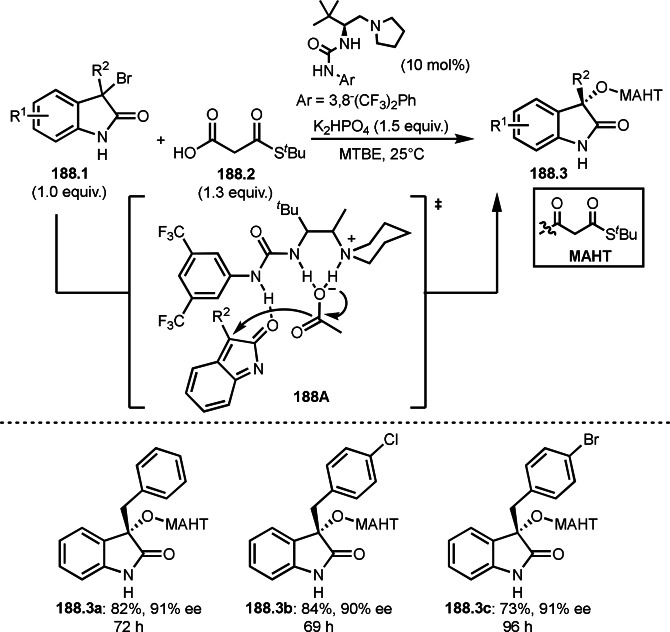
L‐amino acid‐based urea catalyzed asymmetric cross‐coupling with carboxylic acids.

**Scheme 189:**
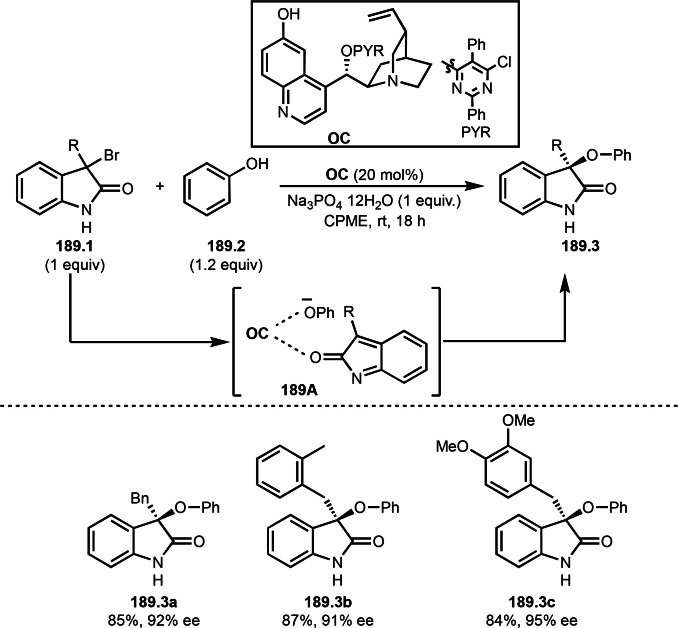
Cinchona alkaloid derivatives catalyzed asymmetric cross‐coupling with phenols.

Liu and Feng's group reported Ni‐catalyzed asymmetric amination of cyclic α‐bromocarboxamide (**186.1**) to synthesize 3‐amino‐3‐substituted oxindoles (**186.3 a**–**c**) (Scheme 186).[^354^, ^355^, ^356^] With the ligand (N,N’‐dioxide (L_2_−Pi(O^*n*^Bu)_2_)) they developed, the reaction proceeded in high yields with high ees. As in Scheme 185, it proceeds via 2H‐indol‐2‐one intermediate. 2H‐indol‐2‐one intermediate has a planar structure, making it easy to control selectivity in chiral *tert*‐alkylations. For example, asymmetric organocatalyzed system is very effective to carry out C−O bond formations (Scheme 187,^[357a]^ Scheme 188,^[358]^ Scheme 189, ^[359]^). Oxime, malonic half‐thioester and phenol were used as nucleophile in those reactions. Cinchona alkaloid and L‐amino acid‐based urea, chiral organocatalysts, were effective in obtaining high ees of substituted oxindoles (**187.3**, **188.3**, and **189.3**). Those ligands were coordinated to 2H‐indole‐2‐one intermediates in the reaction system to achieve selective nucleophilic reactions.

**Scheme 190:**
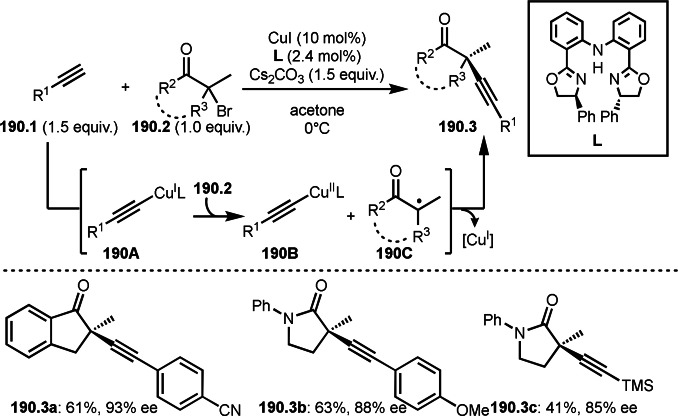
Asymmetric alkynylation with Cu/chiral bisoxazoline phenyl amine catalyst system.

Zhang's group reported Cu‐catalyzed asymmetric cross‐couplings with terminal alkyne (**190.1**) and cyclic α‐bromocarbonyl compound (**190.2**) (Scheme 190).^[360]^ They used chiral bisoxazoline phenyl amine as a ligand and successfully obtained chiral *tert*‐alkylated alkynes (**190.3**) possessing an all‐carbon quaternary center in moderate yields with high ees. The reaction first provided Cu^I^ acetylide (**190A**), which reacted with **190.2** to form **190B** and α‐*tert*‐alkyl radical species (**190C**) via SET. When the resulting radical **190C**, which has a planar structure, reacts with chiral **190B**, enantioselectivity is determined. Liu's group also reported similar asymmetric coupling reaction under Cu/cinchona alkaloid derivatives catalyst system.^[361]^

**Scheme 191:**
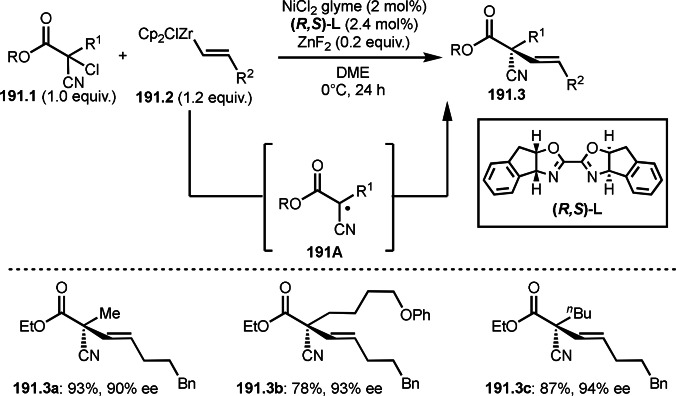
Asymmetric coupling with 1‐alkenylZr catalyzed by a chiral Ni catalyst.

1‐Alkenylation can also be applied to asymmetric reactions. Fu's group reported the reaction of α‐chlorocarbonyl compounds (**191.1**) with 1‐alkenyl‐Zr (**191.2**) under Ni/chiral bisoxazole ligand system to produce all‐carbon quaternary centers (**191.3 a**–**c**) (Scheme 191).^[362]^ In this reaction, both cyclic and acyclic **191.1** can be used. A pyridine substituted oxazole chiral ligand was effective for cyclic **191.1**, in which a key intermediate was a rigid radical species. On the other hand, the reaction with acyclic **191.1** employed bisoxazole chiral ligand ((*R*,*S)−*L). ZnF_2_ is important to promote the reaction. Without the salt, the yield was decreased. This salt coordinates to Cl at **191.1** and may activate the C−Cl bond so that the reaction proceeds at low temperatures. In this case, a radical intermediate (**191A**) is not rigid structure, which is very difficult to control the enantioselectivity. Enantiorecognition step may be the reaction of **191A** with 1‐alkenyl‐Ni/chiral ligand, which gives an alkyl‐Ni^III^L‐1‐alkenyl species. Finally, chiral product (**191.3**) was generated via reductive elimination.

**Scheme 192:**
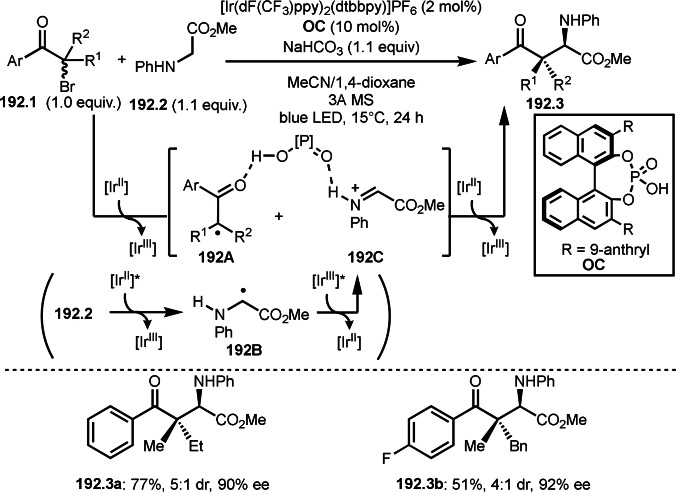
Asymmetric coupling of amino acid derivatives catalyzed by Ir‐photocatalyst/chiral phosphoric acid.

Wang's group discovered Ir‐photocatalyst/chiral phosphoric acid dual catalyst system for asymmetric alkyl coupling reaction (Scheme 192).^[363]^ The reaction of α‐bromocarbonyl compounds (**192.1**) with α‐amino acid derivative (**192.2**) gave chiral alkyl coupling products (**192.3 a**, **b**) in moderate yields with high ees. Ir‐photocatalyst is for the generation of α‐*tert*‐alkyl radical (**192A**) from **192.1** and α‐carbon radical (**192B**) from **192.2**, and chiral phosphoric acid promotes the asymmetric coupling process between **192A** and **192C**. They observed Ir‐photocatalyst (E_1/2_*^III/II^=+1.21 V vs. SCE) was quenched by glycine ester (**192.2**) (E_1/2_^ox^=+0.95 V vs. SCE). The resulting **192B** was oxidized with [Ir^III^]* to form **192C**. **192.3** was finally obtained from the coupling of **192A** with **192C**.

**Scheme 193:**
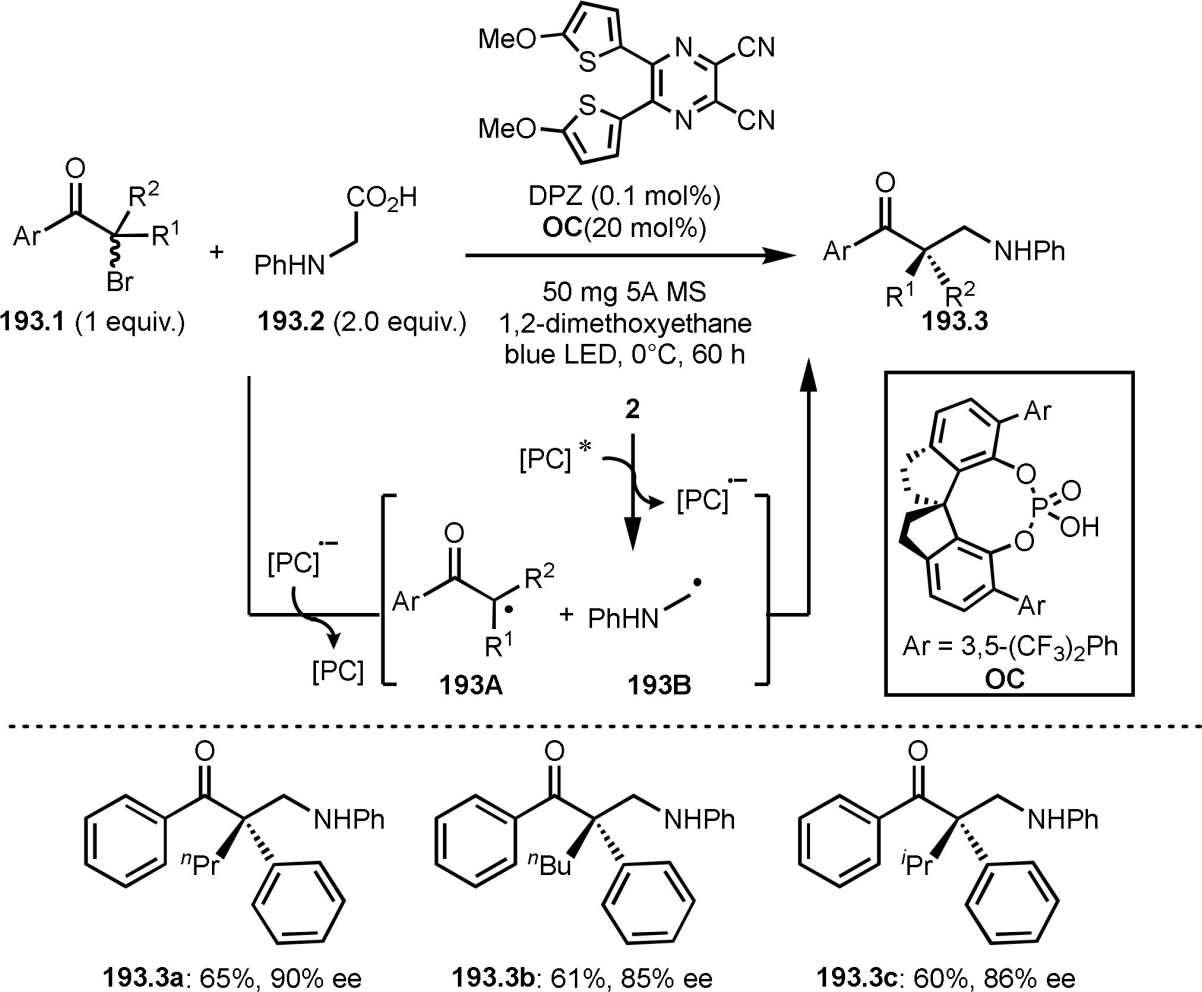
Decarboxylative asymmetric couplings catalyzed by organocatalyst and chiral phosphoric acid.

Wang's group reported the reaction at α‐position of amino acids, whereas Jiang's group reported decarboxylative asymmetric couplings between α‐bromoketones (**193.1**) with α‐amino acid derivative (**193.2**) in the presence of an organophotocatalyst (Scheme 193).[^364^, ^365^] Organocatalyst generated both α‐*tert*‐alkyl radical (**193A**) and aminomethyl radical (**193B**). The enantioselective coupling can be accomplished via phosphoric acid catalyzed process shown in Scheme 192. Under these conditions, both cyclic and acyclic α‐bromocarbonyls can react with amino acids to produce the coupling products possessing chiral quaternary carbon centers.

**Scheme 194:**
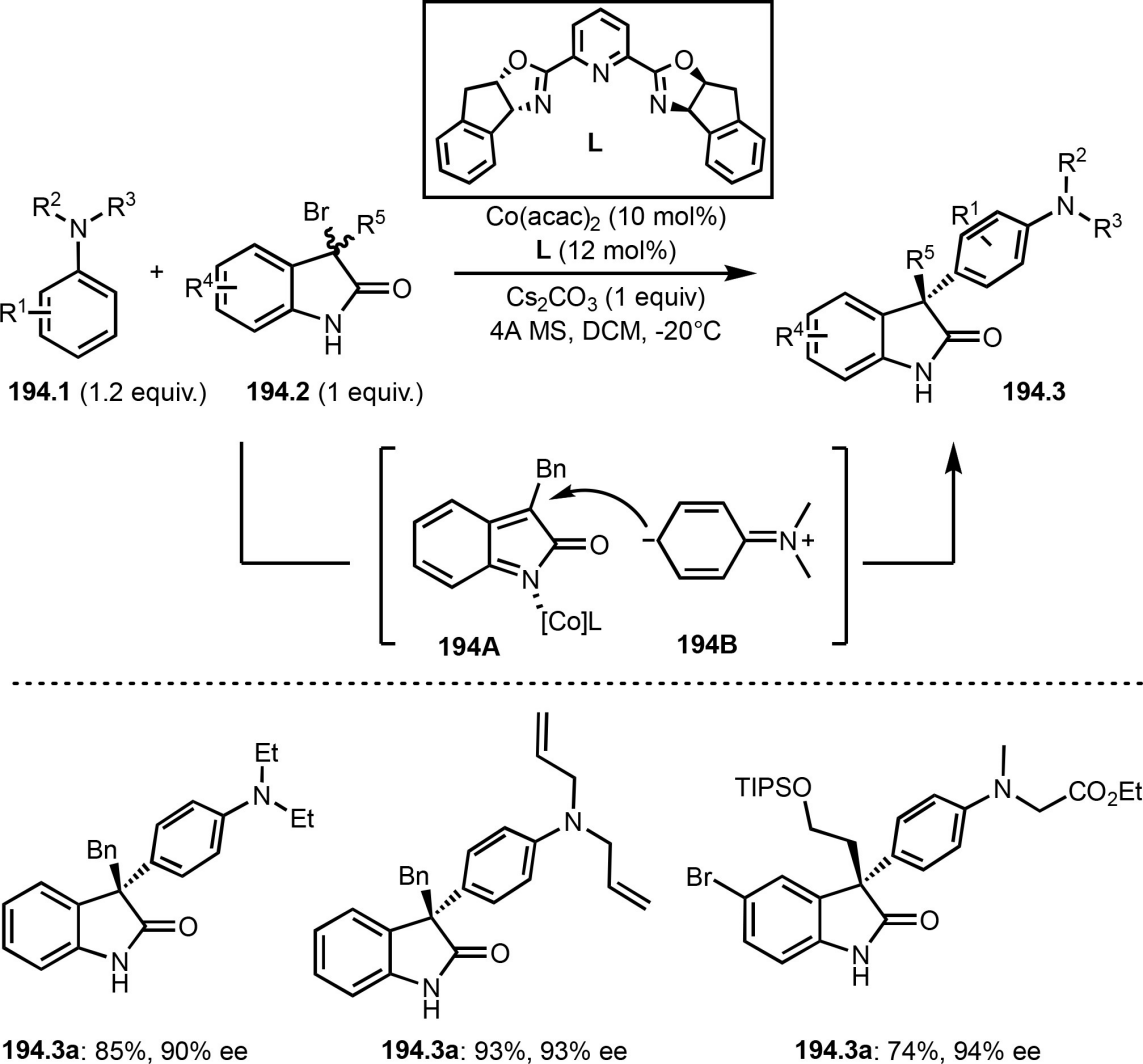
Asymmetric C−H coupling catalyzed by Co/pybox.

Scheme s 186–189 showed the effect of 2H‐indol‐2‐one intermediates on asymmetric coupling with various nucleophiles. Zhang's group reported 2H‐indol‐2‐one intermediates is also effective for asymmetric C−H couplings (Scheme 194).^[366]^ Pybox type chiral ligand enabled Co‐catalyzed asymmetric C−H couplings of aniline derivatives (**194.1**) and cyclic α‐bromocarboxamide (**194.2**) to produce para‐C−H α‐*tert*‐alkyl‐substituted products (**194.3**). In this reaction, the C−H reaction selectivity of **194B** is easily controlled because the chiral ligand/Co‐coordinated 2H‐indol‐2‐one intermediate (**194A**) has a planar structure. With this catalyst system they developed, the reaction provided various C−H coupling produces (**194.3 a**–**c**) in high yields with high ees.

### 7.4 Acidic C−H Functionalization

Enamines and enolates are prepared from the corresponding carbonyl compounds with acidic protons. Their planar structure is suitable for enantioselective reactions. However, the reactions described here do not control the chirality of the α‐carbon of the α‐halocarbonyl compound; the asymmetric reaction proceeds at a different site.

**Scheme 195:**
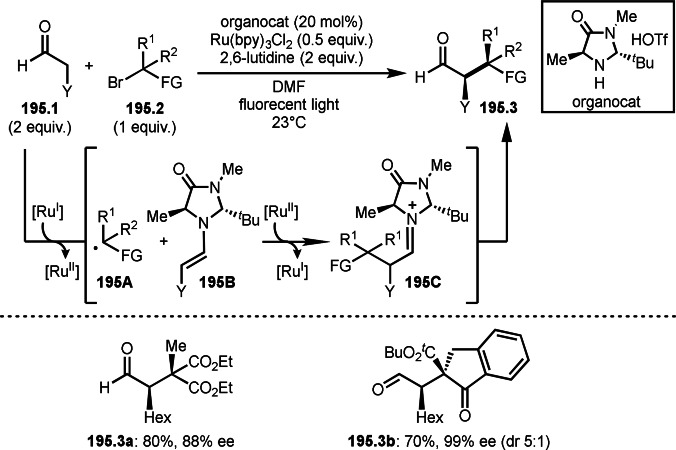
Asymmetric α‐*tert*‐alkylation of aldehydes catalyzed by Ru and organocatalyst.

MacMillan's group reported the reaction of aldehydes (**195.1**) with α‐bromocarbonyl compounds (**195.2**) in the presence of chiral organocatalyst (Scheme 195).[^367^, ^368^] Merging chiral organocatalyst and photoredoxcatalyst has enabled asymmetric *tert*‐alkylation of aldehydes. The imidazolidinone‐based organocatalyst generated chiral enamine intermediate (**195B**), whereas Ru‐photocatalyst generated α‐*tert*‐alkyl radical species (**195A**). Asymmetric radical addition of **195A** to **195B** followed by Ru^II^ oxidation gave **195C**. The reduction potential of the radical species produced in this case is ca. −1 V (−0.92 to −1.12 V versus SCE in CH_3_CN). Hydrolysis then occurred, producing **195.3**. They achieved complex *tert*‐alkylation by combining different reaction formats: photoreaction and asymmetric organocatalysis.

**Scheme 196:**
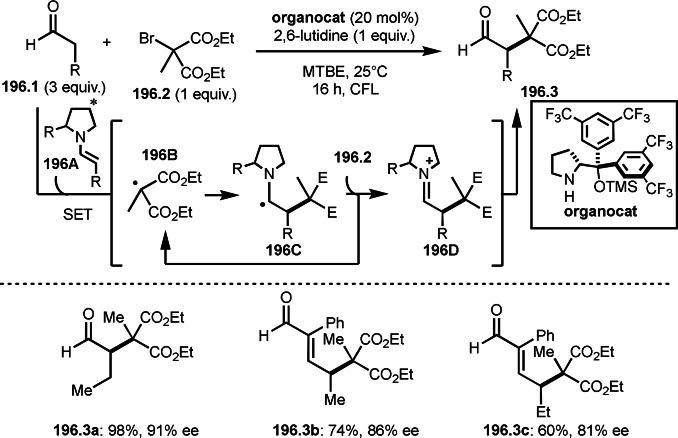
Asymmetric α‐ and γ‐alkylation of aldehydes catalyzed by organocatalyst.

Melchiorre's group reported the reaction of aldehydes (**196.1**) with α‐bromocarbonyl compounds (**196.2**) in the presence of chiral organocatalyst (Scheme 196).^[369]^ Asymmetric α‐ and γ‐alkylation of aldehydes (**196.3 a**–**c**) are easily accomplished via in situ generated chiral enamine intermediates under photo‐irradiation. Photo‐excited enamine (**196A**) reacted with **196.2** to produce an α‐*tert*‐alkyl radical species (**196B**), which reacted with the ground state **196A** to produce **196C**. The enantioselectivity was determined at this step. The reduction potential of **196A** was −2.50 V (vs Ag/AgCl, NaCl sat). This value is enough to reduce **196.2**. Next, **196C** was oxidized with **196.2** to give iminium ion (**196D**) and **196B**. Finally, chiral **196.3** was obtained via hydrolysis of **196D**. They also carried out the reaction under natural solar light, and successfully obtained the product (**196.3 a**) in 98 % yield with 91 % ee.

This is different from the above reaction categories, but the asymmetric auxiliary group allows for selective radical reactions with α‐bromocarbonyl compounds.

**Scheme 197:**
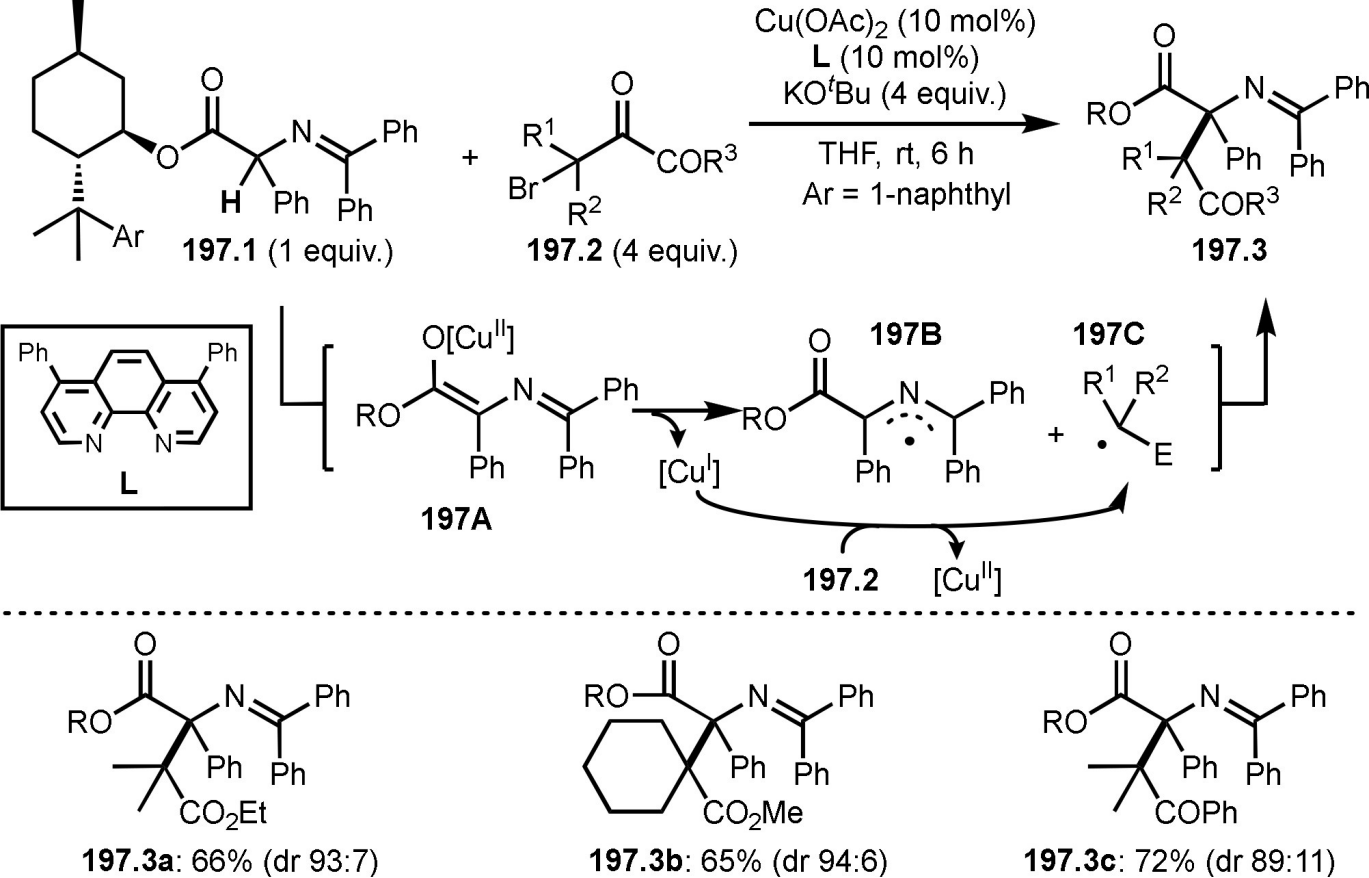
Selective radical addition of amino acid Schiff bases with α‐bromocarbonyl compounds.

Amino acid Schiff bases are one of the most important starting materials for the synthesis of various natural and unnatural α‐amino acids. Yazaki, Nishikata and Ohshima's group reported the reaction of chiral amino acid Schiff bases bearing benzophenone imine (**197.1**) with α‐bromocarbonyl compounds (**197.2**) in the presence of a Cu catalyst (Scheme 197).^[370]^ They used menthol derivatives as a chiral auxiliary in **197.1**. Under their conditions, chiral various highly congested α‐amino acid derivatives (**197.3 a**–**c**) were synthesized. At first, a Cu‐enolate intermediate (**197A**) was generated from the reaction of Cu^II^ and **197.1** in the presence of KO^*t*^Bu. Next, a resonance persistent azaally radical species (**197B**) and α‐*tert*‐alkyl radical species (**197C**) were formed. These radical species coupled together to produce **197.3**. Two different *tert*‐alkyl radicals generation in proximity to the redox active Cu metal center enabled efficient cross‐coupling reactions to construct contiguous tetrasubstituted carbon centers.

### 7.5 Dearomatization

Dearomative reaction is one of the most difficult reactions. Especially, dearomative *tert*‐alkylation is sterically and energetically difficult.

**Scheme 198:**
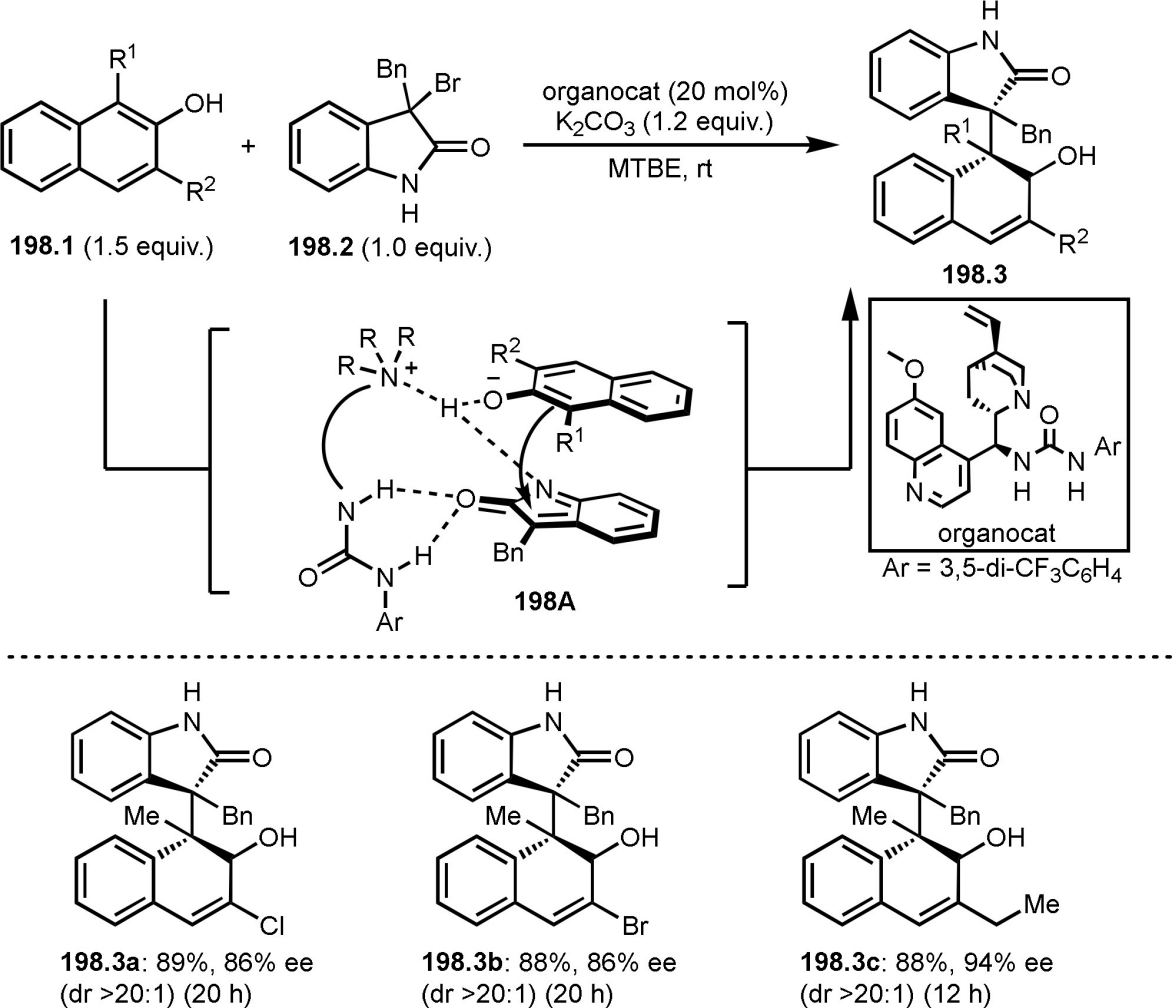
Organocatalyzed asymmetric dearomative addition of naphthol.

Wang's group reported the reaction of naphthols (**198.1**) with cyclic α‐bromocarboxamides (**198.2**) to construct vicinal all‐carbon quaternary stereocenters in the presence of a cinchona alkaloid derived urea (organocatalyst) (Scheme 198).^[371]^ 2H‐indol‐2‐one intermediate possessing a planar structure, which is a putative o‐azaxylylene intermediate, was generated at the first stage of this reaction. Nucleophilic naphthol anion and 2H‐indol‐2‐one intermediate are brought closer together by hydrogen bonding (**198A**) with the organocatalyst, and an asymmetric reaction proceeds. This interesting addition can be applied to the synthesis of various chiral cyclic compounds **(198.3 a**–**c**) with high ees.

### 7.6 Reduction

α‐Halocarbonyl compounds are converted to carbonyl compounds with chiral tertiary carbons by reduction reactions. However, this reduction reaction by radical reaction is very difficult to control chirality. Therefore, asymmetric reduction reactions are very rare. Here reductions with chiral tin hydride and enzymatic reductions will be introduced.

**Scheme 199:**
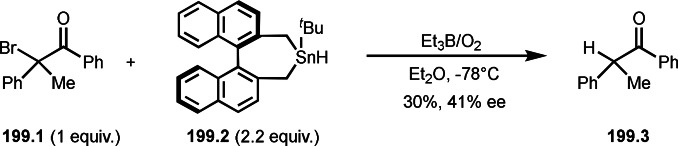
Asymmetric tin hydride reduction of α‐bromoketone.

To reduce C−X bonds to C−H bonds, tin hydride is known to be one of the most effective and convenient radical reaction methodologies. Curran's group reported the reaction of α‐bromoketone (**199.1**) with tin hydride (**199.2**) possessing a chiral binaphthyl fragment to produce 41 % ee of ketone (**199.3**) (Scheme 199).^[372]^ They examined AIBN as a radical initiator. The yield was improved to 70 %, but ee was 11 %. To obtain moderate ee, low temperature (−78 °C) was effective. Yields and ee were very low, but the results were very suggestive and state‐of‐the‐art for the time. Subsequently, various groups improved the chiral tin hydride reduction of α‐halocarbonyl compounds.^[373]^

**Scheme 200:**
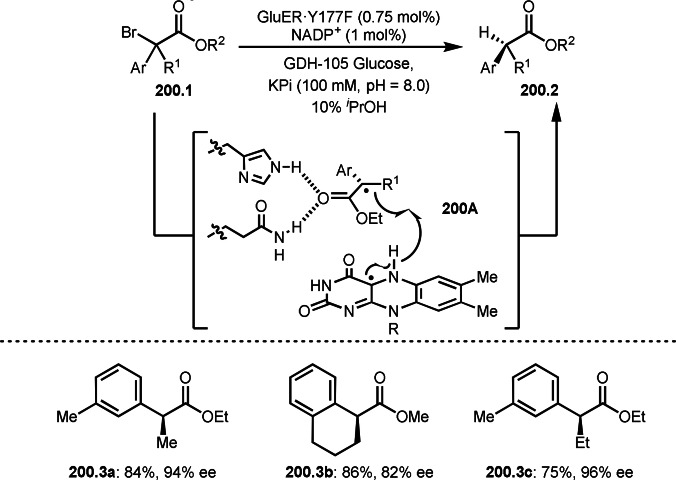
Asymmetric reduction of α‐bromocarbonyl compound by ene‐reductase.

**Scheme 201:**
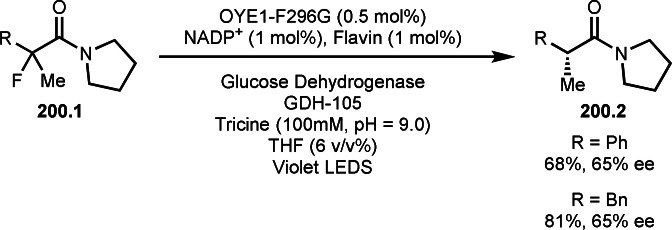
Photo‐enzymatic reduction.

C−X bonds of α‐halocarbonyl compounds ca be reduced by SmI_2_ ^[374a]^ and 1‐methyl‐3,6‐bis(trimethylsilyl)‐1,4‐cyclohexadiene.^[374b]^ These reactions are unique in that they do not use tin, but the corresponding asymmetric reactions cannot be performed. Hyster's group reported enzyme including flavin catalyzed asymmetric reduction of α‐bromocarbonyl compounds (**200.1**) to produce chiral esters (**200.2 a**–**c**) (Scheme 200).^[375]^ The reaction could be applied to a variety of substrates, and high selectivities were accomplished. Flavin is known as a single electron reductant in biological environment. Asymmetric recognition was achieved in a three‐dimensional reaction environment of flavin and enzyme (**200A**). Hyster's group further applied this reaction to develop the photo‐enzymatic reaction (Scheme 201).^[376]^ Merging photo‐reaction and enzyme reaction realized the asymmetric reduction of C−F bonds of α‐fluorocarboxamides (**201.1**), which gave chiral carboxamides (**201.2**).

## 8 Stereospecific Reaction

The S_N_2 reaction, a stereospecific reaction, is a well‐known reaction for converting chiral compounds and can be carried out without compromising the optical active carbons of the various secondary alkyl groups. There are many examples of the reaction with chiral *sec*‐alkyl halides via S_N_2 manner, whereas the corresponding *tert*‐alkyl halides are rare. Stereospecific reactions have recently become a hot topic in this field, but the reaction patterns are not yet satisfactory.^**[377]**^ Therefore, it is not well understood whether the stereospecific reaction of chiral tertiary alkyl halides is stereo‐retention or ‐inversion, as well as the development of an efficient methodology for stereospecific *tert*‐alkylation. In this section, a few reports on this topic using α‐bromocarbonyl compounds as a *tert*‐alkyl source are introduced.

**Scheme 202:**
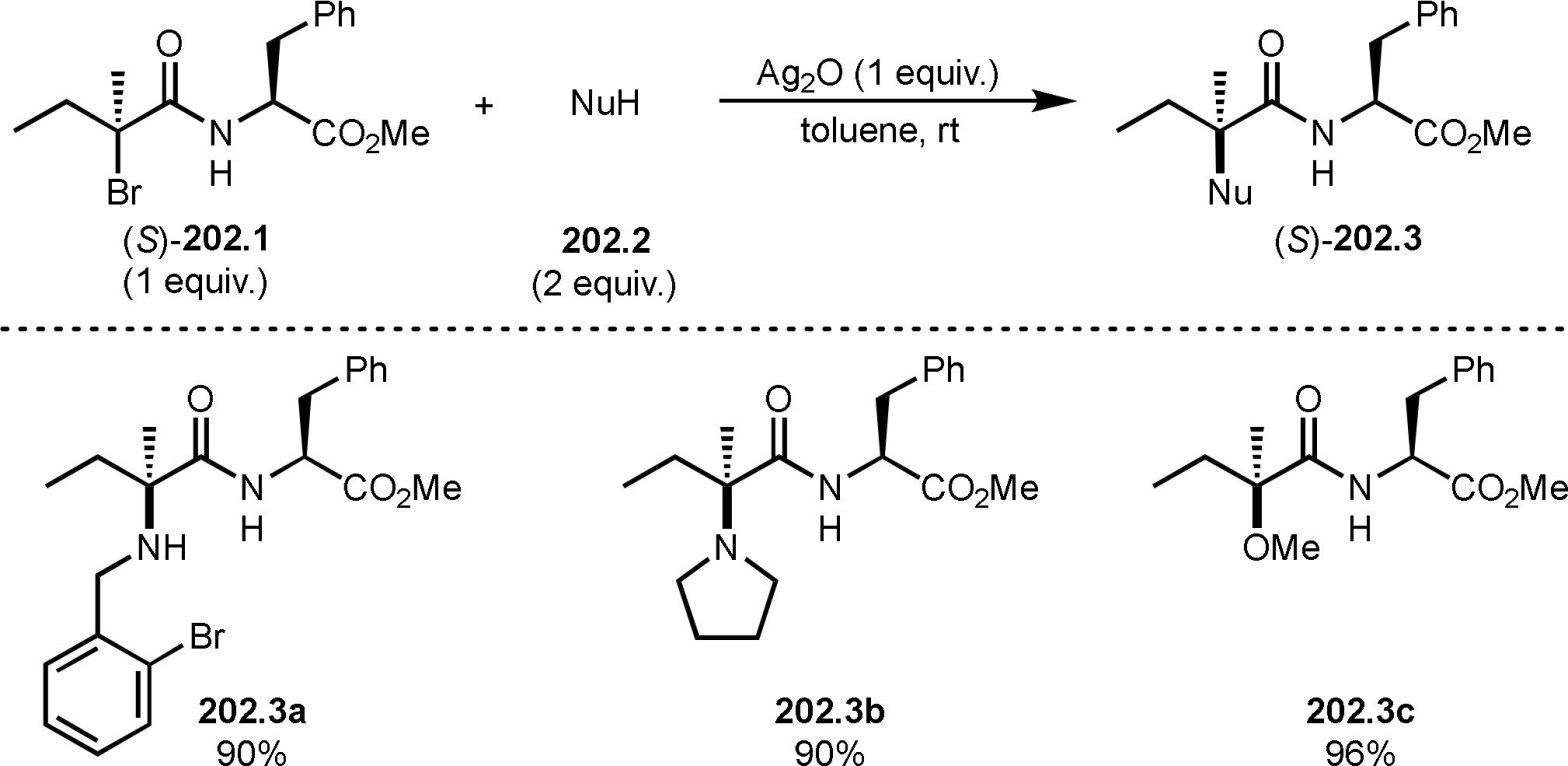
Silver‐mediated stereoretentive amination and etherification of α‐bromocarboxamide.

D′Angeli's group reported stereoretentive amination and etherification of (*S*)‐α‐bromocarboxamide (**202.1**) to produce (*S*)‐products (**202.3 a**–**c**) in the presence of Ag salt (Scheme 202).^[378a]^ Unlike the traditional S_N_2 reaction, this reaction proceeded in a retentive manner. The mechanism of this reaction is not clear, but an aziridinone like intermediate could be generated in this reaction.^[379]^ If aziridinone was involved in this reaction, the intramolecular S_N_2 reaction of **202.1** would produce aziridinone, followed by the intermolecular S_N_2 reaction between aziridinone and NuH to produce the stereoretentive product (**202.3**).

**Scheme 203:**
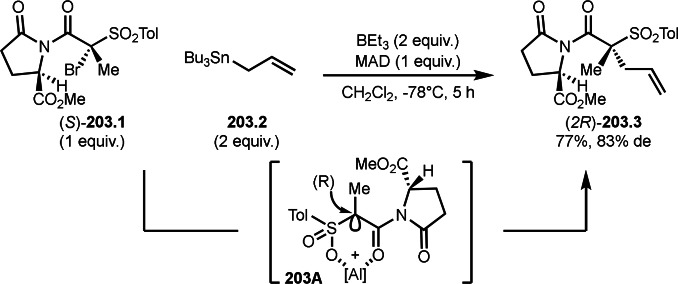
MAD‐mediated stereospecific allylation of α‐sulfonyl imides with allyl stannane.

**Scheme 204:**
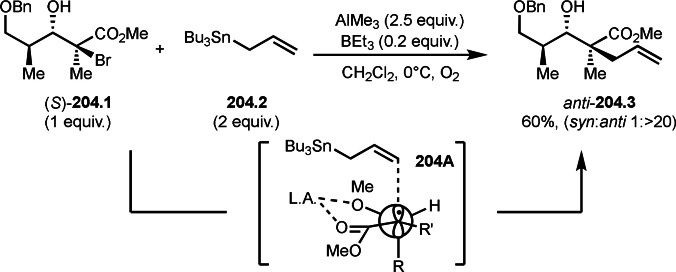
Me_3_Al‐mediated stereospecific allylation of α‐sulfonyl imides with allyl stannane.

Hiroi's group reported MAD (methylaluminum bis(2,6‐di‐tert‐butyl‐4‐methylphenoxide)) mediated stereospecific radical allylation of α‐sulfonyl imides with allyl stannane (Scheme 203).^[380]^ Two oxygens of α‐sulfonyl imide are suitable for the formation of six‐membered chelate intermediate (**203A**) with MAD. Whether the reaction is stereoretentive or inversive is determined by the absolute configuration of the asymmetric auxiliary group. Similar stereospecific radical reaction using allylation has been reported by Cardinal‐David and Guindon's group (Scheme 204).^[381a]^ In this reaction, *sec*‐alkyl alcohol moiety was used as a chiral auxiliary. Stereospecific reaction was accomplished through the Al‐chelated intermediate (**204A**). Different from simple radical allylation with allyl stannane,^[381b–e]^ this intermediate forms a planar structure and the reaction with the nucleophile proceeds from the top of **204A**.

**Scheme 205:**
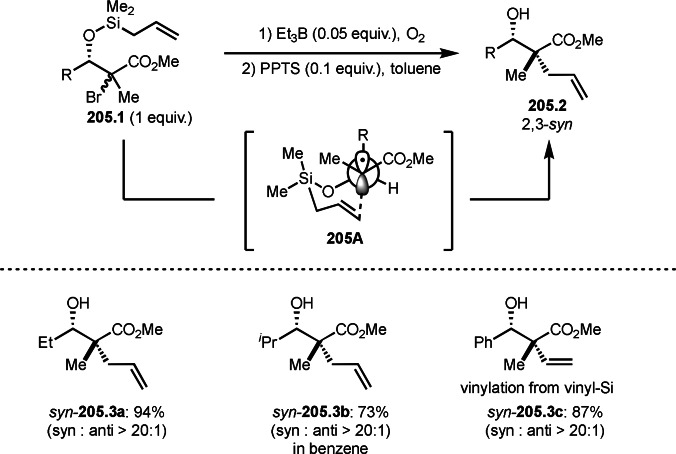
Intramolecular stereospecific allylation and vinylation.

**Scheme 206:**
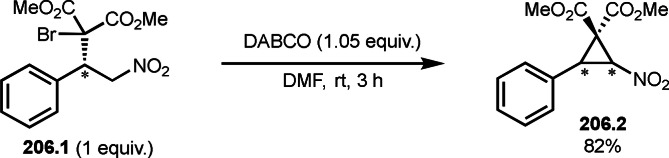
Intramolecular S_N_2 reaction.

Cardinal‐David and Guindon's group intramolecular stereospecific allylation or vinylation of silylated α‐bromocarbonyl compounds possessing silyl ether as a chiral auxiliary (Scheme 205).^[382]^ Radical reactions are difficult to control stereospecificity, but this was achieved by conducting intramolecular reaction via **205A**. In situ generated α‐*tert*‐alkyl radical species immediately reacted with the C−C double bond of **205.1** to produce 2,3‐*syn*‐**205.2** in good yields with high selectivity. Intramolecular stereospecific reaction is also effective to produce a chiral nitrocyclopropane (Scheme 206).^[383]^ This is a simple intramolecular S_N_2 reaction, but the reaction at *tert*‐carbon is rare.

**Scheme 207:**
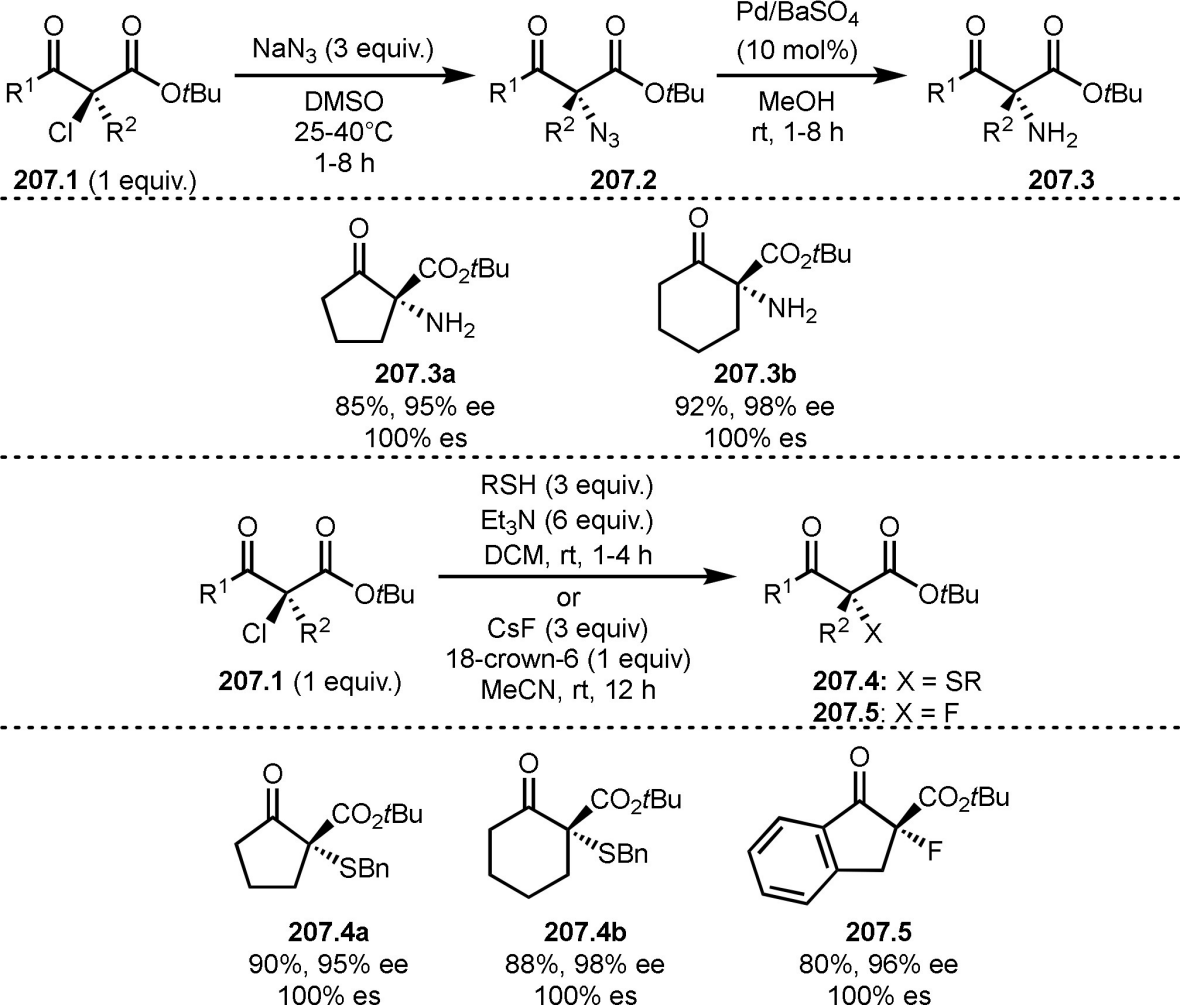
S_N_2 reactions of chloromalonate derivatives with azide, thiol, and fluoride.

**Scheme 208:**
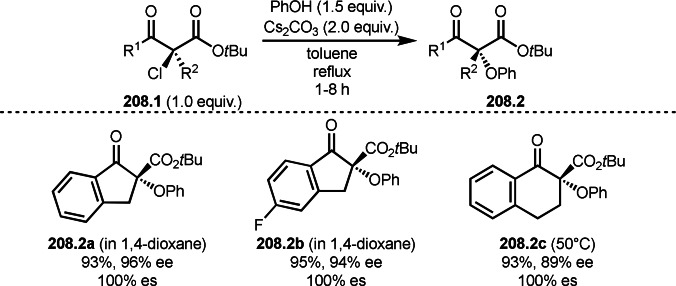
S_N_2 reactions of cyclic chloroketoesters with phenol.

**Scheme 209:**
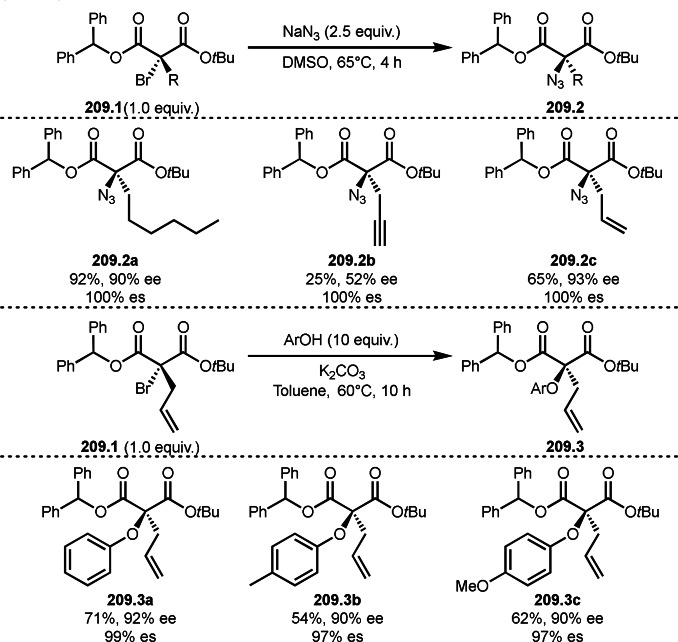
S_N_2 reactions of bromomalonate derivatives with azide and phenol.

Although α‐halocarbonyl compounds possessing a *tert*‐alkyl moiety are good electrophiles, the nucleophiles that can react with them are limited. On the other hand, 2‐halomalonate derivatives have two carbonyl groups and are more electrophilic than α‐halocarbonyl compounds. Therefore, they can react with a wide range of nucleophiles. Shibatomi's group reported S_N_2 reactions of chloromalonate derivatives (**207.1**) with azide, thiol, and fluoride to produce the corresponding products (**207.3**, **207.4**, and **207.5**) (Scheme 207).^[384]^ The reaction also occurred with cyclic chloroketoesters (**208.1**) (Scheme 208).^[385]^ Under their simple conditions, S_N_2 reactions successfully occurred at the chlorinated *tert*‐carbon. The all products were formed via the Walden inversion and high enantiospecificities (es: substrate ee/product ee). Similar reaction was accomplished by Park's group (Scheme 209).^[386]^

**Scheme 210:**
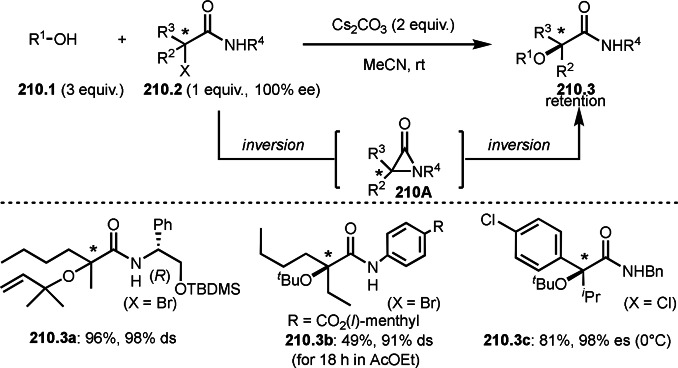
Stereoretentive substitution of α‐halocarboxamides with *tert*‐alkyl alcohols.

The traditional method for carrying out nucleophilic substitution is an S_N_2 or S_N_1 reaction with an alkyl halide. This traditional methodology is reliable and has broad applicability for the synthesis of various aliphatic compounds. However, there are serious disadvantages to these reaction systems: the S_N_2 reaction is not suitable for *tert*‐alkyl halides and bulky nucleophiles such as tertiary alkyl alcohols, and the S_N_1 reaction is not suitable for stereospecific reactions to produce a product without loss of substrate chirality. Nishikata's group reported that enantiopure α‐halocarboxamides (**210.2**) reacted with *tert*‐alkyl alcohols (**210.1**) to produce highly congested ethers (**210.3 a**–**c**) possessing two different *tert*‐alkyl moieties (Scheme 210).[^387^, ^388^] The reaction smoothly occurred at room temperature or even at 0 °C. Interesting point of this reaction is that the all products are stereoretentive. If this was S_N_2 reaction described above, the products should be inversive. They suggested highly electrophilic aziridinone (**210A**) as an intermediate, in which double S_N_2 reactions occurred to produce retentive product (**210.3**).

**Scheme 211:**
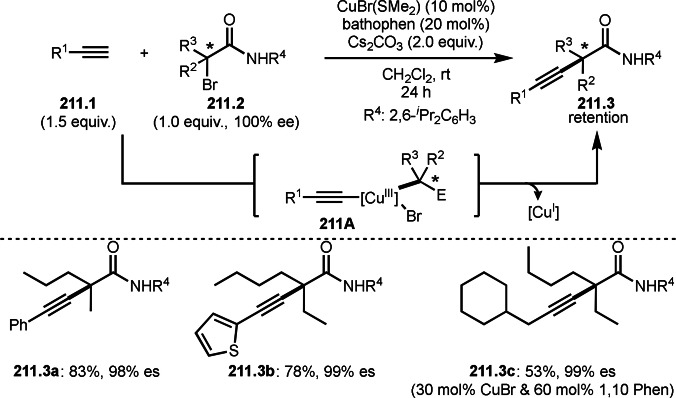
Stereospecific alkynylation of α‐bromocarboxamide.

Nishikata's group also found the stereospecific Cu catalyzed cross‐couplings of alkynes (**211.1**) with enantiopure α‐bromocarboxamides (**211.2**) (Scheme 211).^[389]^ The products obtained were observed to be stereoretentive. This reaction is only suitable for α‐bromocarboxamides, not esters. Therefore, they suspected stereospecific substitution of aziridinones with alkynes, but observed no nucleophilic alkynylation. Carboxamides may play an important role in copper coordination. On the other hand, a Cu^I^‐acetylide complex smoothly reacted with **211.2** to give high es of **211.3** via **211A**. The drawback of this reaction is the substituent on the nitrogen of carbxamide group. Under catalytic conditions, 2,6‐^*i*^PrC_6_H_3_ group have to be used, and other carbon functionalities were not effective. However, α‐bromocarboxamides with carbon functional groups other than 2,6‐ ^*i*^PrC_6_H_3_ group reacted smoothly with stoichiometric amounts of copper acetylide to produce the corresponding **211.3** in a stereoselective manner.

**Scheme 212:**
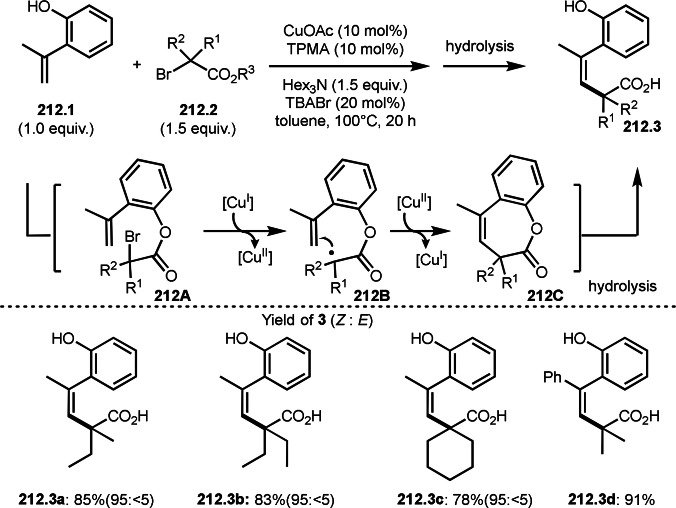
Synthesis of highly congested *Z* (*cis*)‐olefins.

Although it may not fit into this section, the following reactions also fall into this category in terms of giving rise to a stereospecific product. Nishikata's group reported the synthesis of highly congested *Z* (*cis*)‐olefins (**212.3 a**–**d**) possessing a *tert*‐alkyl group (Scheme 212).^[390]^ The reaction of *ortho*‐hydroxysubstituted styrenes (**212.1**) with α‐bromocarbonyl compounds (**212.2**) in the presence of a Cu catalyst gave seven‐membered ring (**212C**) via esterification followed by radical cyclization (**212A** and **212B**). The desired *Z* (*cis*)‐olefin (**212.3**) was obtained after the hydrolysis of **212C**. This esterification/cyclization realized perfect selectivities to obtain the products (**212.3**).

## 9 Divergent Reaction

α‐Bromocarbonyl compounds can be converted to ions, radicals, and organometallic species by combining catalysts and additives. This can be used to perform a divergent reaction to give different products from a single substrate. The divergent reaction to synthesize a wide variety of compounds under different reaction conditions is very important technology from the viewpoint of molecular diversity. However, synthesis of sterically congested molecules using α‐bromocarbonyl compounds as a *tert*‐alkyl source is still rare due to the difficulty in controlling their reactivity. In this section, recently reported 5 examples will be introduced.

**Scheme 213:**
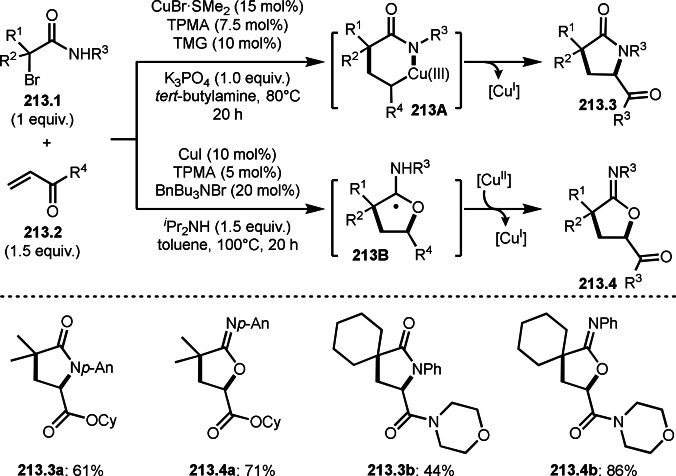
*N*‐ vs *O*‐Cyclization.

Changing existing reaction patterns to other patterns by tuning the catalyst system suggests a new elemental step in the catalytic cycle. Nishikata's group discovered that the *N*‐ *O*‐nucleophilicity of carboxamides was controllable with an optimized Cu‐catalyst system (Scheme 213).^[391]^ The carboxamide functional group is extremely interesting because it contains both nitrogen and oxygen nucleophiles, making carboxamides so‐called ambident nucleophiles. Although there are several reactions with carboxamide, a general methodology to control the reactivities of the nitrogen and oxygen nucleophiles of a carboxamide group has not yet been established. The reaction of α‐bromocarboxamides (**213.1**) and acrylates (**213.2**) gave cyclization products (lactam **213.3** or lactone **213.4**). Cu/K_3_PO_4_/TMG (1,1,3,3‐Tetramethylguanidine) (strong base conditions) system gave lactam **213.3**, whereas Cu/^*i*^Pr_2_NH (weak base conditions) system gave lactone **213.4**. The key to the success of this reaction system is the use of a copper catalyst with a suitable base. Strongly basic conditions (enabling NH deprotonation) afford the lactam (*N*‐attack) via **213A**, whereas weakly basic conditions produce the iminolactone (*O*‐attack) via the alkyl radical intermediate (**213B**). Radical and organometallic reactions diverged in this reaction, depending on the reaction conditions.

**Scheme 214:**
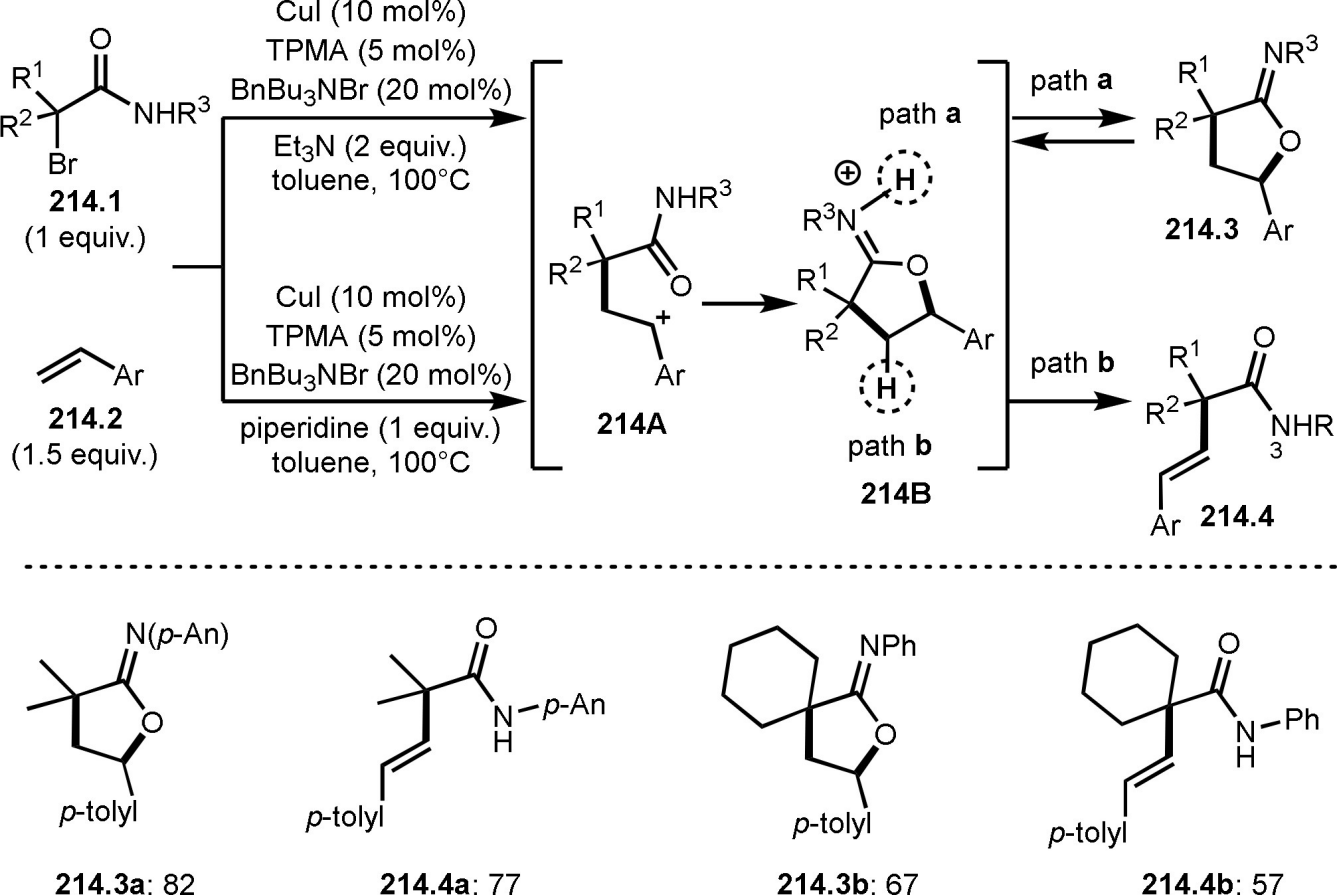
Cu‐catalyzed ATRS vs cyclization.

Nishikata's group next found a Cu catalyst‐amine base system that enables a perfect switch between iminolactonization and Heck like olefination in the reaction of α‐bromoamides and styrenes (Scheme 214).^[392]^ In this study, they discovered that α‐bromocarboxamides (**214.1**) and styrenes (**214.2**) underwent iminolactonization reactions (carbo‐oxygenation), in which simultaneous C−C and C−O bond formation occurred in the presence of a copper catalyst with triethylamine as the base. Conversely, Heck like olefination reactions (ATRS) occurred in the presence of a copper catalyst with piperidine as the base. Control experiments and theoretical calculations revealed that this divergent process is enabled by the in situ generation of ammonium acids (R_3_NH^+^). When 2 equiv. of amine was used, excess amine captured the proton of intermediate **214B** to produce a desired lactam (**214.3**) (path a). On the other hand, when 1 equiv. of amine was used, protolysis occurred to give the olefination product (**214.4**) (path b).

**Scheme 215:**
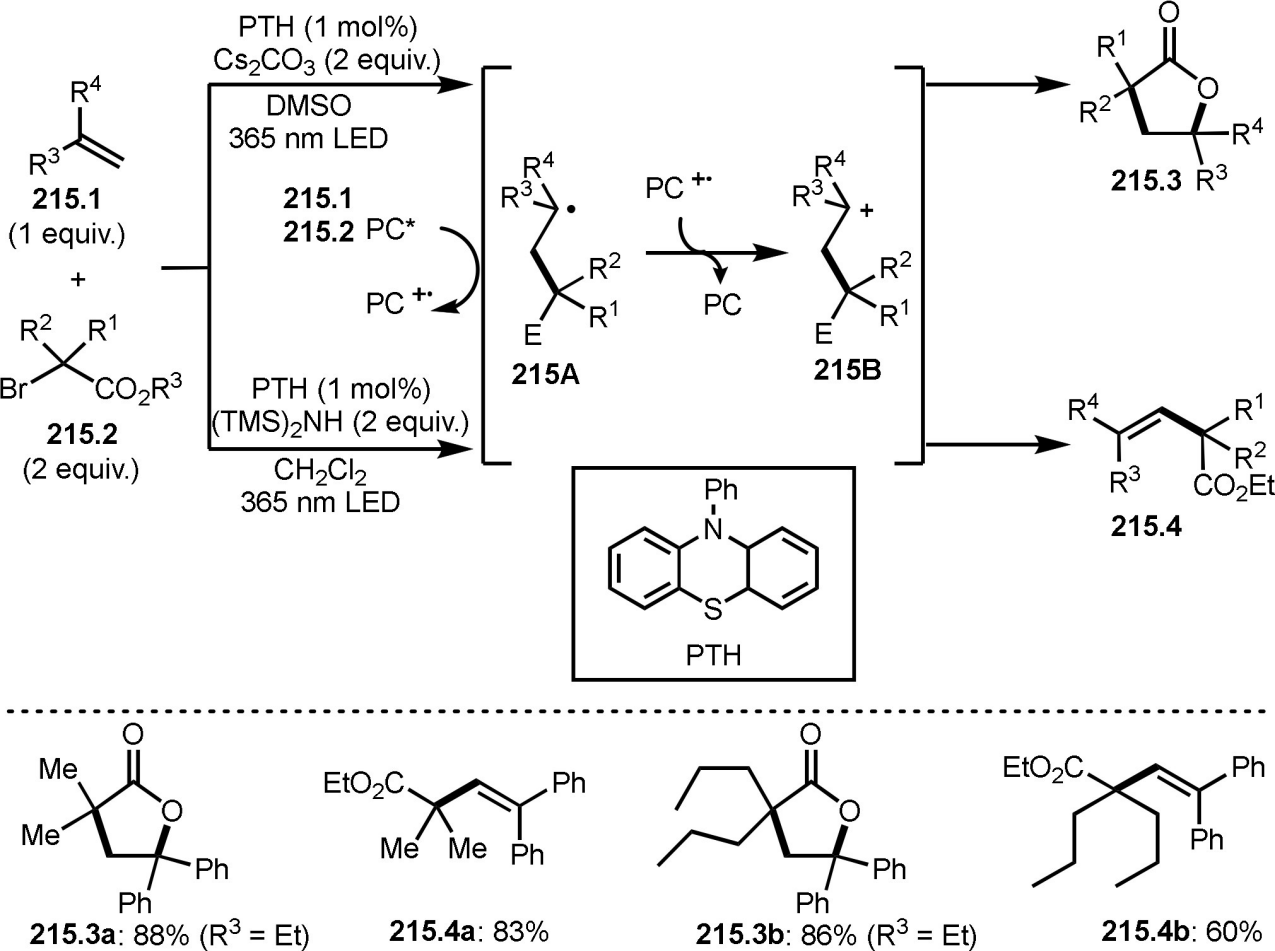
PTH‐catalyzed ATRS vs Cyclization.

Nishikata's group also controlled ATRS and lactonization reaction between styrenes (**215.1**) and α‐bromoesters (**215.2**) under photocatalyst conditions (Scheme 215).[^393^, ^394^] They achieved functionalized alkylation of styrene by PTH‐catalyzed ATRS reaction using cyclic and acyclic α‐bromoesters and ‐nitro compounds and perfluoroalkyl halides as alkyl sources, with 365 nm LED light illumination. PTH/Cs_2_CO_3_/DMSO system gave lactam **215.3**, whereas PTH/Cs_2_CO_3_/CH_2_Cl_2_ system gave lactone **215.4**. The key to the success of this reaction system is not clear, but it is possible that DMSO stabilizes the cationic intermediate (**215B**) and promotes cyclization.

**Scheme 216:**
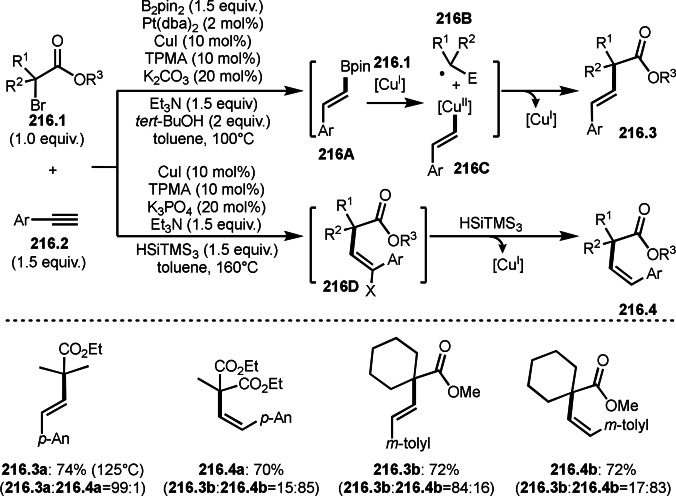
*E*‐ vs *Z*‐Olefination.

Hydroalkylation of alkynes is an indispensable reaction to synthesize alkylated alkenes. Although there have been many reports on hydroalkylation reactions, stereoselective hydroalkylation reactions have not yet been fully investigated. Especially, the addition of functionalized tertiary‐alkyl groups is difficult owing to their steric hindrance. Therefore, controlling trans‐ and cis‐hydroalkylations is challenging. Nishikata's group discovered a Cu‐catalyzed divergent reaction of α‐bromoesters (**216.1**) and alkynes (**216.2**), which gave trans‐ and cis‐hydroalkylation products (**216.3** and **216.4**) (Scheme 216).^[395]^ The reaction via borylation gave trans‐hydroalkylations (**216.3**), whereas the reaction via reduction with silane gave cis‐hydroalkylations (**216.4**). In the synthesis of **216.3**, reductive borylation of alkyne occurred to give **216A** in a *trans*‐manner. Transmetalation between **216A** and Cu salt occurred to give **216C**. **216C** smoothly coupled with **216B** to produce **216.3** via Suzuki‐Miyaura type coupling. On the other hand, ATRA of **216.1** and **216.2** occurred to give a haloalklylated alkene intermediate (**216D**) in an *E*‐manner. **216D** then reduced with silane to produce **216.4**.

**Scheme 217:**
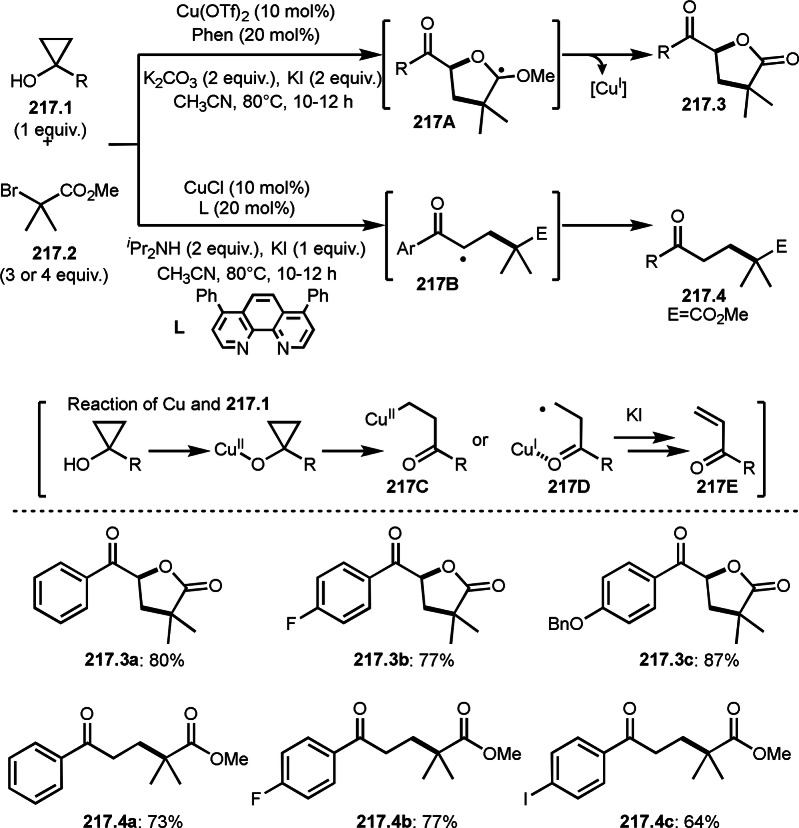
Addition vs cyclization.

Dai's group reported Cu‐catalyzed divergent reaction of cyclopropanols (**217.1**) and α‐bromoester (**217.2**) to give lactone (**217.3**) and β‐*tert*‐alkylated ketone (**217.4**) (Scheme 217).^[396]^ In the presence of K_2_CO_3_ the reaction yielded the γ‐lactones **217.3**, whereas the δ‐ketoester **217.4** is presumably derived by reductive quenching of **217B** with the internal amine component. **217.1** reacted with a Cu salt to give **217C** or **217D**, which generate **217E**. The resulting **217E** reacted with α‐*tert*‐alkyl radical species to give **217A** (radical addition followed by cyclization) or **217B**. An amine is considered to be a reducing agent. Therefore, in situ generated **217B** is reduced with amine to give **217.4**. But **217B** undergoes a cyclization to give **217A**, and **217.3** is formed.

## 10 Application to Total Synthesis

**Scheme 218:**
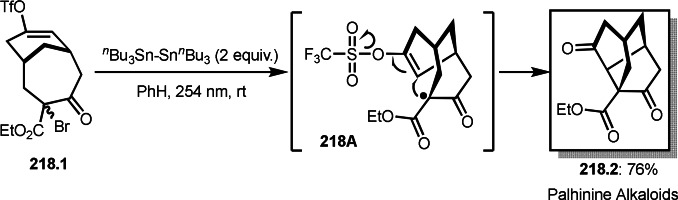
Palhinine alkaloid.

**Scheme 219:**
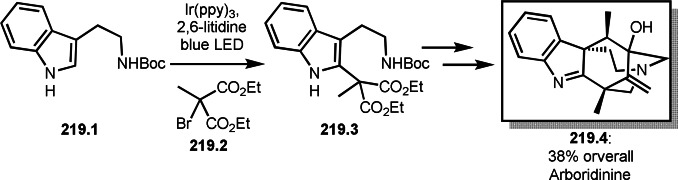
Arboridinine.

**Scheme 220:**
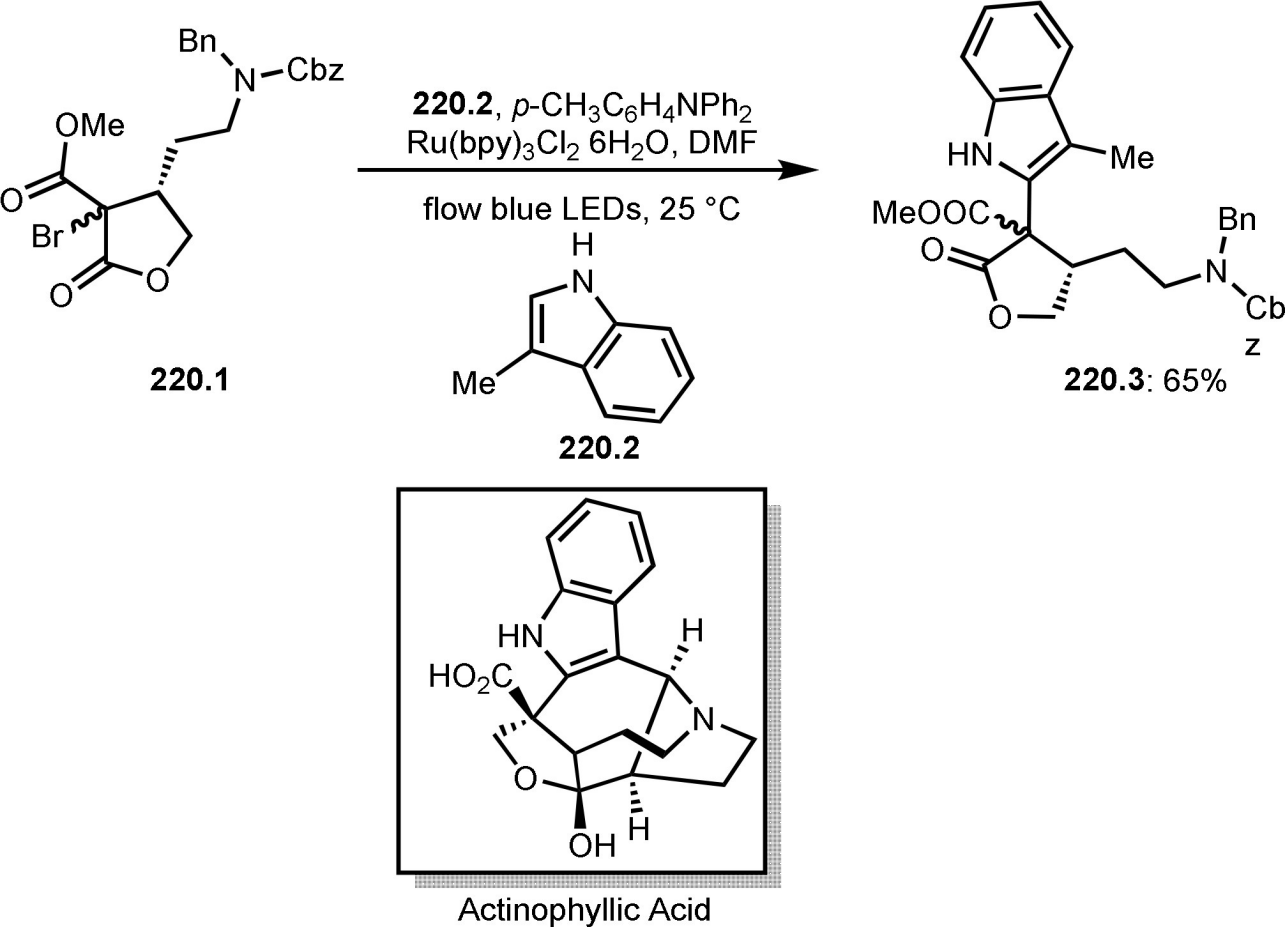
Actinophyllic acid.

**Scheme 221:**
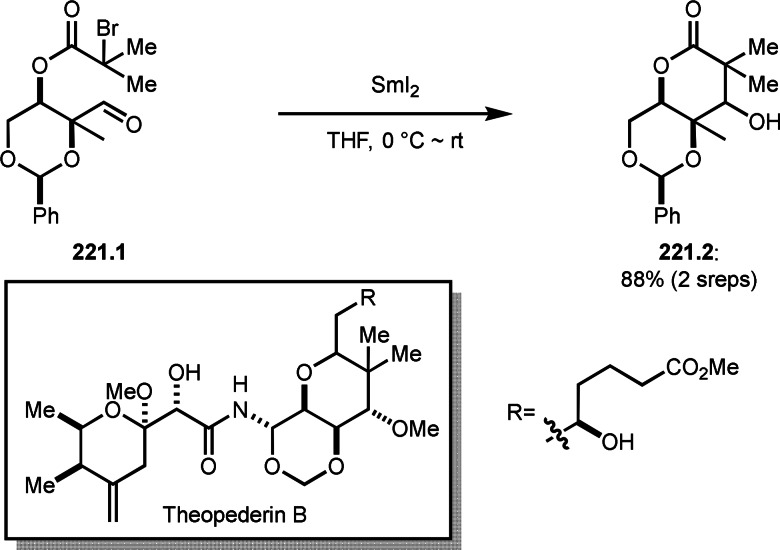
Theopederin B.

**Scheme 222:**
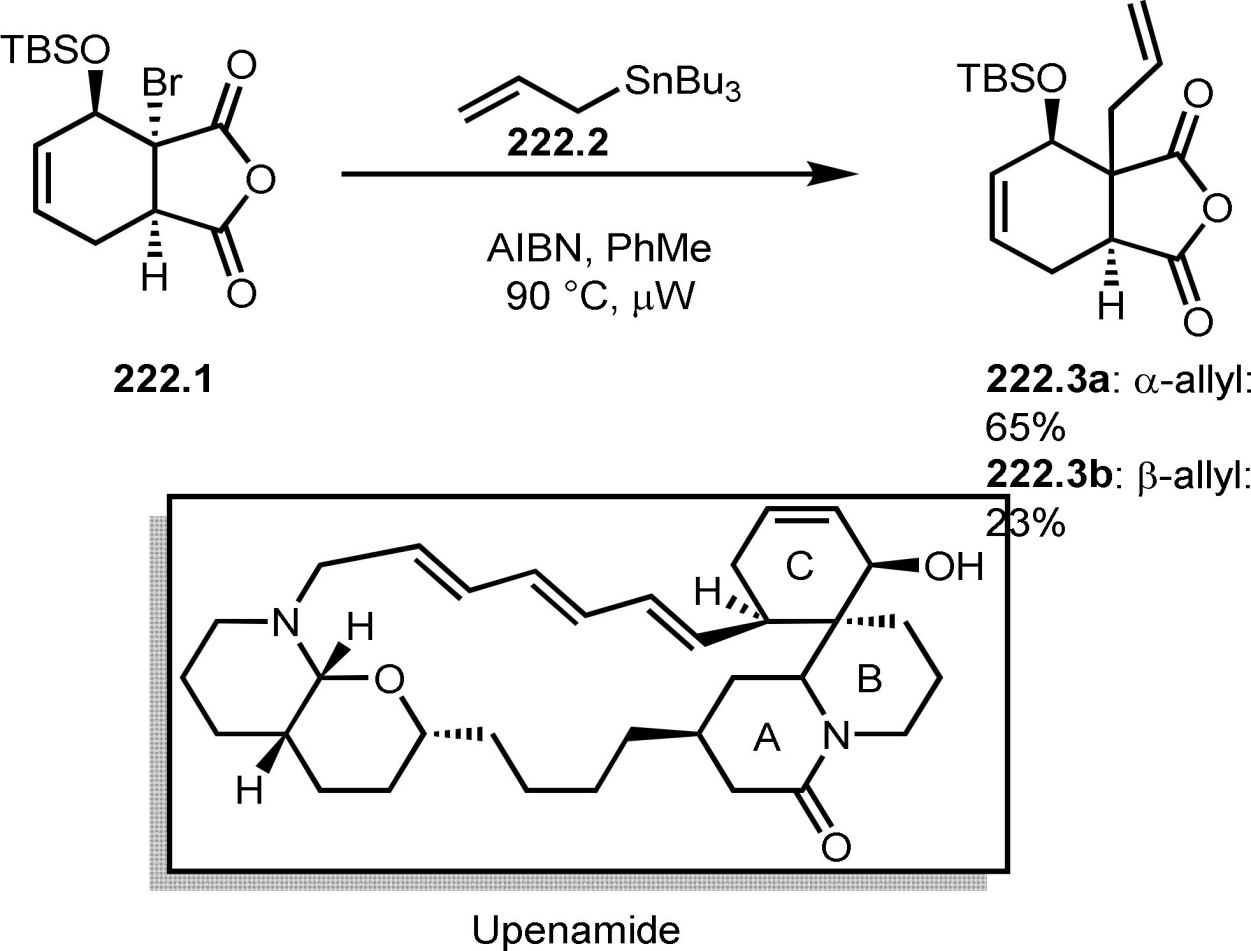
Upenamide.

In the above sections, we have shown that α‐bromocarbonyl compounds can be deployed in a variety of reaction modes, including radical, ionic, and organometallic reactions. Each report also mentions development into useful substances, but they are not the main target. It is known that natural products and other useful molecules containing quaternary carbons are of great synthetic value, and several review articles have been published.^[397]^ α‐Bromocarbonyl compounds are excellent reagents because they readily provide a tertiary alkyl group, and after the reaction, the carbonyl group can be easily converted to the desired quaternary carbon. For example, tin mediated radical reaction of **218.1** provided Palhinine alkaloid via intramolecular radical cyclization (Scheme 218).^[398]^ The key reaction in Arboridinine (Scheme 219)^[399]^ and Actinophyllic acid (Scheme 220)^[400]^ synthesis employed aromatic C−H α‐*tert*‐alkylation of **219.1** or **220.1** with **219.2** or **220.2**. Sm‐mediated reactions allowed **221.1** nucleophilic radical 1,2‐addition reactions, finally yielding Theopedrine B (Scheme 221).^[401]^ On the other hand, allyl tin reaction in the presence of AIBN underwent radical allyl coupling to produce Upenamide (Scheme 222).^[402]^

## 11 Reaction of (Aza)‐Oxyallyl Cation

### 11.1 Oxyallyl Cation

**Scheme 223:**
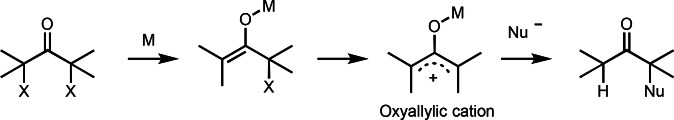
Metal‐mediated oxyallyl cation reaction.

Another important reaction mode in understanding the reactivity of α‐halocarbonyl compounds is the oxyallyl cation. oxyallyl cation is a well‐known chemical species and can be generated from α,α’‐dihalocarbonyl compounds by the action of metal Lewis acids (Scheme 223).[^403^, ^404^, ^405^, ^406^, ^407^, ^408^, ^409^, ^410^, ^411^, ^412^, ^413^, ^414^, ^415^, ^416^, ^417^, ^418^, ^419^, ^420^] The resulting oxyallyl cations can react with nucleophiles or unsaturated bonds, including C−O and C−C, to synthesize a variety of *tert*‐alkylated cyclic and acyclic carbonyl compounds.

**Scheme 224:**
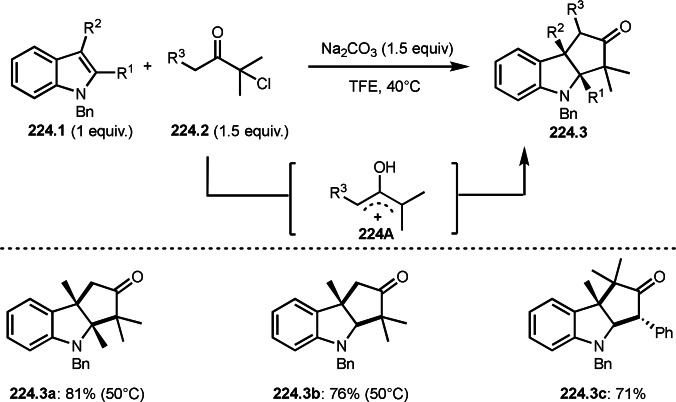
Base promoted oxyallyl cation reaction.

Recently, Wu's group reported that oxyallyl cations can be generated from the corresponding α‐chlorocarbonyl compounds (**224.2**) simply by adding a base (Scheme 224).[^421^, ^422^] In this case, the resulting oxyallyl cations underwent diastereoselective dearomative indole [3+2] cycloaddition reactions with indoles (**224.1**) to produce cyclopenta‐fused indolines (**224.3 a**–**c**) in good yields. 
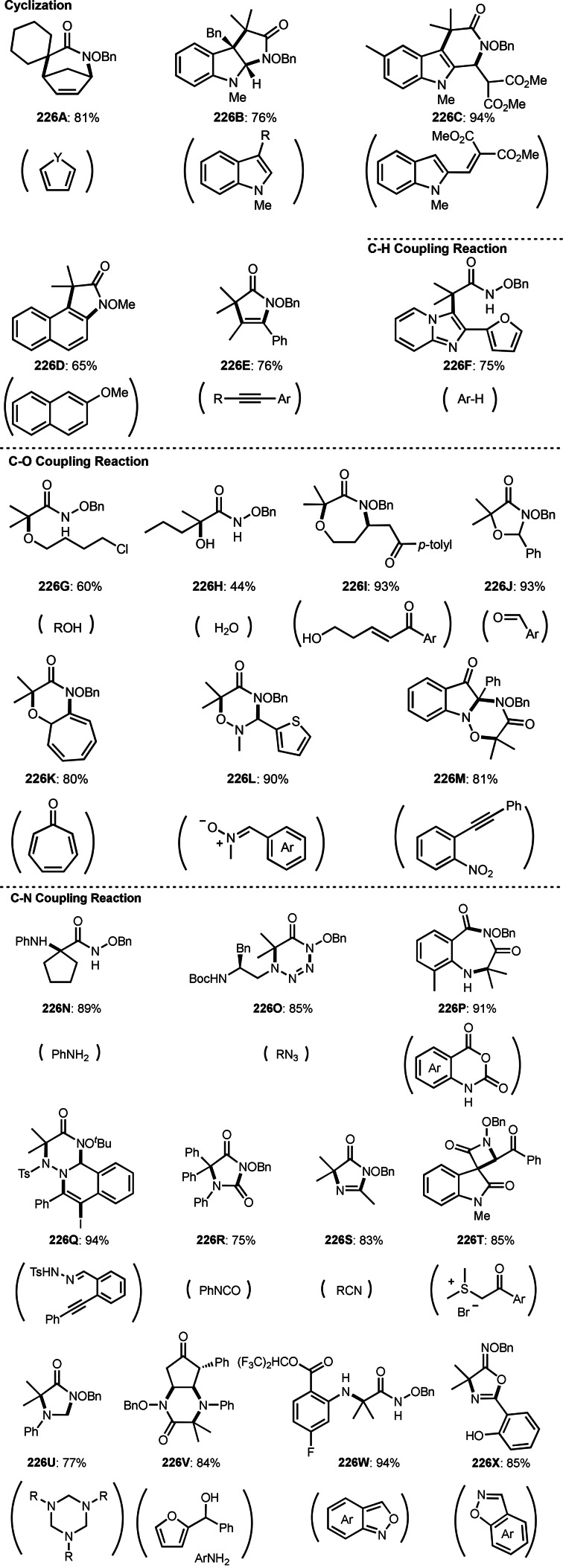


### 11.2 Aza‐Oxyallyl Cation

**Scheme 225:**
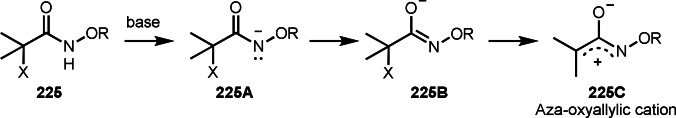
Generation of aza‐oxyallyl cation.

α‐halocarboxamides (**225**) possessing an alkoxyamine moiety can generate aza‐oxyallyl cation (**225C**) in the presence of a base (Scheme 225). If there is no alkoxyamine moiety in α‐halocarboxamides, no aza‐oxyallyl cation is produced. This methodology can be used to synthesize a variety of acyclic and cyclic compounds possessing carboxamides. The methodology is very simple but powerful. Recently several groups reported the aza‐oxyallyl cation reactions.

**Scheme 226:**
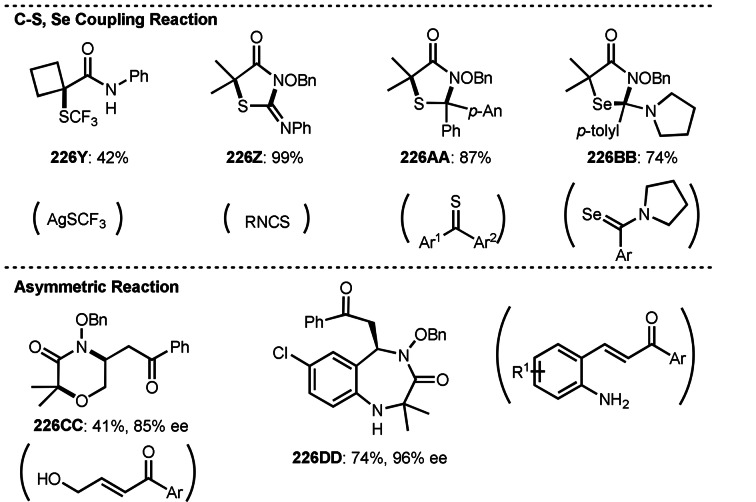
Aza‐oxyallyl cation reactions.

A nice review has been written by Jeffrey in which all the skeletons are presented.^[423]^ Therefore, for the reader‘s convenience, the main structures involved in *tert*‐alkylation are summarized in a table (Scheme 226). The aza‐oxyallyl cation is produced in all reaction systems by the reaction of α‐halocarboxamides possessing an alkoxyamine moiety with a base (amine, M_2_CO_3_, ^*t*^BuOK) in HFIP, CH_2_Cl_2_ or CF_3_C_6_H_5_ mostly at room temperature. This simple operation made the following reactions possible: Cyclization (**226A**,^[424]^ **226B**,^[425]^ **226C**,^[426]^ **226D**,^[427]^ **226E** ^[428]^), C−C coupling reaction (**226F** ^[429a]^), C−O coupling reaction (**226G**,^[430]^ **226H**,^[431]^ **226I**,^[432]^ **226 J**,^[433a]^ **226 K**,^[434a]^ **226 L**,^[435a]^ **226 M** ^[436]^), C−N coupling reaction (**226 N**,^[437]^ **226O**,^[438a]^ **226P**,^[439a]^ **226Q**,^[440a]^ **226R**,^[441]^ **226S**,^[442]^ **226T**,^[443a]^ **226 U**,^[444]^ **226 V**,^[445]^ **226 W**,^[446a]^ **226X** ^[447]^), C−S, Se coupling reaction (**226Y**,^[448]^ **226Z**,^[449]^ **226AA**,^[449]^ **226BB** ^[450]^), asymmetric reaction (**226CC**,^[451a]^ **226DD** ^[452]^) (Scheme 226).

## 12 Miscellaneous Reactions

### 12.1 C−S and C−P Bond Formations

**Scheme 227:**
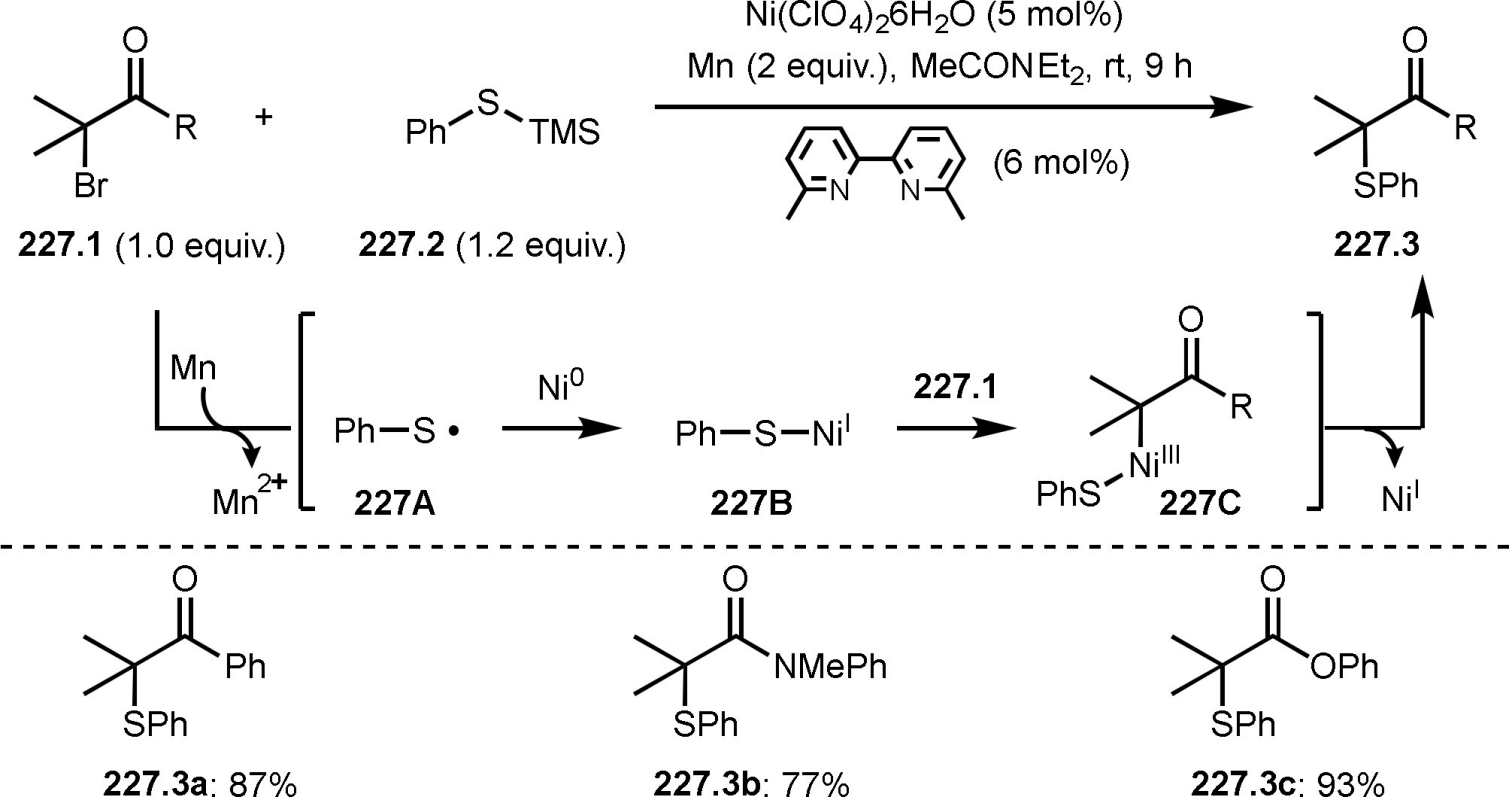
Reaction with phenylthiosilane.

Arylthiosilanes are a good sulfur donor to synthesize *tert*‐alkyl aryl thioethers. Lv, He and Li's group reported the reaction of α‐bromocarbonyl compounds (**227.1**) and phenylthiosilane (**227.2**) in the presence of Ni catalyst to produce *tert*‐alkyl phenyl thioethers (**227.1 a**–**c**) at room temperature (Scheme 227).^[453]^ Phenylthiosilane reacted with Mn to form sulfur‐centered radical (**227B**) or directly with Ni to form PhS−Ni complex (**227B**) by oxidative addition. **227B** then reacted with **227.1** to give **227C**. After reductive elimination of **227C**, **227.3** was produced.

**Scheme 228:**
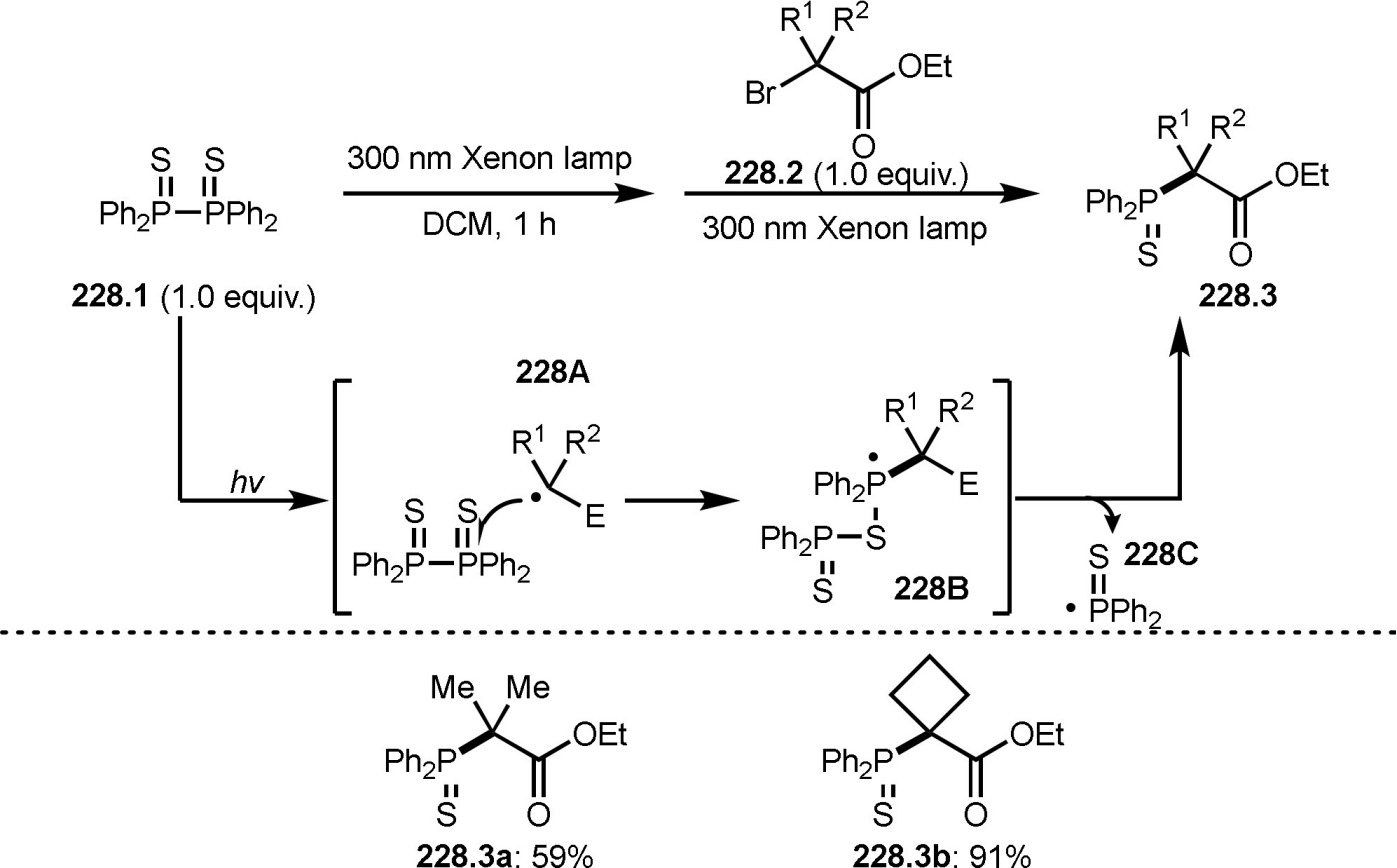
Reaction with tetraphenyldiphosphine disulfide

A P=S double bond is a good α‐*tert*‐alkyl radical acceptor. Kawaguchi and Ogawa's group reported the reaction of tetraphenyldiphosphine disulfide (**228.1**) with α‐bromocarbonyl compounds (**228.2**) to produce *tert*‐alkyl phosphine sulfides (**228.3 a**,**b**) under photo‐irradiation (Scheme 228).^[454]^ Upon photoirradiation, an α‐*tert*‐alkyl radical (**228A**) was generated from **228.2**. **228A** then added to P=S bond of **228.1** to form phosphorus‐centered radical (**228B**). Finally, **228B** released **228C** to produce **228.3**.

**Scheme 229:**
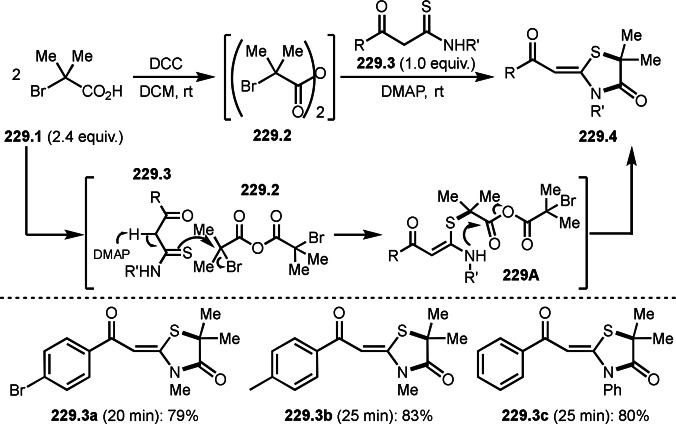
Cascade C−S/C−N bonds formation.

Synthesis of sterically congested heterocycles from α‐halocarbonyl compounds is very difficult. This is because heterocyclic rings and their starting materials can affect the reactivity of radical initiators and α‐*tert*‐alkyl radicals. Singh's group reported DMAP‐promoted cyclization reaction of α‐bromocarboxylic acid (**229.1**) with β‐ketothioamides (**229.3**) to produce 1,3‐thiazolidin‐4‐ones (**229.4**), in which cascade C−S/C−N bonds formation occurred (Scheme 229).^[455]^ In this reaction, the ionic reaction was used to form a heterocyclic ring, and **229.1** acted as an electrophile rather than a radical source. The reaction first generated anhydride (**229.2**) of **229.1**. Next, the anion of **229.3** formed from the reaction of **229.3** with DMAP attacked **229.2** to give **229A**. The resulting **229A** immediately underwent intramolecular cyclization to produce **229.4**.

### 12.2 Reaction with Alkyl Gallium

**Scheme 230:**
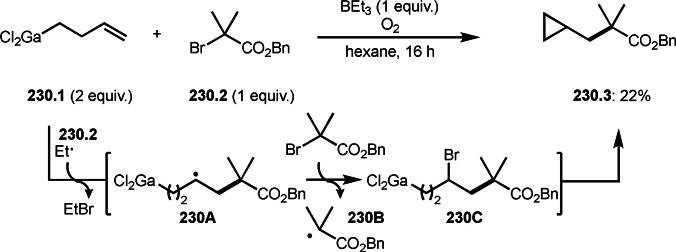
A combined α‐*tert*‐radical reaction and alkylgallium reaction.

Oshima's group reported the combination of α‐*tert*‐radical reaction and alkylgallium reaction (Scheme 230).^[456]^ The reaction of alkylgallium (**230.1**) with α‐bromoester (**230.2**) in the presence of Et_3_B/O_2_ gave cyclopropylalkylated quaternary carbon compound (**230.3**) in 22 % yield. Although the yield was low, this is an important example of a combined radical and organometallic reaction. The reaction of **230.2** with Et_3_B/O_2_ gave α‐*tert*‐radical which underwent ATRA to give **230C**. Finally, an intramolecular alkylgallium attack on the C−Br bond of **230C** produced **230.3**.

### 12.3 C−H Reactions

**Scheme 231:**
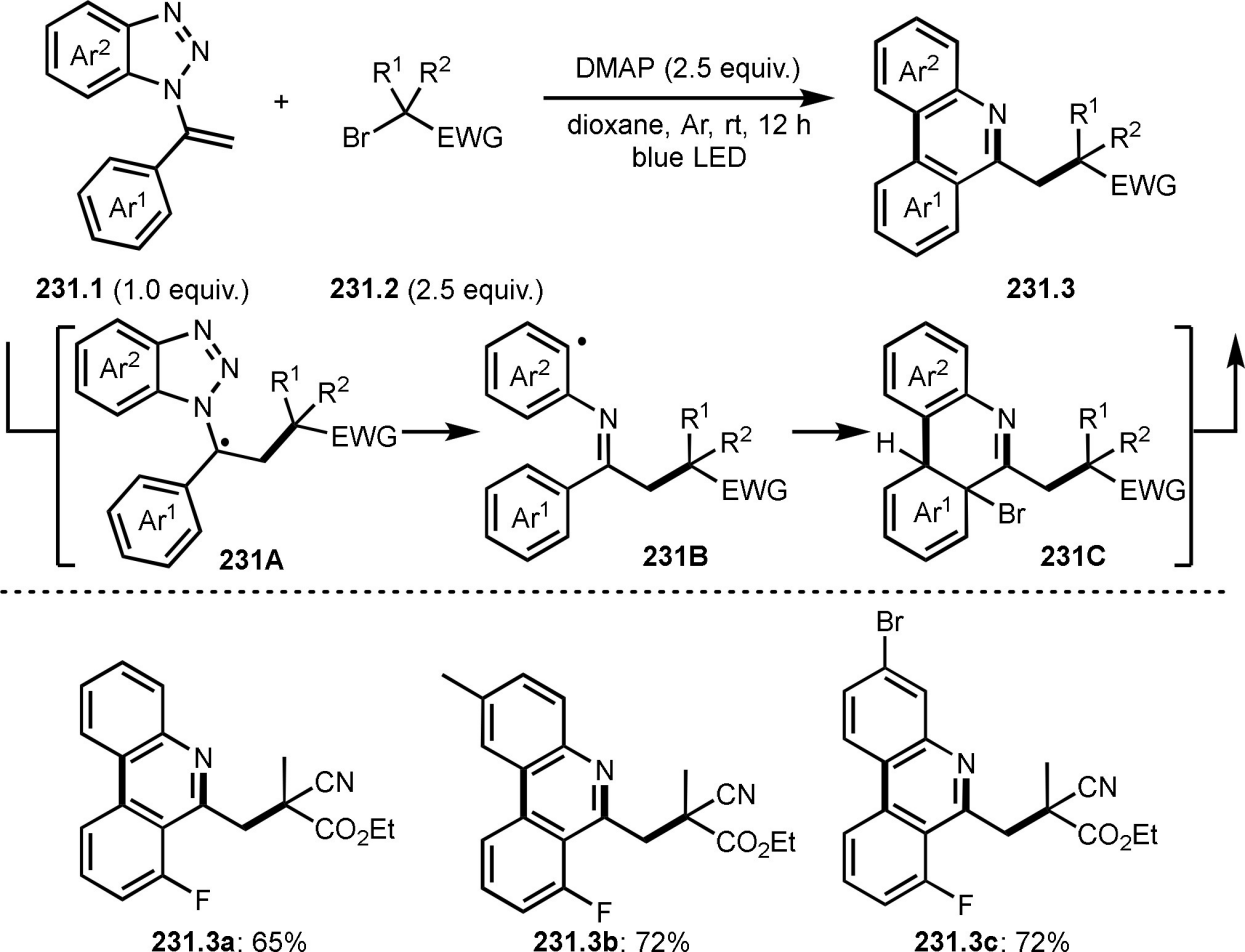
Visible light‐promoted radical‐mediated ring‐opening/cyclization.

A C−H cyclization reaction, in which the dissociation of the C−N bond and the formation of the C−C bond occur simultaneously, was reported by Zhao and Li's group (Scheme 231).^[457]^ The reaction of vinyl benzotriazoles (**231.1**) with 2‐bromomalononitriles (**231.2**) produced functionalized phenanthridines (**231.3 a**–**c**) under photo‐irradiation. **231.2** easily generated α‐*tert*‐alkyl radical species upon photo‐irradiation. No radical initiators, such as a metal catalyst, organocatalyst and photocatalyst, were required. The resulting α‐*tert*‐radical species added to **231.1** to give **231A**. Next, the radical ring‐opening reaction of **231A** proceeded, producing an aryl radical (**231B**) with the generation of N_2_. **231B** then underwent dearomatization to give **231C**, which underwent re‐aromatization to give **231.3**.

**Scheme 232:**
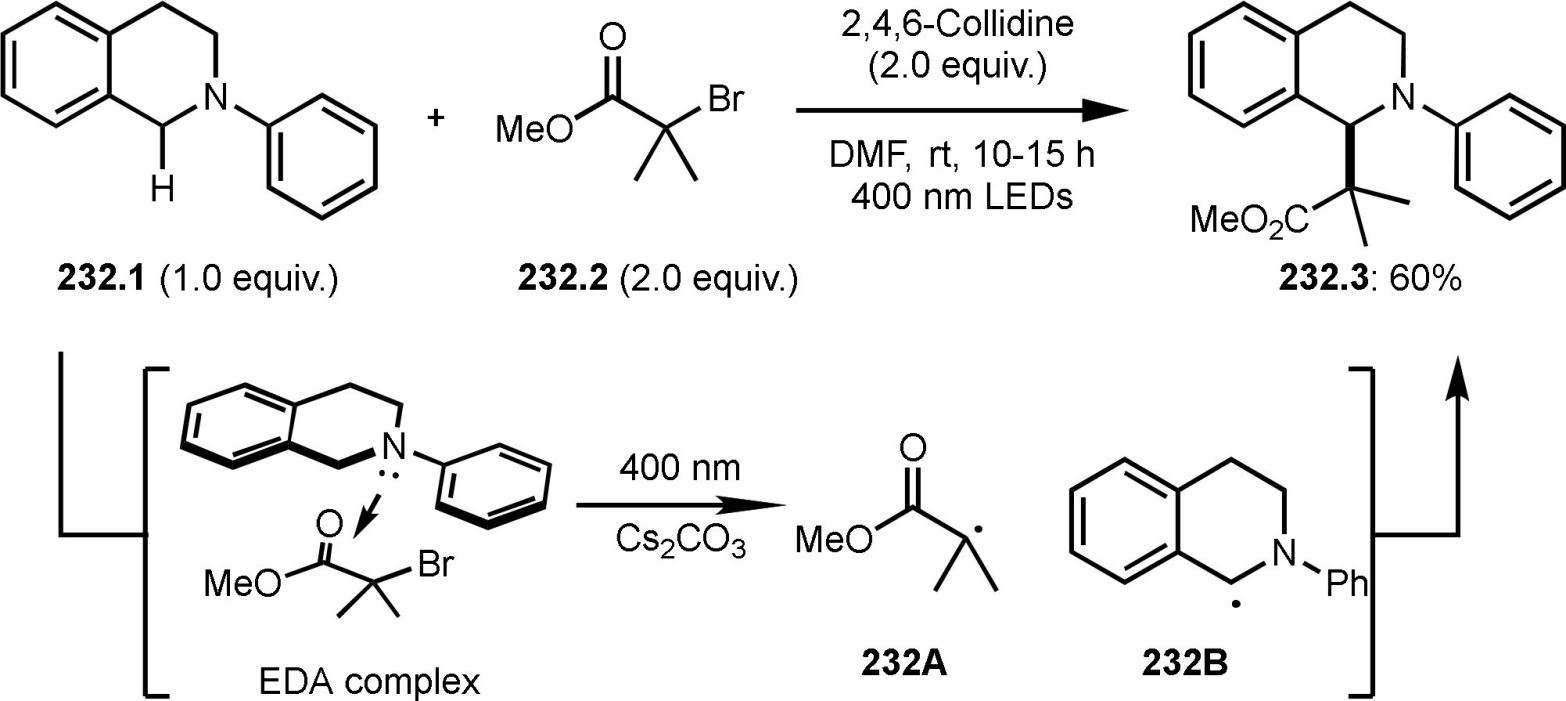
α‐*tert*‐Alkylation of aliphatic C−H bond.

α‐*tert*‐Alkyl radical species react not only with aromatic C−H bonds but also with aliphatic C−H bonds. Zhang's group reported the reaction of N‐phenyl tetrahydroisoquinoline (**232.1**) with α‐bromoester (**232.2**) to produce C−H tert‐alkylated tetrahydroisoquinoline (**232.3**) (Scheme 232).^[458]^ The reaction was performed under light irradiation and did not require a metal catalyst or any special additives to replace the C−H bond in **232.1** with a *tert*‐alkyl group. The key to this reaction is the formation of an EDA (electron‐donor‐acceptor) complex between **232.1** and **232.2**, in which radicals (**232A** and **232B**) were formed. Finally, radical‐radical coupling of **232A** and **232B** occurred, producing **232.3** in 60 % yield.

## 13 Summary and Outlook

The renaissance of radical chemistry since around 2010 has led to the construction of a complex radical reaction system that combines the classical radical chemistry of the past with new methods (ionic and organometallic reactions). At the same time, the use of tertiary alkyl groups, which had been difficult to introduce and had only a few types of reactions, also progressed. In particular, organic synthetic reactions using functionalized α‐bromocarbonyl (chloro−) compounds as a tertiary alkyl source have enabled a wide variety of reaction formats. That is, α‐bromocarbonyl compounds act as nucleophiles as well as electrophiles. This variable reactivity of α‐bromocarbonyl compounds is due to the reactivity of the active C−Br bond. In reactions with metal catalysts, Et_3_B/O_2_, or photocatalysts, α‐*tert*‐radicals are readily formed via SET. The radical species generated react either directly with unsaturated bonds or recombine with metals to react as organometallic species. Depending on the structure of the α‐bromocarbonyl compounds, the reaction with a base will give rise to aziridinone or (aza)oxy‐allyl cation, which is highly electrophilic. Since α‐bromocarbonyl compounds have a carbonyl group, the structure can be converted to the desired functional group after the reaction, and quaternary and tetrasubstituted carbon compounds can be easily synthesized. The convenience of this functional group conversion is also very powerful for the total synthesis of sterically congested useful molecules. As of 2022, reactions using α‐bromocarbonyl compounds have been expanded to include multicomponent, cross‐coupling, substitution, cyclization, rearrangement, stereospecific, and asymmetric reactions, realizing many types of reactions necessary for organic synthesis. On the other hand, reactions of α‐bromocarbonyl compounds, which are difficult to realize, are asymmetric reactions. In this paper, two modes of asymmetric reactions of α‐bromocarbonyl compounds were introduced. One is when a *tert*‐alkyl radical reacts with a nucleophile and the product has a chiral tetrasubstituted carbon, and the other is when the product has a chiral carbon other than the tertiary alkyl moiety. The former is difficult and there are few reported cases. Thus, innovative solutions are needed in the future. We hope that this review will lead to further developments in tertiary alkyl chemistry in the future.

## Conflict of Interests

The authors declare no conflict of interest.

14

## Biographical Information

*Takashi Nishikata (Ph. D.) was born in Tochigi, Japan in 1978 and appointed a full professor at Yamaguchi University in 2020. He completed his Ph. D. at Hokkaido University in 2005. He was also appointed ALCA‐Next researcher (from 2023) and also ERCA researcher (2024) (concurrent). He is the recipient of several honors and awards including: Thieme Chemistry Journals Award (2019), Incentive Award in Synthetic Organic Chemistry, Japan (2018), and Program to Disseminate Tenure Tracking System, MEXT, Japan: Personal selection (2013). His current interests are focused on the development of advancing methods for organic synthesis using metal‐catalyzed reactions or radicals*.

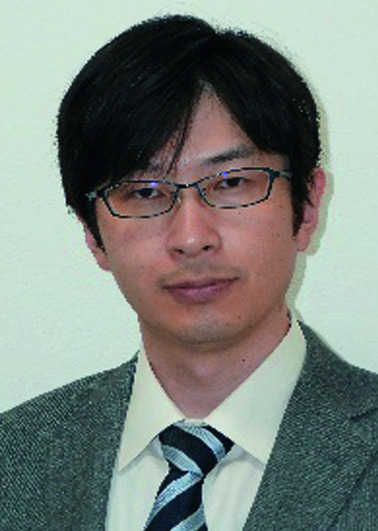

## Data Availability

The data that support the findings of this study are available on request from the corresponding author. The data are not publicly available due to privacy or ethical restrictions.
